# Abstracts of the 16th Annual Meeting of The Israel Society for Neuroscience Eilat, Israel, November 25–27, 2007

**DOI:** 10.1155/2007/30585

**Published:** 2008-01-01

## Abstract

The Israel Society for Neuroscience—ISFN—was founded in 1993 by a group of Israeli leading scientists conducting research in the area of neurobiology. The primary goal of the society was to promote and disseminate the knowledge and understanding acquired by its members, and to strengthen interactions between them. Since then, the society holds its annual meeting every year in Eilat usually during December. At this annual meetings, the senior Israeli neurobiologists, their teams, and their graduate students, as well as foreign scientists and students, present their recent research findings in platform and poster presentations, and the program of the meeting is mainly based on the 338 received abstracts which are published in this volume. The meeting also offers the opportunity for the researchers to exchange information with each other, often leading to the initiation of collaborative studies. Both the number of members of the society and those participating in the annual meeting is constantly increasing, and it is anticipated that this year about 600 scientists will convene at the Princess Hotel in Eilat, Israel.

Further information concerning the Israel Society for Neuroscience can be found at http://www.isfn.org.il.

*Committee*:

**Shlomo Rotshenker** (President)

Hebrew University of Jerusalem

**Talma Brenner**

Hadassah–Hebrew University Medical Center

**Yoram Rami Grossman**

Ben Gurion University of the Negev

**Mmouna Maroun**

University of Haifa

**Yoel Yaari**

Hebrew University of Jerusalem

**Gal Yadid**

Bar-Ilan University

**Zvi Wollberg** (Past President)

Tel Aviv University

**Lili Anglister** (Treasurer)

Hebrew University of Jerusalem

**Michal Gilady** (Administrator)

Rishon Le Zion

Aggregation of the amyloid-beta (A-beta) peptide in the extracellular space of the brain is central to Alzheimer's disease (AD) pathogenesis. Recent findings have shown that synaptic activity promotes release of A-beta through vesicle exocytosis. However, the relationship between vesicular release of A-beta and neurotransmitter release remains obscure. Current hypothesis of AD suggests that elevated neuronal activity leads to increased extracellular concentration of A-beta, which in turn triggers reduction in the number, strength, and plasticity of synaptic connections, eventually resulting in memory impairments. According to this hypothesis, decreased synaptic activity should promote memory function through reducing A-beta level. However, on the other hand, memory-related forms of synaptic plasticity are manifested as increase in synaptic activity. To understand this paradox, we study the relationship between different patterns of synaptic activity, A-beta release, and synaptic transmission using FM-based functional imaging at the level of single synapse and in synaptic networks. Our results show that acute accumulation of endogenously released A-beta enhances presynaptic activity for single spikes that mimic background activity through increasing release probability and the number of functional presynaptic terminals. However, A-beta does not affect presynaptic activity associated with temporally correlated burst of spikes. These results suggest that A-beta might be a key molecule transforming hippocampal synapses to a low-pass filter mode, resulting in decreased short-term facilitation of transmitter release during bursts. We hypothesize that elevation of background presynaptic activity by A-beta might be a trigger for homeostatic down-regulation in the number and plasticity of synapses and, eventually, memory decline in AD.
