# Supplementary material for: Abstracts of the 16th Annual Meeting of The Israel Society for Neuroscience Eilat, Israel, November 25–27, 2007
Source: Neural Plast. 2008 Jan 1;2007:30585. doi: 10.1155/2007/30585 (PMC2366074; doi:10.1155/2007/30585)
Supplement: Related texts

# Abstract: Protective effect of N-acetyl-L-Carnosine, a lipophilic derivative of L-Carnosine, on lipopolysaccaride-induced glial oxidative stress

**Authors:** C. Abramovitch-Dahan, E. Beit-Yannai, S. Ben-Shabat, S. Fleisher-Berkovich

**Affiliations:** Department of Clinical Pharmacology, Faculty of Health Sciences, Ben Gurion University of the Negev, Israel

Neurodegenerative disorders pathology can be characterized by both chronic inflammation and oxidative stress. Themost dominant source of inflammatory and oxidative stress mediators like nitric oxide (NO), prostaglandins, and cytokines are activated microglia. We used lipopolysaccharide (LPS) in order to induce oxidative stress in microglia. L-Carnosine is known as an efficient endgenous antioxidant. The aim of this study was to gain insight into the role of L-Carnosine and NAcetyl- L-Carnosine (NACar), a lipophilic synthetic derivative of L-Carnosine, on NO synthesis inmicroglia. It is shown here for the first time that L-Carnosine (10–20 mM) can inhibit LPS-induced NO synthesis by 75% in microglia. NACar was even more potent than its parent drug, L-Carnosine, in inhibiting LPS-induced NO synthesis. It inhibits NO release by 40% to 80% at concentration as low as 0.5 mM. Our results suggest that L-Carnosine and NACar may play a protective role in oxidative stress process in the brain. Further studies are needed aiming to understand the mechanism by which L-Carnosine and NACar act and to use these compounds as a potential treatment strategy for strategy for neurodegenerative diseases.

# Abstract: Enhanced stress-induced responses in an atroglial NOS2 mouse mutant

**Authors:** Y. Abu-Ghanem, H. Cohen, Y. Amitai, E. Grauer

**Affiliations:** ^1^Department of Physiology, Faculty of Health Sciences, Ben-Gurion University Beer-Sheva, Israel; ^2^Mental Health Center, Ben-Gurion University, Beer-Sheva, Israel; ^3^Israel Institute for Biological Research, Ness-Ziona, Israel

Nitric oxide (NO) is produced in the brain by both neurons and astrocytes. Until recently, astrocytic NO production has been demonstrated mainly as a slow reaction to detrimental events through the activity of an inducible NOS isoform (iNOS). Prior data from our laboratory described for the first time rapid astrocytic NO release in brains of healthy animals. To further explore the role of astrocytic-produced NO, we examined the iNOS knockout (KO) mouse. NO imaging and biochemical assays revealed compensatory NOS activity in mutants' brain, which resulted in 30% higher basal NO levels compared with control animals. Evaluation of the mutants' behavior in several behavioral tests revealed increased anxiety-like responses, with no differences in motor activity (Buskila et al., 2007). To further characterize the behavioral phenotype, we compared the performance of mutant mice to their controls in their response to stress. Seven days after exposure to predator scent stimulation (PSS), mutants exhibited higher anxiety-like behavior in the elevated-plus maze and stronger acoustic startle response. No such differences between groups were observed without the stress exposure. Plasma corticosterone levels were significantly higher in mutant mice following PSS, further demonstrating their heightened sensitivity to stress. Interestingly, mutant mice performed better on the Morris water maze (MWM) under control conditions. The possibility of improved cognition in these mice was not supported as these mutants failed to show improved performance in the object recognition test. The MWM results are probably due to enhanced motivation under water maze stress conditions in mutant mice compared to controls. Taken together, our results confirm higher susceptibility to stressful conditions in an astrocytic NOS mouse mutant.

*Supported by a grant from the Israel* *Science Foundation to Y.A.*

# Abstract: The role of CB1 receptors in the hippocampus and nucleus accumbens in extinction learning and neural plasticity

**Authors:** H. Abush, I. Akirav

**Affiliations:** Department of Psychology, University of Haifa, Israel

The inability to extinguish maladaptive fear responses is a hallmark of anxiety disorders, particularly posttraumatic stress disorder. Thus, suppression of the fear response via the behavioral paradigm of extinction receives increasing attention, since it could become an effective intervention for treating fear-related disorders. The endocannabinoid system, particularly CB1 receptors, has recently emerged as considerably important in the extinction of fear-related learning. However, interest in their therapeutic applications has been restrained by potentially harmful consequences on memory. Given the well-established role of the hippocampus in learning and memory processes, it is likely that the adverse effects of cannabinoids on memory are attributable to their actions within this brain region. The nucleus accumbens represents a brain area that is critical to the expression of rewarding and addictive properties of several classes of abused drugs including cannabinoids. Thus, we set out to examine the role of CB1 receptors in extinction learning and plasticity in the hippocampal-accumbens pathway. To that end, we have used 2memorymodels: light-dark inhibitory avoidance (IA) and LTP. We found that microinjecting the CB1 antagonist AM251 (0.5 *μ*g/0.5 mL) to the hippocampus impaired extinction, but not acquisition of the IA task. However, the direct cannabinoid agonist WIN 55 212-2 (0.5 mg/kg or 6 ng/0.5 mL) had no effect on the acquisition or extinction of IA in the hippocampus, but impaired LTP in the hippocampal CA1 area and in the CA1/subiculum-NAc pathway. Importantly, using an inhibitor of cannabinoid reuptake and breakdown AM404 (200 ng/0.5 mL) was found to facilitate extinction of IA. These observations are consistent with cannabinoid effects on memory, and suggest a twofold effect of cannabinoids on memory within the hippocampus: impairing and facilitating. This effect seems to be dependent on the memory task under examination.

# Abstract: Fine mapping of a schizophrenia susceptibility locus on chromosome 10q23-q26: significant evidence for linkage and a reduced linkage interval

**Authors:** A. Alkelai, Y. Kohn, K. Kanyas, A. Hamdan, D. Lancet, B. Lerer

**Affiliations:** ^1^Biological Psychiatry Laboratory, Department of Psychiatry, Hadassah, Jerusalem, Israel; ^2^Regional Mental Health Center, Taibe, Israel; ^3^Department of Molecular Genetics, Weizmann Institute of Science, Rehovot, Israel

**Introduction**

A genome scan for schizophrenia related loci in Arab Israeli families detected significant evidence for linkage at chromosome 6q23. Subsequent fine mapping, association, and replication studies identified AHI1 as a putative susceptibility gene. The same genome scan revealed suggestive evidence for a schizophrenia susceptibility locus in the 10q23-26 region.

**Methods**

To refine the linkage interval on chromosome 10q and to seek candidate genes in this region, we genotyped 35 additional microsatellite markers in the previously studied Arab Israeli sample, covering a 42.6 cM region between markers D10S583 and D10S217. Linkage and haplotype analyses were performed.

**Results**

The strength of the linkage on chromosome 10q increased from a multipoint, parametric HLOD score of 2.77 to 3.42. The one-unit support interval surrounding this peak decreased from 32.3 cM to 3.79 cM. 35 out of 40 affected individuals that were found to share the same allele of marker D10S88 for which the best HLOD score was found. In addition 26 out of 41 affected individuals were found to share the same haplotype, which consists of D10S88 and D10S1269 markers.

**Conclusions**

This study revealed significant evidence for linkage of Arab Israeli families affected with schizophrenia to chromosome 10q25.1-25.2 with a substantially reduced HLOD-1 region. Haplotype analysis suggests a founder mutation between markers D10S543 and D10S168 (10q25.2). There are 17 protein-coding genes in HLOD-1 region and most of them are brain-expressed with potential pathophysiological relevance to schizophrenia. These findings are supported by those of Levinson et al. (1998) and Mowry et al. (2000) in the same region.

# Abstract: Modeling voltage-gated sodium conductances from cortical pyramidal neurons

**Authors:** M. Almog, M. Gurkiewicz, A. Korngreen

**Affiliations:** ^1^The Mina & Everard Goodman Faculty of Life Sciences, Israel; ^2^The Leslie & Susan Gonda Brain Research Center, Israel

Transmembrane protein mechanisms such as ion channels and their activity are at the essence of neuronal transmission. The most accurate method, so far, for determining ion channel kinetic mechanisms is single-channel recording and analysis. Nevertheless, single-channel recordings carry several holdups and complexities, especially when dealing with voltage-gated channels. We have previously developed a method for fitting cell-attached and whole-cell voltageclamp data to kinetic models of ion channels using genetic search algorithms (GAs). Here, we show the application of this method for testing several previously published and newly conceptualized kinetic models to voltage-gated sodium channels currents recorded, in cell-attached configuration of the patch-clamp technique, from layer 5 pyramidal neurons of the rat cortex. The results emphasize the inability of the commonly used Hodgkin-Huxley paradigm to match and reproduce recorded currents from mammalian cortical neurons. Further, initial tests suggest a trend towards a simplified Markov scheme that adequately reproduces measured sodium currents. Finally, experiments were performed to test the robustness of the best putative Markov models, using Monte Carlo simulations.

# Abstract: Mitochondrial DNA HV lineage increases the susceptibility to schizophrenia among Israeli Arabs

**Authors:** S. Amar, A. Shamir, O. Ovadia, M. Blanaru, A. Reshef, I. Kremer, M. Rietschel, W. Maier, R. Belmaker, R. Ebstein, G. Agam, D. Mishmar

**Affiliations:** ^1^Faculty of Health Sciences, Ben-Gurion University of the Negev, Beer-Sheva, Israel; ^2^Department of Life Sciences, Ben-Gurion University of the Negev, Beer-Sheva, Israel; ^3^Department of Psychiatry, Haemek Medical Center, Afula, Israel; ^4^Department of Psychiatry, University of Bonn, Bonn, Germany; ^5^Department of Psychology, Hebrew University, Jerusalem and S. Herzog Memorial Hospital, Israel

Haplotypes and haplogroups are linked sets of common DNA variants, acting as susceptibility or protective factors to complex disorders. Growing evidence suggests that dysfunction of mitochondrial bioenergetics contributes to the schizophrenia phenotype. We studied mitochondrial DNA haplogroups in schizophrenia patients. Since mitochondria are inherited from the mothers, we used healthy fathers as an ideal case-control group. Analysis of the distribution of mitochondrial haplogroups in schizophrenia patients compared to their healthy fathers (202 pairs) resulted in an overrepresentation of the mtDNA lineage cluster, HV, in the patients (*P* = .01), with increased relative risk (odds ratio) of 1.8. Since mitochondrial DNA is small relative to nuclear DNA, a total mitochondrial genome analysis was possible in a hypothesis-free manner. However, mitochondrial DNA haplogroups are highly variable in human population and it will be necessary to replicate our results in other human ethnic groups.

# Abstract: Perception from a blind perspective: multisensory integration and sensory substitution in blind and sighted individuals

**Authors:** A. Amedi

**Affiliations:** Department of Physiology-Faculty of Medicine & Programof Cognitive Science, The Hebrew University, Israel

Restoration of sight in a blind person imposes great clinical and scientific challenges. Despite intensive efforts, recovery of vision using neuroprostheses has not been achieved. A major reason for this failure might be that the brain in the blind undergoes profound plastic changes and we do not know enough about vision and about how to communicate with this altered cortex to generate meaningful visual perception. In this presentation, new findings regarding the nature of sensory representations and verbal memory information in sighted, blind, and week long blindfolded subjects will be presented. These studies show that interactions between sensory modalities are critical to our understanding of sensory perception in the brain. These findings also show that massive brain plasticity in the occipital cortex is possible during development but possible even in the adult visual cortex. Here, I will highlight the role of transcranial magnetic stimulation (TMS) as a tool to assess the functional relevance of these plastic changes. Finally, I will discuss the use of sensory substitution devices (SSDs) in the context of blindness. In SSDs, visual information captured by an artificial receptor is delivered to the brain of a blind person using nonvisual sensory information via a human-machine interface. I will show that the use of an auditory-to-visual sensory substitution device called “the vOICe” yields successful performance on object recognition tasks, and specific recruitment of ventral and dorsal visual structures both in blind and sighted experts. I will close by discussing the importance of “the vOICe” as a device to be used for daily activities (e.g., object recognition and localization) and its potential use to “guide” the visual cortex to “read” and interpret visual information arriving from a retinal prosthesis.

*A. A. is supported by the Human Frontiers Science Program and an EU 7th Framework Program grant*.

# Abstract: The N170 sensitivity to faces is dissociated from conscious awareness

**Authors:** I. Amihai, L. Y. Deouell, S. Bentin

**Affiliations:** ^1^Neurobiology Department, Hebrew University of Jerusalem, Israel; ^2^Psychology Department, Hebrew University of Jerusalem, Israel

Converging evidence from numerous ERP and MEG studies points to a neural response which discriminates faces from nonface objects, peaking at around 170 milliseconds following stimulus onset. Here we ask whether this discrimination at the neural level correlates with conscious detection of a face, or with its mere presence in the visual field. To investigate this, we used “continuous flash suppression,” a binocular rivalry technique that effectively causes unawareness of the stimulus presented to one eye. In each trial, a face or an Easter egg was continuously presented to one eye, but was temporarily masked after a delay by a dynamic stimulus presented to the other eye. In a control condition, the face or egg was physically removed for the duration of the dynamic mask. In both conditions, the subjects' subjective experience was of two presentations of a face or of an egg. The onset of the first face elicited a typical N170 face-specific response in both conditions. In contrast, the subjective reappearance of the face elicited a face-specific N170 response only in the control condition, when physical face onset was introduced. Thus, extrastriate object-selective responses reflect the physical presence of relevant input, and are not correlated with conscious awareness.

# Abstract: The effects of withdrawal from chronic administration of estradiol on “compulsive” lever-pressing of female rats—a preliminary study

**Authors:** M. Arad, N. Albelda, D. Joel

**Affiliations:** Department of Psychology, Tel-Aviv University, Israel

Obsessive-compulsive disorder (OCD) is a psychiatric disorder psychiatric disorder affecting 1–3% of the population and characterized by intrusive unwanted thoughts (obsessions) and repetitive ritualistic behaviors (compulsions). Sex differences have been found in the phenomenology and course of this disorder, and women have been reported to be vulnerable to an onset or exacerbation of symptoms during premenstruum, pregnancy and postpartum. This suggests a possible involvement of gonadal hormones in the pathopysiology of OCD. In the signal attenuation rat model of OCD, “compulsive” behavior is induced by attenuating a signal indicating that a lever-press response was effective in producing food. The present study tested the effects of chronic administration and of withdrawal from chronic administration of 17 beta estradiol on compulsive lever-pressing of pre-pubertal female rats, in an attempt to mimic late gestation and postpartum. In order to assess the efficacy of the hormone regimen in mimicking the postpartum period, rats were assessed for postpartum depression using the “forced swim test” (FST), an animal model of depression. Rats that were injected with 17 beta estradiol for 7 days and then underwent a withdrawal phase for 3 days tended to exhibit more compulsive lever-pressing compared to control animals (*P* < .08). In the FST, the “withdrawal” rats exhibited significantly more immobility, the depressive-like behavior, than control rats, indicating that the hormone regimen was successful in mimicking the post-partum period. The results suggest that fluctuations in estradiol levels can lead to the onset or exacerbation of OC symptoms.

# Abstract: Amphetamine and ovariectomy induced disruption of latent inhibition in female rats can be reversed by haloperidol only in the presence of estrogen

**Authors:** M. Arad, I. Weiner

**Affiliations:** Department of Psychology. Tel-Aviv University, Israel

Elevated relapse rates and symptoms remission in schizophrenic schizophrenic women tend to correlate with low and high estrogen levels, respectively. Consequently, the estrogen hypothesis of schizophrenia postulates that estrogen is a neuroprotective factor delaying the onset of schizophrenia and reducing its severity in women. Latent inhibition (LI), the capacity to ignore stimuli that received nonreinforced preexposure prior to conditioning, is disrupted in acute schizophrenia patients and in rats and humans treated with the psychosis inducing drug amphetamine. Disruption of LI is reversible by typical and atypical antipsychotic drugs (APDs). Our previous results suggested that LI was disrupted in ovariectomized (OVX) rats and could be restored by 17 beta-estradiol (150 *μ*g/kg) and the atypical APD clozapine (5 mg/kg), but was resistant to the typical APD haloperidol (0.1, 0.2, 0.3 mg/kg). Here we show that haloperidol is able to restore LI only when administered with 17 beta-estradiol (50 *μ*g/kg). Furthermore, under conditions that yield LI in both OVX and sham female rats, amphetamine disrupts LI in all rats, but restoration of LI by haloperidol in amphetamine-treated rats is observed only in sham rats. While resistance of disrupted LI to haloperidol in the absence of estrogen is in line with reduced efficacy of haloperidol in menopausal women, our results suggest that propsychotic action of dopamine (DA) releaser apparently does not require estrogen. This differential sensitivity of OVX rats to DA stimulation and DA blockade may have important implications for the clinical development of schizophrenia and APD treatment in women because it suggests that naturally occurring reduction in hormonal levels may increase the danger of relapse/severity of symptoms while concomitantly reducing the efficacy of conventional treatment.

# Abstract: A novel approach for treatment of Alzheimer's disease: antibodies against beta-secretase cleavage site of APP

**Authors:** M. Arbel, B. Solomon

**Affiliations:** George S. Wise Faculty of Life Science, Tel-Aviv University, Israel

Although the extracellular A-beta plaques are the main hallmark and criteria for ultimately post mortem diagnosis of Alzheimer's disease (AD), it remains to be determined whether extracellular or intracellular A-beta accumulation initiates the disease process. Intraneuronal A-beta accumulation is increasingly linked with early preplaque synaptic and pathological abnormalities occur in brain of AD patients and transgenic animals. Understanding the mechanisms that underline A-beta accumulation in neurons and its relation with the extracellular A-beta pool, may be beneficial in developing therapeutic tools for AD treatment. We developed a novel approach to inhibit A-beta production via antibodies against the beta-secretase cleavage site of APP. This approach limits APP processing by beta -secretase, mainly through the endocytic pathway and overcomes some of the limitations of BACE1 (beta-secretase) inhibition methodologies. Antibetasite antibodies bind human APP expressed by AD cellular model and internalize into the cells after APP binding at the plasma membrane. Antibodies mediated inhibition of beta-secretase cleavage led to a 50% reduction of intracellular A-beta levels. Intracerebroventricular injection of these antibodies to non transgenic mice in which neprilysin activity was inhibited led to 60% and 80% reduction in intracellular and extracellular brain A-beta levels, respectively, highlighting the therapeutic potential of these antibodies. Moreover, systemic administration of antibeta-site antibody to tg2576 mice facilitated a beneficial effect on the mice cognitive function and did not lead to any adverse effects. To the best of our knowledge this is the first attempt to inhibit beta-secretase cleavage of APP by blocking the cleavage site upon the substrate rather then a total inhibition the enzyme. This approach overcomes some of the limitations presented by both BACE1 inhibition methodologies and A-beta-based immunotherapies.

# Abstract: Modalities of feedback induce differential reaching strategies in adaptations to force field

**Authors:** F. Arce, I. Novick, M. Shahar, C. Ghez, E. Vaadia

**Affiliations:** ^1^Department of Physiology, Hadassah Medical School, The Hebrew University, Jerusalem, Israel; ^2^Center for Neurobiology and Behavior, Columbia University College of Physicians and Surgeons, NY, USA; ^3^The Interdisciplinary Center for Neural Computation, The Hebrew University, Jerusalem, Israel

Performance errors induced by perturbation during reaching may drive adaptation differentially based on sensory modalities conveying them. Here we demonstrate that state estimates based on vision and proprioception differentially influence adaptive strategies used in force fields. We studied the contributions of visual and proprioceptive feedback in a reaching task. Separate groups of human subjects adapted to viscous force fields with or without concurrent visual feedback of their hand trajectory and were retested after 24 hours. We found comparable levels of endpoint accuracy after adaptation to force field with and without visual feedback, however, hand paths differed consistently. With visual feedback, induced deviations in initial direction of hand movements were rapidly reduced and paths became rectilinear. On day 2, adaptive changes in initial direction were accelerated and endpoint variability was reduced. Without visual feedback (and proprioception alone), hand paths remained highly curved. While endpoint errors were reduced on day 2, initial directions and curvature remained unchanged. The results indicate two different strategies to achieve final position accuracy-one through changes in trajectory and another through changes in endpoint variability. In the presence of combined visual and proprioceptive feedback, adaptation is dominated by control of trajectory and adjustments in direction. With proprioceptive feedback alone, compensation for the perturbed dynamics is achieved primarily by adaptation of final equilibrium position. We argue that such stark difference in the adaptive responses depends on the sensory sources of the estimates for trajectory and for final reach position. Our findings also support the notion that separate adaptive mechanisms operate to adjust trajectory and endpoint accuracy.

*Supported by BSF, ISF, Johnson & Johnson Fund for Innovative Science, Rosetrees Trust, Ida Baruch Fund*.

# Abstract: Using odors to treat sleep apnea: a test of feasibility

**Authors:** A. Arzi, L. Sela, R. Haddad, A. Green, Y. Dagan, N. Sobel

**Affiliations:** ^1^Department of Neurobiology, The Weizmann Institute of Science, Rehovot 76110, Israel; ^2^Assuta Hospital, Petach Tikva 49361, Israe

Apnea, a repeated suspension of breathing during sleep, is a prevalent sleep disorder with significant impact on daily life as well as on general health. We combined the following information to generate a potential treatment:

1. it is largely held that odorants fail to wake humans from sleep;
2. odorants modify respiratory patterns. Given this, we hypothesized that providing an odorant during apnea may “jumpstart” the respiratory pattern without waking the individual.

To address this, we first set out to test the influence of different odorant regimens on patterns of sleep and sleeprespiration. Subjects slept in a stainless-steel-coated odorant nonadherent room where we measured an electroencephalogram, electrooculogram, electromyogram, electrocardiogram, blood oxygenation, as well as overall and nasal respiration. Subjects wore a small nasal mask where we could deliver odorants in a controlled fashion, with no nonolfactory cues as to odorant onset and offset. The odorant lavender oil was presented to sleeping subjects (using ISIs of 9, 12, and 15 minutes, and stimulus durations of 5, 10, and 20 seconds). In an initial analysis of nasal respiration in the first subject, without odors we found a main effect of sleep stage (*F* = 49, *P* < .0001) reflecting equally vigorous nasal respirations during stage 1 and 2 sleep (*t* = 1.5, *P* = .14), and equally significantly reduced respiratory magnitude in SWS and REM (*t* > 6, *P* < .0001). Whereas odorant presentations had no influence on respiratory magnitude during Stages 1 and 2 sleep (*t* = 1.1, *P* = .27), they induced a significant recovery in magnitude of nasal respiration during SWS and REM (*t* = 3.5, *P* < .01), to levels even slightly higher than those seen at Stages 1 and 2 with and without odor. These pilot data point to feasibility of using odorants to treat sleep apnea.

* *These authors contributed equally to this work.*

# Abstract: Exposing rats to stressors during juvenility increases morphine intake in adulthood

**Authors:** M. Asbe, M. M. Tsoory, B. D. Berger

**Affiliations:** Department of Psychology, The Brain and Behavior Research Center, University of Haifa, Haifa, Israel

Epidemiological studies indicate that exposure to stress early in life increases the risk to develop both mood and anxiety disorders as well as drug dependence or drug seeking behaviors in adulthood. Exposing rats to stress during juvenility (27–29 days of age) was found to increase the prevalence of altered stress responses which corresponded to both mood and anxiety disorders' symptoms and thus was suggested as a model of induced predisposition for these disorders (Avital and Richter-Levin, 2005; Tsoory and Richter- Levin, 2006; Tsoory et al., 2007a). The current study utilized the juvenile stress model and examined its effects on morphine intake in adulthood. Consumption of water alone and/or water + morphine sulfate (0.5 mg/cc) was monitored over several weeks. In comparison with control rats, adult juvenile stressed rats exhibited a higher morphine preference (morphine intake/ (morphine + water intake)). Recent findings suggest that exposure to juvenile stress may alter development-related processes and possibly sensitize stress responses mechanisms (Tsoory et al., 2007b). Taken together with the current data, it is suggested that such sensitized stress responses mechanisms may increase drug seeking behaviors. These findings support the validity of an animal model for the study of the consequences of exposure to stress early in life regarding drug dependence or drug seeking behaviors in adulthood.

# Abstract: A Self-sustaining translational switch for regulating the maintenance of synaptic plasticity

**Authors:** N. Aslam, Y. Kubota, H. Shouval

**Affiliations:** The University of Texas Medical School at Houston, Tex, USA

Long term memory as well as synaptic plasticity outlast the lifetimes of the proteins that encode synaptic efficacies. Since synaptic plasticity is synapse-specific it is essential to identify a mechanism that can sustain changes in synaptic efficacies is a synapse specific manner. One key observation that might supply a possible mechanism is that the consolidation of long term memory and long term plasticity depends on the production of new proteins. Here we suggest that a feedback loop between a translation factor and a plasticity related kinase might provide this stability. Specifically we examine the CaMKII-CPEB pair as a possible instantiation of this idea. Experimental evidence suggests that during the normal brain function most of the CaMKII (83%) is synthesized in dendrites through local translation (Miller et al. 2002, Schuman et al. 2006). Here we propose that the synthesis of CaMKII at active synapses can be regulated through a local translational switch. The proposed translational switch might provide a self-sustaining mechanism to regulate the de novo synthesis of plasticity-related proteins during the maintenance phase of late long-termpotentiation (L-LTP). Our results show that a bistable switch can arise from CPEB mediated polyadenylation of *α*CaMKII mRNA. Our results imply that L-LTP should produce an increase in the total amount of αCaMKII and fraction phosphorylated at potentiated synapses, which is consistent with experimental observations (Ouyang et al. 1999). Through bifurcation analysis, we identify the key parameters that determine whether the system is in a bistable region, this could indicate the key parameters that should be measured experimentally. We also demonstrate that a partial block of αCaMKII translation in the induction phase of L-LTP can block L-LTP, but a partial block of translation in the maintenance phase might not block L-LTP.

# Abstract: Mapping the environment-an experimental model of spatial representation during exploration and navigation

**Authors:** R. Avni, D. Eilam

**Affiliations:** Department of Zoology, Faculty of Life Sciences, Tel Aviv University, Israel

We present a hierarchal model and supporting data on spatial representation during exploration, based on successive phases of spatial information processing: (1) piloting (sequential processing)—egocentric space representation constructed by traveling from one landmark to the next, changing heading direction (origin point for path integration) between landmarks; (2) orienting (parallel processing)—space is represented in relation to a fixed place that is a terminal for repeated sequential processing, with the traveling paths varying yet heading direction points to the fixed location (path integration calculated from that place); (3) navigating (continuous processing)—space representation (map) is constructed by orienting (continuously updating heading direction) in relation to several places. Empirically, we demonstrate a transition from sequential to parallel processing during gerbil exploration. Tested in an unfamiliar dark open field, gerbils explored it using a looping mechanism: traveling slowly in an undulating path that forms a loop by returning to a previously visited place. In looping the gerbils rarely returned more than once to a previously visited place, with no apparently fixed heading direction. Gradually, gerbils shifted from looping to a home base mechanism, spending increasingly greater time in one place and repeatedly returning to it. Exploration became organized in relation to a fixed place, with varying paths that share a heading direction computed in relation to that place. We suggest this shift from looping to home base to reflect a change from momentary to fixed heading direction and thus from piloting to the orienting phase of the model. As the model involves transition from orienting to navigating, a transition from home base to a higher level of spatial organization is predicted. Possible implications of the model to effect of spatial representation damaging (e.g., lesions) are yet to be explored.

*Supported by the Israel Science Foundation*.

# Abstract: Reorganized prospective and retrospective hippocampal memory coding after switching the start and goal of a journey

**Authors:** A. Bahar, M. Shapiro

**Affiliations:** Fishberg Research Center for Neurobiology, Mount Sinai School of Medicine, NY, USA

Rat Hippocampal neurons encode the current location of the animal via place fields. Changes to distal cues in the animal's environment can modulate this encoding, a phenomenon termed place field remapping. In a spatial memory task on a + maze hippocampal cell activity was also influenced by locations that were recently visited, or are forthcoming (i.e., expressing retrospective or prospective memory coding, Ferbinteanu and Shapiro, 2003). Thus, hippocampal neurons can jointly express contextual and mnemonic information that relates to the present, past, and future. This type of processing fits the view that the hippocampus supports episodic memory, and that memory demands can modulate hippocampal coding. The present experiment tested the effects of changing memory demands on retrospective and prospective coding, and examined if modulations of retrospective and prospective coding share the same dynamics as stimulus-induced place field remapping. We trained rats to perform a win-stay task with serial reversals on a + maze. Hippocampal unit activity was recorded as rats placed on either a North or a South start arm navigated to the correct East or West rewarded goal arm. In a novel condition, the start and goal arms were switched, so that the North and South arms served as the goal arms and the East and West arms served as the start arms. Preliminary data showed that all place fields remapped in the novel condition. Concurrently, performance decreased in the Novel condition from 91% to 74% correct, as did the percent of fields showing prospective or retrospective coding (30% to 15% and 50% to 8%, respectively). In contrast, the percent of fields coding location alone increased in the novel condition from 20% to 77%, though the fields were less stable. We are collecting additional data from CA1/CA3 fields in order to better understand the influence of changes to memory demand on hippocampal on-line processing.

# Abstract: Working memory: somatic spikes or synaptic calcium?

**Authors:** O. Barak, G. Mongillo, M. Tsodyks

**Affiliations:** ^1^Department of Neurobiology, Weizmann Institute of Science, Israel; ^2^Laboratory of Neurophysics and Physiology, UMR 8119, CNRS, Université René Descartes, France

The notion that synaptic plasticity supports long-termmemory storage while persistent neuronal spiking activity maintains these memories in a short-term active form is widely held in theoretical neuroscience. We show that synapses can also effectively implement active memory function through their short-term plasticity. In this account, the short-term synaptic variables are used as a “buffer” which is loaded, read-out and refreshed by neural activity. The refresh rate is low, since it depends on the slow time constants associated with synaptic dynamics. The feasibility of this mechanism is demonstrated in a biologically plausible spiking network model. The resulting “working memory” system is robust, metabolically efficient, and can help explain recent electrophysiological results.

# Abstract: Topology Regulates Source Separation in Biologically Realistic Neural Networks

**Authors:** E. Barak, D. Eytan, R. Meir, Y. Y. Zeevi

**Affiliations:** ^1^Faculty of Medicine, Technion-Israel Institute of Technology, Haifa 32000, Israel; ^2^Faculty of Electrical Engineering, Technion-Israel Institute of Technology, Haifa 32000, Israel

This study addresses the issue of relations that exist between topology and dynamics of large sparsely connected neural networks. In particular, it examines the hypothesis that topology governs the source separation ability of neural networks endowed with structural characteristics of cortical networks. For this purpose, extensive simulations of networks with different topologies and similar structural features (e.g., similar average in- and out-degrees) were carried out. Networks responses to various stimuli were analyzed in terms of neural recruitment order during a network burst. The latter measure is assumed to convey information relevant to source separation. The results showed that the responses of networks with Gaussian in-degree and Scale Free (SF) out-degree distributions to different stimuli varied significantly more than those of Erdös Rényi (ER) networks and networks with SF in-degree and Gaussian out-degree distributions. These results support the hypothesis that topology does indeed play a crucial role in source separation tasks executed by neural networks.

# Abstract: AxCaliber—in-vivo measurement of axon diameter distribution with MRI

**Authors:** D. Barazany, P. Basser, Y. Assaf

**Affiliations:** ^1^Department of Neurobiochemistry, Faculty of Life Sciences, Tel Aviv University, Israel; ^2^The National Institutes of Health, Bethesda, Md, USA

The axon diameter distribution (ADD) is an important anatomical feature of nerve fascicles both in normal and abnormal development. Axon diameter directly affects nerve function. It is well known that in myelinated axons, the conduction velocity is directly proportional to axon diameter. Thus, large-diameter axons generally arise in pathways that require short conduction times while smaller axons arise in pathways that can tolerate longer conduction times. In addition, it is hypothesized that in amyotrophic lateral sclerosis (ALS) large diameter axons are damaged selectively, while in autism, small-diameter axons are over-expressed. Despite its importance, the ADD within nerve fascicles has not been measurable in-vivo, and currently can only be assessed by invasive histological means. Here, we propose a new magneticresonance- imaging- (MRI-) based approach called AxCaliber that utilizes diffusion MRI to extract the ADD, in-vivo and noninvasively. Diffusion MRI measures the micron-scale displacements of water molecules; by altering the diffusion time and diffusion weighting morphological parameters of the tissue, such as the ADD and axonal density, can be measured experimentally. In this work, AxCaliber was used to extract the ADD within the corpus callosum (CC) of the rat. Using AxCaliber we were able to segment the CC to at least 3 distinct regions corresponding to the body, genu, and splenium of the CC. A narrow ADD with a mean of about 1 micron characterized the genu and splenium while the body of the CC had a much broader distribution with a mean of about 4-5 microns. This segmentation resembles the known morphological arrangement of the CC as measured from histology. Applications of AxCaliber are expected in longitudinal studies designed to follow nerve growth in normal and abnormal development, as well as in diagnosing disorders and diseases affecting specific populations of axons in the CNS and PNS.

# Abstract: Akt is differently activated in the amygdala-prefrontal cortex-insular cortices circuit in acquisition and extinction of CTA

**Authors:** A. Bareni, M. Maroun, K. Rosenblum

**Affiliations:** Department of Neurobiology and Ethology, Center of Brain and Behavior, University of Haifa, Israel

In conditioned taste aversion (CTA) a single gustatory experience (exposure to novel taste) is followed by visceral trauma. Consequently, the animal develops an aversion for the aversive taste. The memory of aversion is robust, however, repeated exposures to the taste without the association with the malaise leads to extinction of CTA. It is assumed that consolidation and extinction of CTA are each subserved by different neuronal mechanism and various parts of the brain are involved in these mechanisms. The neural circuit that underlies the acquisition and the extinction of CTA includes the amygdala and the insular cortex. Recently research from our laboratory has shown that the ventromedial prefrontal cortex (vmPFC) has a role in extinction of CTA. In the current study, we examined the differential activation of the phosphorylation levels of Akt, a serine/threonine kinase, which is a critical enzyme in signal transduction pathways, in the amygdala-prefrontal cortex-insular cortices circuit during acquisition and extinction of CTA. Our results show that following CTA acquistion there is an increased level of AKT phosphorylation in the amygdala, insular cortex and the prefrontal cortex. In contrast, following extinction training there was a decrease of Akt phosphorylation in the amygdala and PFC while no change was found in the IC. These results hint on the possibility that the amygdala-PFC-insular cortex circuit is differentially involved in acquisition and extinction of CTA.

# Abstract: Differences in early visual processing in synesthesia: an ERP study

**Authors:** K. J. Barnett, J. J. Fox, S. Molholm, S. P. Kelly, S. Shalgi, K. J. Mitchell, F. N. Newell

**Affiliations:** ^1^Institute of Neuroscience (TCIN) and School of Psychology, Trinity College, Dublin 2, Ireland; ^2^Cognitive Neurophysiology Lab, Nathan Kline Institute for Psychiatric Research, USA; ^3^Department of Genetics, Trinity College, Dublin 2, Ireland

Stimuli that elicit synesthesia (e.g., letters of the alphabet) activate cortical color regions of the brain, including V4. While it has been demonstrated that synesthesia is a genuine perceptual phenomenon, its underlying neural substrates are not well understood. For example, the overall integrity of the visual system has never been assessed and it is not known whether differences in sensory-perceptual processing are associated with the condition. Here, we looked at visual evoked potentials (VEPs) to determine differences in sensory-perceptual processing using stimuli that differentially activate the magnocellular and parvocellular pathways of the visual system. High and low spatial frequency gratings (Gabors) and luminance-contrast squares were presented to 15 synesthetes and 15 controls. We report for the first time, early sensory-perceptual differences in synesthetes. The differences were associated with stimuli that do not elicit synesthetic color experiences and were manifest in early C1 and P1 components of the visual evoked potential (VEP). The findings were not confined exclusively to either magnocellular or parvocellular stimuli, but rather indicate widespread differences in the way that synesthetes process visual information during early time windows.

# Abstract: Modulation of dendritic calcium spike by extracellular calcium concentration

**Authors:** D. Bar-Yehuda, H. Ben-Porat, A. Korngreen

**Affiliations:** ^1^The Mina and Everard Faculty of Life Sciences, Israel; ^2^The Leslie and Susan Gonda Brain Research Center, Bar-Ilan University, Israel

It has been shown that in the apical dendrite of L5 pyramidal neurons, when a back-propagating action potential coincides with distal synaptic input, a dendritic calcium spike is generated, leading to the generation of a burst of action potentials at the soma. Many questions have been raised regarding the physiological role of this calcium spike. Lately, a different question has been raised whether the calcium concentration being used in the typical ACSF truly reflects the concentration in the CSF. Recent studies have shown that the calcium concentration in the ACSF is higher than in the CSF in vivo. We have performed in vitro recordings from acute brain slices, using the whole-cell configuration of the patchclamp technique in the current-clamp mode from L5 pyramidal neurons. Regenerative calcium spikes were recorded and compared with calcium concentrations similar to the ACSF and CSF in the bath solution. Our results suggest that while the back-propagating action potential is robust under reduced extracellular calcium concentration, the dendritic calcium spike is suppressed by this modification.

# Abstract: Lentiviral delivery of LMX1a into human Mesenchymal Stem Cells directs towards dopaminergic differentiation

**Authors:** R. Barzilay, T. Ben-Zur, S. Bulvik, E. Melamed, D. Offen

**Affiliations:** ^1^Neuroscience lab, Felsenstein Medical Research Center, Sackler Faculty of Medicine, Tel Aviv University, Israel; ^2^Laniado Medical Center, Netanya, Israel

Human mesenchymal stem cells reside in the bone marrow and are known for their ability to differentiate along the mesenchymal lineage (fat, bone, and cartilage). Recent works suggest the possibility that these cells are also capable of differentiating towards the neuroectodermal lineage. We aimed at inducing dopaminergic differentiation on human mesenchymal stem cells as a possible source for autologous transplantation for Parkinsonian patients. Using lentiviral gene delivery, we sought to reprogram the bone marrow derived mesenchymal stem cells towards dopaminergic differentiation through delivery of LMX1a, which was reported to be a key player in dopaminergic differentiation in both developmental animal models and embryonic stem cells. Transduction of cells with fluorescent reporter genes confirmed efficiency of gene delivery. Upon incubation of the LMX1a transduced cells in differentiation medium we observed increased concentration of the LMX1a transcription factor in the cell nuclei. Several specific dopaminergic developmental genes were upregulated suggesting the generation of dopaminergic precursors derived from adult human bone marrow. Moreover, the transduced cells secreted significantly higher level of dopamine in comparison to untransduced cells. To our knowledge, this is the first time in which a factor involved in the natural dopaminergic neuron development directs adult stemcells towards the dopaminergic phenotype. Using ex vivo gene delivery to adult stem cells, our results shed a new light on the molecular processes involved in adult stem cell dopaminergic differentiation, suggesting that the transcriptional cascade of adult stem cell dopaminegic differentiation has parallel pathways to that of embryonic stem cells.

# Abstract: Activation of the amyloid cascade in ApoE4 transgenic mice induces neurodegeneration resulting in marked cognitive deficits

**Authors:** H. Belinson, I. Dolev, D. Lev, E. Masliah, D. M. Michaelson

**Affiliations:** ^1^Department of Neurobiochemistry, Tel Aviv University, Israel; ^2^Department of Neurosciences & Pathology University of California San Diego, LaJolla Ca 92093-0624, USA

The allele E4 of apolipoprotein E (apoE4), which is the major genetic risk factor for Alzheimer's disease (AD), is associated histopathologically with elevated levels of brain amyloid. This led to the suggestion that the pathological effects of apoE4 are mediated by cross talk interactions of apoE4 with A beta and to the consequent accentuation of the pathological effects of the amyloid cascade. The A beta related processes which are affected by apoE4 and the cellular and molecular mechanisms which underlie their pathological consequences are not known. We have recently shown that inhibition of the A beta degrading enzyme neprilysin by intracerebroventricular infusion of thiorphan into brains of wild type apoE3 and apoE4 mice, results in a rapid and similar increase of their total brain A beta levels. However, the nucleation and aggregation of A beta in these mice were markedly affected by apoE and were particularly and specifically enhanced in the apoE4 mice. We presently employed the neprilysin inhibition paradigm to analyze the brain pathological and cognitive effects which are induced by apoE4 and apoE3 at distinctive time intervals following activation of the amyloid cascade and of the extent to which they correlate spatially and temporally with the accumulation of intraneuronal A beta. This revealed that apoE4 stimulates isoform specifically the accumulation of intraneuronal A beta in hippocampal CA1 neurons. Furthermore, the accumulation of A beta in the CA1 neurons correlates spatially and temporarily (t 1/2 ∼ 1 week) with enhanced lysosomal activation and apoptosis of these neurons and with the occurrence of marked cognitive deficits. These findings render this model uniquely suitable for detailed kinetic analysis of the early stages of the amyloid cascade and of the biochemical mechanisms underlying the effects thereon of apoE4 and other risk factors of AD.

*Supported by The US-Israel Binational Foundation and the Joseph and Inez Eichenbaum Foundation*.

# Abstract: Secreted factor frommutant PC12 cells as a potential element for neuronal survival

**Authors:** S. Beltsov, R. Yaka

**Affiliations:** Department of Pharmacology, School of Pharmacy, The Hebrew University of Jerusalem, Israel

Rat pheochromocytoma (PC12) cells have been extensively studied as a model for neuronal cell line since their first cloning in 1976, because of their neuronal-like properties. Removal of the serum from the growth medium of PC12 wild type (PC12-wt) cells induces cell death by apoptosis. A mutant clone of PC12 cells (PC12-m), can survive up to 5 days in a serum-free medium. Furthermore, it has been shown that PC12-m cells secrete an unknown factor that prevents PC12-wt cells from serum deprivation-induced cell death; PC12-m cells were grown in a serum-free medium for 5 days and the condition medium (CM) was collected. When PC12-wt cells were grown in CM, they survived for up to 5 days. Here we show that the survival of PC12-wt cells in the CM was prevented when an inhibitor of the TrkA receptor, K252a, was added together with the CM, thus showing that cell survival is mediated via TrkA receptor. This factor present in the CM is probably not NGF (which previously has been shown to rescue PC12 cells from serum deprivation-induced death), because boiling of the CM for 10 minutes did not prevent PC12-wt survival capabilities, as opposed to boiling of serum-free medium with NGF. Unlike NGF, acute treatment of PC12-wt cells with CM failed to induce ERK activity, suggesting that the secreted factor acts via a mechanism different than NGF. Interestingly, CM from PC12-m cells also prevented cell death of PC12-wt cells induced by high concentration of cocaine, independent of the TrkA receptor. These results suggest that the CM has neuroprotective abilities; however, the signaling pathways which underlie these abilities are different from those induced by NGF, and dependent on the type of insult used.

# Abstract: Distinct pore properties of leak and voltage-activated potassium channels underlie their unique roles in electrical signaling

**Authors:** Y. Ben Abou, N. Zilberberg, O. Yifrach

**Affiliations:** Department of Life Sciences, Zlotowski Center for Neuroscience, Ben-Gurion University, Israel

Voltage-activated (Kv) and leak (K2P) potassium channels play key yet distinct roles in electrical signaling in the nervous system. Here, we examined how differences in the operation of the activation and slow inactivation pore gates of Kv and K2P channels underlie their unique roles in electrical signaling. Shaker Kv and KCNK0 leak channels demonstrate opposite closed and open pore conformational stability phenotypes, respectively. Replacement of the pore domain of the Shaker channel with that of KCNK0 resulted in a substantial stabilization of the open pore conformation. Systematic replacement of activation gate hydrophobic residues of the Shaker channel to the corresponding glycine residues of KCNK0 resulted in dramatic open-state stabilization effects. Complementary experiments revealed dramatic closed state stabilization effects. By using macroscopic and single channel recordings we were able to monitor the conformational states (whether closed or open) of both the activation and inactivation gates of KCNK0. Our results revealed that (1) leak potassium channels also possess a lower activation gate, (2) that the activation gate is an important determinant controlling the intrinsic conformational stability of the potassium channel pore, (3) the lower activation and upper slow inactivation gates of leak channels cross-talk and (4) in contrast to Kv channels, where the two pore gates are negatively coupled, that is, the opening of the lower activation gate stimulates the closure of the upper slow inactivation gate, the two gates are positively coupled in K2P channels. Our results demonstrate how basic thermodynamic properties of the potassium channel pore, particularly, intrinsic conformational stability and coupling between the pore gates, underlie the specialized roles of Kv and K2P channel families in electrical signaling.

# Abstract: White matter impairment in amyotrophic lateral sclerosis (ALS): diffusion tensor imaging and high b-value DWI study

**Authors:** D. Ben Bashat, V. E. Drory, M. Artzi, B. Nefussy, T. Zachor, Y. Assaf, O. Aizenstein

**Affiliations:** ^1^The Wohl Institute for Advanced Imaging, Tel Aviv Sourasky Medical Center, Israel; ^2^ALS Clinic, Department of Neurology, Tel-Aviv Sourasky Medical Center, Israel; ^3^Tel Aviv Universities, Israel

**Background**

Amyotrophic lateral sclerosis (ALS) is a fatal neurodegenerative disorder of the motor system causing damage to both the upper and lower motor neurons (UMN/LMN). To date, there are no imaging techniques available for objectively assessing the UMN damage.

**Purpose**

The purpose is to evaluate the cerebral white matter quantitatively using diffusion tensor imaging (DTI) and high bvalue diffusion weighted imaging (DWI).

**Method**

MR protocol included T1, T2, DTI (*b* = 1000 s/mm2) and high b-value DTI (b-values up to 12 000 s/mm2). Twenty-three UMN ALS patients and 20 age match healthy volunteers were scanned on a 3T GE MRI scanner. Diffusion tensor tracktography (DTT) was used and mean FA, Prob and Disp were calculated for the motor and sensory fibers separately.

**Results**

On the data from axial images, reduced FA was detected in the study group compared to the control group (*P* < .05) in both the motor and sensory fibers. Reduced FA, Prob, and increase Disp values were also detected in the study group compared to the control group in the high b-value coronal data set (*P* < .05 only for FA). Histogram analysis of FA, Prob, and Disp revealed no differences between the two groups. No correlation was found between FA, Disp, and Prob values and ALSFRS-R or with disease duration. Sub group of ALS patients with Buluar onset demonstrate significant correlation between FA and Eigenvalues to disease duration.

**Conclusion**

This study shows that diffusion tensor image is a sensitive method to detect damage to the corticospinal tract. These methods may improve the early diagnosis of the disease and promote the appropriate treatment for the patients. In addition, also show sensory fibers impairment in ALS patients that has been reported previously only in few cases.

# Abstract: Immunotherapies for Alzheimer's Disease

**Authors:** J. Ben Porata, D. Terkieltaub, O. Goren, N. Yakov, J. Abrameto, N. Sharon, I. Zaretsky, S. Shaki-Loewenstein, S. Hazum, J. Gopher, T. Ohayon, R. Eren

**Affiliations:** Intellect Neurosciences Ltd, Rehovot, Israel

The accumulation of amyloid-beta in the brain results in the formation of neurotoxic fibrillar intermediates, leading to a progressive, debilitating, ultimately fatal, neurodegenerative cascade that results in Alzheimer's disease (AD). Amyloidbeta is generated by proteolytic cleavage of the ubiquitous amyloid precursor protein (APP) which has important physiological functions. Current drug treatments for AD provide symptomatic relief of cognitive and memory impairments without addressing neurodegeneration and the underlying causes of the disease. Immunotherapy emerged recently as an approach to prevent amyloid-beta accumulation. Studies using AD transgenic mice as well as clinical trials provide encouraging data for immunotherapy as an effective approach to reduce amyloid-beta toxicity with the potential to slow or arrest disease progression, provided that key safety issues are addressed and adverse reactions are reduced. The key to develop safe and effective AD immunotherapies lies in designing vaccines and antibodies that are highly specific for amyloid-beta, do not cross react with endogenous functional proteins such as APP and do not activate cytotoxic T-cell responses. Our immunotherapy programs consist of “freeend specific” antibodies and chimeric-peptide vaccines that recognize specifically the free N-terminus or C-terminus of amyloid-beta peptide and, consequently, are capable of discriminating between toxic amyloid-beta peptides and functional endogenous APP peptides. In addition, our vaccines use very short “free-end” peptide fragments that are devoid of harmful T-cell epitopes. This “free-end specific” immunization concept is likely to minimize the risks for an autoimmune response and has the therapeutic potential to promote clearance of amyloid-beta in a safe and effective manner.

# Abstract: Exploring Alpha Correlates In The Brain–An EEG-fMRI Study

**Authors:** E. Ben Simon, I. Podlisky, A. Zhdanov, D. Papo, T. Hendler

**Affiliations:** ^1^Functional Brain Imaging Unit, Wohl Institute for Advanced Imaging, TASMC, Israel; ^2^Sackler Faculty of Medicine, Tel Aviv University, Israel

The frequency of electrical brain activity is commonly thought to be functionally meaningful. However, exactly what functional brain networks mediate activity at given frequency bands remains largely unknown. The alpha rhythm is considered the hallmark of the brain resting state and can be modulated by simply opening or closing eyes. This study aimed to match individually characterized EEG rhythms and simultaneously acquired whole brain functional magnetic resonance (fMRI) BOLD activation associated with spontaneous variations of the alpha rhythm (8–12 Hz), Subjects were instructed to close/open their eyes every 30 seconds. EEG activity was recorded from 32 electrodes simultaneously with whole brain fMRI in a 3T GE scanner. Activity in the alpha band was defined for each subject. Electrodes at which activity was maximal were then selected. The signal filtered at the individually-defined frequency ranges was then convolved with the corresponding fMRI signal and used as a regressor for statistical parametric mapping. Preliminary results show that eyes closed and open are associated with distinct widely distributed activation patterns. Preliminary results for alpha band-related BOLD signals showed differential peak activation distributions in occipital and frontal areas. Using EEG based fMRI analysis we then showed that the a priori defined oscillatory bands are associated with distinct widely distributed activation patterns. The significance of combined measurements will be discussed.

# Abstract: Acute stress in mice; alteration in behavior, immunity and cancer development

**Authors:** N. Benaroya-Milshtein, N. Hollander, A. Apter, I. Yaniv, Y. Haberman, H. Halpert, C. G. Pick

**Affiliations:** ^1^Department of Anatomy, Sackler Faculty of Medicine, Tel Aviv University, Israel; ^2^Department of Psychiatry, Schneider Children's Medical Center of Israel, Petah Tikva, Israel; ^3^Department of Human Microbiology, Sackler Faculty of Medicine, Tel Aviv University, Israel; ^4^Department of Hemato-Oncology, Schneider Children's Medical Center of Israel, Petah Tikva, Israel

**Background**

It is known that the neuroendocrine system and the autonomic nervous system are regulators of brain-immune interaction. However, the functional significance of this interaction under a variety of conditions is not fully understood.

**Objective**

The objective is to investigate the effect of stress paradigm on behavioral tests, immune parameters, and tumor progression and survival of lymphoma-bearing mice.

**Methods**

C3H mice were either exposed to an electric shock followed by 3 reminders (once a week) or were left untreated. Following stress paradigm mice behavior was tested in elevated plus maze, staircase, hot plate and open field, and NK cell activity was measured. In addition, mice were tested for proliferation of lymphocytes, spleen weight and number of leucocytes in spleen. Different mice were vaccinated twice with the immunoglobulin of the B-cell lymphoma 38C-13 and antiidiotype antibodies in sera of mice were determined by ELISA. Other mice were inoculated with tumor cells and monitored for tumor development and for survival.

**Results**

No major differences were found in behavior between the stressed mice and control mice. However, significant difference were found in immunological and cancer parameters. The mice exposed to stress paradigm had higher cytolytic activity than did the standard group (*P* < .05). When mice were immunized with an idiotype-vaccine, those exposed to stress paradigm produced higher levels of antiidiotype antibodies (*P* < .01). In addition, proliferation in the presence of mitogenes was higher in exposed group, so did number of leucocytes in spleen. Exposed mice revealed attenuated tumor growth (*P* < .05), with no difference in survival.

**Conclusions**

Stress paradigm revealed significant immune changes and attenuated tumor development, while no changes were found in behavior or survival after tumor cells inoculation.

# Abstract: Evaluation of the interplay between procedural learning and declarative learning systems revealed in a double-dissociation experiment among Alzheimer disease and Parkinson disease patients

**Authors:** E. Ben-Mair, T. Schonberg, R. Inzelberg, D. Joel

**Affiliations:** ^1^Department of Psychology, Tel Aviv University, Ramat-Aviv, Tel Aviv 69978, Israel; ^2^Rappaport Faculty of Medicine, Technion, Haifa, Israel

The division of memory systems into declarative and procedural is well established. The declarative system is involved in the acquisition and use of episodic and semantic memory, and is related to the hippocampal system. The procedural system is involved in the acquisition of habits and skills, and depends on the striatum. Although the two systems are thought to interact, the nature of interaction is not well understood. Animal studies have suggested that under some conditions the two systems may interfere with each other. Specifically, rats sustaining lesions to the hippocampus performed better than intact rats in a procedural task. The present study tested whether such interference may also occur in humans. Healthy subjects, patients with Parkinson's disease (PD), in which the dopaminergic input to the striatum degenerates, and patients with mild Alzheimer disease (AD), in which the hippocampal system is the first to degenerate, were tested in a card betting task. This task is unique in that about half of normal subjects do not acquire it, suggesting that it is particularly sensitive to interference arising from the declarative system. It was therefore hypothesized that whereas PD patients will not be able to acquire the task, AD patients will perform better than controls, due to a deficient declarative system. AD patients (*n* = 16), PD patients (*n* = 17) and controls (*n* = 21) performed the card betting task as well as immediate and delayed recognition tasks that assess declarative functioning. PD patients were indeed impaired on the card betting task and AD patients were impaired on the “declarative” tasks. However, in contrast to our hypothesis, AD patients did not perform better than controls on the card betting task. In summary, this study adds to previous demonstrations of a double dissociation between the declarative and procedural systems in humans. However, there is no evidence for interference between these two systems in the acquisition of the card betting task.

# Abstract: Zinc-induced Upregulation of T-type Calcium Currents in the Hippocampus

**Authors:** F. Benninger, D. Ekstein, A. Becker, Y. Yaari

**Affiliations:** ^1^Departments of Physiology, Hebrew University-Hadassah School of Medicine, Jerusalem, Israel; ^2^Departments of Neurology, Hebrew University-Hadassah School of Medicine, Jerusalem, Israel; ^3^Department of Neuropathology, University of Bonn Medical Center, Bonn, Germany

A single episode of status epilepticus (SE) caused by pilocarpine, triggers multiple changes in neuronal structure and function (referred to as epileptogenesis) that ultimately lead to chronic epilepsy. One striking change is the upregulation of CaV3.2 T-type calcium current, which causes many hippocampal pyramidal cells to display intrinsic bursting behavior. The processes which couple SE to T current upregulation are not known. We hypothesized before that SE-induced accumulation of zinc may play a role in these processes and we have found, that intracerebroventricular injection of zinc transforms about 60% of CA1 pyramidal cells into bursting neurons. Therefore, we examined here whether zinc also induces an increase in T current density in these neurons. We injected saline with or without ZnCl2 (45 *μ*M) into the right ventricles of adult Sabra rats. Zinc-induced neurodegeneration precluded recordings in the right (ipsilateral) hippocampi. Sharp electrodes current-clamp, whole-cell patchclamp recordings of T currents and immunostainings for CaV3.2 were performed in slices from the contralateral hippocampi. We found that the bursting activity in CA1 pyramidal cells was suppressed by specific blockage of the T-type calcium current in 1/3 of the tested cells. Two days following zinc injection the densitiy of T current was markedly (3-4 fold) increased in approximately 1/3 of the neurons compared to neurons in the control group. Congruently, immunostainings disclosed an increase in CaV3.2 abundance in CA1 pyramidal cells in hippocampi from zinc injected rats. Our findings show that an increase in brain zinc causes upregulation of CaV3.2 abundance leading to an increase in T current and associated neuronal bursting. They support our hypothesis that an increase in free zinc during or shortly after SEmay contribute to epileptogenesis. These findings have therapeutic implications for the prevention of epilepsy following brain insults.

*Supported by SFB* *TR3, BMBF,* *Humboldt Foundation and MOST.*

# Abstract: Inhibitory effect of kinins on microglial inflammation

**Authors:** S. Ben-Shmuel, S. Fleisher-Berkovich

**Affiliations:** Department of Clinical Pharmacology, Ben Gurion University of the Negev, P.O. 653, Beer-Sheva, Israel

Increasing evidence suggests that inflammatory mechanisms are a crucial component in neurodegenerative diseases, such as Alzheimer's disease (AD), and may significantly contribute to the disease progression. One of the pathological hallmarks of AD is chronic microglial activation and consequent over-production of proinflammatory mediators such as prostaglandins, cytokines and nitric oxide (NO). The latter, at high concentrations, may be neurotoxic and can be involved in the neurodegeneration process. Kinins are potent stimulators of the production and release of NO in many cells. Whether this is universal, remains to be investigated. Recent reports suggest a possible neuroprotective role of bradykinin (BK), probably mediated by microglial cells. The aim of the present study was to investigate the role of BK in the regulation of basal and lipopolysaccharide (LPS)- induced NO synthesis in a microglial cell line, BV2, and to assess the involvement of each of the two BK receptors, B1 and B2, in this effect. Long-term (24–48 hours) exposure of cells to BK (10 nM) reduced basal NO release by 75%. B1 receptor agonist, [Lys-des-Arg9]-BK, had the same effect as BK, 70% reduction of NO synthesis at a concentration of 10 nM. BK also attenuated LPS-induced NO production by 30% and 50% at concentrations of 1 nM and 10 nM, respectively. The B1 receptor agonist also attenuated LPS-induces NO production by 30% at 1 nM. These preliminary results imply a novel neuroprotective role of BK via attenuation of NO released from microglial cells under both basal and inflammatory conditions.

*This research was supported by* *the Israel Science Foundation (Grant no. 349/07).*

# Abstract: Cone-shaped lysophospholipids modulate voltage-gated calcium channel currents in pituitary cells

**Authors:** G. Ben-Zeev, I. Nussinovitch

**Affiliations:** Department of Anatomy and Cell Biology, Hebrew University Medical School, Jerusalem, Israel

Lysophospholipids (LPLs) are lipophilic molecules consisting of a hydrophilic head and a hydrophobic tail. LPLs containing a large hydrophilic head and a thin hydrophobic tail were defined as cones. It was hypothesized that incorporation of cones into the outer leaflet of the phospholipid bilayer increases membrane tension and it was demonstrated that cone-shaped LPLs alter the gating of mechanosensitive ion channels. Previous studies demonstrated modulation of voltage-gated calcium channels (VGCC) by membrane stretch. We therefore examined the effects of the cone-shaped molecule, Lysophosphatidylchloline (LPC), on VGCC in pituitary cells. Our findings may be summarized as follows: (1) LPC (10–30 *μ*M) differentially suppressed L-type and T-type calcium channel currents (IL and IT, resp.); the effects on IT were faster and more prominent than the effects on IL; (2) the effects of LPC on IL started after long delays (50–100 seconds), exhibited slow onset kinetics and were reversible only after washout with BSA; (3) the effects of LPC on IL were both dose-dependent and voltage-dependent with a rightward shift in the activation curve of about 9 mV; (4) the effects of LPC on IL persisted after block of G-proteins with GDPbetaS and after block of PKC with GF 109203X; (5) the effects of LPC on IL were mimicked by lysophosphatidylinositol (LPI), a negatively charged cone-shaped lipophilic molecule; (6) the effects of LPC on IL were not mimicked by a short chain LPC (C6:0) and by phosphatidylcholine, a cylindrical-shaped lipophilic molecule. In summary, our results show that cone-shaped lipophilic molecules (LPC and LPI) suppress VGCC in pituitary cells and that this suppression is independent on the polarity of the hydrophilic head. Our results also suggest that incorporation of LPC and LPI into the phospholipid bilayer underlies this suppression, and that molecular shape is a determinant in this suppression.

*Supported by the Israel Science Foundation Grant 826/04*.

# Abstract: Face Recognition Inspired by the Visual Cortex

**Authors:** E. Berkovich, H. Pratt, M. Gur

**Affiliations:** ^1^Department of Biomedical Engineering, Technion, Israel; ^2^Evoked Potentials Laboratory, Technion, Israel

Object recognition is one of the main functions of the human visual system. It is performed intuitively and spontaneously. Unlike computer vision systems, the human visual system is known for its ability to identify an object after spatial transformation and change of viewing and illumination conditions. Mimicking the visual system can therefore enhance computational object recognition. Hence, in this research, low-level features of the image are extracted by appropriate filters, in a manner similar to the human visual system. Previously, we have shown that our biologically motivatedmodel produces improved categorization results (e.g., distinguishing between cars, faces, etc.). In the current work we further analyze the contribution of our biological features to the recognition task. We demonstrate our results on the popular problem of face recognition. We analyze color face images (R,G,B channels) and show that recognition performance improves as more biological principles are incorporated into the model. Worst recognition results are obtained when the images are processed as grayscale (intensity channel); a significant improvement in performance is achieved by representing images in the biological color-space by adding two opponent-color channels (B/Y, R/G) to the intensity channel. Finally, we use the complete ensemble of biological features—intensity and opponent-color channels shaped by appropriate filters at various scales and orientations. On the ensemble of these features, we apply, iteratively, biologically inspired Boosting. At every iteration, the biological features are used to construct weak (face) learners on the training data. The best weak learner is weighted by its accuracy and then added to the final strong learner. At the successive iteration, the training data is reweighted: examples that are misclassified gain weight and examples that are classified correctly lose weight. This technique results in the best recognition performance.

# Abstract: Implicit and explicit morphologically related activation

**Authors:** A. S. Bick, R. Frost, G. Goelman

**Affiliations:** ^1^ICNC, Hebrew University, Jerusalem, Israel; ^2^Psychology, Hebrew University, Jerusalem, Israel; ^3^Medical Biophysics, Hadassah Hebrew University Hospital, Jerusalem, Israel

A dispute exists as to whether morphology is a discrete and independent element of lexical structure or whether it simply reflects a fine-tuning of the system to the statistical correlation that exists among orthographic and semantic properties of words. Imaging studies in English failed to show unequivocal morphological activation that is independent of semantic or orthographic activation (Devlin et al., 2004). In Hebrew, Cognitive research has revealed that morphological decomposition is an important component of reading (Frost et al., 2000), and using fMRI we identified areas in the brain, the lMFG and lIPS, activated distinctly during explicit morphological processing (Bick et al., in press). In this fMRI study, we aimed to replicate our results using an implicit task. This would further establish our results and show that morphological processing is automatic in reading Hebrew. Words were presented in the mask priming paradigm (Forster & Davis, 1984) and subjects perform a lexical decision task. Subjects were not aware of the presented prime. The manipulation was the relation between prime and target. We used primes that were morphologically related, semantically related and orthographically related to targets. In the morphology condition we used both words semantically related to the target and words sharing the same root but having quite distinct meanings. We found different patterns of activity in the different priming conditions. In the lMFG and lIPS activation was significantly reduced when the prime was morphologically related to the target. This effect was not found in the semantic or orthographic condition. Furthermore this attenuation was not influenced by the semantic transparency of the morphological prime. These results strongly resemble our previous results and reflect the importance of morphological processing in Hebrew reading. Scans were performed in Hadassah Hebrew University Hospital, Israel.

*Supported by Center for Complexity Science*.

# Abstract: On the implicit usage of repeated reference stimuli in the visual modality

**Authors:** T. Biron, M. Ahissar

**Affiliations:** ^1^Interdisciplinary Center for Neural Computation, Israel; ^2^Department of Psychology, Israel; ^3^Neurobiology Department, Hadassah Medical School, Israel

Most psychophysical tasks assess performance around a reference stimulus. Previously (Lubin and Ahissar, 2005), we studied which types of reference repetitions improve resolution in auditory 2-tone frequency discrimination. We had 3 conditions: no reference (NR)—no repetition across trials; reference 1st (R1st)—reference tone (1000 Hz) was presented in the first interval; reference 1st or 2nd (R1st-2nd)— reference tone was presented in the 1st or in the 2nd interval. Surprisingly, compared to the NR, listeners' frequency resolution was significantly improved only in R1st, indicating that consistent temporal position is crucial for reference utilization. We now asked whether consistency of temporal position is also crucial in the visual domain. We administered a spatial frequency discrimination paradigm analogous to the auditory paradigm, asking which of 2 sequentially presented grating stimuli is denser. We used the same conditions: NR, R1st (0.5 c/deg) and R1st–2nd. In contrast to the auditory results, the 2 reference conditions were performed similarly, and significantly better (7% ± 1.3) than the NR (22% ± 3.0) condition, indicating that temporal position is not crucial. We further assessed a simultaneous frequency discrimination paradigm, in which the gratings were presented simultaneously and subjects were asked whether the denser grating was on the upper or lower half of the screen. We administered 3 equivalent conditions: NR, RU—the reference was always at the upper half of the screen, RU or L—the reference was either at the upper or the lower half of the screen. In the parallel paradigm only the RU condition was better than NR, indicating that spatial consistency is crucial for reference utilization in the visual domain. These findings show that repeating a reference improves resolution only under specific conditions. Temporal consistency is crucial in the auditory modality, whereas spatial consistency is crucial in the visual modality.

*The study was supported by ISF Center of Excellence grant and by the Institue of Psychobiology*.

# Abstract: The effects of music background on sleep efficiency, anxiety and depression in Schizophrenia patients

**Authors:** B. Bloch, Y. Haliba, L. Vadas, Y. Ben Shitrit, A. Ehood Golz, K. Shelahavich, R. Tangi, H. R. Tavakuli, N. Ziv, I. Kremer, I. Haimov

**Affiliations:** ^1^Psychiatric Department, Emek Medical Center, Afula, Israel; ^2^Department of Behavioral Sciences, The Max Stern Academic College of Emek Yizreel, Israel

**Introduction**

Disturbed sleep is a common complaint among Schizophrenia patients. Since previous studies have demonstrated treatment resistance for sleep-medication treatment for insomnia in these patients, the aim of the present study was to examine the effects of music background as treatment for insomnia in Schizophrenia patients.

**Methods**

Twenty four Schizophrenia patients, who had no other major psychiatric, sleep, or neurological disorders, participated in the study (mean age = 45.67, SD = 9.6, 13 males and 11 women). The study comprised one 7-day, running-in period, followed by one 7-day experimental period. The treatment was music background at desired bedtime. During each of these two experimental periods, subjects' sleep was continuously monitored with a wrist actigraph (Ambulatory Monitoring, Inc.) and subjects were asked to fill out several questionnaires concerned with a wide spectrum of issues, such as depression, anxiety, and life satisfaction.

**Results**

A paired-sample t-test was conducted, comparing objective sleep parameters manifested by patients with music background at desired bedtime and without music exposure. A significant difference was found in sleep latency (*t*(23) = 3.01, *P* < .006), showing shorter sleep latency when music background was played (21.04 ± 14.6) than when it was not (37.01 ± 32.4). A significant difference was also found in sleep efficiency (*t*(23) = −3.35, *P* < .003), showing higher sleep efficiency when music background was played (86.0 ± 15.5) than when it was not (82.44 ± 18.3). A significant difference was found in depression level (*t*(23) = 2.96, *P* < .007), showing lower depression level after background music was played (12.29 ± 12.1) than when it was not (15.25 ± 10.6). No significant differences were found in anxiety level (*t*(23) = .22, *P* > .05), nor in life satisfaction (*t*(23) = 1.28, *P* > .05).

**Conclusion**

Overall, the findings imply the beneficial effect of music background as treatment for insomnia in Schizophrenia patients.

*The last two authors contributed equally to this research*.

# Abstract: Integrating telemetry data in toxicological studies: The effect of chronic exposure of rats to low doses of VX

**Authors:** E. Bloch-Shiderman, I. Rabinovitz, I. Egoz, J. Kapon, L. Raveh, E. Grauer, N. Allon

**Affiliations:** Department of Pharmacology, Israel Institute for Biological Research, Ness Ziona, Israel

Organophosphorus (OP) warfare nerve agents are highly toxic compounds that irreversibly inhibit cholinesterases (ChE). Inhibition of ChE results in the accumulation of acetylcholine in the synaptic cleft, leading to excessive stimulation of cholinergic receptors. Following exposure to high and lethal OP doses, signs of toxicity such as salivation, tremors, convulsions and seizures, respiratory failure and ultimately death are seen. Following exposure to lower sublethal OP doses none of these severe signs are observed. Therefore, it is impossible to elucidate any significant effect following exposure to sub-lethal doses using the standard toxicity data. Comprehensive series of studies have shown to have long-term neurological and psychological disorders (namely insomnia) following sub-lethal OP doses in humans. Since the data suggested a continuous deterioration in the circadian rhythm, it was very challenging, technologically and methodologically, to find a suitable marker for such insult in an animal model. In this study we present an approach of measuring alterations in the circadian rhythm body temperature and changes in ECoG activity by utilizing telemetry following chronic, sub-lethal doses of the nerve agent VX. VX was administrated via Alzet mini osmotic pumps implanted in the rats for one month. Data was collected continuously 24 h/day during the 3 days preexposure and the following 1 month of VX exposure. Fast Fourier transform spectrum analysis was performed for 30 s period, every 3 hours of the EcoG recordings. Changes in circadian rhythm, as well as changes in individual brain wave were detected. This approach might elucidate the potential damage caused by chronic, low-dose OP exposure.

# Abstract: Localization of cognitive function in rats with magnetic resonance imaging

**Authors:** T. Blumenfeld-Katzir, Y. Assaf

**Affiliations:** Department of Neurobiochemistry, Faculty of Life-Sciences, Tel Aviv University, Israel

The brain localization of cognitive functions is limited to invasive lesion or electrophysiological studies. Noninvasive imaging techniques such as functional magnetic resonance imaging (MRI) are less informative in rodents due to the use of anesthetics. In this work we used a novel MRI-behavior correlation framework for localization of cognitive performance in rats. Thirty one rats underwent two MRI sessions (at 7T) at ages of 9 and 12 months (MRI1 and MRI2, resp.). The MRI session included a diffusion tensor imaging (DTI) protocol from which the fractional anisotropy (FA) and apparent diffusion coefficient (ADC) maps were extracted. While the FA is more sensitive to white matter the ADC represent both gray matter and white matter. Between the two MRI sessions the rats performed a 6-day Morris water maze test. From the water maze we used the latency of the rats to reach the platform in the first (d1), fifth (d5), and sixth day (d6) in which the platform was removed (reversal trial). The comparison between the two imaging sessions revealed a significant FA and ADC signal changes in the hippocampus. Correlation analysis between the FA/ADC indices of MRI1 with latency of d1, d5, and d6 were localized at the amygdala, hippocampus and caudate/putamen regions, respectively. Correlation analysis between the FA/ADC indices of MRI2 with latency of d1, d5, and d6 was not localized to any specific region. These results manifest the different cortical domains that affect the behavior at the different days of the water maze; while in d1 stress factors may control the performance of the rat, spatial memory skills and decision making affect the d5 and d6 performance. In addition the fact that between the MRI sessions the hippocampus showed significant signal change may indicate on the plasticity changes that this region underwent following the Morris water maze. To conclude, MRI-behavioral correlations can be used for localization of cognitive functions in rodents.

# Abstract: Optimal multi-modal state estimation by neural networks based on recursive spike train decoding

**Authors:** O. Bobrowski, R. Meir, Y. C. Eldar

**Affiliations:** Department of Electrical Engineering, Technion, Israel

It is becoming increasingly evident that organisms acting in uncertain dynamical environments employ exact or approximate Bayesian statistical calculations in order to continuously estimate the environmental state and integrate information from multiple sensory modalities. What is less clear is how these putative computations are implemented by cortical neural networks. We show how optimal real-time state estimation based on multi-modal sensory information may be effectively implemented by neural networks decoding sensory spikes. We demonstrate the efficacy of the approach on static decision problems as well as on dynamic tracking problems, and relate the properties of optimal tuning curves to the properties of the environment.

# Abstract: Immunotherapy targeting pathologically phosphorylated tau conformers in a tangle mouse model

**Authors:** M. Boimel, N. Grigoriadis, A. Lourbopoulos, O. Abramsky, H. Rosenmann

**Affiliations:** ^1^The Department of Neurology, Hadassah University Hospital, Jerusalem, Israel; ^2^The Department of Neurology, AHEPA University Hospital, Thessaloniki, Greece

While immunotherapy targeting amyloid-*β* has been intensively investigated, only little is known regarding immunotherapy targeting tau aggregates (neurofibrillarytangles, NFTs), a major neuropathology in Alzheimer'sdisease (AD) and tauopathies. We reported recently the hazardous potential of immunizing mice with full length recombinant tau for targeting host normal tau protein, inducing neurological deficits and NFT-pathology. Here we investigated the effect of immunotherapy targeting specifically pathological tau conformers (phosphorylated, P) in a NFT-mouse model. The results are of special interest since last month it has been published that using a similar approach by immunizing with peptide tau-P-Ser396,404 reduced robustly NFTs in NFT-mice (Asuni 2007). We immunized NFT-mice with a mixture of P-tau peptides related to NFTs of AD/tauopathy patients and mice (tau- P-Ser202,Thre205, tau-P-Thre212,Ser214, tau-P-Thre221) with complete Freund's adjuvant and pertussis. The mice were examined for clinical neurological deficits, and effect on NFT-burden. No neurological deficits were noted following tau-P-immunotherapy at least for 8 months. Reduced Gallyas staining relative to controls (∼20% *P* = .02), indicating a reduced NFT-burden, was detected. Some similar trend, yet smaller, was noted in IHC using AT8 and AT180 Abs (recognizing NFT-related P-tau), but did not reach a statistic significance. To conclude, our results suggest a decrease in NFT-burden following tau-P-immunotherapy (tau- P-202,205,212,214,221), mostly demonstrated by Gallyas staining, but less by NFT-related IHC. The effect seems less robust than that reported with tau-P396,404. This may be related to the different tau-P peptides and immunization protocols used, or may point to the limited effectiveness of tau- P-immunotherapeutic approach. Moreover, the lack of clinical deficits points that these specific peptide immunogens are encephalitogenic, encouraging further study of their anti- NFT potential.

# Abstract: Asymmetric visual interactions across the boundary of awareness

**Authors:** Y. S. Bonneh, D. Sagi

**Affiliations:** The Weizmann Institute of Science, Israel

Motion-induced-blindness (MIB), Bonneh et al., Nature 2001, was used to examine the accessible properties of highcontrast visual objects which are not consciously available. Stimuli consisted of a static Gabor target embedded in a rotating grid. Under such conditions, the target perceptually disappears after few seconds. Following (100 milliseconds) Observers' report of disappearance, a high-contrast Gabor probe was presented, and the target was removed within 200– 600 milliseconds. The probe often caused the reappearance of the invisible target within the limited trial time. Surprisingly, we found low reappearance rates when probes were not in the proximity of the target or when orthogonal to it, even when proximal. High reappearance rates were observed with probes proximal and similar to the target. Plaid targets made of superimposed orthogonal Gabor patches reappeared when probes included only one component, but plaid probes were not very effective with component targets. The results show that subconscious objects preserve their location and component-orientation and demonstrate that visual processes sensitive to proximity and feature-similarity operate across the boundary of awareness. By controlling the duration of target presentation we found that “reactivating” the target may take 200–400 milliseconds, depending on the depth of suppression. The asymmetric interaction between visible and invisible plaids suggests that invisible objects break into their feature components.

# Abstract: Training and behavioral recovery after hippocampal lesion are coupled with changes in the pattern of connectivity

**Authors:** A. Boucard, J. Micheau, X. Nogués

**Affiliations:** Center for Cognitive and Integrative Neuroscience, University of Bordeaux, Paris, France

We have previously shown that the activities of the brain structures making up the memory systems defined by White and MacDonald (2002) are mainly correlated with the hippocampus in the place condition of a water escape task, whereas they are correlated with the amygdala in the cue condition. This demonstrates a rearrangement of the functional connectivity depending on the learning condition and suggests that the activation of a memory system should be described as the focusing of functional connectivity toward the central structure of the system. This may explain how several memory systems can share the same structures while remaining independent. Della-Maggiore and coll. (2000) showed that age-related alteration to the system sustaining delayed visual discrimination could be compensated by the reconfiguration of the pattern of connectivity. Indeed, whereas the performances of young and old subjects were comparable, the activated cerebral networks were different. Although, this change in connectivity may be due to the use of a different strategy, this study gives an insight into understanding how decreasing cognitive resources due to age (or pathology) may be compensated. To support this concept, we induced dorsohippocampal lesions tomice. Sham- and hippocampallesioned rodents were submitted to the place learning condition in the water cross-maze. Though impaired during the first three days, lesioned mice had the same level of performance than the sham-mice on the fifth day. We hypothesize that this behavioural recuperation would be coupled with a change in the pattern of connectivity. The activities of mice brain structures are evaluated by counting the number of Zif 268 immunoreactive cells after training in the place condition of the aquatic cross-maze. By using structural equation modelling, the present study investigates how the pattern of effective connectivity induced by learning is changed face to the lesion of the hippocampus.

# Abstract: DA inhibits mitochondrial membrane potential possibly through its interaction with complex I: implications to DA related pathologies

**Authors:** H. Brenner-Lavie, E. Klein, D. Ben-Shachar

**Affiliations:** Laboratory of Psychobiology, B. Rappaport Faculty of Medicine, Technion, Israel

Dopamine is suggested as a prominent etiological factor in several neuropsychiatric disorders such as Parkinson's disease and schizophrenia. In such dopamine related pathologies mitochondrial dysfunction has been reported. Notably, mitochondria are target organelles for dopamine by the virtue of the fact that MAO, the main metabolizing enzyme of dopamine, is located on their outer membrane. We have recently demonstrated that dopamine reversibly inhibited complex I activity in isolated disrupted mitochondria. In order to investigate whether DA-complex I interaction affects mitochondrial function in viable cells, we studied the effect of DA on mitochondrial membrane potential as a marker for the oxidative phosphorylation activity and mitochondrial respiration in intact viable human neuroblastoma SHSY5Y cells. DA significantly dissipated mitochondrial membrane potential and inhibited mitochondrial respiration in cells, similarly to complex I inhibitor, rotenone. Complex I-DA interaction induced mitochondrial dysfunction as bypassing complex I of the respiratory system prevented the DA induced depolarization of mitochondrial membrane potential as well as the reduction in the respiration rate. Unlike complex I, DA did not inhibit complexes II, IV and V activities in disrupted mitochondria. Abnormal interaction between dopamine and mitochondrial complex I was observed in schizophrenia, as in patients dopamine inhibited complex I activity twice as much as that of control subjects or of patients with affective disorders. Notably, antipsychotic drugs but not antidepressants inhibit mitochondrial respiration through complex I. The present study suggests that DA affects mitochondrial function through its interaction with complex I of the oxidative phosphorylation system. We further hypothesize that the interaction between DA and mitochondria is associated with mitochondrial dysfunction observed in DA-related neuropsychiatric disorders, such as in schizophrenia.

*Supported by the Chief Scientist Israel Ministry of Health*.

# Abstract: The possible association between streptococcus group A infection and the emergence of obsessive compulsive disorder

**Authors:** L. Brimberg, I. Benhar, M. Cunningham, D. Joel

**Affiliations:** ^1^Department of Psychology, Tel Aviv University, Israel; ^2^Department of Biotechnology and Microbiology, Tel Aviv University, Israel; ^3^Department of Microbiology and Immunology, University of Oklahoma, Okla, USA

A poststreptococcal autoimmune process has been suggested to be involved in the pathogenesis of a subgroup of children with tics and obsessive compulsive disorder (OCD), identified by the acronym PANDAS for Pediatric Autoimmune Neuropsychiatric Disorder Associated with Strepptococcal infection. It has been suggested that antibodies produced against group A beta-hemolytic streptococci (GABHS) cross react with neural cells, in a process involving molecular mimicry. Although PANDAS has received much attention in recent years, no study to date assessed compulsive-like behaviors in an animal model of GABHS-related neuropsychiatric symptoms. The aim of the present study was therefore to test the hypothesis that group A streptococcal infection may lead to the emergence of OCD. To this end, Lewis rats were immunized with an extract prepared from GABHS, and their compulsive behavior was assessed using two rat models of OCD, namely, the signal attenuation model and the induced-grooming assay. Sera taken from rats immunized with GABHS extract demonstrated stronger immuno reactivity to streptococcus A and to neural tissue compared to sera of control rats and in addition induced high levels of a calcium/calmodulin-dependent protein (CaM) kinase activity in human neuroblastoma cells. Antianibodies immunolabeling was stronger in the striatum and thalamus of immunized rats compared to control rats (control rats' brain showed low level, if any, of anti-IgG immunolabeling). In parallel, rats immunized with GABHS antigens tended to show increased compulsivity in two animal models of OCD. These preliminary results suggest that immunizing rats with GABHS extract can lead to the emergence of “compulsive” behavior.

# Abstract: Global leukocyte DNA methylation in schizophrenia patients

**Authors:** A. Bromberg, J. Levine, B. Nemetz, R. Belmaker, G. Agam

**Affiliations:** ^1^Psychiatry Research Unit, Faculty of Health Sciences, Ben-Gurion University of the Negev, Beer-Sheva, Israel; ^2^Beer-Sheva Mental Health Center, Israel

Meta-analysis recently suggested that a 5*μ*M increase in homocysteine is associated with a 70% higher risk for schizophrenia. Elevated homocysteine is reported to alter macromolecule methylation. We studied whether elevated plasma homocysteine levels in schizophrenia are associated with altered global DNA-methylation. DNA was extracted from peripheral blood leukocytes of 28 schizophrenia patients versus 26 matched healthy controls. Percent of global genome DNA-methylation was measured using the cytosine-extension method. Homocysteine levels were higher in schizophrenia patients than in controls (15.8 *μ*M ± 10.5 versus 10.6 ± 2.7, *P* = .016). No difference in global DNA methylation between schizophrenia patients and control subjects was found (74.0% ± 14.8 versus 69.4 ± 22.0, *P* = .31). A significant interaction between diagnosis and smoking on DNA methylation was obtained (*F* = 6.8, *df* = 1, 47, *P* = .032). Although leukocytes may be a useful cell model to evaluate epigenetic changes such as global DNA methylation in brain, future studies are on the way to compare global DNA methylation in peripheral tissue versus brain in laboratory animals.

# Abstract: Long-lasting olfactory-learning induced modification in the reversal potential of fast IPSPs in the piriofm cortex

**Authors:** I. Brosh, E. Barkai

**Affiliations:** Departments of Biology and Neurobiology, Faculty of Sciences, University of Haifa, Haifa, Israel

We have previously shown that olfactory-discrimination learning results with a series of intrinsic and synaptic modifications in layer II piriform (olfactory) cortex pyramidal neurons, which last for days after learning completion. In particular, enhanced excitatory synaptic transmission is indicated by reduced paired-pulse facilitation, enhanced rate of rise of EPSPs and increased spine density along basal and apical dendrites of these neurons. The purpose of the present study was to examine whether inhibitory synaptic transmission input into pyramidal neurons is also modified after olfactory learning. Intracellular recordings with sharp electrodes were made from layer II pyramidal neurons five days after training completion, in the presence of APV and DNQX, to block glutamatergic synaptic transmission. IPSPs were evoked by electrical stimulations applied in layer III. The averaged fast IPSP's reversal potential was significantly lower in neurons from trained rats (−76.66 mV + 1.3, *n* = 21 for trained versus −70.79 + 1.69, *n* = 18 for naive and −70.77 + 1.28, *n* = 19 for pseudo trained, *P* < .005). We suggest that olfactory learning is accompanied by hyper-polarization of the reversal potential of fast inhibitory synaptic transmission. This shift enhances the effect of such inhibitory transmission, thereby compensating for the increase in excitatory transmission.

# Abstract: Cellular Mechanisms of Deep Brain Stimulation

**Authors:** J. Bugaisen, I. Bar-Gad, A. Korngreen

**Affiliations:** ^1^The Mina and Everard Faculty of Life Sciences, Israel; ^2^The Leslie and Susan Gonda Brain Research Center, Bar-Ilan University, Israel

Deep brain stimulation (DBS) within the basal ganglia (BG) is a common treatment for Parkinson's disease (PD) in its advanced stages and is used in an exploratory manner in multiple movement and behavioral disorders. Multiple experimental studies show that stimulation in globus pallidus (GP) leads to complex modulation of the neuronal activity in the stimulated nucleus. In order to investigate the basic effect of DBS on single neurons we used DBS-like protocols using extracellular electrodes similar to those used in vivo and recorded the cellular response in acute brain slices from rats using the BG whole-cell configuration of the patch-clamp technique. We present characterization of several types of cells in the GP using whole-cell current clamp protocols and the effect of the stimulation on the spiking pattern of these cells. The analysis of these cellular mechanisms of response to stimulation are crucial for developing advanced therapeutic stimulation protocols and pave the way to close loop stimulation devices.

# Abstract: Reorganisation ofmotor deficits in patients with acute cerebellar stroke

**Authors:** U. Bultmann, M. Frings, D. Pierscianek, N. Fritsche, E. R. Gizewski, D. Timmann, M. Maschke

**Affiliations:** ^1^Department of Neurology, University of Duisburg-Essen, Germany; ^2^Department of Diagnostic and Interventional Radiology and Neuroradiology, U of Duisburg-Essen, Germany; ^3^Department of Neurology, Hospital of the Barmherzigen Brueder, Trier, Germany

**Introduction**

Previous studies have shown an advantage of treadmill training over conventional physiotherapeutic treatments in patients with gait disturbances after acute supratentorial stroke. Likewise, repetitive exercises like the “arm ability training” (Platz et al., 2001) lead to an improved motor performance. The present study investigated these therapies in patients with acute cerebellar stroke.

**Methods**

Twelve patients with acute (<four weeks) ischemic lesions of the territory of the superior or of the posterior inferior cerebellar artery and twelve healthy control subjects were examined. One half of the patients received a daily half-hour treadmill training over two weeks. Patients with a relevant arm ataxia received an additional “arm ability training.” The localisation of the lesions was determined by 3D MRI. Gait disturbances and arm ataxia were quantified by clinical scores and by a 3D camera system. All examinations were performed in the acute phase, after two weeks and three months.

**Results**

No significant effect of treadmill training or “Arm Ability Training” could be shown. Treated as well as untreated patients showed a nearly complete restitution of motor symptoms after three months. In seven patients the ischemic lesion involved cerebellar nuclei in the acute phase, but only in one patient after three months. The clinical scores were worse in those patients with involvement of cerebellar nuclei.

**Conclusions**

The results of the present study suggest a relationship between the extent of the initial motor deficits and the involvement of cerebellar nuclei. No effects of the rehabilitative therapies could be shown, possibly because of the good spontaneous recovery fromsymptoms in patients without permanent lesions of the cerebellar nuclei. The results have to be controlled in a larger group of patients.

*Supported by BMBF, German-Israeli Cooperation in Neuroscience (01GA0508), and IFORES (D/107-20540; D/107- 20550)*.

# Abstract: Tuning of the point of view of mirror neurons in monkey premotor cortex

**Authors:** V. Caggiano, L. Fogassi, M. Giese, G. Rizzolatti, P. Thier, A. Casile

**Affiliations:** ^1^Department of Cognitive Neurology, Hertie Institute for clinical brain research, University of Tuebingen, Germany; ^2^Department of Psychology, University of Parma, Italy; ^3^LARL, Hertie Institute for clinical brain research, University of Tuebingen, Germany; ^4^Department of Physiology, University of Parma, Italy; ^5^School of Psychology, University ofWales Bangor, Gwynedd, UK

Mirror neurons are a class of neurons in monkey pre-motor cortex that respond both when the monkey performs an action and when he sees the same action performed by humans or monkeys. While many studies have shown that such neurons are robustly activated under naturalistic conditions, it has been difficult to elicit single-cell responses using filmed stimuli. Here we present neurophysiological evidence in one monkey showing that mirror neurons do respond to movies. In our experiments, we first isolated mirror neurons and further tested them with filmed actions. To keep the monkey's attention to the movie a fixation paradigm was used. The stimuli consisted of filmed goal-directed actions as observed from three different points of view: frontal view, side view and first person view (i.e., the forelimb seen by the acting monkey). About 40% (*n* = 34) of the recorded mirror neurons responded also to filmed actions. Half of them (*n* = 17) preferentially responded to actions as seen from the first person view. The remaining neurons responded either to multiple points of view (*n* = 10) or showed a preference for actions as seen from the side view (*n* = 1) or frontal view (*n* = 6). Our quantitative investigations show that the responses of mirror neurons are modulated by the point of view from which actions are observed. Two nonmutually exclusive explanations can be given for this result. First, under naturalistic conditions, monkeys as well as humans are more often visually exposed to their own actions (i.e., actions as seen from the first person view). Second, the mirror neuron system might have originally evolved to map monkey's actions onto own motor representations possibly for refining object manipulation behaviors. The same system was then, later in evolution, adapted to also understanding the manipulation behaviors of conspecifics.

*Supported by the Deutsche Forschungsgemeinschaft (SFB 550- C10)*.

# Abstract: Osmoticmodulation of neuronal excitability is mediated by KV7/M channels

**Authors:** A. Caspi, F. Benninger, Y. Yaari

**Affiliations:** Department of Physiology, School of Medicine, The Hebrew University, Jerusalem, Israel

Modest reduction in extracellular osmotic pressure (pi) causes neuronal network hyperexcitability and epileptiform seizures. It is commonly believed that this effect of low pi is mediated by constriction of the extracellular space consequent to cell swelling, which enhances excitatory electric field effects. However, we have shown previously that low pi also increases intrinsic neuronal excitability in principal brain neurons. Specifically, we found that low pi promotes bursting by facilitating the spike afterdepolarization (ADP) in hippocampal pyramidal cells. Such an effect undoubtedly would contribute to low pi-induced network hyperexcitability. How does low pi facilitate the spike ADP and bursting? The waveformof the spike ADP is determined by interplay of two low-threshold, noninactivating currents: persistent Na^+^ current (INaP) and M-type K^+^ current (IM). Thus, facilitation of the ADP by low pi may be due to INaP enhancement and/or IM inhibition. We tested these two hypotheses using intracellular sharp microelectrode recordings in CA1 pyramidal cells in situ. Our data favor the second hypothesis for the following reasons: (1) after blocking IM with 20 *μ*M XE991, which by itself caused ADP facilitation and bursting, low pi had no further effect; (2) after blocking INaP with 10 *μ*M riluzole, which by itself caused ADP suppression, low pi still enhanced the ADP; (3) injection of positive current ramps into neurons perfused with ACSF containing Na^+^ and Ca^2+^ channel blockers, disclosed an increase in membrane conductance at voltages positive to −60mV (IM activation threshold), which was prevented by XE991 and enhanced by the IM enhancer retigabine (10 *μ*M). Low pi mimicked the effect of XE991 in reducing membrane conductance, and its effect was occluded by XE991. These and other data suggest that low pi facilitates the spike ADP and bursting activity by inhibiting IM. This hypothesis is currently being examined using voltage clamp techniques.

*Supported by BSF and SFB-TR3*.

# Abstract: The compensation of oculomotor fatigue is based on the adjustment of a Purkinje cell simple spike population signal

**Authors:** N. Catz, P. W. Dicke, P. Thier

**Affiliations:** Department of Cognitive Neurology, Hertie Institute for Clinical Brain Research, Tbingen, Germany

Short-term saccadic adaptation (STSA) is a form of cerebellar-dependent motor learning in which a retinal vector, defining a desired spatial location, becomes associated with a saccade vector, whose amplitude may differ from that of the retinal vector by up to 30%. We have previously reported that STSA is based on adjusting a Purkinje cell (PC) simple spike (SS) population signal and that disturbances of saccadic adaptation, resulting from cerebellar disease may be accompanied by an inability to maintain stable saccade amplitudes if long series of stereotypic saccades have to be carried out. We have interpreted the latter as uncompensated saccadic fatigue, an inability of the saccade system to account for changes of the plant due to usage, an idea which is supported by the comparison of the saccade kinematics of patients and controls. Here we tested the idea that the cerebellar mechanism used to compensate saccadic fatigue is the same as the one causing STSA. To this end, we recorded SS from nonsaccade related vermal PC in lobuli VI and VII while monkeys executed several hundred stereotypic saccades. Indeed, when the animals repeated the same saccades time and again, their kinematics changed while their accuracy was maintained: a decrease in peak velocity was fully compensated by an increase in saccade duration. Parallel to the increase in saccade duration, many of the PC developed new, late saccade-related bursts, fired around the time of the end of the altered saccade. These newly arising saccade-related bursts were fully appropriate to extend the duration of a SS population response as based on the responses of PC, already saccade-related at the outset, by an extent needed in order to account for the increase in saccade duration. These findings are in full accordance with the concept that the cerebellum controls behavior using a population code and, moreover, support the notion that STSA is the manifestation of amechanism needed in order to compensate fatigue.

*Supported by the Deutschen Forschungsgemeinschaft SFB 550*.

# Abstract: Neurobiology of stress: the role of CRF/Urocortin family of peptides and receptors

**Authors:** A. Chen

**Affiliations:** Department of Neurobiology, Weizmann Institute of Science, Rehovot, Israel

The biological response to stress is concerned with the maintenance of homeostasis in the presence of real or perceived challenges. This process requires numerous adaptive responses involving changes in the central nervous and neuroendocrine systems. When a situation is perceived as stressful, the brain activates many neuronal circuits linking centers involved in sensory, motor, autonomic, cognitive, and emotional functions in order to adapt to the demand. However, the details of the pathways by which the brain translates stressful stimuli into the final, integrated biological response are presently incompletely understood. Nevertheless, it is clear that dysregulation of these physiological responses to stress can have severe psychological and physiological consequences, and there is much evidence to suggest that inappropriate regulation, disproportional intensity, or chronic and/or irreversible activation of the stress response is linked to the etiology and pathophysiology of psychological and physiological disorders. Understanding the neurobiology of stress by focusing on the brain circuits and genes, which are associated with, or altered by, the stress response will provide important insights into the brain mechanisms by which stress affects psychological and physiological disorders. We examine the hypothesis that the CRF/Urocortin family of peptides and receptors play an important role in modulating the neuroendocrine and behavioral responses to challenge. Defining the contributions of the individual ligands and receptors of the CRF/Urocortin family to the maintenance of stress-linked homeostasis may improve our ability to design therapeutic interventions for, and thus manage, affective and stress-related disorders. We employ integrated molecular, biochemical, physiological and behavioral methods, focusing on the generation of mice genetic models as an in vivo tool, in order to study the central pathways and molecular mechanisms mediating the stress response.

# Abstract: Epigenetic factors in the vulnerability to PTSD: the role of DNA methylation

**Authors:** Y. Chertkow-Deutsher, D. Ben-Shachar, H. Cohen, E. Klein

**Affiliations:** ^1^Laboratory of Psychobiology, Department of Psychiatry, Faculty of Medicine, Technion, Haifa, Israel; ^2^Anxiety and Stress Research Unit, Faculty of Health Sciences, Ben-Gurion University, Beer-Sheva, Israel; ^3^Department of Psychiatry, Rambam Medical Center, Haifa, Israel

Posttraumatic stress disorder (PTSD) develops in about 20% of those exposed to a threat to life or physical integrity. This implies the participation of dynamic biological processes such as epigenetic modifications. This study investigated whether variations in DNA methylation pattern is associated with maladaptation to traumatic event in a rat model of PTSD. Rats were exposed to a predator and classified into “PTSD-like” and “non-PTSD” according to their response in elevated plus-maze and acoustic startle response paradigm. Hippocampal global DNA methylation pattern was assessed using amplification of inter-methylated sites method (AIMS): successive digestion of genomic DNA by SmaI and Cfr9I restriction enzymes, produced a mix of originally methylated fragments, which were amplified and radiolabeled by PCR and separated on 6% polyacrylamide denatured sequencing gel. From the complex pattern of bands, we isolated 7 fragments displaying differential methylation pattern between the “PTSD-like” and “non-PTSD” groups. These fragments were amplified by PCR reaction, cloned and sequenced. Their location on the rat genome was established using the BLAST engine. Several sequences showed a complete homology with intron or intron-exon boundaries of known genes. The genes identified by this procedure Dll3, PKCeta, Fstl3 and Rps6kb2 participate in synaptic formation and remodeling, differentiation of cells, protein phophorylation and other cellular regulatory processes. We are now in a process of validation the methylation changes using bi-sulfite assay and examining whether these variations have functional significance by quantifying mRNA expression. Our initial results suggest that changes in DNA methylation pattern may be associated with maladaptation to environmental stress and the development of PTSD.

*Supported by The National Institute for Psychobiology, Israel*.

# Abstract: On the use of reference in auditory duration discrimination

**Authors:** M. Cochavi, M. Ahissar

**Affiliations:** ^1^Interdisciplinary Center for Neural Computation (ICNC), Hebrew University, Israel; ^2^Psychology Department, Hebrew University, Israel

The ability to estimate temporal intervals on the scale of tens to hundreds ofmilliseconds is important for adequate motor, visual and auditory abilities. Yet, little is known about how the brain estimates these durations. In a typical paradigm, listeners are asked which of 2 sequentially presented brief tones is longer. We now asked whether applying a consistent reference improves the accuracy of temporal estimations. We applied 3 paradigms: reference short—a 100-millisecond reference tone was presented either in the 1st or in the 2nd interval in each trial, and nonreference tone was always longer (the typical paradigm applied in the literature); reference 1st- the reference tone was always presented in the 1st interval, and the 2nd interval contained a shorter or longer tone; no reference—there was no repeated reference across trials. Sixty sixHUstudents participated in the experiment (31 with musical background), each tested with a single paradigm, administered in 2 sessions (1 week apart). Each session contained 5 subsequent 80-trial assessments, using an adaptive staircase procedure. The reference 1st paradigm was performed significantly better than the other 2 paradigms from the very first assessment. The small difference in protocol between reference 1st and reference short elicited a 2.6 fold difference in thresholds, and a 4.8 fold difference in cross subject variability (11% ± .06 versus 29% ± .29). This benefit remained constant within and across assessment sessions. The no reference condition yielded even somewhat (though not significantly) poorer discriminations (36% ± .21). Musically trained students performed significantly better. These results indicate that a reference can dramatically sharpen discrimination. However, detecting a repeated duration is not immediate for the auditory system. Temporal certainty is of crucial importance, whereas duration certainty, even when consistently mapped to response category (always shorter) is not sufficient.

*The study was supported* *by the ISF Center of Excellence Grant & by the Institute of Psychobiology.*

# Abstract: Themolecularmechanism of neural induction of human embryonic stemcells

**Authors:** M. A. Cohen, P. Itsykson, B. E. Reubinoff

**Affiliations:** ^1^The Hadassah Human Embryonic Stem Cell Research Center, The Goldyne Savad Institute of Gene Therapy, Israel; ^2^The Department of Gynecology; Hadassah Medical Center, Jerusalem, Israel

The induction of neural tissue represents an early and fundamental step in the generation of the vertebrate nervous system. During this process, pluripotent cells are directed to a neural fate. Much of the understanding of neural induction in vertebrates is derived from studies performed in Xenopus embryos. These studies led to the “default model” of neural induction, which proposes that cells within the ectoderm layer of the frog gastrula have an autonomous tendency to differentiate into neural tissue, which is inhibited by BMPs which act as an epidermal inducers. Later works in Xenopus and chick models have shown a more complex picture in which FGFs are major players in addition to BMP inhibition and suggested BMP signaling-dependent and or independent roles of FGF. In attempting to understand the principles that underlie neural induction in humans, we use the human embryonic stem cell (hESC) model. We established a simple one-step approach for derivation of neural precursors (NPs) fromhESCs in chemically defined serum-free suspension culture conditions .This system serve as a defined in vitro model to study early neural differentiation of hESC. Here we report that bFGF promotes the expression of early neural genes such as Musashi1 and NeuroD1, suggesting its role in early neural specification. This effect is not associated with neural cells proliferation. Moreover, this FGF effect is probably, at least in part, not mediated through antagonism of BMP signaling. Nevertheless, hESCs, when cultured in the absence of FGF and BMP-signaling, differentiate towards a neural fate, suggesting a default mechanism of neural induction, similar to that which was reported in amphibian models. Our data suggest that bFGF plays a role, but is not essential, in the neural induction process of hESCs, and supports the “default model” of neural induction in mammals.

# Abstract: Protonation of extracellular histidine residues facilitates C-type gating of the human potassium leak channel K2P2.1

**Authors:** A. Cohen, S. Chen, Y. Ben Abou, N. Zilberberg

**Affiliations:** The Department of Life Sciences and the Zlotowski Center for Neuroscience, Ben-Gurion, Israel

Potassium leak currents play a critical role in determining cell in determining cell resting membrane potential and thus control nerve and muscle excitability. K2P2.1 (KCNK2, TREK-1), a member of the 2P-domain K^+^ channel (K2P) family, is expressed in the central and peripheral nervous system, as well as in the heart, kidney and testis tissues. K2P2.1 activity is regulated by a variety of physical and chemical effectors including temperature, fatty acids, internal pH, mechanical stretch and phosphorylation. Although K2P2.1 is classified as a leak channel, it was shown to posses an exceptional capability to reversibly convert between leak and voltage dependent phenotypes. Here, we report that human K2P2.1 channel, expressed in Xenopus oocytes, is inhibited by external acidosis within the physiological range (pKa = 7.3). Mutations in two outer pore histidine residues dramatically decreased channel responsiveness to external acidosis. Mutation of a conserved glutamate residue (E84) known to be fundamental to C-type gating had a similar effect. Furthermore, those three residues were found to be located in close proximity to one another. We thus propose a novel mechanism for pH sensing in which protonation of histidine residues destabilize hydrogen bonds, formed by E84, that are necessary formaintaining the C-type gate at the open conformation. In accordance with this proposed mechanism, proton induced current decrease was inhibited by external potassium ions and enhanced by a mutation (S164Y) known to accelerate C-type gating. Moreover, proton current inhibition is voltage-dependent being more pronounced at negative potentials. The voltage-dependent current fraction was significantly decreased in the pHo insensitive mutants, presenting I-V relationship of an open rectifier channel. Thus, we suggest that voltage dependent C-type gating acceleration by protons, in physiological pHo, serves as a mechanism for the voltage dependent mode and the outward rectification behavior of K2P2.1.

# Abstract: Exacerbated subthreshold oscillatory activity in ratmodel of essential tremor revealed by in-vivo intracellular recordings of inferior olivary neurons

**Authors:** E. Cohen, I. Lampl

**Affiliations:** Weizmann Institute for Science, Israel

The inferior olive (IO), the sole provider of climbing fibers to the purkinje cells (PCs), is suspected to be centrally involved in the etiology of the most common movement disorder, essential tremor (ET). ET is characterized by a 4–12 Hz tremor of the limbs, head or jaw which is amplified during intentional movements. Systemic application of harmaline, an alkaloid MAO inhibitor, causes tremor in animals comparable to ET, while its bath application to in-vitro preparations increases the occurrence of subthreshold oscillations (STOs) and their amplitude in IO neurons. These combined findings, contributed to the view that oscillatory activity of IO neurons is related to ET. Hence, harmaline treated animals have been used formany years as models for ET. The effect of harmaline on subthreshold activity of IO neurons invivo, however, has never been examined. We have recorded intracellularly IO neurons in anesthetized young adult rats. Several minutes following the injection of harmaline (10– 20 mg/kg, IP), in tune with muscle tremor appearance, IO neurons exhibited a remarkable transition in the oscillatory activity. Compared to control conditions, STOs exhibited at least 2-fold increase in both amplitude and oscillatory epoch duration. This transition was also accompanied by a significant hyperpolarization and an increased firing rate due to the larger amplitude of STOs. Harmaline, however, did not have a drastic effect on the average frequency of STOs relative to control conditions, nevertheless, it significantly narrowed their spectral distribution. Further investigation is required in order to relate the neuronal activity to muscle tremor and examine possible strategies to negate the effects of harmaline and restore normal STO activity.

*Supported by Nella and Leon Benozyio Center for Neurological Diseases*.

# Abstract: A novel role for ERK in maintaining long-term memory relevant excitability changes

**Authors:** S. I. Cohen-Matsliah, I. Brosh, K. Rosenblum, E. Barkai

**Affiliations:** University of Haifa, Israel

Pyramidal neurons in the piriform cortex from olfactorydiscrimination- trained rats show enhanced intrinsic neuronal excitability that lasts for several days after learning. Such enhanced intrinsic excitability is mediated by long-term reduction of the postburst after-hyperpolarization (AHP) which is generated by repetitive spike firing. AHP reduction is due to decreased conductance of a calcium-dependent potassium current, the sIAHP. We have previously shown that such learning-induced AHP reduction is maintained by PKC activation. However, the sequence of protein activation which maintains this exceptionally long-lasting modulation of intrinsic excitability is yet to be fully described. Here we examine whether ERKI/II, which has been implicated in learning andmemory as well as in synaptic plasticity process, is instrumental in this process. TheMEK inhibitor, PD98059, that selectively blocks ERK, increased the AHP in neurons from trained, but in neurons naїve and pseudo trained rats. Consequently, the difference in AHP amplitude and neuronal adaptation between neurons from trained rats and controls were abolished. This effect was not mediated by modulation of basicmembrane properties. Accordingly, the level of phosphorylated ERK in the membranal fraction was significantly higher in pirifrom cortex samples taken of trained rats. The PKC activator, OAG, that was shown to reduce the AHP in neurons from control rats, had no effect on these neurons in the presence of PD98059. Our data show that ERK has a key role in maintaining persistent PKC-mediated learninginduced enhancement of neuronal excitability.

# Abstract: Fast switching fromcomputation based to memory based perception—evidence from ERP

**Authors:** L. Daikhin, M. Nahum, M. Ahissar

**Affiliations:** ^1^Interdisciplinary Center for Neural Computation, Israel; ^2^Hadassah Medical School, The Institute of Medical Sciences, Israel; ^3^Department of Psychology, Hebrew University, Jerusalem, Israel

In two-tone frequency discrimination tasks, participants listen to sequentially presented tones and are asked to decide which tone is higher. Recent results from our lab (Lubin and Ahissar, ISFN 2005) showed that if one of the tones is repeated across trials (reference) at a consistent temporal position (either 1st or 2nd in each trial), performance quickly and significantly improves compared to a no-reference paradigm. These findings indicate that the reference is used effectively, perhaps by eliminating the need for actual comparison between the stimuli presented in each trial. In order to test this hypothesis, ERPs were measured while subjects performed the task. Two conditions were applied: reference 1st, in which the reference tone was always in the first position, and the second tone was either higher or lower, and reference 2nd, in which the reference was always second, and the first tone was either higher or lower. Thus, in reference 1st condition, trial-specific information, which is needed to resolve the task, is presented only at the second interval, whereas in reference 2nd condition, this information is provided already by the first interval. In the reference 1st condition both tones induced N1-P2-N2 waves, but only the second tone induced a P3 wave, indicating that a clear perceptual categorization only occurred after the second tone. In the reference 2nd condition, responses were similar, but the P3 decision wave was elicited after the first tone, and no P3 wave followed the second tone. The switch in the temporal position of P3 indicates that, in the reference 2nd condition, subjects indeed reach a decision after hearing only the first tone, before the second stimulus was presented. Taken together, we find that the presence of a reference stimulus eliminates the need for online comparisons within trials, and automatically enables the use of a better strategy. Noticeably, participants are unaware of this switch.

*The study was supported by ISF Center of Excellence Grant and by the Institue of Psychobiology*.

# Abstract: Themolecularmechanism of pharmacodynamic interactions between antipsychotic drugs and SSRI antidepressants

**Authors:** L. Danovich, O. Weinreb, M. B. H. Youdim, H. Silver

**Affiliations:** ^1^Molecular Neuropsychiatry, Brain Behavior Laboratory, Shaar Menashe MHC, Israel; ^2^Eve Topf Centers, Department of Pharmacology, Technion–Faculty of Medicine, Haifa, Israel

Clinical studies have shown that coadministration of selective serotonin reuptake inhibitor (SSRI) antidepressants (e.g., fluvoxamine) and typical antipsychotics (e.g., haloperidol) can ameliorate negative symptoms in schizophrenic patients. Efficacy against negative symptoms is also seen with certain atypical antipsychotics (e.g., clozapine). The effect of the combined treatment can not be explained by pharmacokinetic interaction, implying that complex molecular mechanism might lie in the basis of the distinctive clinical effect of this treatment. Our previous studies indicated that gamma-aminobutyric acid type A (GABA-A) beta3 receptor subunit, the enzyme glutamic acid decarboxylase (GAD67), and protein kinase C (PKC) beta2, known as importantmodulators of GABA activity, might be distinctively regulated by the combination of SSRIs and antipsychotics, compared to each individual treatment alone. In the present study, we have found that chronic coadministration (14 days, i.p.) of fluvoxamine (10 mg/kg) and haloperidol (1 mg/kg), as well as atypical antipsychotic clozapine (10 mg/kg), significantly decreased GABA-A beta3 receptor subunit protein levels and induced receptor endocytosis in rat frontal cortex. Individual treatment of fluvoxamine or haloperidol did not produce changes in GABA-A beta3 receptor subunit expression or endocytosis. In addition, we have shown that the combined treatment and clozapine similarly regulated molecular signaling pathways that modulate GABA-A receptor function, such as, PKC and ERK1/2. Furthermore, acute treatment (30minute and 1 hour) with fluvoxamine and haloperidol given together, or clozapine, altered ERK1/2 and PKC phosphorylation levels, and time-dependent dynamics of ERK phosphorylation were observed. Together, our findings suggest that the combined treatment may downregulate GABA system in rat frontal cortex, and alter the PKC and MAPK molecular signaling pathways.

# Abstract: Dopamine induces tyrosine phosphorylation of the NR2A subunit (PY1325) in mature rat hippocampus

**Authors:** O. David, H. Kaphzan, C. Anderson, S. Grant, K. Rosenblum

**Affiliations:** ^1^Department of Neurobiology & Ethology, center for brain and behavior, Haifa university, Haifa, Israel; ^2^Team 32–Genes to Cognition, Wellcome Trust Sanger Institute, Hinxton CB10 1SA UK

Dopamine and NMDA receptor are playing a major role in mature brain function and learning processes. Previous research in our laboratory identified convergence of NMDA and dopamine on the levels of ERK2 activation and rsk2 expression in the hippocampus. Furthermore, it was shown that the dopamine activation of ERK2 is NMDA receptor dependent (Kaphzan et al., 2006, 2007). The molecular basis for this functional interaction between dopamine and the NMDA receptors is not clear. However, its relevance for different brain functions is highly important. We hypothesize that dopamine interacts with the NMDA receptor via modulation of its tyrosine phosphorylation in the mature hippocampus. Using pharmacological set-up for mature hippocampal slices and phospho-specific antibodies, we found that application of both high and low doses of dopamine for ten minutes (100 *μ*M, 20 *μ*M) induced the phosphorylation of NR2A(Y1325) (*P* < .05, *n* = 4, *P* < .01, *n* = 8) but did not modulate the levels of NR2A protein itself. Moreover, application of the D2 and D1 dopamine antagonists, Etic (60 *μ*M) and SCH23390 (40 *μ*M) were sufficient to reduce basal phosphorylation of NR2A(Y1325,*P* < .05, *n* = 4), suggesting that intrinsic dopaminergic activity maintains tyrosine phosphorylation of the NR2A in the mature hippocampus. In contrast to dopamine, NMDA application, which induces ERK2 phosphorylation, did not induce NR2A(Y1325) phosphorylation (*n* = 8). Overall, our results suggest that dopamine interacts with the NMDA receptor via tyrosine phosphorylation of NR2A(Y1325). We currently are analyzing the differential effect of the different dopamine receptors on other tyrosine residues and NR subunits.

# Abstract: Dynamic Properties ofMartinotti-Pyramidal Synapses in Layer 2/3 of the Rat Somatosensory Cortex

**Authors:** K. Davidson, A. Korngreen

**Affiliations:** ^1^The Mina and Everard Goodman Faculty of Life Sciences, Bar Ilan University; ^2^The Leslie and Susan Gonda Multidisciplinary Brain Research Center, Bar-Ilan University

It has previously been shown that the height and width of APs recorded at the soma of bitufted interneurons from layer 2/3 of the somatosensory cortex are sensitive to changes in Israel Society for Neuroscience 25 resting membrane potential, suggesting subthreshold activity of voltage-gated conductances. It has also been found that the release of neurotransmitters from axon terminals of certain vertebrate neurons is influenced by the somatic membrane potential and that the voltage fluctuations associated with dendrosomatic synaptic activity propagate significant distances along the axon. These minute fluctuations in the somatic membrane potential of the presynaptic neuron appear to modulate the amplitude and duration of axonal action potentials. We set out to identify and examine synaptic interactions between bitufted interneurons and pyramidal cells in layer 2/3 of the somatosensory cortex of Wistar rats. Bitufted interneurons were identified through their spindle-like somatic morphology and the observed characteristic pattern of AP discharge following depolarizing current injection into the soma. Current injections into interneurons produced AP trains which height and width were observed to be sensitive to dynamic changes in membrane potential. IPSP amplitude in the post synaptic pyramidal cells depended on presynaptic membrane potential. These results confirm our initial hypothesis that not only it is possible for presynaptic somatic membrane potential to affect the magnitude of synaptic potentials in neurons other than cortical layer 5 pyramidal cells but that inhibitory post synaptic potentials may also be affected by this phenomenon.

# Abstract: Effect of exposure to a behaviourally relevant tone on auditory cortex responses to the same tone in mice

**Authors:** L. De Hoz, H. Raizel, I. Nelken

**Affiliations:** Department of Neurobiology, The Hebrew University of Jerusalem, Israel

We studied the effects of behavioural experience on sensory responses in mouse auditory cortex. Young C57BL/6J female mice were kept in a modified Intellicage (Newbehavior, Zurich, SW), a cage in which the animal receives food /ad libitum/ but access to water is contingent upon a correct nosepoke within a specialized drinking apparatus at a single corner of the cage. The behavior of the mice is continuously monitored using “transponders” (reporting chips) implanted subcutaneously. As a result, training can be achieved in the home cage, totally avoiding human handling. To attribute behavioral significance to a tone, half the visits to the drinking corner were accompanied by the presentation of a warning tone at a fixed frequency that was immediately followed by an aversive airpuff. Electrophysiological recordings in auditory cortex were carried out in anaesthetized animals taken out of the cage at various time points before and during the behavioral training. Stimuli consisted of tone sequences in which the warning tone was embedded with varying probabilities. Preliminary results indicate that during behavioural training animals learned to avoid the airpuff. Furthermore, the local field potentials in response to the warning frequency, when it was presented at a low probability as part of an oddball sequence, were more salient than the responses to other frequencies under the same conditions.

*This research is supported by an FP6 grant*.

# Abstract: Neonatal nitric oxide synthase inhibition leads to impaired reversal performance which is alleviated by clozapine: a neurodevelopmental model of negative symptoms

**Authors:** A. De Levie, I. Weiner

**Affiliations:** Department of Psychology, Tel-Aviv University, Tel Aviv 69978, Israel

Based on evidence on abnormalities in nitric oxide (NO) function in schizophrenia, we have developed a neurodevelopmental model of negative symptoms based on neonatal inhibition of NO synthase (NOS) during postnatal days 3–5. In this model, neonatal NOS inhibition led in adulthood to a cognitive deficit that is central to negative symptomatology, namely, attentional perseveration/inflexibility, as manifested in abnormally persistent latent inhibition. In support of its relevance to negative symptoms, this abnormality was resistant to the typical antipsychotic drug haloperidol but alleviated by the atypical APD clozapine. Here we aimed to demonstrate neonatal NOS inhibition-induced attentional perseveration and its alleviation by clozapine using an additional selective attention task, discrimination reversal. Rats were required to learn left-right discrimination in a T-maze with the choice of one of the arms reinforced, and then the two reinforcement contingencies were reversed so that the choice of the opposite arm was reinforced. Typically, the transfer of behavioral control to the current contingencies is impeded by the previously acquired contingencies. We found that neonatal NOS inhibition did not hinder rats' capacity to learn discrimination task or to retrieve the previously acquired stimulus-response contingency, but rather impaired their ability to shift behavioral strategy, that is, to choose the previously unrewarded arm. Thus, attentional perseveration seen in abnormally persistent LI was paralleled by increased perseveration in reversal. Furthermore, as seen in LI, this perseverative behavior was alleviated by clozapine. These results support the notion that neonatal NOS inhibition provides a model of negative symptoms with construct and predictive validity.

# Abstract: A psychophysical study of the thermal grill illusion

**Authors:** R. Defrin, L. Arendt-Nielsen

**Affiliations:** ^1^Department of Physical Therapy, Sackler Faculty of medicine, Tel-Aviv University, Tel-Aviv, Israel; ^2^Center forMotor-Sensory Integration, Aalborg University, Aalborg, Denmark

Interlaced hot and cold stimuli administered simultaneously on the skin induce a paradoxical heat sensation; a phenomenon termed the thermal grill illusion (TGI). Studies on the grill illusion have yielded contradictory results regarding its quality and intensity, which in turn led to controversies concerning the underlying mechanism. The controversies may result from testing the TGI with absolute stimulation temperatures thereby disregarding inter-subjects' variation in temperature sensitivity. Therefore, the first aim was to measure the individual threshold of the TGI. Another aim was to measure the spatial boundaries of the TGI by spatially separating between the cooling and warming stimuli, in order to explore possible mechanisms. Subjects (10 males, 15 females) underwent measurements of heat-pain (HPT) and cold-pain thresholds (CPT) with 9 and 18 cm,^2^ stimulating probes, on the forearm. Subjects also underwent measurement of pain threshold (PT), with one cooling and one heating probes (9 cm^2^ each) activated simultaneously to produce the TGI. The stimuli were separated by 0–30 cm, distances which encompass one or two dermatomes. Simultaneous cold and warm stimuli produced burning pain at all separation distances. PT values (∼26 and 38°C) were significantly lower than CPT and HPT, respectively, and were relatively fixed across all distances except for 30 cm at which PT increased towards CPT and HPT values. In conclusion, innocuous cold and warm stimuli can spatially summate, both within and between dermatomes and evoke a painful TGI. Most likely, nonnociceptive cold and warm channels integrate onto 2nd or 3rd order nociceptive neurons which, in turn induce a unique painful burning resulting from this blend.

# Abstract: Habituation to pain: the effect of absolute vs relative stimulation intensity

**Authors:** R. Defrin, A. Dror, L. Yulzari, C. G. Pick

**Affiliations:** ^1^Department of Physical Therapy, Sackler Faculty of Medicine, Tel-Aviv University, Tel-Aviv, Israel; ^2^Department of Anatomy, Sackler Faculty of Medicine, Tel-Aviv University, Tel-Aviv, Israel

Stimulation at a constant intensity and location usually induce a gradual decrease in receptors' output leading to weakening of subjective sensation, a phenomenon termed habituation. Habituation to pain was not studied in depth and results are controversial. The aim was to investigate the stimulation parameters allowing for pain habituation, that is, to assess the role of absolute versus relative stimulation intensity. Subjects were administered with cold and hot stimuli of either various absolute temperatures or at intensities set individually to induce an initial visual analog scale (VAS) scores of 2, 4, and 6 (weak, moderate, and strong pain, resp.). Subjects rated their perceived pain during stimulation (3–5 minutes). Absolute temperature, initial VAS scores and time significantly affected pain ratings. Temperatures of 43–45°C always induced reduction in pain ratings with time whereas 47-48°C always induced elevation in pain ratings with time, regardless of initial VAS scores. Stimuli equivalent to VAS 2 always induced reduction in pain ratings and those equivalent to VAS 4 and 6 induced elevation in pain ratings with time, regardless of absolute temperatures. Nevertheless, absolute temperature and initial VAS scores cointeracted in that they strengthened the patterns induced by one another. Sex affected the value of pain ratings but not their change with time. Habituation to pain occurs only for relatively weak temperatures or at low VAS levels. With strong stimulation temperatures and/or temperatures inducing moderatestrong pain, pain sensation gradually increases to unbearable intensities. It is hypothesized that the lack of pain habituation at those circumstances serves as a protective mechanism against potential and actual tissue damage. It also appears from the results that individual variations in pain threshold and responsiveness are less influential than stimulation parameters on pain habituation and excitation.

# Abstract: Selective hippocampal atrophy in Multiple Sclerosis quantitave MRI studies

**Authors:** Y. Deri, S. Miron, A. Achiron, C. Hoffmann, E. Konan, A. Biegon

**Affiliations:** ^1^The Joseph Sagol Neuroscience Center, Sheba Medical Center, Israel; ^2^Sackler Faculty of Medicine, Tel Aviv University, Israel; ^3^Multiple Sclerosis Center, Sheba Medical Center, Israel; ^4^Diagnostic Radiology, Sheba Medical Center, Israel; ^5^Brookhaven National laboratories, USA

Multiple sclerosis (MS) is an autoimmune disease characterized by inflammation and demyelination. A significant proportion of MS patients also exhibit cognitive deficits. The hippocampus is a brain region implicated in memory formation, and hippocampal volume decline has been demonstrated in conditions associated with cognitive deficits such as Alzheimer, depression and aging. The aim of the present study was to test the hypothesis that hippocampal volume declines as a function of disease duration in MS; using quantitative MRI. Twenty five women (age range 23–58) who underwent a brain MRI scan were included in the study. 9 subjects (age 23–50) with disease duration of less than 1 year had early (probable) MS (PMS) at the time of the scans. 10 additional subjects (age 29–58), with disease duration 3–24 years, had an established remitting-relapsing disease (RRMS) and 6 healthy women (age 30–54) served as controls. Hippocampal volumes were measured off T2 weighted images (axial plane, slice thickness 2.6–3 mm, no gap) using quantitative image analysis software (Image J), and normalized to skull and whole brain volumes. ANOVA, t-tests and simple regression were used for statistical analysis as appropriate, *α* = 0.05. A significant decrease in Hippocampus/skull and hippocampus/brain ratios was found when the RRMS, but not the PMS, groups were compared to controls (hippocampus/ skull: *F* = 8.12, *P* = .018 RRMS versus control, *P* = .000 RRMS versus PMS, hippocampus/brain: *F* = 5.08, *P* = .006 RRMS versus control, and *P* = .04 RRMS versus PMS). Furthermore, there was a significant correlation between the hippocampus/brain ratio and disease duration (*R* = 0.52,*P* < .03) among the MS patients. To the best of our knowledge this is the first report of a disease-related atrophy in hippocampus of RRMS. Considering the key role played by the hippocampal formation in memory processes, it is likely that preferential hippocampal atrophy is a significant contributor to the mechanism underlying cognitive deficits in MS.

*Supported by J. Sagol doctoral fellowship to YaelDeri*.

# Abstract: Classification of executed movements from fMRI response patterns in primary motor cortex

**Authors:** I. Dinstein, J. Gardner, M. Jazayeri, D. J. Heeger

**Affiliations:** ^1^Center for Neural Science, New York University, NY, USA; ^2^Department of Psychology, New York University, NY, USA; ^3^Physiology and Biophysics Department, University of Washington, Wash, USA

**Purpose**

Several recent studies have shown that fMRI response patterns from early visual areas enable accurate decoding of oriented gratings and motion directions (Kamitani and Tong 2005, 2006). Accurate classification is evidence of underlying neural selectivity for the changing dimension of the stimulus in the area tested. We used the same classification methodology to decode executed movement responses in several cortical areas including primary motor and somatosensory cortex.

**Methods**

Twelve subjects played the common game rock-paperscissors against a video taped virtual opponent whose movements were randomized. Subjects chose their movements freely with the objective of winning the game. We defined several cortical regions of interest (ROIs) including primary motor and somatosensory cortex and extracted the spatial pattern of fMRI responses in each ROI associated with each trial in the game. We then used Fisher linear discriminant analysis to classify responses fromthe three movement types. We tested the accuracy of the classification by decoding the executed movements on independent trials not included in those used to train the classifier (leave one out validation).

**Results**

Executed movements were decoded from the fMRI response patterns in primary motor and somatosensory areas with accuracy of ∼65%, significantly better than chance performance (33% because there were three possible movements).

**Conclusions**

Neural activity in human primary motor and somatosensory cortex is selective for executed movements. Classification can be successfully used to decode executed movements, even when using a fast event related fMRI protocol, with sufficient reliability to distinguish small differences in handmovement. This methodology may, therefore, enable assessment of underlying neural selectivity in the study ofmotor control (e.g., dissociating kinematics from dynamics).

*Supported by the HFSP and Seaver Foundation*.

# Abstract: A preformed signaling complex mediates GnRH activated ERK phosphorylation of paxillin and FAK at focal adhesions in LfiT2 gonadotrope cells

**Authors:** M. Dobkin-Bekman, R. Seger, Z. Naor

**Affiliations:** ^1^Department of Biochemistry, Tel Aviv University, Ramat Aviv 69978, Israel; ^2^Department of Biological Regulation, The Weizmann Institute of Science, Rehovot 76100, Israel

Most receptor tyrosine kinases (RTKs) and G-protein coupled receptors (GPCRs) activate MAPK cascades but still exert diverse functions and therefore signal specificity remains an enigma. Also, most GPCR ligands utilize families of receptors for mediation of diverse biological actions; however the type I GnRH receptor (GnRHR) seems to be the sole receptor mediating GnRH-induced gonadotropin release. Signaling complexes associated with GPCRs may provide the means for signal specificity. Here we describe a signaling complex associated with GnRHR, a unique GPCR lacking a C-carboxy tail. Unlike in cases of other GPCRs this signaling complex is preformed and exposure of LbetaT2 gonadotropes to GnRH induces dynamic rearrangement of the complex. The signaling complex core is c-Src and its binding partners include PKC delta, epsilon and alpha, Ras, MEK1/2, ERK1/2, tubulin, FAK, paxillin, vinculin, caveolin-1, KSR- 1 and the GnRHR. A short exposure to GnRH (5 minutes) causes MEK1/2, ERK1/2, tubulin, vinculin, and the Gn- RHR to detach from c-Src followed by reassociation with the complex by 30minutes. On the other hand, FAK, paxillin, the PKCs and caveolin-1 stay bound to c-Src and KSR appears in the complex only after 30 min of GnRH stimulus. GnRH stimulated ERK1/2 activity in the complex in a c-Src-dependent manner. The GnRH activated ERK1/2 then phosphorylates FAK and paxillin, while caveolin-1 is phosphorylated on Tyr14 apparently by the activated c-Src. RTKs and GPCRs translocate ERK1/2 to the nucleus to phosphorylate and activate transcription factors. Therefore the role of the multiprotein signaling complex seems to sequester a cytosolic pool of activated ERK1/2 to phosphorylate FAK and paxillin at focal adhesions apparently for cell migration and spreading.

*Supported by ISF and GIF*.

# Abstract: Less is more: the perceptual advantage of being color blinded

**Authors:** R. Doron, A. Meshulam, Y. Mandel, M. Rosner, M. Belkin, U. Polat

**Affiliations:** Sackler Faculty of Medicine, Goldschleger Eye Research Institute Tel Hashomer, Tel Aviv University, Israel

Color vision enables discrimination and segmentation between very similar objects, thus adding an important dimension to visual processing. One out of eight men has a lacks cones function in the retinal fovea, and as color blind, relies only on monochromatic information. In this study we explored the possibility that the visual system has adapted to the loss of information provided by the color system by achieving superior performance in the monochromatic system to compensate for the missing information. We measured basic functions of the visual system such as visual acuity, stereopsis, day contrast sensitivity (CS), and foveal mesopic CS. The mesopic CS was measured under conditions of full darkness, and with natural density filters that covered the monitor, transmitting background luminance of 0.03 cd/m^2^. The subjects were healthy young men (17– 35), with corrected-to-normal vision acuity (6:6 or better), with color blinded individuals (*n* = 9) or controls (*n* = 7). The results show a significant advantage of the colorblinded persons in the perceptual functions: better visual acuity, higher CS (average 35% during the day, and 24% at night), and better stereopsis (53%). Thus, the results indicate that the processing of monochromatic perceptual functions in color-blinded persons is superior to that of people with normal color vision. This suggests that the advantages of monochromatic vision lie in the consequence of an adaptation process throughout development in order to compensate for the loss of the important dimension of color vision.

# Abstract: Novel taste elevates c-fos expression in GABAergic neurons in the dysgranular Insular Cortex

**Authors:** G. Doron, K. Rosenblum

**Affiliations:** ^1^Department of Psychology, Israel; ^2^Department of Neurobiology and Ethology, Faculty of Science, University of Haifa, Israel

Conditioned taste aversion (CTA) may be acquired when an animal consumes a novel taste (conditioned stimulus, CS) and then experiences the symptoms of poisoning (unconditioned stimulus, US). Animals will later avoid the taste that was previously associated with malaise. Several molecular changes were found to be correlated with novel taste learning and CTA in the insular cortex (IC). However, very little is known on the circuit level within the insular cortex subserving taste learning (i.e., the specific cortical layers and cell type which mediate the molecular changes). Previous studies, that used the immediate early gene (IEG) c-fos protein as a functional marker of neuronal activity in the IC, have shown that c-fos expression is elevated in the IC following taste aversion learning. In the current study we used confocal microscopy along with c-fos, the GABAergic GAD67 and nuclear immmunofluorescence markers, in order to determine whether the change in c-fos expression is localized to GABAergic interneurons in the IC following novel taste training and CTA. Our results indicate that although total cfos expression is not changed following novel taste only, it is specifically higher in GABAergic neurons in layers I-IV of the dysgranular IC (DI). Moreover, in accordance with previous results, total c-fos expression is elevated in the IC following CTA, but also specifically in GABAergic neurons in layers IIV and layers IV-VI. In contrast, no layer specific elevation of c-fos expression in GABAergic neurons can be detected following LiCl injection only, although the total c-fos expression is higher following LiCl i.p. administration. We propose that novel taste learning is mediated through GABAergic layer specific activation in the DI, while CTA learning is involved in more sparse activation of neurons in the insular cortex.

# Abstract: Interbarrel synaptic connections in the rat barrel cortex

**Authors:** D. J. Dothan, I. Lampl

**Affiliations:** Department of Neurobiology, Weizmann Institute of Science, Rehovot 76100, Israel

A basic attribute of the neocortex is its organization into functional, vertically oriented columns, which is particularly prominent in the “barrel” field of the somatosensory cortex of the rat. Though the barrels in layer 4 have been extensively investigated, there is disagreement regarding direct interactions between layer 4 neurons of neighboring barrels. Whether or not these interactions exist is fundamental to our understanding of sensory integration of thalamic inputs and the origin of multiwhiskers response of layer 4 cells. One theory claims that direct interbarrel synaptic connections influence neighboring barrels. An opposing theory states that interactions between barrels are negligible and subcortical mechanisms are responsible for multiwhisker responses. To test for monosynaptic interbarrel connections, we made invitro intracellular current-clamp recordings in layer 4 of thalamocortical slices. Dissected slices connected only through layer 4 showed that electrical stimulation in one barrel triggers both antidromic and orthodromic responses in neurons of neighboring barrels, although these responses did not necessarily always appear together. Although EPSPs evoked by stimulation of a neighboring barrel were smaller than those evoked by direct barrel stimulation, this experiment provides an indication of a direct flow of information between barrels and suggests it can play a significant role in cortical gain control and determination of the cortical receptive fields.

*This work was supported by ISF grants*.

# Abstract: Advanced molecular genetic tools to further investigate the essential protein ADNP and its family member ADNP2

**Authors:** E. Dresner, M. Kushnir, I. Gozes

**Affiliations:** Human Molecular Genetics and Biochemistry, Sackler Medical School, Tel Aviv University 69978, Israel

Activity-dependent neuroprotective protein (ADNP) was first discovered as a protein associated with neuroprotection. The gene is highly conserved, and contains zinc fingers and a homeobox domain. ADNP is essential for brain formation and ADNP knockout mice exhibit failure of neural tube closure and death on E8.5-9.5. Upregulation of transcripts associated with lipid metabolism, and down regulation of transcripts associated with neurogenesis and organogenesis was observed in the knockout embryos. Recently, ADNP was found to interact with members of the mating type switching/ sucrose nonfermenting (SWI/SNF) chromatin remodeling complex. ADNP2 (KIAA0863) is a new protein, paralog to ADNP, with a very similar structure and expression pattern. Our research aim is to further understand the function of these proteins and their counter-relationship. Our findings indicatemutual regulation between ADNP and ADNP2. A reduction in ADNP2 expression was seen in the ADNP knock-out mice, and a reduction in ADNP was seen in knock down of ADNP2. As a first step, several research tools were constructed. First, ADNP and ADNP2 knock down systems using siRNA technology were established in P19 cells. These tools enable the examination and comparison of the phenotypic effect of ADNP and ADNP2 down regulation in both pluripotent and neouro-differentiated P19 cells. In addition, in order to trace the cellular localization of ADNP2, green fluorescent protein was attached to ADNP2 to form a chimeric protein, which was expressed in HEK293 cells. Finally, antibodies against two peptide sequences in ADNP2 were produced and affinity purified to identify the ADNP2 protein. Taken together, these tools will enable further investigations into the counter-relationship of the essential protein ADNP and its family member ADNP2.

*Support by BSF, ISF, Lily, and Avraham Gildor Chair, Adams Super Center, and Allon Therapeutics Inc*.

# Abstract: Neuronal rescue in thiamine deficiency

**Authors:** V. Dror, M. Rehavi, Y. Assaf, S. Eliash

**Affiliations:** Physiology and Pharmacology, Sackler Faculty of Medical, Tel Aviv University, Israel

Thiamine deficiency (TD) is a model of chronic impairment of oxidative metabolism leading to neuronal loss. Rats are fed a thiamine-deficient diet accompanied by injections of the central thiamine antagonist, pyrithiamine. They exhibit neurological impairments, cognitive deficits as well well-defined neuropathological lesions. Neurodegeneration develops over a period of 12 to 14 days and can be reversed by thiamine administration. Use of this model can help elucidate mechanisms of neuronal degeneration as well as important paths of neuronal rescue. Furthermore, since TD is partially reversible after thiamine administration, the effect of neuroprotective drugs on recovery can also be studied. In this study we investigated the neuroprotective effects of rasagiline, a MAO-B inhibitor, in TD rats, using neurological score, cognitive testing and histopathology. Magnetic resonance imaging (MRI) was used to study the pathology and brain changes in the rasagiline-treated and untreated-TD rats. Magnetic resonance spectroscopy (MRS) was used measure metabolic changes. We found that rasagiline significantly delayed the induction of the TD syndrome and decreased the severity of the associated neuropathology. Rasagiline improved cognition in the Morris water maze. The severity of both the cognitive and histopathological changes was significantly less in rasagiline-TD rats. T2-weightedMR imaging revealed severe time-dependent damages, mainly in the thalami and the inferior colliculi which were diminished with rasagiline treatment. We found that rasagiline both ameliorated and delayed neuronal injury. MRS showed an increase in lactate levels in the thalamus of untreated-TD rats which was postponed and decreased in the thalami of the rasagiline-TD group. Our results demonstrated significant neuroprotection by rasagiline in this model which could have implications for clinical neurodegenerative disorders.

# Abstract: Constraining neuron models by experimental data

**Authors:** S. Druckmann, A. Gidon, F. Schuermann, H. Markram, I. Segev

**Affiliations:** ^1^Interdisciplinary Center for Neural Computation, the Hebrew University of Jerusalem, Israel; ^2^Department of Neurobiology, Institute of Life Sciences, the Hebrew University of Jerusalem, Israel; ^3^Brain Mind Institute, Ecole Polytechnique Fèdèrale de Lausanne, Switzerland

Themain challenge in constructing conductance-based compartmental conductance-based compartmental models that capture the firing pattern of neurons is constraining the density of the various membrane ion channels that determine these firing patterns. Presently, quantitative data on the density of a certain ion channel along a specific dendritic region is by and large lacking. The dominant method to date is to record the in vitro experimental response of the cell to a set of current stimuli and then attempt to replicate the response in a detailed compartmental model of that cell. Yet, in experiments, intrinsic noise gives rise to a large variability in the voltage responses to repetitions of the exact same input. Thus, the common approach of fitting models by attempting to perfectly replicate, point by point, a single chosen trace out of the spectrum of variable responses does not seem to do justice to the data. In addition, finding a single error function that faithfully characterizes the distance between two spiking traces is not a trivial pursuit. To address these issues, we propose to adopt a multiple objective optimization (MOO) approach that allows the use of several error functions jointly. When more than one error function is available, the comparison between experimental voltage traces and model response can be performed on the basis of individual features of interest (e.g., spike rate, spike width). Each feature can be compared between model and experimental mean, in units of its experimental variability, thereby incorporating into the fitting a measure of this variability. We demonstrate the success of MOO when used in conjunction with genetic algorithm optimization, in generating an excellent fit between model behavior and the firing pattern of two distinct electrical classes of cortical interneurons, accommodating and fast-spiking. We argue that the multiple, diverse models generated by this method could serve as the building blocks for realistic simulation of large neural networks.

# Abstract: Endocrine regulation of serum butyrylcholinesterase activity: a study using dystrophin deficient mdxmice

**Authors:** A. R. Durrant, L. Tamaye, L. Anglister

**Affiliations:** Department of Anatomy and Cell Biology, Hebrew University Medical School, Jerusalem 91120, Israel

Postpubertal dystrophin-deficient mutant (mdx) mouse has been reported to exhibit both muscular dystrophy and raised serum acetylcholinesterase (AChE) concentrations [1]. In this work we report a butyrylcholinesterase (BuChE) deficiency (>30%) in mdx-sera when compared to wild-type (wt). Since AChE activity appears to be down-regulated at puberty in wt-sera but not in mdx [1], and since serum BuChEs in male rats is downregulated by the gonadal steroid testosterone [2], then possibly both ChEs are subjected to hormonal regulation, which may be impaired in mdx mutants. We therefore tested whether orchidectomy can attain the agonadal baseline level of wt ChEs in the mdx mutants. Sera of adult (20–22weeks old) male mdx and wt mice were collected and assayed for BuChE and AChE activities, by hydrolysis of butyryl- and acetyl-thiocholine substrates, using selective inhibitors. Six days after orchidectomy, BuChE activities were elevated in both strains; yet, the BuChE deficiency in mdx remained. Testosterone-replacement reversed the effect of orchidectomy. Gonadal modulation was specific for serum BuChE, not AChE. Our findings are consistent with the involvement of the hypothalamic-hypophyseal axis in the BuChE impairment of dystrophin-deficient mdx sera.

*Supported by MDA (LA)*.

## References

[1] Oliver LJ, Chatel J-M, Massoulie J, Vigny M, Vallette FM (1992). Molecular forms of acetylcholinesterase in dystrophic (mdx) mouse tissues. *Neuromuscular Disorders*.

[2] Illsley NP, Lamartiniere CA (1981). Endocrine regulation of rat serum cholinesterase activity. *Endocrinology*.

# Abstract: Leaders of Activity in Dissociated Neural Cultures

**Authors:** J. Eckmann, S. Jacobi, E. Moses, C. Zbinden, S. Marom

**Affiliations:** ^1^Department of Theoretical Physics, University of Geneva, Switzerland; ^2^Section of Mathematics, University of Geneva, Switzerland; ^3^Department of Physics of Complex Systems, The Weizmann Institute of Science, Israel; ^4^Department of Physiology and Biophysics, Faculty of Medicine, Technion, Israel

Recently, it was shown that neurons bursting in culture have a tendency to fire according to some order (Van Pelt 2005). Specifically, a subpopulation of the culture fires earlier than average (Eytan and Marom 2006). The role of these early-tofire neurons in the population bursting is not clear, as they may be only probes of the ongoing population activity on one hand, or lead the recruitment of activity in the burst on the other hand. In the light of this question, we have analyzed the spontaneous bursting activity data of dissociated rat hippocampal and cortical neuron cultures that were recorded over a few weeks using multi-electrodes. The cultures were prepared and grown in three labs using different protocols. Most of the bursts were preceded by a preburst activity, indicating a slow recruitment of activity in the neurons sampled by the electrodes, and therefore, in the whole culture. Still, surprisingly, we find that the locality of the electrode firing occurs in the preburst but is diminished in the burst. Leader electrodes were defined as those few electrodes that consistently fired first in prebursts. We observed that these leaders are stable over a few tens of hours, and that their distribution can only be temporarily disturbed using external stimulation. We show that the identity of the leader initiating the preburst of a given burst determines, statistically, the electrode firing statistics during the following burst. Based on these results, we suggest that the leaders belong to a subnetwork of neurons in the cultures and serve as nucleation centers for the recruitment of neural activity.

*JPE, CZ were supported by the Fonds National Suisse, SM by the ISF, EM and SJ by the ISF and Minerva*.

# Abstract: Association between DLG4 (PSD-95) scaffolding protein and autism: relations to adaptive behavior and cognition

**Authors:** S. Edelman, E. Lerer, A. Paz, T. Zeev-Ben-Mordehai, S. Israel, L. Nemanov, J. L. Sussman, I. Silman, S. Levi, S. Salomon, N. Yirmiya, R. P. Ebstein

**Affiliations:** ^1^Hebrew University, Jerusalem, Israel; ^2^Weizmann Institute of Science, Rehovot, Israel; ^3^S. Herzog Memorial Hospital, Jerusalem, Israel; ^4^Hadassah-Hebrew University Medical School, Jerusalem, Israel

PDZ-domain scaffolding proteins such as PSD-95 (DLG4) along with neurexins, neuroligins, and SHANK3 comprise the elements of trans-synaptic cell-adhesion complexes that are thought to bridge pre- and postsynaptic division. Neuroligins, *β*-neurexins, and SHANK3 have all been linked by both linkage and association studies to autism spectrum disorder (ASD), however, PSD95 has not yet been studied in this context. We identified and genotyped all six tagging SNPs (htSNPs) across the DLG4 gene region using HapMap and the Haploview program. PBAT-Helix tree was used to test association by family-based methods, in 152 probands from 133 families for htSNPs with DSM IV autistic disorder as well as IQ and vineland adaptive behavior scales (VABSs) scores. The results were also verified using UNPHASED. Nominal association was observed between1htSNP (rs17203281) and diagnosis (*P* value FBAT = .029), and 3 htSNPs were associated with IQ and VABS scores (IQ: rs929229 *P* = .043, rs3826408 *P* = .008; VABS: rs929229 *P* = .005, rs3826408 *P* = .022, rs507506 *P* = .024). Multivariate and haplotype analysis showed significant association between DLG4 and all three phenotypes (diagnosis, IQ and VABS). The FBAT-GEE multivariate statistic was significant for all four SNPs and for all three phenotypes (*P* = .0098). A common (25%) fourlocus haplotype (rs17203281-rs3826408-rs929229-rs507506: G-C-A-A) was significantly associated with all three phenotypes (FBAT-GEE, *P* = .03). Importantly, the current investigation, by demonstrating association of common tagging SNPS with ASD, VABS and IQ across the DLG4 gene region, suggests that dysfunction of synaptic complex scaffolding proteins such as PSD-95 may be a general risk factor in this disorder. The finding that IQ and VABS scores are also associated with DLG4 suggests that other disorders with Israel Society for Neuroscience 31 cognitive and social function deficits exhibited in daily living skills might be profitably investigated for association with PSD-95 and related scaffolding proteins.

# Abstract: Deficits in spatial learning in mice afterminimal traumatic brain injury demonstrated by a novel dry maze test

**Authors:** S. Edut, V. Rubovitch, S. Schreiber, J. Feldon, C. G. Pick

**Affiliations:** ^1^Department of Anatomy and Anthropology, Sackler Faculty of Medicine, Tel-Aviv University, Israel; ^2^Department of Psychiatry, Tel Aviv Sourasky Medical Center and Tel-Aviv Uni'Sackler Faculty of Medicine, Israel; ^3^Laboratory of Behavioural Neurobiology, Swiss Federal Institute of Technology (ETH Zurich), Switzerland

Spatial learning and memory deficits in rodents have largely been studied using the Morris water maze (MWM). Although originally the water maze task was designed for rats, it has also been applied to mice. However, learning in the MWM relies on swimming abilities, which evolutionary are not well developed in mice. It is thus not surprising thatmice when compared with rats show an impairment. We have therefore assessed spatial learning and memory in mice using a dry maze. The dry maze is a paradigm for measuring spatial learning and memory in mice. In the present study, evaluation of cognitive function was performed in ICR mice following minimal traumatic brain-injury (mTBI) or shaminjury. Mice were tested 7 days or 30 days post injury. The dry maze is a circular plastic arena which comprises 20 small wells arranged in a circular manner. The maze paradigm involves three parts: (1) training part, which lasts 2 days during which all 20 wells are baited with drinking water; (2) learning part which lasts 7 days and only one of the wells is baited (the same well for the entire week); (3) probe part which lasts 2 days during which a different well was baited. The mice were kept on water depravation (1 hour of water per day) during the entire experiment. Each animal's path was tracked and its latency to reach the well was recorded. In addition, the percentage of success in reaching the well was calculated. Significant differences were observed between the injured mice in comparison with the sham group following both time intervals. Seven days post mTBI the mice showed learning deficits in the learning part as well as in the probe part. Thirty days post mTBI, there were no significant differences in the learning part, but deficits were evident in the probe part. These results demonstrate that there are long term deficits in cognitive learning abilities in mTBI mice which are measurable in the highly sensitive paradigm of the dry maze.

# Abstract: Obsessive-compulsive disorder: practical application of animal behavior as a conceptual model in studying compulsive rituals in humans

**Authors:** D. Eilam, R. Zor, H. Szechtman, H. Hermesh

**Affiliations:** ^1^Department of Zoology, Tel-Aviv University, Israel; ^2^Geha Mental Health Center and Department of Psychiatry, Tel-Aviv University, Israel; ^3^Department of Psychiatry and Behavioural Neurosciences, McMaster University, Canada

A concept and methodology derived from animal studies was utilized as a framework for studying rituals in obsessive compulsive disorder (OCD) patients and deriving objective and observable criteria for compulsive rituals across patients. OCD rituals performed by patients in their own home were videotaped and compared with the behavior of healthy individuals instructed to perform the same rituals. The videotaped rituals were deconstructed into visits to specific locations or objects (ritual space), and to the acts performed at each location/object (ritual basic components). Quantitative analyses revealed that compulsiveness emanates from the expansion of repeats for some acts and visits, and from the addition of superfluous act types. Best discrimination between OCDand control rituals (90.9% success) was provided by the parameter “maximum of act repeats in a ritual” (R2 = 0.77). It is suggested that the identified properties of compulsive behavior are consistent with a recent hypothesis that ritualized behavior shifts the individual's attention from a normal focus on structured actions to a pathological attraction onto the processing of basic acts, a shift that invariably overtaxes memory. Characteristics and mechanisms of compulsive rituals may prove useful in objective assessment of psychiatric disorders, behavioral therapy, and OCD nosology.

*Supported by Adams Super Center for Brain Studies, Tel-Aviv University, Israel*.

# Abstract: Gene expression profiling in neuronalmodel of Gaucher disease

**Authors:** V. Einat, T. Farfel-Becker, A. Futerman

**Affiliations:** Department of Biological Chemistry, Weizmann Institute of Science, Rehovot, Israel

Gaucher disease is the most common lysosomal storage disorder (LSD). This metabolic disorder is caused by mutations in the gene encoding glucocerebrosidase (GlcCerase), and as a result, glucosylceramide (GlcCer) accumulates within the cell. Types 2 and 3, the most severe forms of the disease, are characterized by neurological impairment and neuronal cell death. Over the past few years, our laboratory has shown in neuronal models of Gaucher disease that Glc- Cer enhances agonist-induced Ca^2+^ -release from intracellular stores via the ryanodine receptor (RyR), and that the enhanced Ca^2+^ -release is directly responsible for neuronal cell dysfunction and death. Moreover, in in vitro studies, we have shown that GlcCer directly interacts with and modulates the activity of the RyaR, the major Ca^2+^ -release channel in the ER, and that this also occurs in human brain tissue obtained post-mortem from Gaucher disease patients. These findings suggest that defective Ca^2+^ -homeostasis may be a mechanism responsible for neuropathophysiology in acute neuronopathic Gaucher disease. However, it is still not clear how accumulated GlcCer leads to neuronal cell death. Thus, we are performing comprehensive gene arrays studies of cultured hippocampal neurons. We compared untreated neurons to Gaucher disease biochemical model neurons using conduritol-B-epoxide (CBE), an active site-directed inhibitor of GlcCerase. Our data suggest significant alterations in gene expression in this model. Among the genes that were transcriptionally regulated are genes related to nervous system development and genes involved in mitochondria function. These results will hopefully shed light on the pathways activated in neurons upon GlcCer accumulation, and will thus lead to a clearer picture of the cellular mechanisms involved in neuronal cell dysfunction and death.

# Abstract: Inferring the representation of arm movements in the human motor cortex from the fMRI signal

**Authors:** M. Eisenberg, L. Shmuelof, E. Vaadia, E. Zohary

**Affiliations:** ^1^Interdisciplinary Center for Neural Computation, The Hebrew University, Jerusalem, Israel; ^2^Department of Physiology, Hadassah Medical School, The Hebrew University, Jerusalem, Israel; ^3^Department of Neurobiology, Life Science institute, The Hebrew University, Jerusalem, Israel

Neurons in the motor cortex are typically tuned to the direction of limb movements. There's also evidence for similar tuning of the local field potential (LFP), representing the pooled neural activity (at roughly 1mm). But it is currently unclear if the different directions of movements are represented in clusters within the limb areas of the human brain. To that end, we conducted a functional MRI study in which subjects performed a reaching task towards 4 targets using an MRI-compatible joystick. Regions of interest (ROIs) were selected on the basis of their activation during the various reaching conditions. In each ROI, we studied the degree to which the spatial patterns of the fMRI activation (across all voxels within an ROI) were correlated across trials. The correlation coefficient was computed for trials repeating the same motion (“same” condition) and for trials with different directions of movement (“different condition”). We found that in the primary motor cortex, premotor cortex, supplementary motor area, and various regions along the intraparietal sulcus, the spatial pattern of fMRI activation was positively correlated across repetitions in the “same” condition, whereas the correlation between trials in different directions was slightly negative. The results indicate that a non homogenous representation of directional movements is present in the human motor areas. While, clearly, important information may be lost due to the low spatial resolution of fMRI and its inherent noise, one can still predict the direction of hand movement on a single trial from the fMRI signal at a level better than chance. This method may be a useful tool for investigating interactions and hierarchies of brain areas involved in sensorimotor action and perception.

*The first two authors contributed equally to this work*.

# Abstract: Patterns of brain language organization as a function of linguistic performance following recovery from childhood insults to the left hemisphere

**Authors:** O. Elkana, D. Ben Bashat, U. Kremer, T. Hendler, R. Fros, A. Schweiger

**Affiliations:** ^1^Psychology Department, Hebrew University, Jerusalem, Israel; ^2^Wohl Institute for Advanced Imaging, Sourasky Medical Center, Tel-Aviv, Israel; ^3^Pediatric Neurology, Sourasky Medial Center, Tel-Aviv, Israel; ^4^Behavioral Sciences, Academic College of Tel Aviv-Yafo, Israel

**Introduction**

Functional neuroimaging studies of aphasic stroke patients offer the prospect of a better understanding of the neuronal mechanisms that underlie language recovery. There is ongoing debate concerning the capacity of the right hemisphere (RH) to takeover language abilities after a focal brain lesion to the left hemisphere (LH).In this study, we investigate systematic patterns of brain language organization as a function of linguistic performance following recovery from childhood insults to the language-dominant hemisphere.

**Methods**

We present preliminary data of 7 children with a history of single, left hemisphere CVAs, who were diagnosed with nonfluent aphasia at onset (ages 6–17) and subsequently recovered. Twelve age- and sex-matched healthy right handers served as controls. All underwent fMRI scanning during three linguistic activation tasks, during the chronic stage (at least 2 years postonset).

**Results**

Outcomes yielded predominant tendency of RH Lateralization in patients versus LH lateralization in the healthy control group. The patterns of activation in the RH in patients were in brain areas homologous to LH regions involved in language functioning under normal circumstances. However, correlation analyses with linguistic performance demonstrated that better linguistic performance is associated with greater involvment of the left hemisphere. This finding held true for patients' linguistic performance inside the MRI scanner (for Rhyming and Comprehension tasks), as well as for behavioral linguistic mesurments which were carried out outside the scanner (verbal fluency, naming and reading).

**Conclusions**

These findings imply that the left hemisphere becomes more involved as the patient becomes more proficient at the linguistic task. Hence, it appears that better recoverymay reflect more involvement of perilesional regions of the damaged LH.

*Funded by the Israel Science Foundation (ISF).*

# Abstract: The role of PSD-95 in consolidation of taste learning

**Authors:** A. Elkobi, K. Belelovsky, L. Barki-Harrington, K. Rosenblu, I. Ehrlich

**Affiliations:** ^1^Center of Brain and Behavior, Department of Neurobiology and Ethology, Haifa university, Haifa 30905, Israel; ^2^Friedrich Miescher Institute, Maulbeerstrasse 66, 4057 Basel, Switzerland

Taste learning is correlative with phosphorylation of proteins regulating translation, in the brain area underlying its function. In addition, inhibition of protein synthesis in the gustatory cortex attenuates long but not short term taste memory. we employed two parallel different proteomic approaches to identify modulation on the protein expression levels in the gustatory cortex in correlation with novel taste learning. Both screening and additional direct methods identified and verified the synaptic protein, PSD-95 as an induced protein. The correlative induction of PSD-95 with learning is MAPK dependent and is also induced at synapses of the taste cortex. Moreover, the time window of PSD-95 increased expression is narrow and learning novel but not familiar taste induced its expression. This learning-related induction of PSD-95 was MAPK dependent, and was expressed locally at synapses of the taste cortex. To examine whether PSD-95 induction in the cortex was necessary for the taste memory trace formation, we attenuated PSD-95 expression in the gustatory cortex in vivo, by temporally and spatially restricted lentiviral mediated RNAi. Indeed, PSD-95 levels in the gustatory cortex were necessary for learning novel taste in thesiRNA injected rats.

# Abstract: Novel approach to inhibit inositol monophosphatase as an alternative to Lithium as amood stabilizer

**Authors:** Y. Eskira, I. Levi, O. Almog, G. Agam

**Affiliations:** ^1^Department of Clinical Biochemistry and Psychiatry Research Unit, Ben-Gurion University, Israel; ^2^Mental Health Center, Beer Sheva, Israel

**Background**

Lithium salts (Li) are the standard treatment for bipolar disorder. The “inositol depletion” hypothesis proposes that Li acts by depletion of brain inositol, based on the uncompetitive inhibition of inositol monophosphatase (IMPase) by Li, resulting in decreased inositol, and subsequent down regulation of the phosphoinositide cycle (PI). Berridge proposed that depletion of brain inositol levels and the consequent reduced rate of resynthesis of phosphatidilinositol and of second messenger signal generation leads to a decreased response to neurotransmission through this pathway. It has recently been shown that IMPase is an activation target for calbindin D28k. Purified calbindin attaches to a specific amino acid sequence on purified IMPase enhancing its activity by several fold. The effect of calbindin on IMPase activity is most meaningful at conditions that otherwise lead to low IMPase activity such as low substrate concentration. This suggests that calbindin could be a key endogenous regulator of the phosphatidylinositol cycle.

**Aim**

Find out whether short oligopeptides comprised from the IMPase sequence to which calbindin was shown to attach interfere with the interaction and abolish IMPase activation by calbindin.

**Methods**

IMPase activity in brain homogenates in the presence or absence of 60 mM Li (to discriminate between IMPase and nonspecific phosphatases), 20 *μ*M recombinant calbindin and with or without 10 *μ*M specific peptides was quantified spectrophotometrically bymeasuring Pi release fromthe substrate inositol-1-phosphate.

**Results and conclusion**

Calbindin's activation of IMPase by 1.66 fold was abolished by specific peptides derived from the sequence of the interaction site of the IMPase protein with calbindin but not by a scrambled peptide. This finding may lead to the development of molecules capable of inhibiting endogenous IMPase activity as an alternative mood stabilizing drugs.

*Supported by a Stanley Foundation grant to O. A. and G. A*.

# Abstract: Myoclonic bursting of the ankle joint musculature in spinal cord injury patients is attenuated by activation of crossed-inhibitory pathways

**Authors:** A. Etlin, B. Shamir, Y. Mor, D. Sasson, G. Zeilig, A. Lev-Tov

**Affiliations:** ^1^Department of Anatomy and Cell Biology, The Hebrew University Medical School, Jerusalem, Israel; ^2^Department of Neurological Rehabilitation, The Chaim Sheba Medical Center, Tel Hashomer, Israel; ^3^NBT Inc. Jerusalem Israel

Spastic myoclonus produced in spinal cord injury patients with incomplete thoracic lesions was studied using simultaneous video monitoring and surface EMG (s-EMG) recordings from the ankle joint antagonistic muscles tibialis anterior (TA) and lateral gastrocnemius (LG). Myoclonic activity has been revealed in the recorded muscles following dorsiflexion of the ankle joint, during body weight supported (BWS) treadmill locomotion, and after BWS locomotion. Statistical analysis of the myoclonic activity using Wavelet transform and coherence, showed that the myoclonus was characterized by a typical ∼6–8 Hz tremor and that the flexor activity (TA) was slightly led by the activity of the extensor (LG) during the dorsiflexion-induced myoclonus, but not during or following BWS treadmill locomotion. The myoclonus produced by dorsiflexion of the ankle could be attenuated in four out of the six patients by dorsiflexion of the contralateral ankle. We suggest that the myoclonus is not necessarily due to a repeated activation of stretch reflex and that it involves activation of spinal central pattern generators (CPGs). We also propose that activation of intact crossed inhibitory commissural pathways is capable of suppressing the contralateral myoclonus.

# Abstract: The Importins of Retrograde Transport in Injured Nerve

**Authors:** M. Fainzilber

**Affiliations:** Weizmann Institute of Science, Israel

The long axonal process of a neuron poses a problem when information on damage to the axon must be conveyed to the distant cell soma. It is known that injured peripheral nerve axons can signal to their cell bodies, and cause a “reprogramming” of the cell body for long range elongating outgrowth. We have recently discovered that the mechanism for this process is based on local synthesis of nuclear import proteins in the axon at the injury site. These proteins can then bind signaling proteins tagged with nuclear import signals, and transport them back to the cell body where they induce changes in the regenerative properties of the nerve. Our current efforts are focused on understanding how the system is regulated, what signals it transports, and what is the transcriptional response at the cell body. The presentation will focus on new data on regulation of the importins system in axons, initial identification of candidate signaling proteins, and characterization of the transcriptional response in neuronal cell bodies.

# Abstract: Establishment of a dominant submissive relationships model in Sabra outbredmice

**Authors:** Y. Feder, A. Ogran, A. Zinn, G. Yadid, A. Pinhasov

**Affiliations:** ^1^Department of Molecular Biology, Ariel University Center, Ariel, Israel; ^2^Leslie Susan Gonda Multidisciplinary Brain Research Center, Bar-Ilan University, Ramat-Gan, Israel

Depression is a multifactorial disease caused by both genetic and environmental factors and the prevalence of depressive disorders appears to be increasing in modern society. The major tools in the quest to reveal the molecular basis of depression and discover new antidepressants are animal models. Since depression in humans develops gradually and the effect of antidepressant treatment in clinics is effective only after chronic administration, development of animal models of chronic depression is essential. To meet this challenge, a Dominant Submissive Relationship (DSR) model was developed for mood stabilizing and antidepressant drug testing. In the DSR model pairs of animals compete during daily 5- min interactions for limited amounts of food. Under these conditions, about 30–40% of the pairs develop dominantsubmissive relationships that are characteristic of normal social behavior. Treatment of submissive animals with known antidepressants significantly reduced submissive behavior in a dose-dependent manner. Therapeutic efficacy was delayed in concordance with the delay of therapeutic efficacy of antidepressants routinely seen in clinic. These characteristics make the DSR model a valuable tool for study etiology of depression and for the testing of antidepressants. The purpose of this study is to evaluate ability of outbreed Sabra mice to develop dominant submissive relationships. Two month old male Sabra mice were subjected to the DSR test. The data show that about half of the animals developed strong and stable D-S relationships during second week of experiment that remain stable during third week of experiment. This study revealed that Sabra outbred mice are able to develop strong D-S relationships and may serve as a potential model for screening antidepressants and investigation of genetic elements that are responsible for dominant and/or submissive behavior.

# Abstract: Transplanted fibroblasts reduce neuronal synchronised bursting discharge and correct Parkinsonian asymmetry and L-Dopa-induced dyskinetic movements in 6-hydroxydopamine-lesioned rats

**Authors:** Y. Feld, J. P. Finberg, Y. Loboda, Z. Gluzman, O. Mohsen, M. Reshef, S. Marom

**Affiliations:** ^1^Genegrafts Ltd, Israel; ^2^Molecular Pharmacology Department, Rappaport Faculty of Medicine, Technion, Haifa, Israel; ^3^Physiology Department, Rappaport Faculty of Medicine, Technion, Haifa, Israel

Motor dysfunction in Parkinson's disease results from decreased striatal dopaminergic input, leading to increased neuronal activity in striatal output pathways, primarily GPi, SNr and STN. We are developing autologous fibroblast transplantation in these areas of neuronal hyperactivity as a potential treatment for Parkinson's disease, based on our observation that addition of dissociated fibroblasts to a primary culture of cerebral cortical neurons reduces synchronization of neuronal discharge. Primary cultures of rat cortical neurons were prepared and plated on multi-electrode arrays. Mature fibroblasts were added to the cultures and electrical recording was taken 3-4 days later. Discharge rate of cortical neurons cultured on multielectrode array was reduced to 32±5% control at 3 days following fibroblasts seeding. Unilateral 6-OHDA lesion in medial forebrain bundle was carried out in Fischer rats. Syngeneic dermal fibroblasts (200 000) were transplanted at entopeduncular nucleus (EP; rodent homologue of GPi) and SNr. Apomorphine-induced rotational behavior (0.25 mg/kg) was reduced 2, 4, 8, and 26 weeks following transplantation, to 22 ± 9, 28 ± 12, 30 ± 11, and 24 ± 7% control respectively (*P* < .01 at each time point, *n* = 4−6).Daily administration of L-Dopa (6 mg/kg) and carbidopa (1.5 mg/kg) caused a gradual increase in dyskinetic movements in sham-operated rats, which reached a peak at 3 weeks after commencing treatment. The intensity of dyskinetic movements was reduced in rats which had been transplanted with fibroblasts in STN or EP. In histopathological analysis performed 7 months following transplantation the transplanted cells were located at the site of transplantation without signs of significant inflammation. We suggest that this technology can serve as a broad platform for the treatment of various neurological disorders in which local areas of inappropriately high neuronal activity exist, and specifically for Parkinson's disease.

# Abstract: Distinct involvement of the endothelin system in glial inflammatory pathways

**Authors:** T. Filipovich, S. Fleisher-Berkovich

**Affiliations:** Ben Gurion University of the Negev, Department of Clinical Pharmacology, Israel

The endothelin system, comprising of three 21-amino acid peptides, endothelin ET-1, ET-2, ET-3 and their receptors ETA and ETB, are synthesized and expressed in various cells and tissues, including the brain. Evidence suggests that endothelins may play a key role in the regulation of inflammatory process under pathological conditions such as Alzheimer's disease. We investigated the role of endothelins (ET-1, ET-2, ET-3) in the regulation of prostaglandin E2 (PGE2) and nitric oxide (NO) synthesis, which are important mediators of inflammatory responses. This was studied both under basal conditions and in lipopolysaccharide induced neonatal rat glial cells. Under basal conditions, all three endothelins, given at concentrations of 10nM and 100 nM, increase PGE2 and NO release by about 2-3 fold in a dose dependent manner. However, in LPS induced cells, all endothelins decrease the production of PGE2 and NO to about one half. BQ123 and BQ788, antagonists of ETA and ETB receptors, respectively, completely blocked both the stimulatory and inhibitory endothein actions. These results demonstrate for the first time the dual ability of endothelins, acting via ETA and ETB receptors, to influence the production of inflammatory mediators in glial cells under basal and inflammatory conditions. Targeting the ET system may be a promising approach in modifying the course of inflammation during AD.

*This research was supported by the Israel Science Foundation (Grant no. 500/04)*.

# Abstract: Cholinergic involvement in sensory-induced activation of locomotor networks in the mammalian spinal cord

**Authors:** E. Finkel, D. Blivis, A. Etlin, A. Lev-Tov, L. Anglister

**Affiliations:** Department of Anatomy and Cell Biology, Hebrew University Medical School, Jerusalem 91120, Israel

Stimulation of sacrocaudal afferents (SCA) has been found to induce a locomotor-like and body-axis stabilizing rhythm in the isolated spinal cord of the neonatal rat and mouse. The SCA-induced rhythm depended on synaptic activation of sacral interneurons whose axons cross the cord and ascend rostrally mainly through the contralateral ventral funiculus (VF). In the present work we have used fluorescent labeling techniques and confocal microscopy to identify these interneurons and characterize their association with the cholinergic system that has been shown to produce locomotor like episodes in the isolated spinal cord. Retrograde fluorescent labeling of the cut VF at the lumbosacral junction revealed several groups of S1–S4 interneurons contralateral to the fill. Labeled interneurons were mostly located in the intermediate gray, a few in the dorsal horn, and some in the ventral horn. The laminar locations and trajectories of these neurons are similar to those of projection neurons of the spinothalamic, spinoreticular, and spinocerebellar tracts or of ascending propriospinal pathways. Immunocytochemical labeling of the cholinergic transmission components, choline acetyltransferase (ChAT), vesicular ACh-transporter, acetylcholinesterase and muscarinic AChRs, revealed that subpopulations of these sacral interneurons produced and/or received cholinergic innervation. Further studies are required to clarify the contribution of these cholinergic elements to pattern generation and inter-segmental communication in the mammalian spinal cord.

# Abstract: Effect of chronic stress and fluoxetine therapy on IGF-1 receptor expression in the brain and on behavioral and cognitive parameters of rats

**Authors:** M. First, I. Tarasenko, M. Taler, N. Grunbaum-Novak, R. Weizman, I. Gil-ad, A. Weizman

**Affiliations:** ^1^Lab of Biological Psychiatry, FMRC and Sackler Faculty of Medicine, Tel Aviv University, Israel; ^2^Brull Community Mental Health Center, Sackler Faculty of Medicine, Tel-Aviv University, Israel; ^3^Research Unit, Geha Mental Health Center, Israel

Chronic mild stress (CMS) in rats is a widely used model of depression, which is linked to stress-induced decreases of neurogenesis in the hippocampus. Antidepressant treatments appear to protect against hippocampal volume loss. Moreover, antidepressants administration reverses the behavior of stressed animals such as anhedonia (decreased sucrose preference) and increased immobility in the forced swim test. Our goals were to evaluate the effect of chronic mild stress and fluoxetine (5 mg/kg/day i.p.) therapy on the IGF1 system in the rat brain and on behavioral and cognitive parameters. Male rats were exposed sequentially, over a period of 3 weeks, to a variety of mild stressors (Wilner 1997), and another control groups of rats received the same treatment without stress. Body weight gain of fluoxetine treated rats did not differ from the control group. After 3 weeks of CMS animals were exposed to the open field (OF) test followed by a Morris water maze (MWM). In the OF fluoxetine treated stressed rats showed decreased immobility, and increased frequency and duration time spent in the inner zones of the arena, suggesting anxiolytic and more motivated behavior. However, in the MWM we observed a significant facilitating effect of fluoxetine in the acquisition phase in the nonstressed animals only. After autopsy we dissected brains and separated the cortex and hippocampus. IGF- 1 receptor (IGF-1R) expression was determined by Western blot analysis. We found that CMS significantly reduced the IGF-1R levels in the cortex and the hippocampus, and fluoxetine treatment reversed the expression to normal levels. We suggest that decreased IGF-1R in the brain following CMS may contribute to depression and anxiety, which are reversed by fluoxetine therapy. However, fluoxetine therapy slightly improved performance in the MWM in normal but not in stressed rats suggesting that more factors are involved in the process of improving cognition.

# Abstract: Neural correlates of visual recognition revealed through backwardmasking and intracranial electrode recordings

**Authors:** L. Fisch, E. Privman, U. Kramer, S. Kipervasser, F. Andelman, M. Neufeld, I. Fried, R. Malach

**Affiliations:** ^1^Department of Neurobiology, Weizmann Institute of Science, Rehovot 76100, Israel; ^2^Sackler School of Medicine, Tel Aviv University, Tel Aviv 69978, Israel; ^3^EEG and Epilepsy Unit, Department of Neurology, Tel Aviv Medical Center, Tel Aviv 64239, Israel; ^4^Functional Neurosurgery Unit, Tel Aviv Medical Center, Tel Aviv 64239, Israel; ^5^Division of Neurosurgery, David Geffen School of Medicine, Semel Institute for Neuroscience, UCLA, USA

Backward masking—where visual images are briefly (10– 100 milliseconds) flashed and followed by a mask, often rendering them unrecognizable—offers an opportunity to study neural correlates of object recognition. Previous fMRI studies of this phenomenon have revealed that recognition is tightly correlated to high BOLD activity in ventral stream visual areas. Here, we used intracranial EEG (iEEG) recordings, which offer good localization and a msec temporal resolution, to investigate in more detail the neuronal events which correlate to recognition in this paradigm. Recordings were done using subdural intracranial electrodes, implanted in epilepsy patients strictly for clinical purposes. Patients viewed a briefly (16 milliseconds) presented target image belonging to one of three categories—faces, houses, and tools—followed by a fixed mask image. Interposed between target and mask was a blank period of variable duration, resulting in a total time difference (SOA) of 16– 200 milliseconds between target and mask onset. The subject had to overtly name the target's category. Speech utterances were recorded. Working at threshold SOA, where image recognition was successful in only about half the trials, allowed comparison between trials identical in terms of objective parameters (SOA and retinal stimulation) but differing in the subject's state of recognition. Our results showed a drastic recognition-associated increase in LFP parameters. The amplitude of gamma activity was highly correlated to the state of recognition in a number of electrodes. Interestingly, the duration of these gamma power increases often outlasted the image exposure duration. In other electrode sites, recognition-related changes were more prominent in the shape and amplitude of the N170 evoked potential recorded. These effects were only found in electrodes that showed a clear object selectivity. The results point to the potential power of intracranial recordings to uncover neuronal correlates of perceptual awareness.

*Supported by ISF grant to R. Malach and US-Israel BSF grant to Malach and Fried*.

# Abstract: PhysiologicalMechanisms of Neurodegeneration Induced by Activity Deprivation

**Authors:** I. Fishbein, E. Schonfeld, V. Greenberger, M. Segal

**Affiliations:** Department of Neurobiology, Weizmann Institute of Science, Israel

When deprived of spontaneous network activity by chronic exposure to tetrodotoxin (TTX), cultured cortical and striatal neurons die over a period of one to two weeks. Prior to their death neurons retract their dendrites, lose dendritic spines yet express a two fold increase in miniature excitatory postsynaptic currents (mEPSCs). Unlike TTX treatment, chronic blockage of glutamate receptors does not have a toxic effect of its own. Moreover, blocking the AMPA subtype of the glutamate receptor protects TTX-silenced neurons from degenerating, indicating that mEPSCs becomes toxic to chronically silenced neurons. Further investigation revealed that chronically silenced neurons are impaired also in their ability to clear out calcium from the cytoplasm, which may explain their vulnerability to relatively small calcium changes. The molecular mechanism of TTX-induced neuronal death exhibited some apoptotic characteristics such as the upregulation of the Bax and Bak genes, mitochondrial fission and DNA fragmentation. On the other hand, it lacked caspase-3 activation, indicating that a caspase independent form of apoptosis is activated once action potentials are blocked. We conclude that neuronal activity is beneficial to neuronal survival only when it involves spontaneous trains of action potential, and that in the absence of network activity, subthreshold synaptic currents are in fact toxic and may set in motion mechanisms of programmed cell death.

# Abstract: Unsupervised learning in network of temportrons

**Authors:** D. Fisher, M. Tsodyks, H. Sompolinsky

**Affiliations:** ^1^Neurobiology Department, Weizmann Institute of Science, Rehovot 76100, Israel; ^2^Faculty of Physics, Weizmann Institute of Science, Rehovot 76100, Israel; ^3^Racah Institute of Physics and ICNC, Hebrew University, 91904 Jerusalem, Israel; ^4^Center for Brain Science, Harvard University, CambridgeMA 02138, USA

Tempotron learning rule [1] for a leaky integrate-and-fire (LIF) neuron provides a powerful method for learning and decoding time- and synchrony-based neuronal code. Tempotron learning rule is supervised, that is, the modification of LIF input synaptic strengths is governed by the external teaching signal which specifies the correct spiking behavior for each given input. However, in biological systems the learning of temporal code is not necessarily supervised. In present work we design architecture and learning rules for a small LIF network capable of unsupervised (self-supervised) learning of temporal neuronal code. The learning rule for every LIF is based on the tempotron learning rule with additional long-term synaptic depression. Recurrent connectivity and signaling rules provide for selfsupervision in the network. As an example, we demonstrate the capability of this “network of tempotrons” to distinguish between Poisson-spiking representations of two or more different spatio-temporal sequences with identical average spike counts in each channel.

## References

[1] Gütig R, Sompolinsky H (2006). The tempotron: a neuron that learns spike timing-based decisions. *Nature Neuroscience*.

# Abstract: Silencing plus over-expression of selected genes as a novel model of sporadic Parkinson's disease

**Authors:** T. Fishman, M. B. H. Youdim, S. Mandel

**Affiliations:** Eve Topf Center, Technion, Department of Pharmacology, Faculty of Medicine, Israel

Our objective is to develop a model of sporadic PD by selectively silencing genes identified in our transcriptomic and protein profiling of human SNpc from sporadic PD brains, while enforcing the expression of others. The mouse substantia nigra-derived cell-line SN4741 was employed as a model to study the involvement of the identified proteins intimately connected to the neurotoxic cascade (e.g., SKP1A, HSC-70 and ALDH1A) and the response to cell injury induced by the dopaminergic neurotoxin MPP+, serum deprivation and H2O2. SN4741 cells were infected with small-hairpin RNA (shRNA)-encoding lentiviruses targeting the selected transcripts or with nontarget shRNA control (scramble). The percentage of silencing was evaluated using real-time PCR and Western blot analysis with specific antibodies. Parameters of cell survival were assessed. Alterations in cell cycle were analyzed by flow cytometry. MPP+ andH2O2 induced a time and dose-dependent alteration in the protein expression of the oxygen and iron-regulated proteins transferrin receptor and prolyl hydroxylase (PHD2/EGLN1) and decreases in SKP1A, HSC-70 and ALDH1A. ShRNA-mediated silencing of Skp1A, a component of the SCF ubiquitin (E3) ligase complex, showed a significant delay in completion of the cell cycle and an increased susceptibility to MPP+ and serum starvation. The approach to develop an “ultimate model” of PD by silencing or over expressing single genes that were identified asmutated in the familial cases has notmet with the expected success. We have reproduced to a significant extent the gene and protein alterations initially described in our studies with SNpc from Parkinsonian subjects, in a dopaminergic cell line from mice SNpc. The successful results obtained with the present study will help us to develop an experimental mice model of sporadic PD based on viral-mediated RNAi delivery which will be of help for developing novel neuroprotective drugs with possible disease modifying activity.

# Abstract: A novel mechanism regulating the synthesis of the NMDA receptor coagonist D-serine: implications for NMDA receptor activation

**Authors:** V. N. Foltyn, L. F. Balan, E. Dumin, H. Wolosker

**Affiliations:** Department of Biochemistry, Faculty of Medicine, Technion-Israel Institute of Technology, Haifa 31096, Israel

The NMDA-type of glutamate receptors play major roles in excitatory neurotransmission, synaptic plasticity and memory formation. Data from several laboratories have shown that the unusual D-amino acid, D-serine, is a physiological regulator of NMDA receptors by binding to the “glycine site” at the NR1 subunit. Brain D-serine is synthesized from Lserine by the serine racemase enzyme. Serine racemase was shown to be expressed in astrocytes and recently demonstrated in neurons as well, but little is known on its regulation. We now explored mechanisms regulating serine racemase enzyme and D-serine synthesis in the brain. Despite the fact that serine racemase lacks transmembrane domains, a significant portion of the enzyme is tightly bound to detergent-resistant membranes from brain. Immunocytochemical experiments revealed highest levels of membranebound serine racemase at the plasma membrane of neuronal processes. We found that membrane association of serine racemase is mediated by palmitoylation, which leads to almost complete inactivation of the enzyme. Treatment of primary cultures with NMDA increased several fold the levels of membrane-bound serine racemase by enhancing the palmitoylation of the serine racemase enzyme. Our data indicates that NMDA receptor activation promotes feedback inhibition of serine racemase to downregulate D-serine levels. Palmitoylation of serine racemase provides a novel mechanism regulating D-serine synthesis, with implications for NMDA receptor transmission.

# Abstract: Development of the endocannabinoid system: its critical importance for embryonal implantation, neuronal development and postnatal suckling; implications of its pre- or postnatal manipulation

**Authors:** E. Fride

**Affiliations:** Ariel University Center of Samaria, Israel

Cannabinoid (CB1,CB2) receptors are activated by delta- 9-tetrahydrocannabinol (THC), the major biologically active ingredient of the Cannabis sativa plant, or by endogenous (endo)cannabinoids such as anandamide and 2- arachidonoyl glycerol (2AG). The“endocannabinoids” and their synthesizing and degrading enzymes as well as the CB receptors form the“endocannabinoid” system (ECS) which appears to be an important regulator of many (neuro)physiological functions including feeding, appetite, motor functions and inflammation. The ECS has also been shown to play an essential role in several developmental functions. Thus high levels of anandamide degradation enable uterine implantation of the embryo. Further it has been shown that the endocannabinoids and their receptors play a fundamental role in axon guidance and synaptogenesis during mid gestation. During postnatal life, CB1 receptor activation in the newborn is essential for the initiation of milk suckling. Thus we have shown that two different CB1 receptor antagonists (SR141716 and VCHSR1) impair mouse pup development and suckling, when administered within 24 h of birth. Further, intracerebral injections of SR141716 also impair pup development but only if injected at the base of the brain, suggesting a role for hypothalamic or basal ganglia receptor activation in pup suckling. Finally, prenatal marihuana, THC or anandamide exposure have been shown to affectmidgestational weight and the development of the prefrontal cortex, dopamine system and CB1 receptors in the mature offspring. In conclusion, the ECS fulfills many physiological functions in the mature organism. However, only at several junctions during development, seems the ECS to play a critical role. Therefore, the repercussions of pre- or postnatal exposure to (endo)cannabinoids should be studied more extensively.

# Abstract: Characteristics and possible role ofmicrosaccades during visual fixation

**Authors:** M. Fried, A. Arieli

**Affiliations:** Weizmann Institute of Science, Israel

The role of fixational eye-movements (FeyeMs), which are composed of drifts, tremors and microsaccades (MSs), in visual perception is controversial. There is a long-standing debate about MSs whether they contribute to vision. A recent debate is whether MSs and the so-called saccadic-intrusions (SI) are different names to the same phenomenon. We have recorded systematically FeyeM at high resolution from 4 naїve human subjects under various conditions. We report here that (i) in all subjects spontaneous MSs are primarily in the horizontal plane (80.1%: 2766 Horizontal MS with median amplitude of 0.34 degrees versus 687 Vertical MS with median amplitude of 0.12 degrees); (ii) voluntary saccades are often accompanied by an overshooting component which is larger in the horizontal direction. This overshooting component is independent of the size and duration of the saccade, but has the same size and duration distributions as the spontaneous MSs, and thus might be generated by the same mechanism generating spontaneous MSs; (iii) moving elements in the background reduce the amount of spontaneous MSs, in a direction specificmanner, suggesting thatMSs have a role in vision thatmight be related to the sensed image; (iv) the kinematics of MSs and SIs are similar. These results support the claim that while a single MS changes the position of the eye, SI is composed of a sequence of two or more MSs which eventually return the eye to the original position.

*This work was supported by the German Federal Ministry* *of Education and Research, Israeli Ministry of Science and Technology Foundation.*

# Abstract: Improved cognition in female triple transgenic AD mice treated with lipophilic metal chelators is associated with reduced astrocyte activation, amyloid-Beta, and p-tau pathology

**Authors:** J. E. Friedman, R. Eilam, K. V. Madala Halagappa, M. P. Mattson, A. I. Bush, P. A. Adlard, Y. David, A. Kozak

**Affiliations:** ^1^D-Pharm Ltd., Rehovot, Israel; ^2^Department of Vet. Resources, Weizmann Inst. Sci., Rehovot, Israel; ^3^Laboratory of Neurosciences, Natl. Inst. on Aging Intramural Res. Program, Baltimore, Md, USA; ^4^Oxidation Disorders Laboratory, The Mental Health Research Institute of Victoria, Parkville, Victoria, Australia

Transition metals such as zinc and copper contribute to the pathologies inherent in Alzheimer's disease (AD). This includes not only zinc-dependent amyloid-beta aggregation and precipitation but also astrocyte activation, release of inflammatory cytokines and tau hyperphosphorylation. Treatment with lipophilic chelators (neuron 30, 665, 2001; neurobiol; aging 25:1315, 2004) has been shown to reduce the plaque load and level of insoluble ABeta. Lee et al. have shown (PNAS 99:7705, 2002) in Tg2576 mice that synaptic zinc is gender dependent, being greater in females, and is correlated with significantly higher levels of ABeta plaques and insoluble ABeta. 18-month-old 3xTgAD mice that express mutations in presenilin-1, APP and tau, demonstrate severe memory deficits when tested in the Morris water maze (MWM). We have found that female 18 month-old 3xTgAD mice have greater deficits than do males, never achieving a significant improvement in performance over an 8-day period. This correlates with higher levels, in females, of p-tau- 2.8-fold on immunoblots from cortical extracts and higher astrocyte activation (GFAP immunostaining) than do similarly aged males,*P* <.01. Performance in females is improved by treatment with the membrane activated chelators (MACs) DP-109 and DP-460, resulting in significant learning within 3days (*P* < .01). P-tau immunoreactivity in female mice, following treatment with MACs, was significantly reduced in the same areas that GFAP staining was reduced. Synaptophysin staining was increased, indicating increased synaptic densities, particularly in the cingulate cortex of females (*P* < .05) in response to treatment, possibly explaining the improvement inMWMbehavior. Our data suggest that amelioration of cognitive deficits in aged 3xTgAD mice treated with MACs is associated with a multifaceted effect of MACs on the pathologies characteristic of AD, that is, reducing the toxic ABeta species, inhibition of astrocyte activation and tau hyperphosphorylation.

*JEF, YD, and AK are employees of D-Pharm*.

# Abstract: Recruitment order coding in large-scale recurrent networks of cortical neurons

**Authors:** A. Gal, D. Eytan, S. Marom

**Affiliations:** ^1^Network Biology Laboratories, Technion, Haifa, Israel; ^2^Interdisciplinary Center for Neural Computation, The Hebrew University, Jerusalem, Israel

Perhaps the most severe constraint on the nature of neural representation is the multiplicity and wide range of timescales that are characteristic of processes underlying neuronal excitability and synaptic communication. We suggest that under this constraint, the order in which neurons are recruited following a stimulus is an invariant, information-carrying statistic of response features. We demonstrate experimentally that in spontaneously formed networks of cortical neurons in vitro, the information that the response carries about the stimulation source is preserved when precise times to first-spikes are reduced to recruitment order. We looked into the nature of order coding by considering its dependence upon precise firing times of individual neurons and correlations between pairs of neurons.

# Abstract: Venomous wasp targets different head ganglia to manipulate specific behaviors of its cockroach prey

**Authors:** R. Gal, F. Libersat

**Affiliations:** Ben-Gurion University of the Negev and the Zlotowski Center for Neuroscience, Israel

The parasitoid wasp A. compressa manipulates cockroaches to serve as live food supply for its offspring. To do so, the wasp injects a venom cocktail specifically into two ganglia located in the head of the cockroach prey. This sting evokes two unique behavioral effects on the stung cockroach: first, the cockroach grooms spontaneously and excessively for 30 minutes. Second, the cockroach enters a long-lasting hypokinetic state during which it does not initiate spontaneous locomotion and is less responsive to aversive stimuli. The goal of the present study was to identify which of the two cockroach head ganglia, namely the supra- and subesophageal ganglia, is responsible for which of the two venom effects. To achieve this goal we manually injected crude venom (300 ug/ul, or saline in controls) into either ganglion (115 nl and 70 nl, respectively) and quantified the behavioral outcome in an open-field arena. Venom injected directly into the supraesophageal ganglion significantly increased spontaneous grooming and general locomotion without affecting responsiveness to aversive stimuli. In contrast, venom injected directly into the subesophageal ganglion significantly decreased general locomotion and responsiveness to aversive stimuli but did not initiate grooming. We therefore conclude that the two distinct venom-induced behavioral effects observed in cockroaches stung by A. compressa can be traced, at least in part, to specific ganglia in the cockroach head. Our results suggest that the venom induces excessive grooming by manipulating neuronal circuits in the supra-esophageal ganglion and the hypokinetic state by manipulating neuronal circuits in the subesophageal ganglion.

# Abstract: Sensory Adaptation to Different Stimulus Strengths in Rat Whisker to Barrel Pathway

**Authors:** E. Ganmor, Y. Katz, V. Mazig, J. Heiss, I. Lampl

**Affiliations:** Department of Neurobiology, Weizmann Institute of Science, Israel

Standard short term synaptic plasticity models (Tsodyks and Markram, PNAS 1997) predict that higher probability of release will entail more rapid adaptation. In layer 4 of the somatosensory cortex adaptation is believed to result from short term synaptic depression of thalamocortical synapses. We observed in vivo that both excitatory and inhibitory inputs (estimated using intracellular recordings) evoked by strong whisker stimulation adaptmore than the input evoked by weaker stimulation. Assuming that stronger whisker stimuli increase presynaptic firing rate and are associated with higher probability of release in the synapses of the sensory pathway, these results cannot be explained by synaptic depression mechanisms. In an attempt to explain this observation we recorded from single units in the ventro posterior medial thalamic nucleus (VPM) and applied the same stimuli. Although VPM firing adapted profoundly less than cortical synaptic inputs, PSTH of VPM cells showed similar stimulus strength dependent adaptation rate providing an explanation to the results observed in the cortex. This, nevertheless, raised the question of how the phenomenon occurs in the VPM. Next we investigated a possible role for the reticular thalamic nucleus (RT), which provides feedback inhibition to VPM cells, in the observed stimulus strength dependent adaptation rate. Paired recordings of single VPM units and multiunit activity in the RT before and after electrolytic lesion of the RT show that the RT plays a major role in shaping the adaptation of VPM cells as previously shown ([1]). However, even following RT lesion stimulus strength dependent adaptation rate was observed, suggesting that RT-VPM interplay cannot explain the counterintuitive phenomenon, and points to the trigemino-thalamic synapse as the next suspected sight which may explain this behavior.

*This work was supported by The Israel Science Foundation Grant 1037/03*.

## References

[1] Castro-Alamancos MA (2002). Different temporal processing of sensory inputs in the rat thalamus during quiescent and information processing states in vivo. *Journal of Physiology*.

# Abstract: Robustness and structure of 2nd-ordermaximum entropymodels describing the state distribution of neural populations

**Authors:** E. Ganmor, R. Segev, E. Schneidman

**Affiliations:** ^1^Department of Neurobiology, Weizmann Institute of Science, Israel; ^2^Department of Life Sciences, Ben Gurion University of the Negev, Israel

One of the key difficulties in describing and analyzing the simultaneous spiking activity of large groups of neurons, is that the number of possible activity patterns of the group grows exponentially with the size of the population. We have recently shown that the minimal mathematical model that relies only on the pairwise relations between cells but assumes no higher-order interactions gives a surprisingly accurate description of the distribution of network states in several neural systems ([1]). Notably, these maximum entropy models ([2, 3]) are defined by only a quadratic number of parameters. This study investigates the properties of these 2nd order models for describing network state distribution. Using joint recordings of dozens of retinal ganglion cells responding to natural and artificial movies, we tested several different simplified 2nd order models and approximations. While a model assuming independence of single neurons clearly fails, a proper choice of pairwise interactions allows for an accurate description using a number of parameters which is linear in the size of the network. Furthermore, although coincident firing is rare relative to the firing rate of single neurons, learning the pairwise model from samples converges at a rate similar to that of the independent model. The pairwise model proved to be the best model for windows shorter than 20 seconds. We used graph theoretical tools to explore the detailed structure of the networks ([4]), and found several local subnetwork structures that are over represented in the studied networks. This may help in further reducing the complexity of accurate models for these networks. Finally we show that the pairwise model allows us to discriminate between different visual stimuli based on single trial analysis.

*This work was supported by the Center for Complexity Science (RS and ES)*.

## References

[1] Schneidman E (2006). *Nature*.

[2] Jaynes ET (1957). Information theory and statistical mechanics. *Physical Review*.

[3] Schneidman E, Still S, Berry MJ, Bialek W (2003). Network information and connected correlations. *Physical Review Letters*.

[4] Milo R, Shen-Orr S, Itzkovitz S, Kashtan N, Chklovskii D, Alon U (2002). Network motifs: simple building blocks of complex networks. *Science*.

# Abstract: The involvement of stress and cannabinoid CB1 receptors in extinction of inhibitory avoidance in the amygdala

**Authors:** E. Ganon, I. Akirav

**Affiliations:** Department of Psychology, University of Haifa, Israel

The ability to extinguish emotional responses in the face of a no-longer relevant conditioned cue is an essential part of a healthy emotional memory system. Despite the efficacy of behavior therapy for anxiety disorders, extinction-like treatments require repeated cue exposures and are vulnerable to reversal by a number of environmental factors, particularly stress. The endocannabinoid system and CB1 receptors in particular, has recently emerged as considerably important in the regulation of emotionality and stress, and in extinction of fear-related learning. CB1 receptors are highly expressed in the amygdala, which is known to be important for anxiety and emotional learning. The main purpose of this study is to examine the effects of exposure to a stressful experience on inhibitory fear avoidance extinction, and the potential involvement of CB1 receptors in these memory processes in the amygdala. We found that exposure to a stressful experience of placing rats on an elevated platform for 30 min impaired consolidation of extinction of inhibitory avoidance. Injecting rats with a low dose of the CB1 agonist WIN55,212-2 (0.25 mg/kg) prior to exposing them to the stressor, resulted in intact extinction. Thus, the CB1 agonist reversed the impairing effects of the stressor on extinction learning. Importantly, we were able to localize the blocking effect of the CB1 agonist to the amygdala; accordingly, WIN55,212-2 (6 ng/0.5 ml) reversed the impairing effects of the stressor on extinction when microinjected to the amygdala before placing rats on the elevated platform. These results may support a wide therapeutic application of cannabinoids in the facilitation of extinction therapy that is often accompanied by stressful life events.

# Abstract: Single cell resolution network dynamics in the rat barrel cortex using calcium imaging two-photon microscopy in vivo

**Authors:** L. Garion, I. Pehter, R. Azouz, J. Schiller

**Affiliations:** ^1^Techion Medical School, Israel; ^2^Ben-Gurion University, Israel

One of the major challenges in studying the function of sensory circuits is to understand how the representation of complex, natural stimuli arises fromthe basic response properties of neurons. Rodents in their natural environment use their whiskers to distinguish between surfaces having subtly different textures and shapes. They do so by actively sweeping their whiskers across surfaces in a rhythmic forward and backward motion. While it is widely accepted that the brain “reconstructs” these complex sensory stimuli from the information contained in the concerted activity of ensembles of neurons, the nature and mechanisms underlying this sensory information processing remains largely unknown. To determine the role of neuronal interaction in coding of tactile information we employed two-photon laser ScanningMicroscopy in combination with loading of the cortex with membrane permeable calcium sensitive indicators (AM dyes) which is loaded in a large fraction of the neurons in the layer 2/3 network. This new method enabled us to simultaneously measure from many cells in the activated network. Upon effective tactile stimulation the electrical activity of neurons in the network were monitored via the calcium signal evoked by the action potentials. To measure from large number of neurons and yet keeping good temporal resolution we developed a new scanning methods that scans a random line scan path that was defined a priori by the user. We have been able to record calcium transient signals from tens of neurons up to 400 *μ*m deep in the tissue. We find that the spatiotemporal pattern of activation was critically dependent on the nature of the stimulation delivered.

# Abstract: Learning temporal representations through reward dependant expression of synaptic eligibility traces in recurrent networks

**Authors:** J. Gavornik, Y. Loewenstein, H. Shouval

**Affiliations:** ^1^University of Texas Medical School at Houston, Tex, USA; ^2^Hebrew University, Israel

Experimental recordings have demonstrated that neurons in the primary visual cortex are able to learn to express temporal intervals as a function of reward timing (Shuler and Bear, 2006). The form of this expression is a stimulus evoked period of transient activity that persists until the expected reward time. It is not known how biological networks are able to learn temporal representations with time scales that are orders of magnitude larger than the intrinsic excitability time constants of individual neurons. We have previously developed a synaptic plasticity rule that allows a network with recurrent lateral connections to learn synaptic weights sufficient to set network decay times that correspond with trained temporal intervals by expressing novel plasticity eligibility traces, called protoweights, as permanent lateral recurrent synaptic weights. We demonstrate that our rule is able to encode multiple intervals in both deterministic networks of passive integrator neurons and noisy stochastic networks of nonlinear integrate and fire neurons. An extension to non orthogonal inputs is examined. Synaptic expression in our model is modulated by an external reward signal that is inhibited by activity at the network or single neuron level, this distinction has implications for possible biological mechanisms and possible consequences under different experimental conditions. We examine the temporal structure of both noise and stimulus evoked correlations, in order to make experimentally testable predictions for key elements of our encoding and learning paradigms.

# Abstract: Involvement of the opioid system in the decreased responsiveness induced in cockroaches stung by a parasitoid wasp

**Authors:** T. Gavra, R. Gal, F. Libersat

**Affiliations:** Ben-Gurion University of the Negev and the Zlotowski Center for Neuroscience, Israel

Cockroaches stung in the brain by the parasitoid wasp Ampulex compressa enter a long-lasting hypokinetic state. This state is characterized by decreased responsiveness to aversive stimuli, suggesting a manipulation of a neuromodulatory system in the cockroach brain. A likely candidate is the opioid system, which is known to affect responsiveness to stimuli in insects. The aim of the present study was to explore this possibility. To achieve this goal we injected different opioid-receptor antagonists (Naloxone, nonspecific; Nor-BNI, kappa-receptors; Naloxonazine, mu-receptors; ICI 174,864, delta-receptors; or saline in controls) to cockroaches prior to stinging by A. compressa. We then tested the responsiveness of these cockroaches to electric foot shocks in a shuttle box. All opioid-receptor antagonists significantly decreased the avoidance threshold of stung cockroaches to foot shocks. The most profound effect, however, was achieved with the kappa-specific antagonist Nor-BNI. These results suggest that the venom of A. compressa might manipulate responsiveness in stung cockroaches by affecting the opioid system predominantly via kappa-opioid receptors. If true, to our knowledge, this would be the first evidence of a behavioral manipulation of one insect by another mediated via the opioid system.

# Abstract: BL-1020, first in class GABA enhanced antipsychotic candidate: results of a D2 receptor occupancy study in humans

**Authors:** Y. Geffen, L. Appel, M. Davidson, S. Kapur

**Affiliations:** ^1^BioLineRx Ltd, Jerusalem, Israel; ^2^GEHC Uppsala Imanet, Sweden; ^3^Sheba Medical Center, Israel; ^4^Department of Psychological Medicine, Institute of Psychiatry, King's College London, UK

**Background**

BL-1020 is a novel compound composed of the dopamine- 2 (D2) antagonist Perphenazine and the inhibitory neurotransmitter GABA (gamma-amino-butyric acid), indicated for the treatment of schizophrenia. Preclinical studies show that BL-1020 delivers GABA to the brain, increases cortical dopamine, binds to GABA-A receptors and effectively reduces psychotic behavior with significantly fewer side effects than Perphenazine. A single dose escalating, double blind, placebo controlled Phase 1 trial, in 48 healthy volunteers indicated that BL-1020 is safe, well tolerated with a wide therapeutic window, an MTD phase IIa, 6 weeks, study in schizophrenic patients is ongoing.

**Method**

A PET study to assess the degree of D2 receptor occupancy in the human brain was conducted in healthy male volunteers, following administration of BL-1020, using [11C] raclopride as PET tracer. The trial (Uppsala's Imanet, Sweden), consisted of 12 healthy volunteers (3 cohorts of *n* = 4) that received a single oral administration of BL-1020 (16, 24, and 32 mg). PET scans were done at baseline, 6 hours, and 24 hours post dosing. The volunteers were also monitored for safety, PK and changes in plasma prolactin levels.

**Results**

PET data showed that BL-1020 caused a dose dependent D2 receptor occupancy in the striatum. Oral administration of BL-1020 (32 mg) resulted in occupancy of 50% at 6 hours. PK modeling suggests that this dose is sufficient to obtain D2 occupancy greater than 70% in a multiple dosing scenario, and thus should be within the therapeutic window. There was a linear correlation between plasma prolactin levels and estimated striatal D2 occupancy. There were no significant changes in ECG, vital signs, physical examination or laboratory values.

**Conclusion**

These results are consistent with BL-1020 product profile of a safe, well tolerated, and provide the opportunity to test the hypothesis that GABA-enhanced D2 blockade may offer enhanced antipsychotic activity with minimal side effects.

*This work has been supported by BioLinRx*.

# Abstract: Neural correlates of free recall in the human medial temporal lobe

**Authors:** H. Gelbard-Sagiv, R. Mukamel, M. Harel, R. Malach, I. Fried

**Affiliations:** ^1^Neurobiology Department, Weizmann Institute of Science, Rehovot, Israel; ^2^David Geffen School of Medicine, University of California Los Angeles, USA; ^3^Functional Neurosurgery Unit, Tel Aviv Medical Center, Sackler School of Medicine, Tel Aviv University, Israel

The process that leads to the moment of free recollection is still a major puzzle in human memory research. We directly investigated the involvement of the medial temporal lobe (MTL) in this process by recording from single neurons in neurosurgical epilepsy patients while they were watching short movie clips and later were freely recalling them. Patients provided written informed consent to participate in the experiment and the study conformed to the guidelines of the Medical Institutional Review Board at UCLA. Each recording session was composed of two parts. In the first part, (viewing session) patients were exposed to a sequence of short (5–10 seconds) audio-visual clips with well known content such as famous movie scenes, people, locations or events. Each clip was presented 6 times in a pseudo-random order. Interleaved blanks of 0–5 seconds were used after each clip. A typical session contained 10–16 different clips and lasted about 10min. In the second part of the experiment (free recall session), following a short (1–5 minutes) break, patients were asked to name the clips they remembered regardless of order. Performance on this task was 90.7% on average. Recordings were obtained from 332 MTL single or multiunits in 16 recording sessions with 9 patients. Of these, 30 neurons (9%) exhibited strikingly selective firing in response to a specific clip, which persists throughout clip duration as long as 10 seconds and even following clip offset. Importantly, we found that verbal reports of memories of these specific clips at the time of free recall were preceded and accompanied by highly selective firing of these same neurons. This phenomenon was manifested mainly in hippocampus and entorhinal cortex neurons and rarely in other MTL structures. These results suggest that a subset of neurons in MTL participates in both perceiving and retrieving of specific items, and hint to a possible role of these neurons in initiating the process of recollection.

*NINDS grant to I. Fried ISF to R. Malach Binational US-Isreal grant to R. Malach and I. Fried*.

# Abstract: Can the effects of putative human pheromones be attributed to chemical sensing?

**Authors:** S. Gelstein, N. Sobel, Y. Roth

**Affiliations:** ^1^Department of Neurobiology, The Weizmann Institute of Science, Rehovot 76110, Israel; ^2^Department of Otolaryngology, the Edith Wolfson Medical Center, Israel

Exposing humans to several specific steroidal compounds induces compounds induces pheromonal like responses, such as alterations in mood, hormonal state, and psychophysiology. However, it is not yet clear whether these compounds affect through chemical sensing or through alternative pharmacological routes such as transdermal diffusion. In order to address this, we set out to replicate previous experiments, but here include a group of anosmic (without a sense of smell) women. We hypothesized that if the effect is through transduction in the main olfactory system, then these women should not show the effects. The putative pheromone 4, 16- Androstadiene (AND) or placebo (yeast) were sniffed 10 times at the beginning of a ∼2 hour recording session where we measured psychophysiological parameters (skin conductance, electrocardiogram, ear pulse, finger pulse, abdominal respiration, thoracic respiration, skin temperature, and body movement), mood (17 emotional descriptors), and salivary hormones (testosterone and cortisol), while subjects viewed mood-inducing video clips. To date, 3 subjects have been studied. Consistent with previous reports, this experimental arrangement modulated mood states and physiological arousal in the intended manner in all 3 subjects. Data from an intended cohort of 30 subjects will be presented.

# Abstract: The antidepressant effect of repeated subconvulsive electrical stimulation of reward related brain sites in a rat model for depressive behavior: a comparison to the effect of electroconvulsive therapy

**Authors:** R. Gersner, E. Toth, A. Zangen

**Affiliations:** Weizmann Institute of Science, Israel

Electroconvulsive therapy (ECT) is the most effective treatment formajor depression. Our aim was to test whether subconvulsive electrical stimulation (SCES) of specific rewardrelated brain regions such as the prelimbic cortex (PLC) or nucleus accumbens (NAC) can induce an antidepressant effect in a widely used rat model for depressive behavior. We have utilized a battery of behavioral tests including sucrose preference, forced swimming test, home-cage locomotion, exploration of a novel environment and the Morris water maze. Also, we measured the levels of brain-derived neurotrophic factor (BDNF). We found that repeated SCES treatment of either the NAC or the ventral (but not the dorsal) PLC reversed the main behavioral deficit and the reduction of BDNF levels in the dorsal hippocampus induced by CMS, much like ECT did, but without causing cognitive side effects. These results suggest that SCES treatment of ventral PLC and NAC can induce an antidepressant effect similar to ECT, but without causing a cognitive deficit, implicating these areas in the pathophysiology of depression.

# Abstract: Stochastic synaptic transmission is advantageous for learning complex temporal firing patterns in neurons

**Authors:** A. Gidon, M. London, I. Segev

**Affiliations:** ^1^Department of Neurobiology, Institute of Life Sciences, the Hebrew University of Jerusalem, Israel; ^2^Wolfson Institute for Biomedical Research, University College London, WC1E 6BT London, UK; ^3^Interdisciplinary Center for Neural Computation, the Hebrew University of Jerusalem, Israel

Classical supervised learning algorithms (e.g., the Perceptron learning rule) implicitly use the contribution of an input synapse to the firing output by changing the synaptic weight in proportion to its correlation with the output. In the biological context, however, the contribution of a specific input to the final neuronal output might be difficult to calculate and, in particular, it might not be available as an explicit parameter for plasticity. Here we propose a novel idea for the role of the inherent probabilistic nature of synaptic release (the occasional failures of vesicle secretion at the pre-synaptic terminal) for learning. We present an algorithm that utilizes synaptic stochasticity in order to sample the input space and to implicitly estimate the contribution of each synapse for the “success” of the desired target output. The algorithm is based on the positive correlation between the contribution of a synapse and the “achievement” in attaining the desired output of randomly sampled input subgroups in which it participates (i.e., positively contributing synapses are more abundant in “successful” input subgroups and vise versa for “unsuccessful” input subgroups) . We further examine the performance of this learning rule in terms of memory capacity and convergence time and in terms of its robustness to a high variability in the synaptic weights. Finally we demonstrate how this abstract idea can simplify a complex learning task of mapping temporal input pattern to an arbitrary output pattern in a realistic model neuron.

# Abstract: Rho/ROCK signalling down-regulatesmyelin phagocytosis in microglia

**Authors:** M. Gitik, F. Reichert, S. Rotshenker

**Affiliations:** Department of Anatomy and Cell Biology, Faculty of Medicine, Hebrew University, Israel

Microglia play a critical role in the innate immune response of the CNS to injury and disease by removing degenerating myelin through phagocytosis. Phagocytosis of C3biopsonized myelin is mediated by complement receptor-3 (CR3) and phagocytosis of nonopsonized myelin by CR3 and scavenger receptor-A (SRA). Small GTPases of the Rho family play a central role in motile responses that involve actin and/or microtubule networks, from neurite extension to phagocytosis. Among these, Rho, Rac and CdC42 regulate actin. C3 transferase toxin (Clostridium botulinum) that selectively inhibits Rho, enables studying rho-dependent signalling. Rho activates ROCK (Rho kinase; serine/threonine protein kinase) that phosphorylatesmyosin light chain phosphatase, increasing thereby myosin II activity and the formation of stress-fibers (bundles of F-actin and myosin). In the present study we examined the role of Rho/ROCK signalling on myelin phagocytosis and stress-fiber formation in microglia. Rho-inhibitor C3 augmented phagocytosis of C3biopsonized and nonopsonized myelin in microglia that express CR3 and SRA combined and separately, suggesting that Rho down-regulates CR3 and SRA mediated myelin phagocytosis. Phagocytosis of C3bi-opsonized and nonopsonized myelin was further augmented by ROCK-inhibitor Y-27632, suggesting that ROCK also downregulates myelin phagocytosis. F-actin, visualized by phalloidin staining, formed a network of stress-fibers throughout the cytoplasm of normal microglia, and was further present at the perimeter of cells. F-actin fibers became disassembled in ROCK inhibited microglia that further displayed many more filopodia than normal, suggesting that Rho/ROCK signalling upregulates stress-fibers and downregulates filopodia. Notably, filopodia engulf myelin in the course of phagocytosis. Thus Rho/ROCK may downregulate myelin phagocytosis by upregulating stress-fibers and downregulating filopodia.

*Supported by Israel Science Foundation 11/06*.

# Abstract: Sensory-motor processes in the understanding of spoken words: an fMRI study

**Authors:** I. Glick, U. Hadar

**Affiliations:** ^1^Department of Psychology, Tel Aviv University, Israel; ^2^Wohl Institute for Advanced Imaging, Tel Aviv Sourasky Medical Center, Israel

Recent neuropsychological and neuroimaging evidence suggests that the cerebral language system may be described as a distributed functional network, rather than the traditional view of two localized centers. Within this network, the classical language sites (Broca's andWernicke's areas) may process such word attributes as morphology, phonology and syntax, whereas the network of sensory-motor properties may process specific semantic information. This sensory-motor semantic network may engage various nonlinguistic areas that are close to the related sensory or motor processors. Using fMRI, we identified two separate centers in the sensorymotor network that process two aspects of word semantics, one related to motor and one to somatosensory properties of words. Twelve right-handed, native Hebrew speakers participated in this study. Stimuli words were divided into different categories according to their semantic properties: motor, somatosensory and control (animal names). Words were presented in category blocks and, for each word, subjects performed a semantic decision (is the word pleasant or unpleasant?) or a phonological decision (does the word have two syllables). Compared to control, the semantic processing of motor words yielded activation in left MTG and bilaterally in the fusiform gyrus. Previous studies (Chao 1999; Price 2003) found that these areas are activated when contrasting the naming of tools with the naming of animals. Additionally, the semantic processing of somatosensory words, compared to control, yielded activation in left inferior parietal lobule (ba40), an area which is part of the secondary somatosensory cortex (Eickhof 2006). By contrast, the phonological processing of the stimuli did not exhibit differential activations according to the semantic properties of the words. We concluded that the processing of motor and somatosensory semantic properties of words are subserved by distinct cortical sites.

*This study was supported by the Levie-Edersheim-Gitter Institute For Functional Brain Imaging*.

# Abstract: Altered cholinergic gene expression in pilocarpine-treated mice

**Authors:** Y. Gnatek, N. Najami, Y. Bitan, A. Friedman

**Affiliations:** Departments of Physiology and Neurosurgery, Soroka Medical Center, Ben Gurion University, Israel

Temporal lobe epilepsy (TLE) is the most common form of partial epilepsy and is associated with significant morbidity. TLE patients often show hippocampus histopathology characterized by cell loss in the CA3 and CA1 subfields and in the dentate hilus. In experimental animals injection of the muscarinic agonist, pilocarpine, induces status epilepticus (SE), followed 2-3 weeks later by the appearance of spontaneous seizures and hippocampal damage similar to that observed in human TLE patients. Temporal lobe structures, including the hippocampus, are enriched with cholinergic innervation and dysfunction of the cholinergic system is well known in several neurological disorders. However, it is not known what role cholinergic dysfunctionmay have in TLE. The aim of the present study was to explore changes in cholinergic transmission and their role in the pathogenesis of TLE. To this end we have established the pilocarpine model in mice and searched for changes in expression of key cholinergic genes during epileptogenesis. 97 FVB/N mice were injected with pilocarpine (280–340 mg/Kg). Continuous video recordings made in 11 mice showed high rate of spontaneous seizures (82%) 4–8 weeks after SE. RT-PCR demonstrated increased mRNA levels in both the synaptic (S) and readthrough (R) alternatively spliced transcripts of the ACHE gene. ACHE upregulation was noticed already at 48 hours following SE and correlated with an increase in the enzyme activity as was shown in activity gel and Karnovski staining. Increased expression lasted for two weeks and were found to be significantly increased in both the hippocampus and temporal cortex. In situ hybridizations confirmed the expression of ACHE in all regions of the hippocampus, and immunostainings confirmed its expression in principal pyramidal neurons, GABAergic interneurons, but only rarely in astrocytes. The upregulation of ACHE during the latent period of epileptogenesis suggest a role for cholinergic dysfunction in epileptogenesis.

# Abstract: Impaired migration signaling in the hippocampus following prenatal hypoxia

**Authors:** H. M. Golan, V. Kaplan-Louzone, M. Zuckerman, R. J. Perez-Polo

**Affiliations:** ^1^DevelopmentalMolecular Genetics, and Zlotowski ctr for Neuroscience, BGU, Israel; ^2^Biochemistry and Molecular Biology, UTMB, Galveston, Tex, USA

Prenatal hypoxia ischemia is a major cause of neurodevelopmental impairment in the newborn, associated with risk for motor, behavioral and cognitive impaired outcomes. We have previously shown, in a mousemodel, thatmaternal pretreatment with magnesium sulfate (Mg) partially prevented the behavioral consequences of maternal hypoxia in the adult offspring. We now used this mouse model of maternal hypoxia to examine the immediate molecular responses of signaling pathways associated with both cell death and neurogenesis. We also characterized responses to maternal pretreatment with MgSO^4^. Maternal hypoxia at embryonic day 17 (E17) failed to trigger inflammation or cell death in fetal brain at 24 hours after hypoxia. However, maternal hypoxia decreased in the hippocampus levels of neuronal migration signaling: Reelin (53 % of control, *P* < .05), Disable 1 (Dab1, 77% of control, *P* < .01), and amyloid precursor protein (APP, 64% of control, *P* < .01) 2 hours after the insult. These changes persisted for 24 hours. At later times, Reelin levels in hippocampi of newborns in the maternal hypoxia treated group increased compared to controls (14% and 51% of control, 5 and 20 days after the insult, respectively, *P* < .05). Full protection from maternal hypoxia effects on hippocampal Reelin levels resulted from maternal pretreatment with MgSO^4^. In the newborn cortex, there was a higher sensitivity toMgSO^4^ treatment. Taken together, the long-termneurodevelopmental outcome of prenatal and perinatal hypoxia may depend on perturbation of developmental signals that affect neuronal migration.

*Supported by BSF Grant no. 2003166*.

# Abstract: The transcription factor Nato3 is implied as a novel regulator of floor plate and spinal cord development

**Authors:** T. Golan-Lev, E. Nissim-Eliraz, A. Mansour, S. Zisman, O. Schatz, A. Klar, N. Ben-Arie

**Affiliations:** ^1^Cell and Animal Biology, Institute of Life Sciences, The Hebrew University of Jerusalem, Israel; ^2^Anatomy and Cell Biology, Hadassah Medical School, The Hebrew University of Jerusalem, Israel

The floor plate is one of the key organizers of the central nervous system in vertebrates and plays important roles in both ventral patterning and axonal guidance in the neural The floor plate is one of the key organizers of the central nervous system in vertebrates and plays important roles in both ventral patterning and axonal guidance in the neural tube. The floor plate is induced at the ventral midline of the neural tube through the action of Sonic hedgehog (Shh), which in turn activates the transcription of target genes. Nato3 is a member of the basic Helix-Loop-Helix (bHLH) family of transcription factors, which regulate the specification/ differentiation of specific cell lineages during neurogenesis. We found that within the developing spinal cord MNato3 and CNato3 expression is restricted to the floor plate in mouse and chick, respectively. However, the signaling pathway through which MNato3 acts is currently unknown. Here, we aimed at identifying cis control elements and molecular mechanisms that target MNato3 expression to the floor plate. To identify the DNA regions that direct MNato3 expression to the floor plate we applied a reporter gene assay in chick embryos using in ovo electroporation. Our results reveal a short genomic DNA region upstream of MNato3, which is necessary and sufficient to derive mouse Nato3 expression in the chick floor plate. We next looked for evolutionarily conserved transcription factor binding sites within this region, which may regulate the expression of MNato3. In vitro studies confirmed that the putative conserved binding sites identified in vivo and in silico are indeed functional, and relate MNato3 to the Shh signaling pathway. Our results provide the first linkage between MNato3 and the signaling pathway these factors regulate, and suggest that MNato3 may have an important role in spinal cord development.

# Abstract: A specific processing of afferent theta inputs via synaptic kainate receptors in O-LM neurons

**Authors:** M. Goldin, J. Epsztein, I. Jorquera, A. Represa, Y. Ben-Ari, V. Crèpel, R. Cossart

**Affiliations:** Institut de Neurobiologie de la Mèditeranèe, Marseille, France

GABAergic interneurons are a diverse population with an important role in network function. Distinct GABAergic circuits were shown to differentially process information dynamics ([1–4]). This integrative specificity of GABAergic circuits was partly attributed to the kinetics of their glutamatergic afferents ([5–7]). Different glutamate receptor subtypes can mediate synaptic currents with specific temporal properties. In particular, kainate receptors (KA-R) were shown to be preferentially expressed in CA1 stratum oriens interneurons ([8]) but little is known about their functional distribution among cell types. Combining multibeam twophoton imaging of network activity in the CA1 region from somatostatine(SOM)-GFP mice with targeted electrophysiological recordings and morphological analysis, we show that SOM-EGFP+ interneurons are preferentially recruited by synaptic stimulations at theta frequency and that KA-Rs selectively participate in this activation in O-LM cells. Such preferential processing of excitatory inputs via KA-Rs by distally projecting GABAergic microcircuits may explain their pivotal role in theta band frequency generation.

*Supported by Marie Curie grant*.

## References

[1] Pouille F, Scanziani M (2004). Routing of spike series by dynamic circuits in the hippocampus. *Nature*.

[2] Klausberger T, Magill PJ, Márton LF (2003). Brain-state- and cell-type-specific firing of hippocampal interneurons in vivo. *Nature*.

[3] Klausberger T, Márton LF, Baude A, Roberts JDB, Magill PJ, Somogyi P (2004). Spike timing of dendrite-targeting bistratified cells during hippocampal network oscillations in vivo. *Nature Neuroscience*.

[4] Miles R, Tóth K, Gulyás AI, Hájos N, Freund TF (1996). Differences between somatic and dendritic inhibition in the hippocampus. *Neuron*.

[5] Fuchs EC, Doheny H, Faulkner H (2001). Genetically altered AMPA-type glutamate receptor kinetics in interneurons disrupt long-range synchrony of gamma oscillation. *Proceedings of the National Academy of Sciences of the United States of America*.

[6] Pouille F, Scanziani M (2004). Routing of spike series by dynamic circuits in the hippocampus. *Nature*.

[7] Traub RD (1993). *The Journal of Physiology*.

[8] Cossart R, Epsztein J, Tyzio R (2002). Quantal release of glutamate generates pure kainate and mixed AMPA/Kainate EPSCs in hippocampal neurons. *Neuron*.

# Abstract: Influence on offspring behavior of stress to the rat dam before she becomes pregnant: effects of cross-fostering

**Authors:** G. Gonen, N. Ferdman, B. Even, C. Maroun, O. Katz, L. Prshin, J. Schulkin, M. Leshem

**Affiliations:** ^1^Department of Psychology, University of Haifa, Israel; ^2^Department of Physiology and Biophysics and Neuroscience, Georgetown University, DC, USA

Pregestational adversity impacts offspring affective behavior (Shachar-Dadon et al., 2006). To discover whether preconception stressmight exert its effects by altering the dam'smaternal behavior, in turn influencing the development of offspring behavior, we investigated the effects of cross-fostering offspring of preconception stressed dams to control dams and vice versa. Virgin rats were undisturbed (controls), or exposed to varied, unpredictable, stressors for 7 days, ending 1 week prior to mating. Pregestational stress did not affect conception rates, duration of pregnancy, litter size, or gender ratio of offspring, but offspring of stressed dams were marginally lighter at birth as well as at 2 months, whereas fostering increased body weight. Two hundred and eighty eight offspring in 6 experimental groups were tested in a variety of tasks to evaluate affective and social behavior. There were complex interactions due to influence of preconception stress, gender, and fostering, which suggested that preconception stressed mothers contribute partially to the alterations in offspring behavior. However, some of the variance was also explained by offspring differences—that is, even if raised by control mothers, offspring of preconception stressed dams showed behavioral changes.

*Supported by the BSF to ML and JS*.

# Abstract: Stable firing patterns in neural networks: a three-level model of consciousness

**Authors:** G. Gordon

**Affiliations:** Department of Chemical Physics, Weizmann Institute of Science, Rehovot 76100, Israel

A novel, comprehensive theory of the neural correlates of consciousness is presented. It states that two types of neural networks, namely feedforward networks and Hopfield-like feedback loops, compose three levels of consciousness: a low, unconscious processing level, amiddle, conscious perception level, and a high level, integrating consciousness. We claim that only stable firing patterns of complexHopfield-like feedback neural networks give rise to conscious perception. Thus, low-level feedforward networks of sensory processing do not involve conscious perception, whereas the high-level feedback loops of many middle-level areas give rise to one, cohesive consciousness. Examples from visual and auditory systems are presented, as well as a thorough discussion on the role of attention and subcortical areas within this framework. Several new conjectures arise from this theory and new experimental designs are suggested.

# Abstract: Does abnormal binocular experience during critical period limit normal development of visual cognitive functions?

**Authors:** D. Gotthilf-Nezri, S. Gilaie-Dotan, A. Perry, Y. Bonneh, S. Bentin, R. Malach, U. Polat

**Affiliations:** ^1^Tel Aviv University, Sackler faculty of medicine, Goldschleger Eye Research Institute,Tel Hashomer, Israel; ^2^Department of Neurobiology, Weizmann Institute of Science, Rehovot, Israel; ^3^Department of Psychology, The Hebrew University of Jerusalem, Jerusalem, Israel; ^4 ^Center for Neural Computation, The Hebrew University of Jerusalem, Jerusalem, Israel

Amblyopia is a reduction of best-corrected visual acuity caused by abnormal visual experience during the “critical period,” which prevents normal development of the visual perceptual functions, usually in one eye. The cognitive functions are not affected, probably due to normal visual input from the other eye. Does abnormal binocular visual experience limit the development of normal visual cognition? Here we report the case of LG, a 20-year-old male, with reduced visual acuity in both eyes, which does not result from a known optical deficiency and could not be corrected by spectacles. He has been diagnosed as congenital associative visual agnosia with severely impaired face identification; he does not recognize very familiar faces including his own and his parents', and has dificulies identifying distant objects. He has no other neurological disorders, he can read, his IQ is above average, and he functions normally in any other aspect. His early and high-level visual areas displayed robust fMRI activation by visual stimuli, whereas intermediate visual areas displayed profound ihibition during visual processing. We employed a treatment of amblyopia tailored to LG, training him twice a week, monocularly and binicularly. After sixmonths of training, a remakable improvement in all visual parameters was noted: Distance VA (LogMar) from 0.66 to 0.4 and 0.5 to 0.46 (R, L), near VA from 0.48 to 0.36 and 0.4 to 0.32, Lateral interactions, from 0.08 (suppression, log units) to −0.35 (facilitation) and from −0.1 to −0.36. The crowding effect was reduced from 0.233 to 0.06 and from 0.17 to 0.02. Stereopsis improved from 30 to 20 arcsec. Very importantly, we noticed a subjective improvement in his processing time for recognition and the ability to identify objects and shapes requiring global integration. Thus, improvement of the visual functions may be transferred to improvement—perhaps on a slower time scale—of cognitive functions and brain areas that were impaired.

*Supported by a grant from the Israel Science Foundation*.

# Abstract: NAP reduces TAU hyperphosphorylation and enhances learning in a novel transgenic mousemodel

**Authors:** I. Gozes, A. Pinhasov, S. Mandel, R. Pikman, N. Grigoriadis, O. Touloumi, Z. Pittel, I. Vulih-Shultzman

**Affiliations:** ^1^HumanMolecular Genetics and Biochemistry, Sackler Medical School, Tel Aviv University, Israel; ^2^Aristotle University of Thessaloniki, Stilp. Kyriakidi str 1, GR-54636 Thessaloniki, Greece

Complete deficiency of activity-dependent neuroprotective protein (ADNP) is embryonic lethal, hence, the outcome of partial ADNP deficiency was examined. ADNP+/− mice exhibited cognitive deficits, significant increases in phosphorylated tau, tangle-like structures and neurodegeneration as compared to ADNP+/+ mice. Increased tau hyperphosphorylation is known to cause memory impairments in neurodegenerative diseases associated with tauopathies, including the most prevalent Alzheimer's disease. The current study suggested that ADNP is an essential protein for brain function and plays a role in normal cognitive performance and social interactions. ADNP-deficient mice offer an ideal paradigm for evaluation of cognitive enhancers. NAP (NAPVSIPQ) is a peptide derived from ADNP that interacts with microtubules and provides potent neuroprotection. NAP treatment partially ameliorated cognitive deficits and reduced tau hyperphosphorylation in the ADNP+/− mice ([1]). NAP is currently being tested by Allon Therapeutics Inc. (www.allontherapeutics.com) in a phase II program in Alzheimer's disease.

*Supported by BSF, ISF, Lily, and Avraham Gildor Chair, Adams Super Center and Allon Therapeutics Inc*.

## References

[1] (2007). *Journal of Pharmacology and Experimental Therapeutics*.

# Abstract: Movement parameters are related to inter-trial variability of single units activity in motor cortex

**Authors:** Y. Grinvald, D. Inbar, N. Zach, E. Vaadia

**Affiliations:** ^1^Physiology Department, Hadassah Medical School, Hebrew University, Jerusalem, Israel; ^2^Interdisciplinary Center for Neural Computation (ICNC), Hebrew University, Jerusalem, Israel

Common analysis of neuronal data involves averaging of activity across many trials or events ignoring possible effects of trial-by-trial variability. The objective of this study is to examine representations of behavioral parameters with single trial resolution. We simultaneously recorded, single unit activities using 32 independently moveable microelectrodes from primary motor (M1) and pre-motor areas of the monkey during performance of “center-out” reaching movements and during visuomotor learning. We computed the relations between firing rate of single cells in single trials to several behavioral parameters (reaction time, movement time, peak velocity, trajectory curvature and others). We found that firing rate variability of single cells could be significantly correlated with behavioral events, and in many cases with more than one behavioral parameter. The correlation was highest during movements towards the cell's preferred direction. Interestingly, the degree of correlation could change at different time windows and was not restricted to the time of increased mean firing rate. Further, the trial-by-trial analysis revealed relations that could not be observed in the averaged evoked response of the cell. These findings show a useful tool for observing relations between the activities of single cells at the level of single trials and may explain variability in behavioral performance. These correlates may improve inference of behavior on the basis of neural activity.

*Supported by BSF, J and J Fund for Innovative Science, FIF by Rosetrees Trust, Abraham and Ida Baruch Foundation*.

# Abstract: Integrated contextual representation for objects' identities and their locations

**Authors:** N. Gronau, M. Bar

**Affiliations:** ^1^The open university of Israel, Raanana, Israel; ^2^Martinos Center for Biomedical Imaging atMGH, Harvard Medical School, Mass, USA

Visual context plays a prominent role in every day perception. Contextual information can facilitate recognition of objects within scenes by providing predictions about objects that are most likely to appear in a specific setting, along with the locations that are most likely to contain objects in the scene. Is such identity-related (semantic) and locationrelated (spatial) contextual knowledge represented separately or jointly as a bound representation? We conducted an fMRI priming experiment whereby semantic and spatial relations between prime and target object pictures were independently manipulated. This method allowed us to determine whether the two contextual factors affect object recognition with or without interacting, supporting a unified versus independent representations, respectively. Results revealed a semantic by spatial interaction in reaction times for target object recognition. Namely, significant semantic priming was obtained when targets were positioned in expected (congruent), but not in unexpected (incongruent) locations. fMRI results showed corresponding interactive effects in brain regions associated with semantic processing (inferior prefrontal cortex), visual contextual processing (parahippocampal cortex), and object-related processing (lateral occipital complex). In addition, activation in frontoparietal areas suggests that attention and memory-related processes might also contribute to the contextual effects observed. These findings indicate that object recognition benefits from associative representations that integrate information about objects' identities and their locations, and directly modulate activation in objectprocessing cortical regions. Such contextual schemas are useful in maintaining a coherent and meaningful representation of the visual world, and in providing a platform from which predictions can be generated to facilitate perception and action.

# Abstract: RHO/ROCK signaling negatively modulates delimination of neural crest cells downstream of BMP and G1/S transition

**Authors:** M. Groysman, C. Kalcheim

**Affiliations:** Department of Anatomy and Cell Biology, Hebrew University of Jerusalem-Hadassah Medical School Jerusalem, Israel

During neural crest (NC) ontogeny, an epithelial to mesenchymal transition is necessary for cell emigration from the dorsal neural tube. This process is likely to involve a network of gene activites which remain largely unexplored. We demonstrate that Rho signaling, via Rock, inhibits the onset of NC delamination. In vivo loss-of-function of RhoA or RhoB or of general Rho signaling by C3 transferase enhanced NC delamination. Consistently, treatment of neural primordia with C3 or with the Rock inhibitor Y27632 both accelerated and enhanced NC emigration. Reciprocally, Rho activation by LPA inhibited the process. Next, we analyzed the responsible mechanisms. Inhibition of Rho/Rock profoundly affected the morphology of NC cells by disrupting actin stress fibers and vinculin focal adhesions. Moreover, it stimulated a premature downregulation of N-cadherin and of cell-cell adhesions. Reciprocally, retention of stress fibers, focal adhesions and N-cadherin were monitored upon treatment with LPA. Possible interactions between Rho and the BMP-dependent pathway leading to NC delamination were addressed. Blocking Rho or Rock function rescued NC delamination in explants treated with noggin or with the cell-cycle inhibitor mimosine. In the latter case, NC cells emigrated while arrested at G1. Hence, Rho signaling negatively regulates NC delamination through modification of cytoskeleton assembly and cellcell adhesions by acting downstream of BMP and of G1/S transition.

# Abstract: The restless mind—a relation between rest and the self in the brain—a deep-TMS study-presentation of preliminary results

**Authors:** M. Gruberger, T. Hendler, A. Zangen, Y. Levkovitz

**Affiliations:** ^1^Department of Psychology, Tel-Aviv University, Israel; ^2^The Emotion-Cognition Research Center, The Shalvata Mental Health Center, Hod-Hasharon, Israel; ^3^Functional Brain Center, Tel-Aviv Sourasky Medeical Center, Tel-Aviv, Israel; ^4^Sackler Faculty of Medicie, Tel-Aviv University, Israel; ^5^Department of Neurobiology, TheWeizmann Institute of Science, Rehovot, Israel

The human tendency to think spontaneously in the absence of cognitive load is one aspect of the “self ” challenging neuroscience research. This tendency was suggested to be related to the “default-mode” brain network, which shows increased activity at rest. Support for this notion stems from studies indicating that the medial prefrontal cortex (MPFC), a main component of this network, is associated with selfrelated functioning. The current study examined a direct manipulation of MPFC, an important addition to prior research which used only correlative imaging techniques. The manipulation of MPFC in conscious participants was performed by means of deep transcranial magnetic stimulation (dTMS) administered with the H-coil, a novel TMS coil for deep brain stimulation. The effect of a “temporary lesion” in the MPFC was assessed using inhibitory dTMS, followed by a 2-minute “rest” period. This “lesion” was expected to attenuate “default-mode” activity during rest, resulting in an altered sense of “self ” and induced sense of dissociation. Forty subjects are planned to participate in this study, which will include real versus sham dTMS conditions and “rest” versus “no-rest” conditions. Here we present preliminary results obtained from the first ten participants, five in the “dTMS + rest” condition and five in the “dTMS + no-rest” condition. Senses of self and of dissociation were examined using situational self-awareness questionnaires and a situational sense of dissociation questionnaire. Results show that whereas subjects in the “dTMS + no-rest” condition did not experience a noticeable change, subjects in the “dTMS + rest” experienced a dissociative sensation, accompanied by alterations in the level of self-awareness. Subjects' free reporting support this data, and include describing a temporary feeling of detachment and numbness. These preliminary results support the notion that MPFC activity during rest might be significant for an integrative, on-going sense of self in humans.

*This study was supported by the Adams Super-Center for Brain* *Studies, Tel-Aviv University, Israel.*

# Abstract: The conductance-based tempotron: amodel for time warp invariant auditory processing

**Authors:** R. Guetig, H. Sompolinsky

**Affiliations:** ^1^Racah Institute of Physics, Israel; ^2^Interdisciplinary Center for Neural Computation, Hebrew University, Israel; ^3^Center for Brain Science, Harvard University, Mass, USA

In many natural tasks auditory systems must satisfy two seemingly conflicting requirements: on one hand, information processing often relies on exquisite sensitivity to fine temporal features of the incoming signal. On the other hand, the overall time scale of these input signals can be subject to substantial variability. For instance in speech, variations in speaking rate can readily introduce a time warp ranging from two fold compression to two fold dilation. How does the neural system overcome this challenge? To address this question we study a neural model of spike-based learning of an auditory discrimination task, extending our recent tempotron model. In our present model, synaptic inputs are modeled as conductances rather than current pulses. In the conductance-based tempotron the time scale of the voltage dynamics strongly depends on the total synaptic conductance rather than being a fixed parameter. As a result, the statistics of inputs as well as the synaptic learning rule, interact with the time scale of neural information processing by changing the effective integration time constant of the cell. Importantly, since the effective membrane time constant of the neuron scales inversely with its total conductance, the neuron exhibits a high degree of time warp invariance. We have applied our model to speech recognition problems. Using a simple model of the auditory periphery, sound signals are converted into spike patterns by thresholding their power-spectral densities, and fed into a small population of conductance-based Integrate and Fire neurons. Surprisingly, using our tempotron learning rule, the system achieved nearly perfect word recognition accuracy on a standardized digit recognition task. Our results show that simple biologically plausible mechanisms of spatio-temporal integration and synaptic plasticity, can endow the neural system with powerful time warp invariant auditory processing capabilities.

*Supported by Minerva Foundation, ISF (Center of Excellence no. 8006/00), MAFAT*.

# Abstract: Visual-auditory integration in the barn owl: a neuroethological approach

**Authors:** Y. Gutfreund, A. Reches, Y. Zachar

**Affiliations:** Department of Physiology, The Faculty of Medicine, The Technion, Israel

Research on multisensory integration has expanded rapidly in recent years attracting scientists from a variety of fields including psychophysics, MRI, robotics and single cell studies. In my presentation I will highlight the potential of the barn owl as a unique animal model for research on visual auditory integration. Barn owls are highly efficient nocturnal predators that have evolved precise and sensitive visual and auditory systems. Barn owls operate in noisy and dim lighted environments, environments where the benefit of merging information from different modalities is substantial. Thus, the ability to integrate visual and auditory signals may be critical to the survival of this species. I will describe recent results from my lab on the properties of multisensory neurons in the forebrain and midbrain of the barn owl and will compare these findings with results obtained from cats (an animal model commonly used for the study of visual-auditory integration).

*This work was supported by* *an ISF BIKURA grant.*

# Abstract: Where aremy arms? An octopus visually controlling its arm movements

**Authors:** T. Gutnick, R. A. Byrne, M. J. Kuba, B. Hochner

**Affiliations:** Department of Neurobiology, Institute of Life Sciences and Interdisciplinary Center for Neural, Israel

Octopuses depend on the high flexibility of their arms to carry their arms to carry out a large variety of task like hunting, swimming and mating. Thus far only the motor primitive reaching for a target movement has been shown to be visually guided. Other movements that do utilize the high flexibility of the arms, like those used for searching and probing, have been hypothesized to be largely autonomous and not directly controlled by the central nervous system. We designed a behavioral task allowing us to define a relationship between the CNS and the peripheral nervous system that enables directed arm movement. Eye-arm coordination was tested using a transparent Plexiglas maze. 9 adult Octopus vulgaris were trained to insert a single arm through a tube for a food reward. Initially, food was presented inside a tube for several days, and animals learned to reach in to collect a food reward. Next, the transparent three armed maze was introduced fully baited. These introductory phases accustomed the subjects to reach through a tube, out of the water and into a goal box in order to retrieve food. In the experimental phase a food reward was placed in only one of the three compartments that was visually cued, octopuses were required to guide their arms towards the rewarded compartment. Criteria for successful learning were the completion of 5 correct trials in a row. 6 of the 9 animals completed the experiment, reaching criterion within 22 to 208 trials. After criterion was reached 20 control trials were preformed using an opaque maze. During control trials the animals' performances fell back to chance level. This study is the first behavioral test showing that octopuses can learn to visually control arm movements that are not restricted to the control of few degrees of freedom (like reaching). Our findings suggest that octopuses are capable of central control of arm movements and perhaps cross reference between different sensory inputs and motor outputs.

# Abstract: IMPA1 and lithium-like behavior

**Authors:** I. Hadas, G. Agam, R. Belmaker, Y. Bersudsky

**Affiliations:** Stanley Research Center, Faculty of Health Sciences, Ben Gurion University and Mental Health Center, Israel

Lithium has been the standard pharmacological treatment for bipolar disorder over the last 50 years, however the molecular targets through which lithium exerts its therapeutic effects are still not defined. Inhibition of the activity of the enzyme inositolmonophosphatase by therapeutically relevant lithium concentrations raised the possibility that this enzyme is the primary target of this drug. Mammalian inositol monophosphatase is coded by two genes, IMPA1 and IMPA2. IMPase activity levels were found to be reduced (up to 65% in hippocampus) however inositol levels were not found to be altered and animals survived during two weeks on inositol deficiency food. We characterized the behavioral phenotype of mice with a knockout IMPA1 gene (IMPA1−/− ) to study the in vivo physiological functions of IMPA1, in general, and more specifically its potential role as a molecular target in mediating lithium-dependent physiological effects. In brains of adult IMPA1−/− mice Behavioral analysis of the IMPA1−/− mice indicated an increased motor activity in the open-field test, the forced-swim test and the tail suspension test. In conclusion the IMPA1−/− mouse indicates that genetic inactivation of IMPA1 can mimic some actions of lithium.

# Abstract: Transcriptional control of commissural neuron cell fate

**Authors:** Y. Hadas, N. Shkolnik, A. Klar

**Affiliations:** Department of Anatomy and Cell Biology, Hebrew University, Hadassah Medical School, Jerusalem, Israel

The dI1, dI2, and dI3 classes of interneurons originate at the dorsal spinal cord, from a group of progenitors defined by the expression of bHLH transcription factors: Math1, Ngn1+Ngn2, and Mash1, respectively. The expression of the bHLH transcription factors is followed by the expression of Lim-HD transcription factors Lhx2 + Lhx9, Lim1 and Isl1, respectively. In all postmitotic dI neurons sequential activation of other transcription factors leads to the expression of receptors for floor plate derived guidance molecules (DCC, Rig1, TAG-1) which enable the commissural interneuron to reach the floor plate. Ectopic expression in the chick neural tube was used in order to investigate the role of different transcription factors in the determination of dI neurons and the segregation of dI neurons to different dI subclass. We found that a bHLH gene, NSCL1 transiently induce expression of the commissural neuron receptors TAG1 and Rig1 but does not induce the expression of Lim-HD proteins. Ectopic expression also demonstrated corepression between the lim-homeodomain proteins: Lhx9 inhibits the expression of Lim1 and Isl1, Lim1 inhibits the expression of Lhx2/9 and Isl1 inhibits the expression of Lhx2\9 and Lim1. Thus, two parallel differentiation pathways may regulate commissural interneuron fate. The initial commissural axonal trajectory might be governed by the activation of TAG1/Rig-1/DCC expression by the bHLH proteinNSCL1. Subsequently, subtype specification is determined by the expression of Lim-HD proteins.

# Abstract: Tyrosine kinase Syk regulates differentiallymyelin and pathogen phagocytosismediated by complement receptor-3, dectin-1, and mannose receptor

**Authors:** S. Hadas, F. Reichert, S. Rotshenker

**Affiliations:** Department of Anatomy and Cell Biology, Faculty of Medicine, Hebrew University, Israel

Microglia play a critical role in the innate immune response of the CNS to injury and disease by removing, through phagocytosis, tissue debris (degenerating myelin in injury and demyelinating diseases) and pathogens (yeast). Phagocytosis of C3bi-opsonized myelin is mediated by Complement Receptor-3 (CR3) and phagocytosis of nonopsonized myelin by CR3 and Scavenger Receptor-A (SRA). Phagocytosis of C3bi-opsonized Zymosan (yeast derived particle) is mediated by CR3, and phagocytosis of nonopsonized Zymosan by Dectin-1 (beta-glucan receptor) and mannose receptor (MR; alpha-mannan receptor). The protein tyrosine kinase spleen tyrosine kinase (Syk) is critical for immune responses. In the present study, we tested the role of Syk in regulating phagocytosis of myelin and Zymosan in microglia. Syk inhibitors, Syk-Inhibitor and R406, augmented phagocytosis of C3bi-opsonized and nonopsonizedmyelin in microglia expressing CR3 and SRA combined or separately. Syk thus downregulates phagocytosis of C3bi-opsonized and nonopsonized myelin by CR3 and SRA. Syk-Inhibitor did not modulate phagocytosis of C3bi-opsonized Zymosan but inhibited phagocytosis of nonopsonized Zymosan. Syk thus upregulates Dectin-1 and/or MR mediated phagocytosis of nonopsonized Zymosan and down-regulates CR3 mediated phagocytosis of C3bi-opsonized Zymosan. Syk-Inhibitor inhibited phagocytosis of C3bi-opsonized and nonopsonized Zymosan in microglia lacking CR3, thus no CR3 mediated phagocytosis, supporting the suggestion that Syk upregulates Dectin-1 and/or MR mediated phagocytosis of nonopsonized Zymosan. Syk-Inhibitor augmented phagocytosis of C3bi-opsonized Zymosan in HL60 cell line that express CR3 but neither Dectin-1 nor MR, thus CR3 mediated phagocytosis only, supporting the suggestion that Syk downregulates CR3 mediated phagocytosis of nonopsonized Zymosan. Thus altogether, Syk downregulates CR3 and SRA mediated phagocytosis and upregulates Dectin-1 and/or MR mediated phagocytosis.

*Supported by Nider Sachsen Israeli Cooperation*.

# Abstract: A Method for Odorant Comparison

**Authors:** R. Haddad, R. Kahan, D. Harel, N. Sobel

**Affiliations:** ^1^Department of Computer Science and Applied Mathematics, theWeizmann Institute of Science, Israel; ^2^Department of Neurobiology, Weizmann Institute of Science, Israel

In order to study a sensory system, one needs to quantify the response to stimuli that vary systematically. Whereas in vision and audition one can vary the stimuli by wavelength and frequency, respectively, there is no equivalent metric in olfaction, and the rules linking neural responses to odorant features remain unknown. Several studies have linked response patterns to restricted odorant features, such as number of carbons, and type/position of functional group. These metrics, however, fail to account for response patterns to odorants varying along other structural dimensions. To generate a multidimensional odor metric, we obtained 1664 molecular odorant features, and generated a metric where each molecule was represented as a vector of descriptor values. Revisiting numerous studies, we found that this novel olfactory metric was always better at accounting for neural responses than the specificmetric used in each study. Critically, this single metric was equally applicable across studies in different animals, and olfactory neurons that used odorants varying along different feature types and different neuronal response measurement methods, where it explained previous anomalies in odorant response profiles. Thus, we propose an olfactory metric that provides a unified account of independently obtained results, and offers a quantitative measure for odorant comparison. Finally, we recommend on sets of odorants that are best in terms of spanning the physicochemical space for use in olfactory neuronal response experiments.

# Abstract: Novel spine-like gold structures as a three dimensional substrate for neuro-electronic hybrids

**Authors:** A. Hai, D. Kamber, A. Dormann, J. Shappir, S. Yitzchaik, M. Spira

**Affiliations:** ^1^Department of Neurobiology, The Life Sciences Institute; ^2^School of Engineering, Israel; ^3^Department of Inorganic and Analytical Chemistry, The Hebrew University of Jerusalem, Israel

Integration of neurons with microelectronic devices has been a subject of intense studies over the last decade. One of the major problems in assembling efficient neuroelectronic hybrid systems is the weak electrical coupling between the components. This is mainly due to the fundamental property of living cells to form and maintain an extracellular cleft between the plasma membrane and any substrate to which they adhere. This cleft shunts the current generated by the neuron and the device and thus reduces the signal-to-noise ratio. Increasing the seal resistance formed between the neurons and the gate surface is thus a challenge which could improve the coupling coefficient. Here we report a new approach to improve the physical and electrical coupling between neurons and the surface of electronic chip by harnessing a basic property of cells namely, the internalization of particles by phagocytosis. To that end we fabricate gold micro-structures in the form of dendritic spines that protrude from the transistor gate surface (micrometer scale). The protruding structures were functionalized by poly-l-lysine. Using on line confocal microscope imaging of neurons expressing GFP-actin we observed that cultured Aplysia neurons readily engulf the goldspines forming tight physical contact between the cells and the surface of the device. Electron micrographs of the contact area confirm that the plasma membrane of the neurons engulfs the gold-spines and that the gap formed between the plasma membrane and the artificial spine is 30—70 nm. Measurements of the extracellular field potentials by passive gold spines indicate that the neuron-gold spine junction may improve the electrical coupling of neuro electronic hybrids significantly.

# Abstract: Sensory gating in primary insomnia: A potential objectivemeasure of reduced sleep quality

**Authors:** I. S. Hairston, L. S. Talbot, E. Polina, G. L. June, A. G. Harvey

**Affiliations:** Psychology Department, University of California, Berkeley, Calif, USA

Primary insomnia (PI) is the difficulty initiating and/or maintaining sleep, or experiencing nonrestorative sleep in the absence of another sleep disorder, psychiatric or physical cause. The hyperarousal hypothesis is the leading theory for the aetiology of PI. The model poses that due to cognitive and physiological hyperarousal, individuals with PI fail to experience the full deactivation of sensory and cognitive processing which normally occurs during sleep. A potential implication of this hypothesis is that processing of information during sleep would differ between people with PI and healthy sleepers. In this study we tested whether participants with PI exhibit impaired gating of external sensory stimulation during sleep. The markers of sensory processing included evoked K-complexes and changes in event-related potentials (ERPs). 15 participants meeting DSM criteria for insomnia, for a minimum of six months, and 15 controls slept in the lab for polysomnography and ERP testing. The ERP stimulus consisted of pairs of clicks (10 milliseconds ∼50 dB, ISI = 500 milliseconds, ITI = 3000 milliseconds). This stimulus was presented during wake, while watching a silentmovie, and then during subsequent sleep. Polysomnography data was staged, and blocks of stimuli that occurred during stage II were used for ERP analysis. The number of evoked K complexes was quantified by comparing number of K complexes without stimuli, to an equivalent period when stimuli were presented. Participants with insomnia had significantly increased P300 during wake and sleep, with a smaller attenuation to the second stimulus. During sleep, both groups showed an equivalent amount of evoked in K complexes in response to stimulation. However, overall participants with insomnia had more K complexes. These findings indicate that PI is associated with altered gating mechanisms in both sleep and wake, and is the first objective measure for sleep disorder known in this population.

*Supported by NARSAD, APA postdoctoral fellowship,* *and UNCF-Merck postdoctoral fellowship.*

# Abstract: The role of object comparison in category learning and the emergence of expertise

**Authors:** R. Hammer, G. Diesendruck, D. Weinshall, S. Hochstein

**Affiliations:** ^1^Interdisciplinary Center for Neural Computation, Israel; ^2^Neurobiology Department, Institute of Life Sciences, Israel; ^3^School of Computer Sciences and Engineering, Hebrew University, Jerusalem, Israel; ^4^Gonda Brain Research Center and Department of Psychology, Bar-Ilan University, Ramat-Gan, Israel

Category learning is a fundamental cognitive capacity that enables meaningful and economical representations of objects and events. Recent studies, both in the field of machine learning and in the field of cognitive development, stressed the importance of objects comparison for the process of category learning. We claim that although theoretically categories can be learned and refined by either identifying attributes shared by objects within specific categories as well as identifying the attributes discriminating objects from different categories, there is a difference in the usability of these two types of comparison by humans. In a recent study we compared adults' performance with that of children in a category learning task. We discovered that when presented only with objects known to be from the same category, children learned the categorization rules as well as adults. However, when presented only with objects from different categories, unlike adults, most children failed to learn the categorization rules. This difference between learning from pairs of the same versus different categories may explain known phenomena in cognitive development, such as the common difficulty of young children in learning subordinate-level categories, and the late emergence of expertise. We suggest that such difficulties results from cognitive, not perceptual, immaturity– mainly poor logico-inferential capacity for extracting information from exemplars identified as belong to different categories.

*This study was supported by* *a “Center of Excellence” grant from the* *Israel Science Foundation, a grant from the* *US-Israel Binational Science Foundation, and* *a grant by the EU under the* *DIRAC integrated project IST-027787.*

# Abstract: Purinergic signaling in satellite glial cells in sensory ganglia is enhanced in mousemodels of chronic pain

**Authors:** M. Hanani, R. Kushnir, P. S. Cherkas

**Affiliations:** Hebrew University, Hadassah Medical School, Hadassah University Hospital, Jerusalem 91240, Israel

Satellite glial cells (SGCs) are the main type of glial cells in sensory ganglia. The morphology of these cells has been described in detail, but little is known about the physiological and pharmacological properties. We have shown previously that SGCs in trigeminal ganglia (TG) are endowed with functional P2Y purinergic receptors, and the objective of the present work was to find out whether these receptors are altered under pathological states–axotomy of the infraorbital nerve or complete Freund's adjuvant (CFA) injection in the submandibular region of mice. Trigeminal ganglia were removed 4–7 days after treatments and were tested in vitro. We examined changes in intracellular Ca^2+^ in SGCs in TG in response to ATP using Fluo-3 or Fura-2 as Ca^2+^ indicators. The effects of submandibular inflammation were tested in short term cultures (20 h), and that of axotomy in intact ganglia. In controls the threshold of SGC response to ATP concentration was 5 microM (>20% of the cells responded), and reached maximum at 100 microM; at least 25 cells were examined at each concentration. In SGCs from treated mice the threshold for ATP effect was 0.01 microM (i.e., 500-fold lower than control) and reached maximum at 10 microM. These results apply for both treatments. In this work we showed for the first time that the sensitivity of SGCs in TG to the inflammatory mediator ATP greatly increased after axotomy and inflammation. We conclude that SGCs are likely to be involved in pathological changes in TG and might contribute to the abnormal signaling that takes place in chronic pain states.

*Supported by BSF and ISF.*

# Abstract: Prenatal hypoxia influences cerebellum gene expression

**Authors:** O. Haramati, V. Chalifa-Caspi, J. Perez-Polo, H. Golan

**Affiliations:** ^1^Developmental Genetics Faculty of Health Sciences and Bioinformatics Core Facility, Ben Gurion University, Israel; ^2^Bioinformatics Core Facility and the National Institute of Biotechnology, Ben Gurion University, Israel; ^3^Biochemistry and Molecular Biology, University of Texas Medical Branch, Galveston, Tex, USA; ^4^Developmental Genetics Faculty of Health Sciences and Zlotowski Center, Ben Gurion University, Israel

Prenatal hypoxia is related to a variety of motor dysfunctions and behavioral problems in the adult life. One of the treatments used for prenatal hypoxia is MgSO_4_ (Mg), although the exact mechanism is unknown. Our primary goal was to find gene networks that were modulated by hypoxia and elucidate the mechanisms involved in Mg prophylactic action. Pregnant mice were treated i.p. at 17 days gestation with Mg/PBS every 20minutes (4 hours) followed by 2-hour hypoxic (9% O_2_, 3% CO_2_ ) or normoxic conditions. The mice divided for 3 groups: SA (PBS+normoxia), SH (PBS+hypoxia) and MgH (Mg+hypoxia). Fetus cerebelli were isolated 2 hours, 24 hours, and 20 days after insult. mRNA was extracted and gene expression was analyzed by affymetrix DNA chips (mouse 430 2.0). Mixedmodel 2-Way ANOVA (Partek) was used to compare effect of treatment (SH-SA and MgH-SA). SH modified the expression of 7, 3 and 1127 genes, at 2 h, 24 h and 20 days after hypoxia, respectively (at least 1.5 folds change compared to SA group (FC) *P* < .05), while pretreatment with Mg followed by hypoxia changed the expression of 10, 10 and 832 genes at parallel times. Among the genes changed 20 days after insult 74 were modified more than 2.5 FC by SH and only 15 when Mg was applied (14 appeared in both comparisons). To identify signaling pathways modulated by the treatments, the Gene Set Enrichment Analysis was used. Three gene sets were modulated in at least 2 time points (FDR < 0.05). Cell cycle gene set was upregulated 2 h after hypoxia followed by down-regulation at later time points. Mg induced opposite influence upregulating cell cycle genes 24 hours and 20 days after the insult. Mitochondria gene set was downregulated at all times followed SH. Mg caused downregulation at 2 h and 20 days, in contrast to upregulation at 24 hours after the insult. Overall, prenatal hypoxia effect is most noticeable 20 days after the injury. Opposite change due to Mg pretreatment was observed in some times, suggesting interference in similar mechanism.

*Supported by BSF Grant no. 2003166*.

# Abstract: Is it a European car or a Japanese car? An ERP study of diagnostic information use in visual expertise

**Authors:** A. Harel, S. Bentin

**Affiliations:** ^1^Department of Psychology, Hebrew University of Jerusalem, Israel; ^2^Center of Neural Computation, Hebrew University of Jerusalem, Israel

Expertise in object recognition requires knowledge of the diagnostic visual features of the object. The nature of the diagnostic information and the stage in the visual processing hierarchy at which it is utilized are still unknown. We compared performance and event-related potentials (ERPs) of 15 car experts and 15 novices performing a category verification task. The objects categorized were from categories with which the two groups had different levels of experience (faces, cars, and airplanes) and were categorized at the basic and subordinate levels. The information contained in the images was manipulated by filtering them at different spatial frequencies (SF). Experts and novices showed equivalent performance for faces and airplanes. Face subordinate categorization relied more on low SFs than on high SFs, while subordinate categorization of airplanes relied more on high SFs. Subordinate categorization of cars, however, differed between experts and novices. Experts relied more on high SFs than on low SFs while novices performed at chance level irrespective of SF. Electrophysiologically, N170, an early face-selective ERP component, was modulated by expertise but this effect did not interact with SF or categorization level. In the experts' left hemisphere N170 amplitude in response to cars was equivalent to N170 amplitude to faces, both higher than to airplanes. In novices the N170 in response to cars was equivalent in response to airplanes, both being smaller in amplitude than the N170 to faces. Our findings suggest that the diagnostic information needed for expert object recognition is quantitatively and not qualitatively different from everyday object recognition, as similarly to airplane subordinate categorization in novices, expert subordinate car categorization relied on high SFs. Furthermore, the expert use of specific SF scales does not occur at the early perceptual stages reflected by the N170 and may be related to later post-perceptual processes.

# Abstract: Attentional resources become available to the auditory systemduring the Visual Attentional Blink

**Authors:** K. Haroush, S. Hochstein, L. Y. Deouell

**Affiliations:** ^1^Department of Neurobiology, Israel; ^2^Department of Psychology, Israel; ^3^The Interdisciplinary Center for Neural Computation, Israel

The Attentional Blink (AB) is a well-explored phenomenon during which a first target within a rapid sequence of stimuli is successfully detected yet a second target fails to be reported. The question whether the AB deficit is of merely visual nature or affects severalmodalities, such as auditory and tactile, has been extensively debated. Previous studies used dual task paradigms to address this question. Themain problem with this approach is that the dual tasking effect could not be separated from the blink effect. In the current study, we bypassed this obstacle by registering Event-Related Potentials (ERPs) to an unattended deviant stimulus within an auditory stream while participants are engaged in a visual AB paradigm as their only behavioral task. The mismatch negativity (MMN) ERP signature of involuntary change detection is the difference between the response to auditory standards and deviants. Surprisingly, we find that when conditional identification of the second visual target fails (at lag 3), the MMN significantly increases. We suggest that when the visual system blinks, attentional resources are freed up for use by the auditory modality, resulting in facilitated change detection processes as reflected in the augmented MMN response.

*Supported by the Israel Science Foundation* *and the US-Israel Binational Science Foundation.*

# Abstract: Cell-driven angiogenesis and neurogenesis after stroke with platelet derived microparticles

**Authors:** Y. Hayon, R. Leker, D. Varon, A. Brill

**Affiliations:** ^1^Neurology, Hadassah Medical Center, Hebrew University, Jerusalem, Israel; ^2^Hematology, Hadassah Medical Center, Hebrew University, Jerusalem, Israel

**Background**

Activation of endogenous neural stem cells (eNSCs) has been proposed as a novel form of therapy in a variety of neurological disorders, including stroke. Previous data suggest that application of external factors can boost a long-termendogenous repair mechanism. Upon activation platelets shed microparticles (PMPs) which contain a variety of growth factors that are central to angiogenesis and neurogenesis. Because interrelations between newborn neural and endothelial cells are essential to eNSC survival, maturation and differentiation we evaluated the effects of PMP after brain ischemia.

**Methods**

Spontaneously hypertensive rats underwent permanentmiddle cerebral artery occlusion (PMCAO) and were given BrdU to label newborn cells. Animals were treated with vehicle or with PMPs delivered via a biodegradable polymer applied to the brain surface immediately following injury. Animals were tested with a reproducible motor and sensory score for 90 days after PMCAO. Infarct volumes were measured at 90 days post—PMCAO. We used immunohistochemistry to evaluate the fate of newborn cells in the ischemic brain. Newly formed blood vessels were counted in the brains to evaluate angiogenesis.

**Results**

Our results demonstrate a dose dependent increase in the number of newborn cells in animals treated with PMPs and a significant increase in the number of blood vessels observed at the infarct boundary zone. Treatment with PMP significantly improved behavioral deficits in a dose dependent manner and there were no significant differences in infarct volumes between the different treatment groups.

**Conclusions**

PMPs significantly induce neurogenesis and angiogenesis after PMCAO in a dose-dependent manner and these effects are correlated with better functional outcome and reduced disability in the long term after stroke despite the lack of a neuroprotective effect. This strategy can be used in subsequent studies to further improve functional gain after brain injury.

*Supported by Peritz and Chantel Scheinberg*.

# Abstract: Adaptation to repetitive whisker stimulation is highly correlated among neurons in the rat barrel cortex

**Authors:** J. Heiss, E. Oron, A. Grinvald, I. Lampl

**Affiliations:** Department of Neurobiology, Weizmann Institute of Science, Israel

It is thought that adaptation in the barrel cortex results from short term synaptic depression of thalamocortical inputs. The probabilistic nature of synaptic transmission and its modulation by short term depression should contribute to cell-specific trial to trial variability during repetitive stimulation. Here we compared the trial to trial variability of subthreshold synaptic adaptation in vivo, during periodic whisker deflection in couples of simultaneously recorded cells. Additionally, we compared the adaptation exhibited by a large population of neurons, which was detected using fast optical imaging based on voltage sensitive dyes (VSDs), with the adaptation of intracellularly recorded cells. Because the VSDs signal reflects primarily the average synaptic potentials of the neuronal population, rather than spiking activity, this allowed us to extend the comparison of adaptation of synaptic inputs to the population level. Considerable trial to trial variability in adaptation was reveled by measuring the responsiveness of the cell to repetitive stimulation. These experiments showed a high correlation of the adaptation between nearby neurons recorded simultaneously as well as between a single cell and the optically monitored large population of responsive neurons in its vicinity. These results suggest that adaptation of cortical neurons depends not only on short term synaptic depression, but also on a coordinated process that homogenize adaptation among nearby neurons either at the thalamic or cortical level.

*This work was supported by ISF—The Charles H. Revson Foundation and the Goldsmith foundation.*

# Abstract: The Origin of Hering's Law of Equal Innervation

**Authors:** T. Hendel, M. Gur

**Affiliations:** Faculty of Biomedical Engineering, Technion–Israel Institute of Technology, Israel

Hering's law of equal innervation implies that eye movements are accomplished by two independent subsystems: one for purely conjugate movements (version) and one for purely disconjugate movements (vergence). Thus, in order to fixate a certain point in space, the oculomotor system does not move each eye separately, but, rather, sends the extra-ocular muscles a combination of version and vergence signals to direct the eyes to the sought for fixation point. Experiments devised to test Hering's law have indeed verified the existence of two types of eye movement, each with its own characteristics: versional movements are either extremely fast (hundreds of degrees/sec) preprogrammed saccadic movements or slow smooth pursuit movements, whereas vergencemovements are slow (tens of degrees/sec) smooth eye movements that are evoked by retinal disparity and rely on visual feedback. The clear discrepancy between the two types of eye movements has usually been taken as a confirmation of Hering's suggested law. However, more precise measurements of eye movements have shown seeming deviations from Hering's law in accommodative vergence experiments. In light of these apparent deviations from Hering's law, some researchers have completely abandoned the concept of equal innervation to the two eyes. Others retained the basic law, but hypothesized a “holding reflex,” which needs to be added to the other known types of eye movements. In contradistinction, we argue that Hering's law of equal innervation is actually demanded by the existence of the ancient (in evolutionary terms) conjugate system in the visual system. We then explain the “deviations” from Hering's law both qualitatively and quantitatively within the paradigm of equal innervation using the well-known mechanism of smooth pursuit.

# Abstract: Tone Detection in Noise in theMouse Auditory Cortex

**Authors:** I. Hershenhoren, I. Nelken

**Affiliations:** ^1^Department of Neurobiology, Edmund Safra Campus, Givat Ram, Israel; ^2^Interdisciplinary Center for Neural Computaion, Hebrew University, Jerusalem, Israel

Sounds in natural settings always appear over a noisy background, and the way the auditory system extracts sounds from such backgrounds is of extreme importance both theoretically and practically. The theory of masking of tones by noise drove human psychoacoustic research and resulted in arguably the most important concept in hearing, that of the critical band. In the mouse, the critical band is wide relative to other mammals. As a result, the mouse is a useful model for discriminating between peripheral and central processing mechanisms in masking. We are using intracellular and extracellular recordings in vivo in mice to study neuronal responses in the auditory cortex to tones masked by broadband noise or by slowly fluctuating broadband noise maskers. The masked threshold (lowest level at which the presence of a tone can be observed in the neuronal responses in presence of a masker) increases with noise level. Such results can be explained by energy masking in the peripheral auditory system: once the signal-to-noise ratio within the critical band centered at the target tone frequency is high enough, the tone is detected. However, when Additional information is supplied to the auditory system by slowly modulating the masker, masking can be reduced substantially below the values expected from pure energy masking.

*Supported by a grant from the Joint Niedersachsen–Israel program.*

# Abstract: Recognising your own hands: attribution biases & brainmechanisms

**Authors:** N. P. Holmes, P. Revol, A. Farné, G. Rode, C. Spence, G. Calvert, Y. Rossetti

**Affiliations:** ^1^Espace et Action, INSERM Unite 864, Bron, France; ^2^Department of Psychology, Hebrew University, Jerusalem, Israel; ^3^Department of Experimental Psychology, Oxford University, Oxford, UK; ^4^Department of Psychology, Bath University, Bath, UK

Visual self-recognition in humans is thought to depend upon activity in a number of frontal brain regions, particularly in the right hemisphere. Behaviourally, self-recognition tasks elicit a number of response-related biases. For example, in previous experiments investigating hand-response preferences in self-face-recognition, groups of participants who responded “self ” (or “mine”) with the left hand and “other” (or “not mine”) with the right hand, performed better than other groups of participants responding with the opposite stimulus-response mapping. It was suggested that such hand-response differences implicated a right hemisphere specialisation for self-recognition. We report experiments exploring an alternative hypothesis: that improved performance when using the left hand to respond “self ” may reflect the interaction of two different response tendencies. Our results showed that, when uncertain about the correct response, participants tended to press the left button, regardless of the stimulus-response mapping, and also tended to press the “not mine” button, reflecting a failure to recognise the stimulus. When “self ” responses were made with the left hand, these two tendencies worked in opposite directions, equalising the proportions of “self ” and “other” responses. When the reverse stimulus-responsemapping was used, however, both tendencies resulted in more ‘other’ than “self ” responses, decreasing self-recognition performance. In order to dissociate the neural mechanisms of self-recognition fromthose of decision-making, we also used slow event-related functional magnetic resonance imaging (ER-fMRI) and analysed blood-oxygenation level dependent (BOLD) activity in a number of right hemisphere regions of interest.

# Abstract: Stereotyped whisker and head movements are associated with object localization in unrestrained rats

**Authors:** G. Horev, P. M. Knutsen, M. Pietr, E. Ahissar

**Affiliations:** Department of Neurobiology, The Weizmann Institute of Science, Israel

It has been shown that rats use an active sensing process to discriminate offsets in horizontal (antero-posterior) location with their whiskers. The rats were trained to locate the position of two objects using their whiskers alone and to associate between the position of the objects and the position of a reward. Our goal here is to identify motor strategies that underlie this association. Kinematic variables of head and whiskers trajectories were compared between sequential contacts made with the objects in each trial, in rats that were trained to associate (Trained) and in rats that were trained to sense the objects but received the reward constantly (Naїve). Out of 12 variables tested, 3 showed significant changes between sequential contacts. These variables were whisker's angle relative to the head, head velocity, and touch duration. These dependencies were observed in both Trained and Naїve rats, however values differed significantly between the groups. We also observed that the variance of 3 variables: whisker angle velocity, whisker curvature and head velocity was significantly lower in Trained rats. Another variable, the distance between the eyes, was significantly higher in Trained rats. This parameter is highest when the head of the rat is parallel to the ground; therefore we conclude that in this task Trained rats tilt their head less than Naїve rats. Our results indicate that the behavior of Trained rats is more stereotyped than the behavior of Naїve rats. Meaning that in order to associate between the positions of the poles and the reward, these rats learned to control specific aspects of their behavior. Variables with reduced variance in trained rats are candidates to be controlled by the rats. The relations between these variables and more refined temporal and spatial variables which might encode spatial offsets for these rats during task performance will be studied next.

# Abstract: Individual differences in stress responsiveness affect the consequences of juvenile stress on coping with stress in adulthood

**Authors:** O. Horovitz, M. M. Tsoory, G. Richter-Levin

**Affiliations:** ^1^Psychology Department, University of Haifa, Israel; ^2^The Brain and Behavior Research Center, Israel; ^3^The Institute for the Study of Affective Neuroscience (ISAN), Israel

Epidemiological studies associate early life stress (ELS) with increased risk to develop PTSD and depression (Agid et al., 2000). While most ELS animal models concentrate on the perinatal to pre-weaning period (Sanchez et al., 2001) recent studies indicated “juvenility” in rats (∼28 days of age) as an ELS sensitive age (Tsoory and Richter-Levin, 2006). Sex and individual differences in novel setting exploration were associated with trait anxiety and stress responsiveness in both humans and animals (Shear et al., 2005, Muallâ et al., 1993, Wood and Shors, 1998, Cavigelli and McClintock, 2003; Mâllo et al., 2005; Mâllo et al., 2007). Therefore, the current study assessed the rats' novel setting exploration as an index of stress responsiveness and evaluated how it affects the consequences of juvenile stress on coping with stressful challenges in adulthood by utilizing the two way shuttle avoidance task and Saccharine preference. Compared with controls, adult juvenile stressed rats exhibited poor avoidance learning and a lower Saccharin preference following the avoidance task; No differences in Saccharine preference were evident prior to the avoidance task. Furthermore, “Low explorer” Controls exhibited a higher Saccharine preference than “High explorer” Controls, while an opposite trend was evident among adult juvenile stressed rats. These results lend further support for the juvenile stress as a model of induction of predisposition to develop mood and anxiety disorders following subsequent stress exposure in adulthood.

# Abstract: Lesions of the posterior vermis in the rhesusmonkey cause deficits in visual motion perception

**Authors:** A. Ignashchenkova, S. Dash, H. Dietrich, T. Haarmeier, P. Thier

**Affiliations:** Department of Cognitive Neurology, Hertie Institute for Clinical Brain Research, Germany

Patients with lesions of the cerebellum are often impaired in performing tasks involving motion perception, but the critical locus for such lesions remains vague. In order to study the nature of the deficit and to identify the region of the cerebellum that may be involved, we trained monkeys in tasks of visual acuity, spatial attention, luminance change detection, and the detection of coherent motion in a subset of dots moving within a random dot field. Two regions of the cerebellar cortex, namely, the oculomotor vermis (lobuli VI to VIII) and the dorsal paraflocculus, are likely candidates, since they receive visual inputs and hence may be involved in the motion perception. As a first step towards analyzing the critical locus we made vermal lesions including lobulus VI through VIII in three monkeys and tested them postoperatively on visually guided saccades and the perceptual tasks described before. All the monkeys showed the expected saccadic dysmetria immediately after the lesion, which subsided after a few days. Two monkeys tested for visual acuity showed opposite effects, one improved postlesion and another had deteriorated visual acuity, but both these monkeys showed preserved improvement in allocating covert visuo-spatial attention upon cueing. The third monkey was tested on luminance change detection task and had no impairment after the lesion. Overall, there were no general visual deficits in these monkeys after the lesion. Two out of the three monkeys tested for coherent motion detection task were impaired after the lesion. The third monkey which did not show any deficits after the lesion had a relatively small lesion. One of the impaired monkeys never recovered for a particular spatial location (16 deg, downward) where the effect of lesion was most profound. We conclude that the vermis plays a critical role in motion perception although other cerebellar areas might contribute as well.

# Abstract: The interplay between Juvenile stress and Enriched Environment on behavior and L1-CAM

**Authors:** Y. Ilin, G. Richter-levin

**Affiliations:** The Brain and Behavior Research Center, University of Haifa, Haifa, Israel

Recent evidence support the hypothesis that exposure to stress or trauma during early childhood may disturb the formation of functional brain pathways, in particular, of the limbic circuits. Previous findings from our lab indicate that an exposure of rats to a relatively brief stressful experience during juvenility (27–29 days of age) has profound and long-lasting behavioral effects (Avital and Richter-Levin, 2005; Tsoory et al., 2006). The importance of the environment in the regulation of brain, behavior and physiology has long been recognized in biological, social and medical sciences. Animals maintained under enriched conditions have clearly been shown to have better learning abilities than those maintained under standard conditions. We set out to investigate the long-term effects of Juvenile stress and effects of enriched environment (EE) on the ability of animals to cope with subsequent learning tasks and on the expression of the cell adhesion molecule L1, suggested to be involved in environment-induced neuronal re-organization. Three groups were tested: Juvenile Stress–subjected to Juvenile stress; Enriched Environment–subjected to Juvenile stress and then, from day 30 on to EE; and Naїves. In adulthood, coping and stress responses were examined using the elevated plus-maze, open field, exploration and avoidance learning. In adulthood, “juvenile” stressed rats exhibited reduced exploration in a novel setting, and poor avoidance learning, with learned helplessness-like behaviors, while exposure to EE impede these long lasting effects of juvenile stress. Preliminary findings suggest that these where associated also with lasting alterations in the expression of L1.

*This work was supported by the EU's PROMEMORIA Grant no. 512012 to G.R-L.*

# Abstract: Correlated neuronal activity in primary and pre motor cortices of monkeys during performance of different visuomotor association tasks

**Authors:** D. Inbar, Y. Grinvald, N. Zach, E. Vaadia

**Affiliations:** ^1^Department of Physiology, Hadassah Medical School, Israel; ^2^Interdisciplinary Center for Neural Computation (ICNC), Hebrew University, Jerusalem, Israel

It has been shown that patterns of neuronal activity in motor cortices and tuning curves might change during sensorimotor learning (Mitz et al., 1991, Gandolfo et al., 2000, Paz et al., 2003). In this study, we examine co-variation of response patterns and temporal cross-correlation to study dynamics of interactions between cells during performance and adaptation to different visuomotor tasks. Neuronal activity from monkeys' motor cortices was recorded in each session by a multimicroelectrode array. Each recording session was comprised of the following blocks of trials: (1) standard 8- direction center-out task (a well-known task); (2) adaptation to visuomotor-rotation; and/or (3) adaptation to arbitraryassociation (two colors associated with two different movement directions); (4) repetition of the standard center-out task. This poster presents patterns of correlation found in these different behavioral states. First, we demonstrate that correlation patterns between neighboring cells in M1 and Premotor cortices may change and examine if and how these changes are related to behavior. Pairs of cells with correlated firing patterns during performance of standard task, tend to change correlation strength or pattern during adaptation. This change may last during the repetition of the standard center-out task that ends the session. Second, the strength and pattern correlation between pairs of cells at the beginning of the trials might imply the responsiveness of these cells to behavioral events that follow. These results may suggest that the cortical network can reorganize during performance of visuomotor associations to generate correct behavior.

*Supported by a Center of Excellence grant (1564/04) by the Israel Science foundation (ISF), by Bundesministerium fr Bildung und Forschung (BMBF-DIP), and by the US-Israel Binational Science Foundation (BSF)*.

# Abstract: Molecular correlations following mating in the rodent female olfactory bulb

**Authors:** S. Inberg, K. Rosenblum, S. Wagner

**Affiliations:** Center for Brain and Behavior, Department of Neurobiology and Ethology, Haifa University, Israel

Mated female mouse learns the individual identity of the male during the mating and critical period of 4–6 hrours after the mating, subsequent exposure to another mouse or to his bedding during the 3-4 following days cause pregnancy block. This pregnancy failure is named “Bruce effect.” The neural changes underlying this associative memory to stud male occur in the accessory olfactory bulb at the first stage of sensory processing system, but it do not exclude the role of the main olfactory bulb in processing of olfactory cues in different social relationships, including in the case of female's memory formation to stud male. We are investigating the changes in both protein expression and protein post translation modifications induced in the olfactory bulb of female mice by mating. We found that there is an increase in GABAAReceptor-alpha 1 subunit level in days 1, 3 aftermating, followed by a return to basal levels (control group) in day 6. These changes occur in parallel to an increase in p- ERK levels in days 1, 3 and return to basal levels in day 6. We hypothesize that these changes are part of the molecular mechanism mediating the female's memory formation to stud male.

# Abstract: Neural correlates of recollection- and familiarity-based memory decisions

**Authors:** A. Ishai, M. Wiesmann

**Affiliations:** ^1^Institute of Neuroradiology, University of Zurich, Switzerland; ^2^Institute of Neuroinformatics, University and ETH Zurich, Switzerland

We used event-related fMRI to investigate whether recollection- and familiarity-based memory judgments are modulated by the degree of visual similarity between old and new art paintings. During the study phase, subjects performed a flower detection task, followed by a Remember/ Know/New surprise memory test. The old paintings were randomly presented with new paintings, which were either visually similar or visually different fromthe old items. Consistent with our prediction, subjects were significantly faster and more accurate to reject new, visually different paintings than new, visually similar ones. The proportion of false alarms, namely Remember and Know responses to new paintings, significantly decreased with decreased visual similarity. Moreover, Know responses were associated with significantly longer latencies. The retrieval task evoked activation within multiple visual, parietal and prefrontal regions, within which Remember responses elicited stronger activation than Know responses. New, visually different paintings evoked weaker activation than new, visually similar items in the intraparietal sulcus. Contrasting Recollection with Familiarity revealed activation predominantly within the precuneus, where the hemodynamic response elicited by Recollection peaked earlier than the hemodynamic response evoked by Familiarity. Our results suggest that successful memory retrieval of complex pictures is mediated by a distributed cortical network with differential hemodynamic profiles during Recollection- and Familiarity-based judgments, and support the “mnemonic accumulator” hypothesis, according to which recognition memory decisions are based on the integration of sensory signals.

*Supported by the Swiss National Science Foundation Grant 3200B0-105278 and Swiss NCCR: Plasticity and Repair*.

# Abstract: Supported by the Swiss National Science Foundation Grant 3200B0-105278 and Swiss NCCR: Plasticity and Repair. Association between the oxytocin receptor (OXTR) gene and pro-social behavior in two economic games: the Dictator and Social Value Orientations

**Authors:** S. Israel, A. Knafo, M. Reibold, E. Lerer, G. Bornstein, R. P. Ebstein

**Affiliations:** ^1^Department of Psychology, Hebrew University, Jerusalem, Israel; ^2^S. Herzog Memorial Hospital, Jerusalem, Israel; ^3^Human Genetics, Hebrew University, Jerusalem, Israel

Animal models demonstrate the role of oxytocin in modulating affiliative behaviors. Oxytocin (OXT) is linked to maternal and psychosexual behaviors in lower mammals and pair-bond formation in prairie voles. Moreover, recent investigations show that OXT facilitates social cognitive abilities: for example, OXT knockout mice show deficits in social memory. Recent research suggests that OXT is involved in prosocial behavior in healthy humans; intranasal OXT increases trust, recognition of emotion in faces, and reduces social anxiety. Importantly, three recent investigations provide convergent evidence for association between oxytocin receptor (OXTR) single nucleotide polymorphisms (SNPs) and autism. Our own study, shows that association with autism is mediated by acquisition of adaptive social skills. In two independent studies, we have recently demonstrated that a related nonapeptide receptor, arginine vasopressin 1a (AVPR1a), is associated with both autism and pro-social giving behavior in economic tasks. In the current investigation, we demonstrate that OXTR SNPs and haplotypes predict pro-social allocation of funds in two economic games, Dictator and Social Value Orientations. Using a family based method, we observed preferential transmission of individual alleles in the Dictator Game (UNPHASED) for SNPs rs1042778 (*P* = .0504) and rs2268494 (*P* = .0218) as well as in Social Value Orientations (PBAT) for SNP rs2268494 (*P* = .0009). Notably, these two SNPs also conferred risk for autism in our recently published study. In summary, our laboratory has shown association for two social neuropeptide receptors OXTR and AVPR1a with both autism and prosocial behavior as measured in economic games. Acknowledgements: Hebrew University BINCA grant (GB, RPE and AK), the IsraelNational Institute for Psychobiology (AK), the Israel Science Foundation (GB) and Phillip Morris International (RPE)

# Abstract: Comparing eyemovements to detected vs. un-detected stimuli

**Authors:** M. Jacob, S. Hochstein

**Affiliations:** ^1^Interdisciplinary Center for Neural Computation, Israel; ^2^Department of Neurobiology, The Hebrew University of Jerusalem, Israel

Why do we perceive some elements in a visual scene, while other stimuli remain un-detected? To learn about the sequence of events leading to detection, we are studying the difference between fixations on detected versus un-detected stimuli. We are using two tasks for this study, the Set game§(ISFN, 2005-6) and a novel Identity Search task, designed to contain detected and comparable un-detected stimuli, and a division into distinct search-fixation regions. In addition, it is easy to generate numerous displays for either task, and they require several seconds of search, but result in momentary recognition. Cards in the Identity Search task contain a square array of scrambled mixtures of black and white square units. Card number, array size and choice of number of black square units are under experimenter control. Within each display, all cards are different, except for two pairs of identical cards. Subject task is to find one of these pairs, indicating the cards with mouse clicks. The entire display is replaced for each trial. We compared fixations on cards belonging to the detected pair with those on the undetected pair in the same display. We find that detected pair cards are fixated more frequently than undetected pair cards, and that within the search sequence, there are fewer intervening fixations on other cards between fixations on detected than on undetected pair cards. These findings raise the question of what comes first: does observing certain cards more frequently and more closely lead to finding the pair, or does an implicit sense of the presence of a pair lead to these features and ultimately to the explicit “aha” discovery of the pair? Analysis of the break point between selective fixations on detected cards, as found for the Set game, may provide an answer to this question.

*Supported by the Israel Science Foundation(ISF) and the USIsrael Binational Science Foundation(BSF)*.

# Abstract: Short and long term consequences of exposing rats to stressors during juvenility

**Authors:** S. Jacobson-Pick, G. Richter-Levin

**Affiliations:** The Brain and Behavior Research Center, University of Haifa, Mount Carmel, 31905 Haifa, Israel

**Background**

Previous studies indicate that childhood emotional trauma serves as a major predisposing factor for the emergence of affective disorders later in life. It is not clear thoughwhether childhood stressful experiences serve as a predisposing factor or perhaps they prompt the onset of the disorder itself. Methods: the current study utilized the “juvenile-stress” model to examine its effects on anxiety indices and exploratory behavior in the open field and elevated plus maze, as well as on circulating corticosterone levels. These effects were evaluated both soon after the exposure and in adulthood, compared to controls. Additionally, the effects of acute corticosterone administration on anxiety indices and exploratory behavior of juvenile and adult rats were also examined compared to controls. Results: soon after the exposure, juvenile stressed rats exhibited increased levels of activity and spent more time in the “unsafe” regions of the arenas compared with unexposed rats. A reverse pattern of effects was evident when tested in adulthood. Similar results were obtained when juvenile and adult rats were injected with corticosterone thirty minutes before tested. Nevertheless, in comparison with naïve rats, rats that were exposed to “juvenile stress” and challenged in the open field and elevated plus maze exhibited increased CORT levels both soon after the exposure and in adulthood.

**Conclusions**

Opposite pattern of effects was found in juvenility and in adulthood, but increased levels of CORT seems to be involved in both. Taken together, this differential outcome may correspond with different age-dependent reactivity to stress hormones.

*This work was supported by the EU's PROMEMORIA Grant no. 512012 to GRL.*

# Abstract: Mapping regions for high frequency stimulation in two rat models of obsessive-compulsive disorder

**Authors:** D. Joel, O. Klavir, C. Winter

**Affiliations:** ^1^Department of Psychology, Tel-Aviv University, Israel; ^2^Department of Psychiatry and Psychotherapy, Charit´e CampusMitte, University Medicine Berlin, Germany

Obsessive-compulsive disorder (OCD) represents one of the most common psychiatric disorders. Functional and structural imaging studies implicate the involvement of basal ganglia-thalamo-cortical circuits in the pathophysiology of this disorder. In patients remaining resistant to pharmacoand behavioral therapy, ablative lesions of structures and pathways within these circuits have been shown to reverse clinical symptoms. Following the finding that high frequency stimulation (HFS) can replace lesion in the treatment of Parkinson's disease and other motor disorders, there have been attempts to establish HFS also for the treatment of OCD. To date there have been several case reports on the effects ofHFS of the anterior limb of the internal capsule and of the ventral striatum in OCD patients, and of the subthalamic nucleus in patients with comorbid Parkinson's disease and OCD. The results of these studies are encouraging in showing that HFS may be effective in the treatment of OCD. Yet the variety of targeted brain areas, the inconsistency in the demonstration of beneficial effect and the variability in the time needed to obtain a therapeutic effect, highlight the need for better mapping of brain regions whose stimulation may produce beneficial effects in OCD patients. We have recently addressed this issue by assessing the effects of HFS of the subthalamic nucleus, nucleus accumbens and globus pallidus in two rat models of OCD, the signal attenuationmodel and the quinpirolemodel. These models differ in terms of the manipulation used to induce compulsive-like behavior, the nature of the compulsive-like behavior, and the response to pharmacological treatment. These differences make it more likely that a demonstration in the two models of an anticompulsive effect, reflects a genuine anticompulsive effect of HFS of the brain region assessed, rather than being specific to some parameter of a particular model that is not necessarily related to the modeled disorder.

# Abstract: Local circuit plasticity in the dentate gyrus induced by a week titanic stimulation

**Authors:** M. Jubran, G. Richter-Levin

**Affiliations:** Department of Neurobiology and Ethology, Center of Brain and Behavior, University of Haifa, Israel

The hippocampus plays an integral role in learning and memory processes via its multiple intra and extra neuronal connections. Hippocampal long-term potentiation (LTP) of excitatory synaptic transmission was extensively examined and was proven to be accompanied by lasting modifications of GABAergic interneurons. However, several studies suggest that these two seemingly analogous forms of plasticity might exhibit dissociated pharmacological sensitivity and diverse susceptibility to modulation. In the present study, we examined the in vivo effect of a week titanic stimulation (WTS), ineffective in producing efficient LTP, to the perforant path (PP) on GABAergic interneurons activity and plasticity in the hippocampal dentate gyrus (DG) of the anesthetized rat. Frequency-dependent inhibition (FDI), a GABA mediated feed-forward inhibition, and paired-pulse inhibition (PPI), a form of feed-back inhibition were used to reflect the activity of GABAergic interneurons in the DG. We report here that although the application of WTS did not induce long-lasting modifications of the population excitatory post-synaptic potential in the granule cells of the dentate gyrus, it succeed to induce lasting reduce baseline inhibition as indicated by FDI and PPI. These results demonstrate a potential involvement of interneuronal plasticity in conditions where electrically induced principal cells plasticity is not apparent or fails to be maintained.

# Abstract: Failure of neurons to regenerate after axotomy stems fromthe assembly of an endbulb rather than a competent a growth cone

**Authors:** D. Kamber, H. Erez, M. E. Spira

**Affiliations:** Department of Neurobiology, Institute of Life Science, The Hebrew University of Jerusalem, Israel

Current understandings of the mechanisms that underlie success or failure of neurons to regenerate after axotomy emphasize two classes of factors: the intrinsic capacity of the neuron to regenerate, and the presence of extrinsic factors that either inhibits or support regrowth. A critical step in regeneration of transected axon is the transformation of the cut axonal end into a specialized compartment which orchestrates in time and space the use of cell resources for growth processes, and integrates extracellular signals into growth patterns, target recognition and synaptogenesis. The failure of a cut axonal end to transform into a competent GC apparatus and it's transformation into an endbulb (retraction bulb) was recognized as a factor which critically interferes with regeneration by the pioneering studies of Ramon y Cajal (1928). Nevertheless, whereas the mechanisms that transform the cut axonal end into a competent GC have been investigated, the mechanisms underlying endbulb formation and preventing regenerative processes still remain elusive. In the symposium I will (a) describe the self-assembly mechanisms that underlie the transformation of a differentiated axon into a functional growth cone after axotomy (Erez et al., 2007); (b) analyze recent experimental results that relate improper restructuring of cytoskeletal elements at the cut axonal end to the failure to assemble a competent GC by neurons with high capacity to regenerate; finally (c), I will discuss pharmacological and molecular approaches to overcoming the subcellular barriers that impede regrowth from the endbulbs.

# Abstract: On themechanism that promote controlled and targeted regrowth of an injured axonal branch out of many

**Authors:** D. Kamber, M. E. Spira

**Affiliations:** Department of Neurobiology, The Hebrew University of Jerusalem, Israel

Following three days in culture, the morphology and dimensions of cultured Aplysia neurons reach stead state (SS). We found that under these conditions transection of one axonal branch is followed by its regrowth while other noninjured branches maintain their morphology unchanged. Two mechanisms can account for this differential regrowth: axonal injury “label” the microtubule (MT) tracks that connect the site of injury with the cell body. Thereby, recruited cellular components are preferentially transported to the site of injury. Alternatively, the recruited cellular resources are equally transported to all branches but are preferentially “captured” and utilized at the site of injury by a newly formed growth cone. The termination of the preferential growth process should involve timely down regulation of the preferential supply or the “capture” of the cellular resources. Using on line confocal imaging of the cytoskeletal elements and organelles we suggest that simple principals govern the phenomenon of preferential growth. Non growing neurons maintain SS dimensions by the equal rates of membrane retrieval and membrane fusion within any axonal segments. Axonal transaction reduces the rate of membrane retrieval in proportion to the removed surface area of the distal branch(s), while the rate of transport of Golgi derived vesicles to the site of injury remains unchanged and is proportional to the number of MT tracks leading from the somata to the site of injury. The anterogradely transported vesicles fuse with the plasma membrane at the newly formed GC. We suggest that since the rate of membrane retrieval is proportional to the surface area, while the regenerating branch grow to its original dimensions the rate of membrane retrieval increases until it equals the rate of membrane supply. At this point the regrowth processes will be terminated.

# Abstract: Boundary conditions of corticosterone-dependent enhancement or alteration ofmemory consolidation

**Authors:** N. Kaouane, L. Calandreau, F. Esclassan, S. Tronel, N. Mons, R. Jaffard, N. Abrous, P. Piazza, A. Desmedt

**Affiliations:** ^1^CNIC, CNRS UMR 5228, Bordeaux 1, France; ^2^INSERM U588, Bordeaux 2, France

Extensive evidence indicates that administration of the adrenocortical hormone corticosterone (CORT) facilitates the consolidation of emotional experience. Nevertheless, high release of CORT, which can occur in extreme stressful situation, can result in memory deficits. This suggests that beyond a certain level of stress, the influence of CORT on memory consolidation may switch from a facilitating effect to a deleterious effect, leading to maladaptive emotional responses with regards to the aversive learning experience. Nevertheless, the boundary conditions under which such a switch can be observed are still elusive. In order to specify these conditions, we used in mice two fear conditioning procedures known to result in a preferential conditioned fear response either (i) to a discrete tone (i.e., tone-shock pairing: the tone is predictive of a mild footshock occurrence) or (ii) to contextual cues (i.e., tone-shock unpairing: tone not predictive). First, we show that increasing the intensity of footshock from a very low (0.3 mA) to a relatively high (1 mA) level gradually enhances conditioned fear responses in an adaptive manner, i.e., as a function of the tone-shock contingency. However, beyond the critical footshock intensity of 1mA, fear responses become maladaptive, i.e., independent on the conditioning procedure. Second, preliminary results indicate that under mild footshock intensity (0.5 mA), post-training intra-hippocampal infusions of CORT dose-dependently enhance adaptive conditioned fear responses. However, beyond a critical dose maladaptive conditioned fear responses are again observed. Finally, we also assessed whether the CORT-dependent switch from adaptive to maladaptive fear responses was associated with changes in ERK1/2 activation-related recruitment of the hippocampal-amygdalar circuit. Altogether, these results throw light on boundary conditions of CORT-dependent normal and pathological fear learning.

*This study was supported by the CNRS, the FRC, and a PICS No. 3939*.

# Abstract: Novel environment and co-application of NMDA and dopamine induce rapid translation of RSK2 in theMature Hippocampus

**Authors:** H. Kaphzan, G. Doron, K. Rosenblum

**Affiliations:** Department of Neurobiology and Ethology, Faculty of Science, University of Haifa, Israel

Ribosomal S6 kinase2 (RSK2) is known to take part in several signal transduction cascades including Mitogen Activated Protein Kinase/Extracellular Regulated Kinase (MAPK/ERK). Following our recent observation that ERK can serve as a coincidence detector for fast and slow neurotransmission in the hippocampus, we analyzed the status of RSK2 phosphorylation subsequent to application of NMDA, dopamine, or both to preparations of mature hippocampal slices in Sprague–Dawley rats. RSK2 was indeed phosphorylated; however, in addition, the amount of RSK2 protein (60%) was induced within 10min following stimulation. Moreover, the induced expression of RSK2 could be detected in both the cell body layer and the dendrites of hippocampal CA1 cells. Pharmacological analysis showed that RSK2 induction was MAPK ERK Kinase (MEK)-ERK independent, but mammalian Target of Rapamycin (mTOR) and translation dependent. We suggest that the fast kinetics of RSK2 translation that follows physiological stimulations, together with recent observations that its overexpression is vital for the attenuation of major signal transduction cascades, indicate an expanded physiological function of RSK2 in neurons, and sheds new light on the role of RSK2 in the Coffin– Lowry syndrome.

# Abstract: The correlation of intracellular zinc, zinc homeostatic proteins and apoptosis

**Authors:** N. Karol, M. Hershfinkel, M. Forberg, T. Kaisman, D. Fishman, I. Sekler, W. Silverman

**Affiliations:** ^1^Department ofMorphology, Zlotowski Center for Neuroscience Ben-Gurion University Beer Sheva, Israel; ^2^Department of Physiology Zlotowski Center for Neuroscience Ben-Gurion University Beer Sheva, Israel

A rise in intracellular Zn^2+^ has long been considered the principle challenge necessitating the impressive array of zinc homeostatic proteins present in mammalian cells. We and others have begun to focus on another, separate pool of Zn^2+^, associated with zinc homeostatic proteins (ZHP) and mitochondria which is dynamically regulated. These proteins bind zinc with a relatively high affinity but release it in response to oxidative stimuli, for example, reactive oxygen species, hypoxia or nitric oxide (NO). By virtue of its ability to damage mitochondria and activate apoptoptic signaling cascades, Zn^2+^ released in this way could play an important role in cell death in the developing and ischemic CNS. To establish an association between developmental cell death and intracellular zinc (Zni), we have adopted a flow cytometry strategy designed to analyze large numbers of developing cells from two brain regions: the olfactory bulb (OB) and cerebellum (CB). After isolating the cells in suspension, they are loaded with a zinc-sensitive dye, Zinpyr-1, and briefly exposed to a cell death marker, e.g., propidium iodide or Annexin-V prior to analysis in a FACSCanto flow cytometer. Induction of Zn^2+^ release from metallothioneins, e.g., by addition of a nitric oxide donor, or cell death in an identified neuronal population, e.g., by fetal ethanol exposure, allows us to correlate the specific challenge to observed levels of Zni in dying or surviving cells. Through these experiments, and together with in vitro studies we are conducting, we hope to gain insight into the role of intracellular zinc in basic physiological processes such as cell death.

*Supported by Israel Science Foundation (572/04) WS*.

# Abstract: Effect of anesthesia and breathing on the receptive field size of thalamic cells in the somatosensory system

**Authors:** Y. A. Katz, I. Lampl

**Affiliations:** Weizmann Institute of Science, Israel

The concept of receptive field (RF), the set of external stimuli external stimuli that will significantly alter the firing pattern of a neuron, is fundamental to sensory processing. Whether RF structure and size depends on the state of wakefulness or the physiology of the animal or not, is controversial. Cortical neurons of the somatosensory cortex (S1) of the rat respond to several whiskers. The origin of large RF of S1 neurons remains unclear. In a previous study we suggested that large RF emerged by cortical integration and that the RF of cells in the ventral posterior medial thalamic nucleus (VPM) are confined mostly to a single whisker. Other studies suggested that large RF of cortical cells reflect large RF in the VPM. In particular, large RF was demonstrated under light anesthesia or when a state of wakefulness is mimicked by stimulation of the brainstem reticular formation. In contrast, we found that under very light anesthesia RF in the VPM is mostly confined to one whisker. Hence, varying the level of anesthesia does not have a significant effect on their RF size. In these experiments, however, tracheotomized rats were artificially respirated. In self breathing rats, showing similar reflexes and fast EEG spectrum as in respirated rats, RF size was larger (2.1 ± 0.4 compared to 1.2 ± 0.1 in respirated rats). Despite the larger thalamic RF in self breathing rats, the number of whiskers that evoke response falls short of the size that was reported in several other studies. Further study is required in order to reveal the physiological factors which determine the RF of thalamic cells.

*This work was supported by The Israel Science Foundation Grant 1037/03*.

# Abstract: Constraining compartmental models using a parameter peeling procedure

**Authors:** N. Keren, A. Korngreen

**Affiliations:** ^1^Mina and Everard Faculty of Life Sciences; Leslie and Susan Gonda Brain Research Center, Israel; ^2^Bar-Ilan University, Israel

Constraining compartmental models of neurons using stochastic algorithms has become a critical task in the study and understanding of neuronal behaviour. We have recently suggested that measurements from multiple different locations along the dendrite and the axon facilitate in fitting a set of model parameters. However, practical data recordings from L5 pyramidal rat neurons are feasible from two locations simultaneously (soma + dendrite). Moreover, the common simulative model assumes axonal physiology which has several erroneous assumptions such as high channels concentrations that try to overcome the high potential needed for generating an action potential at the axon hillock. In order to estimate the parameters of a compartmental model describing only the somato dendritic section of the neuron we have developed a progressive process that fits the data using a parameter pealing procedure. In this procedure we assume recorded experimental data from both the soma and dendrite. We use a voltage-clamp placed at the soma to inject the voltage read from experimental data, and a current-clamp electrode at the dendrite to fit by the experimental data. This method allows us to disregard complexities arising from axonal physiology. In addition, in each step we constrain only part of the parameters thus making sure that we do not fall into local minima. Our results show accurate parameters fit and propose a protocol for constraining a model for a segment of a complex neuron.

# Abstract: ERP Source estimation by MRI constrained linear solutions

**Authors:** A. S. Keren, A. B. Geva, L. Y. Deouell

**Affiliations:** ^1^The Interdisciplinary Center for Neural Computation, The Hebrew University of Jerusalem, Israel; ^2^Department of Psychology, The Hebrew University of Jeruslalem, Israel; ^3^Electrical and Computer Engineering, Ben-Gurion University of the Negev, Israel

Intensive efforts are made to integrate EEG and fMRI data in order to benefit both from the high temporal resolution of the first and from the good spatial localization of the latter. Here we propose a platform based on structural and functional MRI, where established as well as novel estimation methods can be applied in order to estimate the sources of Event Related Potentials (ERP). The extraction of anatomical and functional constraints is accomplished by incorporation of imaging, experimental and computational tools: Both fMRI and ERP data are collected from the same subject in similar paradigms. The cortical surface of the subject is extracted from the structural MRI and represented as tiled triangles or linked nodes. On each node marked as active by a relevant fMRI contrast a dipole is seeded, oriented orthogonally to the cortical mesh. An electric model of the head is used to estimate the projection of the dipole at each node on each scalp electrode. Based on the resultant projection or “ lead filed” matrix, linear estimation methods such as Weighted Minimum Norm and Weighted Minimum Laplacian (AKA LORETA) can be applied with proper adjustments to surface geometry. The linear framework can be generalized to incorporate spatiotemporal constraints and filters, which capture richer physiological mechanisms. The proposed platform is demonstrated on real data from a human subject.

# Abstract: A functional circuit underlying male sexual behavior uncovered in the adult female mouse brain

**Authors:** T. Kimchi, J. Xu, C. Dulac

**Affiliations:** Department of Molecular and Cellular Biology, HHMI, Harvard University, Cambridge, Mass, USA

Pheromones regulate aspects of social and sexual behaviors throughout the animal kingdom. In mice, pheromone detection is mediated by the vomeronasal organ (VNO) and the main olfactory epithelium (MOE). Male mice deficient for TRPC2 (TRPC2−/−), an ion channel essential for VNO sensory transduction, are impaired in sex discrimination and male-male aggression. We report here that TRPC2−/− female mice show loss of sex discrimination and reduction in female-specific behavior, which includes maternal aggression and lactating behavior. Most strikingly, mutant females display unique characteristics of male sexual and courtship behaviors such as mounting, pelvic thrust, solicitation, anogenital olfactory investigation, and emission of complex ultrasonic vocalizations. The same behavioral phenotype is observed after VNO surgical removal in adult animals, and is not accompanied by disruption of the estrous cycle and sex hormone levels. These findings suggest that VNO-mediated pheromone inputs act in wild-type females to repress male behavior and activate female behaviors. Moreover, they imply that functional neuronal circuits underlying male-specific behaviors exist in the normal female mouse brain. Last, we have preliminary evidence that the neuronal switch that regulates sex-specific mating behavior might be related to dopaminergic cell population in the hypothalamus.

# Abstract: A critical role for histone 3methylation in thermal control development

**Authors:** T. Kisliouk, N. Meiri

**Affiliations:** Institute of Animal Science, ARO, The Volcani Center, Bet Dagan, 50250 Israel

Thermoregulation like other sensory mechanisms develops during a critical period. Neuroanatomically, the body temperature is balanced by the preoptic anterior hypothalamus (PO/AH) and controlled by thermo-sensitive neurons. Thermal-input during the critical period of thermal control establishment causes a plastic change in the ratio between thermo-sensitive neurons and innate PO/AH cells and can modulate temperature tolerance. Here we describe epigenetic changes associated with thermal-control establishment in chicks. During thermal conditioning (Heat exposure of 37.5°C of 3-day-old chicks, for 24 hours) there is a transient induction in both histone H3 dimethylation at lysine 9 (H3-K9) and at lysine 27 (H3-K27) in the PO/AH. The peak of dimethylation occurred at 2 h for H3-K9 and at 6 h for H3-K27. These chromatin modifications are limited to the critical period of thermal control establishment. When chicks that had passed the critical age (10-day-old) were exposed to similar thermal treatment there were no significant changes in histone H3 methylation. The enzymes that catalyze these modifications, G9a and EZH2 respectively are induced during similar time windows. Co-localization of H3-K27 and EZH2, during heat conditioning was verified by double immunofluorescence staining. Furthermore, attenuation of EZH2 expression using antisense inhibition alters thermal responses both during heat conditioning and during heat challenge later in life. To correlate the histone covalent modification with thermal regulation and neuronal plasticity, the feasibility of BDNF for transcription during heat conditioning was analyzed using the chromatin immunoprecipitation technology (ChIP). It was established that H3- K9 and H3-K27 exert different effects on the DNA-histone proximity in the promoter area of BDNF. Taken together, these results correlate epigenetic chromatinmethylation with thermal- adaptation-related hypothalamic plasticity.

*Supported by ISF*.

# Abstract: The effects of high frequency stimulation and temporary inactivation of the globus pallidus in the signal attenuation ratmodel of obsessive compulsive disorder

**Authors:** O. Klavir, C. Winter, D. Joel

**Affiliations:** ^1^Department of Psychology, Tel-Aviv University, Israel; ^2^Department of Psychiatry and Psychotherapy, Charit´e CampusMitte, University Medicine Berlin, Germany

Obsessive-compulsive disorder (OCD) is a common psychiatric disorder. Functional and structural imaging studies implicate the basal ganglia-thalamo-cortical circuits in the pathophysiology of this disorder. In patients remaining resistant to pharmaco- and behavioral therapy, ablative lesions within these circuits have been shown to reverse clinical symptoms. Following the finding that high frequency stimulation (HFS) can replace lesion in the treatment of Parkinson's disease and other motor disorders, there have been some encouraging attempts to establishHFS also for the treatment of OCD. Yet the variety of targeted brain areas, the inconsistency in the demonstration of beneficial effect and the variability in the time needed to obtain a therapeutic effect, highlight the need for better mapping of brain regions whose stimulation may produce beneficial effects in OCD patients. We are attempting to address this issue by mapping regions for HFS for the treatment of OCD, using the signal attenuation rat model of this disorder. In this model, the attenuation of an external feedback for lever-press responding leads, in a subsequent extinction test, to excessive leverpressing that is not accompanied by an attempt to collect a reward. This behavior, which has been named “compulsive” lever-pressing, has been shown to have face, predictive and construct validity as a model of compulsive behavior in OCD patients. We have recently found that HFS of the subthalamic nucleus (STN) reduces compulsive lever-pressing. The present study tested the effect of HFS of the globus pallidus (GP), which is interconnected with the STN, on compulsive lever-pressing. In addition, in order to better understand the mechanism of action of HFS, we have also tested the effect of temporary inactivation of the GP.

# Abstract: In vivo two-photon calcium imaging of periglomerular neurons in themouse olfactory bulb

**Authors:** H. Kopel, A. Mizrahi

**Affiliations:** Department of Neurobiology and the ICNC, the Hebrew University of Jerusalem, Israel

Adult neurogenesis is restricted to a few regions in the mammalian brain. One of these regions is the olfactory bulb (OB), the first station in olfactory processing pathway. In the OB, newborn neurons integrate into the circuitry as fully functional local interneurons (INs). These INs formthe local network of granule cells and periglomerular neurons (PGNs), both of which are thought to modulate incoming and outgoing olfactory input, respectively. However, the contribution of the newborn cells to the activity of the existing networks, if any, remains unknown. The long-term goal of this study is to reveal the functional role of PGNs within their local networks. To address this issue, we first developed an experimental system to record sensory-evoked physiological activity from PGNs, in vivo. Here we report preliminary data of PGNs physiological responses based on imaging of calcium transients and describe our experimental protocol for targeting adult-born PGNs. We imaged PGNs located at the dorsal surface of the OB using bolus loading of the calcium indicator oregon green bapta-1 AM (OGB) into the glomerular layer. OGB loading resulted in labeling of hundreds of neurons that showed strong calcium responses (upto 30% dF/F) to electrical stimuli. In order to characterize the sensory response profiles of the PGNs we are currently mapping the activated regions of the OB in response to specific olfactory stimuli using intrinsic-signal imaging (ISI). IS maps are then used to identify the activated glomeruli that are subsequently imaged with two-photon calcium imaging. In addition, to directly study the newborn neurons, we are using lentivirusmediated labeling of the progenitor stem cells (using red fluorescent proteins) together with the calcium imaging technique. This experimental strategy now allows us to examine the functional contribution of the newborn cells to the existing network and may reveal the role of newborn neurons in olfactory coding and plasticity.

# Abstract: Glutamatergic excitotoxicity in cerebellar stroke: contribution of genetic background to neurological and histopathological outcomes in amouse model

**Authors:** E. Kovalev, Z. Tal, G. Zimmermann, S. Ben-Ari, H. Soreq, Y. Yarom, S. Shoham

**Affiliations:** ^1^Research department, Herzog Hospital, Jerusalem, Israel; ^2^Neurobiology, Hebrew University, Jerusalem, Israel; ^3^Biological chemistry, Hebrew University, Jerusalem, Israel

Acute stroke to the human cerebellum involves glutamatergic excitotoxicity and causes variable degrees of motor impairment. The present study focuses on the contribution of genetic background to the variability in stroke outcome. Kainate, a glutamate analog, wasmicroinjected to cerebellum in two inbred strains of mice–FVB/N and C57BL. One and 14 days post injection, C57BL mice displayed more severe neurological outcome compared to FVB/N mice. Damage to the Purkinje layer and electrophysiological response of Purkinje cells (PC) to kainate in a slice preparation were similar in both strains. However immunohistochemical staining of calcium binding proteins calbindin and parvalbumin revealed greater morphological pathology in PC axons of C57BL than of FVB/N mice, suggesting that the more severe neurological impairment in C57BL mice was due to greater impairment of axonal output from the cerebellar cortex. Toward understanding axonal pathology in this model, we focused on a structure formed by basket cell fibers around the PC axon initial segment called the “pinceau”. The results indicate that higher basal expression of certain pinceau elements, including the voltage gated potassium channel Kvbeta2 and potassium hyperpolarization-activated channel, HCN1, predict greater resistance to glutamatergic neurotoxicity in the cerebellum.

*Supported by Israeli MOST and German BMBF*.

# Abstract: Characterization of the first anti-Shaker specific toxin fromthe venom of the Israeli scorpion Buthus occitanus israelis

**Authors:** A. Kozminsky-Atias, N. Zilberberg

**Affiliations:** Department of Life Sciences and the Zlotowski Center for Neuroscience, Ben-Gurion University, Israel

Scorpion venoms are a rich source of varies potassium channel blocking peptides. To identify novel toxins with unique selectivities we studied the polypeptide content of the Israeli scorpion Buthus occitanus israelis (Boi). Seventy three novel putative toxins were discovered following analysis of 400 randomly chosen venom gland-derived cDNA clones. Twenty toxins were predicted to block potassium channels. Accordingly, we identified more than 20 distinct peptides from Boi crude venom which are active against different potassium channels, for example, Shaker, Kv1.3 and RomK1. Here we describe the isolation, cloning and genomic organization of BoiTx1, the first toxin from the Israeli scorpion Buthus occitanus israelis. BoiTx1 is a 37 amino acid-long peptide with six conserved cysteines. BoiTx1 shows 82%– 75% homology to members of the alpha-KTx3 toxin family, thus classified as an alpha-KTx3.10. The pharmacological effects of BoiTx1 were studied on various cloned potassium channels expressed in Xenopus laevis oocytes. BoiTx1 inhibited currents through Drosophila Shaker channels with an IC50 value of 3.5 ± 0.5 nM. In contrast to its structurally related toxins, BoiTx1 is at least hundred-fold more potent against the fly, Shaker channel, then towards its mammalian homologs, Kv1.1 and Kv1.3. Thus, BoiTx1 is the first member of the alpha-KTx3 family that preferentially affects insect potassium channels. This finding could lead to better understanding of the structural basis of channel selectivity in potassium channel blocking peptides.

# Abstract: Testosterone improves visual-spatialmemory in hypogonadalmen

**Authors:** E. Kravitz, M. Davidson, A. Gigi, G. Orr, S. Seidman, M. Weiser, A. Biegon

**Affiliations:** ^1^The Joseph Sagol Neuroscience Center, Sheba Medical Center, Israel; ^2^Sackler Faculty of Medicine, Tel Aviv University, Israel; ^3^Department of Psychiatry, Sheba Medical Center, Israel; ^4^Department of Psychiatry, Columbia University, Israel; ^5 ^Brookhaven National laboratory, NY, USA

**Background**

Testosterone, the primary gonadal hormone in males, is associated with some cognitive functions, particularly spatial skills and spatial memory. This hormone may also have neuroprotective properties. We studied the effects of exogenous testosterone on cognitive functioning in middle-aged hypogonadal men with dysthymia (a mild, chronic depressive disorder). Methods: Fifteen hypogonadal men (41–66 years old, mean 49.8, SD 7.3), suffering from dysthymia were enrolled in a randomized, double-blind clinical trial in which they received testosterone 200 mg or matched placebo IM biweekly for seven weeks. Subjects completed a neuropsychological test battery at the time of enrollment and at the end of the double-blind phase. The battery included the Rey auditoryverbal learning test (Rey-AVLT), Rey Osterreich complex figure test (Rey-CFT, tests visual-spatial memory abilities), and digit-symbol coding test (tests visual-motor coordination and attention).

**Results**

Repeated Measures ANOVA showed a significant improvement in the visual-spatial memory abilities of the testosterone treated group (*N* = 8) compared with the placebo group (*N* = 7) in the immediate-recall trial (T2) and delayed-recall trial (T3) of the Rey-CFT. This was exemplified by a significant time by treatment interaction in performance on the T2 and T3 of Rey-CFT (*F*(1, 13) = 5.127, *P* < .05, and *F*(1, 13) = 4.7, *P* < .05, resp.). No such interaction was found in performance on either the auditory-verbal memory test or the digit-symbol coding test. Conclusion: Normal testosterone levels may be important in the maintenance of specific cognitive abilities, contributing to better performance of visual-spatial tasks but not auditory-verbal or attention tasks. These finding have implications for the assessment and treatment of age-related hypogonadism and possibly also Alzheimer's disease, and should be replicated in a larger sample, and in nondepressed men.

*Supported by J. Sagol doctoral fellowship to Efrat Kravitz*.

# Abstract: Modifications and adaptations of arm use in Octopus vulgaris

**Authors:** M. J. Kuba, R. A. Byrne, T. Gutnick, L. Zullo, K. E. Thonhauser, B. Hochner

**Affiliations:** ^1^Department of Neurobiology & ICNC, Hebrew University, Israel; ^2^Institute of Zoology, Karl-Franzens-University, Graz, Austria; ^3^Department of Neuroscience and Brain Technologies, The Italian Institute of Technology, Italy

The motor control of the highly flexible eight arms of the common octopus (Octopus vulgaris) has been the focus of several recent studies. In our present studies we investigate the ability of the octopuses motor output programs to adapt to new tasks. To do so we forced our animals to perform tasks challenging their standard repertoire of arm movements. We conducted a set of experiments that effected either, bend propagation and fetching movements, or required on-line central control of searching movements and an establishment of learning processes. In our first experiment we introduced a physical constrain to the base of the octopus arm. Animals were placed inside a transparent Perspex box (40 × 40 × 40 cm) with a hole at the center of every surface that allowed the insertion of a single arm only (1.5 cm×). During the experiment the subjects had to reach out through a hole to retrieve a food reward offered outside the box. The accuracy of the reaching towards a target movement did not improve in consecutive experimental sessions. However, the accuracy and speed of fetching movements improved both within and across sessions. A second set of experiments investigated the ability of octopuses to learn to turn their arm in a specific direction in an opaque Y shaped maze. The animals received neither chemical nor tactile information on the direction of the turn. Therefore the correct decision to turn left or right inside the maze could only be made based on proprioceptive information on the position of the arm. 4 out of 6 subjects were able to successfully complete this task in less then 90 trials. Further experiments investigated if the octopus can turn its arm inside a maze according to visual feedback. 6 out of 9 animals learned to direct the movement of their arms inside a three ways choice maze based on visual information. These experiments show that octopuses are able to modify the execution of motor primitives as well as more searching and crawling related movements.

*Supported by Israel Science Foundation. M. Kuba was supported by a Golda Meir fellowship*.

# Abstract: Role of the 18 kDa Translocator protein and the adenine nucleotide transporter in erucylphosphohomocholine-induced cell death in human glioma cells

**Authors:** W. Kugler, L. Veenman, M. Gavish, M. Lakomek

**Affiliations:** ^1^Universitäts-Kinderklinik, D-37075 Gättingen, Germany; ^2^Technion-Israel Institute of Technology, 31096 Haifa, Israel

The 18 kDa Translocator protein (TSPO), formerly called peripheral-type benzodiazepine receptor (PBR), exerts various cell functions and is involved in a functional structure designated as the mitochondrial permeability transition pore (MPTP). Our previous studies indicated that TSPO may be an important component in the MPTP related pro-apoptotic mechanisms activated by the novel membrane-seeking antineoplastic agent erucylphosphohomocholine (ErPC3). Thus, we decided to use two well-known TSPO ligands, PK 11195 and Ro5 4864, to study their interference with ErPC3's proapoptotic activity. Surprisingly, PK 11195 and Ro5 4864 inhibited apoptosis induction by ErPC3 in a concentrationdependent manner. In addition, they appeared to block cytochrome c release, and caspase-9 and -3 activation typically induced by ErPC3. This indicates that the MPTP and TSPO take part in the activation of the mitochondrial apoptosis pathway by ErPC3. TSPO molecules are often found in conjunction with VDAC and ANT, which are suggested core components of theMPTP. ANT appears as a bi-functional protein: an ADP/ATP translocator and a lethal pore regulated by multiple apoptosis regulators. To study in more detail the mechanisms whereby ErPC3 may affectmitochondrial functions, for example, membrane potential, ATP production, we analysed in a first attempt a possible correlation between ErPC3, TSPO, and ANT by investigating cellular ATP levels using human glioblastoma cell lines U87MG and U118MG. Treatment of these cells with TSPO ligands and ErPC3 affected ATP levels. Depending on the cell type, PK 11195 and Ro5 4864 enhanced or reduced ATP levels, whereas ErPC3 reduced cellular ATP levels in all cell lines tested. Interestingly, co-administration of these TSPO ligands with ErPC3 restored cellular ATP levels. Together, these results suggest that not only TSPO but also ANT is involved in ErPC3's action.

*Supported by the Volkswagen-Stiftung (Joint Lower Saxony- Israeli Research Projects; VWZN2047)*.

# Abstract: Shaping of tuning properties inmotor cortex during Long term visuoMotor learning

**Authors:** H. Lalazar, M. Shahar, E. Vaadia

**Affiliations:** ^1^Interdisciplinary Center for Neural Computation The Hebrew University of Jerusalem, Israel; ^2^Department of Physiology Hadassah Medical School The Hebrew University of Jerusalem, Israel

Many studies have examined the relation between various movement parameters and the neural responses in the motor cortex of behaving primates. Encoding for many movement parameters have been shown, such as speed and direction, muscle activations, joint angles, and the sensory cues for movement. In such studies the primates are “overtrained”— trained for many months or years until they reach expert and constant performance. In the current study we recorded from the primary motor cortex of a monkey for several months as she learned a three-dimensional point-to-point reaching task. During this period her behavioral performance improved from a novice level to highly proficient. We recorded the full arm kinematics (using optic sensors), and 96 neural signals from a chronically implanted electrode array. Our task involved a wide variety of movements in order to span a large portion of the movement space and thus allow us to sample the tuning properties in a broad manner. We examined the changes in the encoding of these motor parameters in relation to the parameters of the enhanced movements. In some cases we could track the encoding of a single neuron for several days, as they remained convincingly well isolated and identifiable.

# Abstract: Neural correlates of subjective and objective awareness: An ERP study

**Authors:** D. Lamy, M. Salti, Y. Bar-Haim

**Affiliations:** Tel Aviv University, Israel

The study of the neural correlates of consciousness (NCC) has typically focused on a subjective definition of awareness. Using a backwardmasking paradigm, we dissociated between objective awareness and subjective awareness by having participants first produce a forced-choice response to the location of a liminal target, and then report on their subjective awareness of the target presence. We recorded event-related brain potentials (ERPs) and compared neural activity when observers reported being aware versus unaware of the target but localized it correctly, thereby isolating the neural correlates of subjective awareness while controlling for differences in objective performance. In addition, we compared neural activity when participants were subjectively unaware of the target presence and localized it correctly versus incorrectly, thereby isolating the neural correlates of objective awareness. Importantly, all conditions involved stimuli that were physically identical and were presented for the same duration. The results show that the amplitude of the P3 component correlates with the degree to which the target is consciously perceived

# Abstract: Different effects of voluntary and involuntary attention on EEG activity in the gamma band

**Authors:** A. Landau, M. Esterman, L. Robertson, S. Bentin, W. Prinzmental

**Affiliations:** ^1^University of California, Berkeley, Calif, USA; ^2^Hebrew University, Jerusalem, Israel

Recent studies in animals and humans have shown that EEG activity in the gamma range can be modulated by attention. In the present study we compared this activity for voluntary and involuntary allocation of spatial attention in a spatialcuing paradigm with faces as targets. The visual stimuli and trial timing were kept constant across attention conditions with only the predictive value of the cue changing. Gammaband response was linked to voluntary shifts of attention but not to the involuntary capture of attention. The presence of increased gamma responses for the voluntary allocation of attention, and its absence in cases of involuntary capture, suggests that the neural mechanisms governing these two types of attention are different. Moreover, these data allow a description of the temporal dynamics contributing to the dissociation between voluntary and involuntary attention. The scalp distribution of this correlate of voluntary attention is consistent with a top-down process involving primarily contralateral anterior and posterior regions.

# Abstract: Predicting pleasantness of binary mixtures in olfaction

**Authors:** H. Lapid, D. Harel, N. Sobel

**Affiliations:** ^1^Department of Neurobiology, Weizmann Institute of Science, Israel; ^2^Department of Computer Science and Applied Mathematics, Weizmann Institute of Science, Israel

Although the rules underlying the perceived intensity of binary mixtures has been investigated, only minimal efforts have been directed at elucidating the rules underlying the perceived pleasantness of such mixtures. To address this, 84 subjects ranked the relative and absolute pleasantness of 4 distinct binary mixtures (15 pairs, ISI = 4 s, ITI = 30 s, flow = 6 l/min, pulse = 2 sec) made of different mixing ratios (0:100%, 25:75%, 50:50%, 75:25% and 100:0%, olfactometer generated vapor phase) and of the separated constituents of the mixtures diluted with clean air to the overall air flow of the mixture. 3 mixtures consisted a relatively pleasant odorant mixed with an unpleasant one (L-Carvone- IsoValericAcid, Linalool-ValericAcid, PhenylethylAlcohol- ButanoicAcid) and one mixture consisted of two unpleasant odorants (IsoValericAcid-ValericAcid). Each trial was repeated twice with odorant order counter-balanced and trial order randomized. The mixture's separated constituents were in turn ranked for their intensity on a VAS scale. Based on these results, we propose here a novel prediction model for the pleasantness of binary mixtures from the pleasantness of their separated components at different mixing concentrations and their respective intensities. This model does not require presetting of an interaction constant between the mixture components, nor does it require any factorization of the pleasantness weights. It does, nonetheless, require solid psychophysical data of the separated components at their different concentrations, and currently it can only explain the behavior of intermediate pleasantness of the mixture.

# Abstract: Reconstructing the olivo-cerebellar systemin a dish

**Authors:** Y. Lefler, M. Belenky, Y. Yarom

**Affiliations:** ^1^Department of Neurobiology and The ICNC, The Hebrew University of Jerusalem, Israel; ^2^Department of Cell and Animal Biology, The Hebrew University of Jerusalem, Israel

In order to unravel the functional organization of the olivocerebellar loop, one needs a simultaneous access to all its components. Since it is rather impossible to achieve this in in-vivo conditions, we are developing a culture system that captures the entire circuit, which is based on brain slices of 7 to 18-day-old rats. Once isolating the brain, the cerebellum and the attached brain stem were sliced into 250– 500 *μ*M-thick slices, in a plain that ought to preserve the inferior olive, the deep cerebellar nuclei and a parasagittal portion of the cerebellum. Slices were then placed on membranes of 30-mm culture inserts with 0.4 *μ*M pore size (Millicell, Millipore) and cultured over culture medium containing 50% basal medium, 25% Hanks'balanced salt solution, 25% horse serum, L-glutamine, antibiotics and glucose. Slices were maintained in culture for 1–6 weeks at 37°C with 5% CO_2_ enriched atmosphere. Whole cell patch-clamp recordings from Purkinje cells (PCs) were made after removing the inserts'wall and placing it in a recording chamber perfuse with 5% CO_2_ and 95% O_2_ enriched Ringer's solution. Our results show that these cultures form an active network of cerebellar neurons. PCs preserved their characteristic two-dimensional dendritic tree, and displayed suband supra-threshold spontaneous activity. The subthreshold activity consisted of depolarizing and hyperpolarizing potentials that respectively decreased and increased in amplitude upon depolarization, which suggests that they represents chemical synaptic inputs. Supra-threshold activity, which was often observed, was manifested as overshooting action potentials that were carried by Na^+^ and Ca^++^ currents. Since PCs retain their physiological and morphological characteristics, it is reasonable to assume that this organotypic culture will pave the way for reconstruction of the olivocerebellar loop in a dish, which will allow for simultaneous recording from all the major components of the system.

# Abstract: Collinear facilitation at the periphery: does it differ from the fovea?

**Authors:** M. Lev, U. Polat

**Affiliations:** Sackler Faculty of Medicine, Goldschleger Eye Research Institute, Tel Aviv University, Tel Hashomer, Israel

Collinear facilitation is a common phenomenon, but it has been challenged recently at the human periphery. We probed the facilitation using a Yes/No detection task by measuring the false-positive reports (false-alarm, Pfa) and hit rate (Phit) for a low-contrast Gabor target (between two flankers) that appeared randomly at the fovea or at the periphery (2.4 deg) to the right or left side. We compared three target-flanker configurations: orthogonal at a distance of 15-lambada (target alone), and collinear or orthogonal at 5-lambada. The report for the target present was high (Phit, Pfa) for the collinear but not for the other configurations. The overall increase in the target-present responses only for the collinear configuration is consistent with the excitatory range of interactions. However, the sensitivity of the collinear configuration at 5-lambada is higher than the target but only slightly higher than the orthogonal configuration. Therefore, the usual expression of a facilitation effect as a sensitivity measure is distorted by the strong excitation (Polat & Sagi, 2006). The existence of a similar pattern of higher Phit and Pfa for the collinear configuration at the fovea and periphery suggests that collinear facilitation is a common phenomenon that affects both the periphery and fovea.

*Supported by the Israel Science Foundation and the National Institute for Psychobiology in Israel*.

# Abstract: Gender dependent behavioral effects of perinatal gamma-aminobutyric acid (GABA) potentiation and methylenetetrahydrofolate reductase (MTHFR) deficiency inmice

**Authors:** T. Levav, E. Blumkin, H. Golan

**Affiliations:** Department of Developmental Genetics, Ben-Gurion University, Zlotowski Center for Neuroscience, Israel

Fetal-Anticonvulsant Syndrome may cause developmental delay, cognitive dysfunction, and behavioral impairments: hyperactivity, attention deficit disorder and autistic-like behavior. We examined the interactions between genetic susceptibility and anticonvulsant drug treatment, by performing behavioral phenotyping of MTHFR deficient offspring perinatally treated with a GABA potentiating substance. Four groups of offspring were compared: Balb/c wildtype (wt) and Mthfr−/+ mice injected daily subcutaneously on postnatal days 4–10 with saline (Ct) or vigabatrin (GVG; 50 mg/kg). GVG treatment, but not MTHFR, delayed motor and sensory reflex development. In the open field task, adult male GVG treated mice demonstrated increased mobility, regardless of genetic background. Hyperactivity was expressed in a longer distance moved by GVG-wt and GVGMthfr−/+ mice (1255.6 and 1363.2 cm) and a higher velocity (4.2 and 4.5 cm/s), compared with Ct-wt mice (965.1 cm and 3.2 cm/s, resp.; *P* < .05). In adult female mice no significant alterations in mobility were observed. Anxiety was reduced in male GVG-wt and GVG-Mthfr−/+ mice, expressed by a higher duration ratio center/margin, compared to Ctwt mice (0.35 and 0.40 versus 0.18, resp.; *P* < .05). In female mice, anxiety was reduced in GVG-wt and Ct-Mthfr−/+, compared to Ct-wt, and MTHFR deficiency combined with GVG treatment abolished the anxiety related effect. Recognition memory was impaired, regardless of gender, in both GVG-wt and GVG-Mthfr−/+ mice, as well as in Ct-Mthfr−/+ mice. These mice did not distinguish between novel and familiar objects. In a sociability task, a genotype effect was observed in male and female. This genotype effect was not observed in a social preference test, while in female GVG treatment in wt and Mthfr−/+ mice reduced preference to an unfamiliar mouse (*P* < .05) Overall, MTHFR deficiency and GABA potentiation differentially affects a spectrumof behaviors in male and female mice.

*Supported by Eshkol Fellowship, Israeli Ministry of Science, Culture and Sport, for Tamar Levav*.

# Abstract: Characterization of the IMPA1 deficient mouse

**Authors:** I. Levi, A. Shamir, K. Cryns, T. Steckler, D. Moechars, R. Belmaker, G. Agam

**Affiliations:** ^1^Department of Clinical Biochemistry, Faculty of Health Sciences, Ben Gurion University of the Negev, Israel; ^2^Psychiatry Research Unit, Faculty of Health sciences, Ben Gurion University and Mental Health Center, Israel; ^3^Research and Early Development Europe, Johnson and Johnson Pharmaceutical Research and Development, USA

Lithium has been used to treat bipolar disorder over the last five decades, but the molecular mechanism of its therapeutic effect has not yet been unraveled. We phenotyped IMPA1 knockout mice to study the possibility that the product of this gene, the enzyme inositol monophosphatase, is a molecular target in mediating lithium dependant physiological effects. The IMPA1−/− mice die in utero between day 9.5 and 10.5 post coitum (p.c.) demonstrating the importance of IMPA1 in early embryonic development. The embryonic lethality could be reversed by myoinositol supplementation via the pregnant mothers. In the adult's IMPA1−/− brain IMPase activity levels were found to be strongly reduced (up to 65% in hippocampus). However, inositol levels were not found to be changed. Rescued adult IMPA1−/− mice exhibit a strongly increased sensitivity to pilocarpine-induced seizures, supporting the idea that IMPA1 represents a physiologically relevant target of lithium. The IMPA1−/− mouse represents a novel model to study inositol homeostasis, and indicates that genetic inactivation of IMPA1 can mimic some lithium behavioral effects.

# Abstract: Emotion recognition vs. non-effective visual processing deficits in schizophrenia

**Authors:** R. Levin, S. Elran, M. Ermilov, S. Edelman, D. C. Javitt, U. Heresco-Levy

**Affiliations:** ^1^Psychiatry Department, Ezrath Nashim-Herzog Memorial Hospital, Jerusalem, Israel; ^2^Hadassah Medical School, Hebrew University, Jerusalem, Israel; ^3^Nathan S. Kline Institute for Psychiatric Research and NYU School of Medicine, NY, USA

Deficits in face emotion identification and discrimination were previously reported in schizophrenia. However, it is not clear whether the impairment in decoding emotion based on facial expression represents a specific dysfunction domain or may reflect generalized visual processing deficits. In the present study, we evaluated both emotion recognition and generalized, nonaffective visual processing parameters (GVP) and their relationship with clinical state in schizophrenia patients. Thirty stable schizophrenia inpatients and 15 age- and sex-matched control subjects participated in the study. Decoding emotion processing assessed by (1) the face emotion identification test (FACE-ID); (2) the face emotion discrimination test (FACE-DIS). GVP was assessed using (1) the closure flexibility-concealed figure task (CFT); (2) the perceptual organization (PO). For all patients, the positive and negative syndrome scale (PANSS) was administered. Patients showed significantly impaired performance on FACE-DIS, although between-group differences in FACE-ID were not statistical significance. Large effectsize between-group differences were also observed on both GVP tasks. A MANOVA analysis including all four test measures simultaneously indicated both a main effect of group (*F* = 29.1, *df* = 1, 40, *P* < .0001) and a significant (*F* = 7.91, *df* = 3, 38, *P* < .0001) group x test effect. Post-hoc ttests indicated the existence of a greater deficit among patients on the nonaffective relative to affective visual processing tests used in this study. Significant positive correlations were found among patients between impaired FACEDIS and FACE-ID performance and the GVP measures. Positive, negative and total PANSS scores correlated significantly with FACE-DIS (*r* = −0.48, *P* < .05) and FACE-ID (*r* = −0.65, *P* < .01) performance. These findings indicate that in schizophrenia, basic visual processing deficits may contribute to the impaired ability to decode emotions based on facial expression.

# Abstract: The importance of being stable and balanced-a therapy inspired network approach

**Authors:** N. Levit-Binnun, E. Moses

**Affiliations:** Department of Physics of Complex Systems, TheWeizmann Institute of Science, Rehovot, Israel

We combine recent understanding obtained on the activation of networks in the brain with experience of therapists and clinicians dealing with developmental disorders, to propose a novel paradigm that can explain several aspects of behavior and development. The major input from the clinic is in finding the efficient modes of intervention and therapy methods that enable the re-shaping of behavior. This allows us to identify susceptible or weak parts of the network that, when functioning normally, underlie the development of a purposeful self. Their abnormal functioning, on the other hand, seems to lead to disturbed human experiences such as autism and schizophrenia. A careful interpretation of the most up-todate clinical work reveals the specific components and the underlying dynamics of a fundamental network that are required for normal development. These components include the sensory, emotion and motor systems, each of which has a critical influence on the functionality of the other two components and on the recruitment of other important functions (such as memory and motivation). The dynamic notions that we find as crucial for the functioning of the network are balance and stability. Balance refers to the functioning of the network and its components within a range of parameters that enable good functionality. Stability assures that fluctuations in the input and output of the network are not prohibitively large. Severe instability may drive the development away from its normal route (as in autism), and may even lead to network collapse (as in schizophrenia). Regaining stability and balance is therefore the goal of a successful intervention.

# Abstract: Biochemical and behavioral effects of triiodothyronine (T3) may provide a basis for the antidepressant attributes this compound

**Authors:** T. Lifschytz, G. Shalom, T. Golzer, R. H. Segman, B. Lerer, M. E. Newman

**Affiliations:** Biological Psychiatry Laboratory, Hadassah Hebrew University Medical Center, Jerusalem, Israel

Thyroid hormones had been long used in the treatment of depression, mostly as supplements to antidepressants, to enhance or accelerate clinical response. Little is known of the mechanisms underlying these effects, which we attempted to uncover by using different pharmacological, behavioral and molecular paradigms. In vivo microdialysis was used to evaluate the effects of fluoxetine 5 mg/kg, T3 20 mcg/kg and their combination, administered to male Sabra rats for 7 day, on serotonin levels and the activity of presynaptic serotonergic receptors in the cortex and hypothalamus. Reduction of hypothalamic serotonin levels was observed in rats treated with T3 alone or combined with fluoxetine. Reduction in the activity of 5HT1b autoreceptors was observed in the hypothalamus of rats receiving T3 plus fluoxetine, but not in those receiving either compound alone. The novelty suppressed feeding test was used to explore the effects of fluoxetine 10 mg/kg, T3 20 mcg/kg and the combination of fluoxetine 5 mg/kg+ T3 20 mcg/kg, for 12days, on the time required for a food deprived animal to commence eating in an unfamiliar environment (latency). Only the fluoxetine 5 mg/kg+ T3 20 mcg/kg combination caused a significant shortening of latency. Finally, we studied the effects of fluoxetine 5 mg/kg, T3 20 mcg/kg and their combination, administered for 7 days, on mRNA levels of 5HT1a and 5HT1b receptors in different brain areas. In the raphe nucleus the combination of T3+ fluoxetine caused a decrease in mRNA for both receptors. Similar reductions were observed in rats treated with T3 alone or combined with fluoxetine in the amygdala, CA1 and dentate gyrus (5HT1a receptor mRNA) and the frontal and enthorinal cortices (5HT1b receptormRNA). Taken together, the results of these different experimental paradigms suggest that the mechanisms underlying the antidepressant activity of T3 may be mediated by changes in the activity of presynaptic serotonergic autoreceptors.

# Abstract: Regional sensitivity to neuroinflammation: in vivo and in vitro studies

**Authors:** S. Liraz-Zaltsman, I. Fishbein, V. Greeneberger, E. Shohami, A. Biegon

**Affiliations:** ^1^The Joseph Sagol Neuroscience Center, Sheba Medical Center, Israel; ^2^The Hebrew university Department of pharmacology, Jerusalem, Israel; ^3^Weizmann institute of science, Rehovot, Israel; ^4^Brookhaven National laboratory, USA

Neuroinflammation is believed to play a major role in several acute human neuruopathologies and chronic conditions such as Alzheimer's and Parkinson's disease. Recent observations suggest that the intensity of the neuroinflammatory response to various challenges is region dependent. The goal of our study is to expand and substantiate our knowledge of regional sensitivity to neuroinflammation and explore the underlying mechanisms. Neuroinflammation was induced in vivo by intracisternal (ic) injections of endotoxin (LPS) in male Sabra mice. Animals were tested for neurological and cognitive deficits using a neurological severity score (NSS) and the novel object recognition test (ORT). Regional neuroinflammation was measured by quantitative autoradiography with [3H] PK11195 a peripheral benzodiazepine receptor ligand that marks the activation of Microglia. Quantitative analysis of neuroinflammation was performed in five brain areas. In vitro, we employed mixed (e.g., cortex/ striatum) neuronal cultures exposed to glutamate and/or TNFalpha, for 24 h, with neuronal survival as an endpoint. ic LPS Mice showed moderate neurological deficits and significant deficits in the ORT. Increased [3H] PK11195 density was found in all regions, with the highest increase (26%, *P* < .0001) in entorhinal cortex, followed by perirhinal, temporal and cingulate cortex (*P* < .05). The striatum had the smallest increase (13%, NS). In vitro, we found a significant survival difference between cortical and striatal neurons exposed to glutamate (*P* = .01) and TNFalpha (*P* = .002). Exposure to glutamate and TNFalpha reduced the number of cortical neurons to 5.68%+/−4.3 74.55%+/−18.6 of control respectively while the number of striatal neurons was reduced to 21.04%+/−14.09 and 115.69%+/−25.01 respectively. A better understanding of the regional determinants governing sensitivity to neuroinflammation may help in identifying new targets for treatment or prevention of neuroinflammatory damage in human disease

# Abstract: Simultaneous MEG and subthalamic local field potential recordings in Parkinson patients reveal the frequency-specific spatial pattern and directionality of subthalamo-cortical interactions

**Authors:** V. Litvak, W. Penny, A. Eusebio, C. C. Chen, R. Oostenveld, J. Kilner, P. Limousin, L. Zrinzo, M. Hariz, K. Friston, P. Brown

**Affiliations:** ^1^Institute of Neurology, University College London, London, UK; ^2^F.C. Donders Centre for Cognitive Neuroimaging, University of Nijmegen, Nijmegen, The Netherlands

Insight into how brain structures interact is critical for understanding the principles of brain function and may lead to better diagnosis and therapy. To study interactions between the cortex and deep brain structures (basal ganglia and the thalamus) we recorded simultaneously local field potentials (LFPs) from deep brain stimulation (DBS) electrodes and magnetoencephalographic (MEG) signals from the cerebral cortex (CTF 274 channel system) in Parkinson's disease (PD) patients with bilateral DBS electrodes in the subthalamic nucleus (STN). We report the results from two patients. Information transfer between the STN and cortex was evaluated using a multivariate autoregressive (MAR) model in combination with whole-head topographical mapping and source localization. In case 1 (male, 38 y.o., left-handed, 10 years post PD onset) the right STN LFP predicted the MEG signal over the right sensorimotor area and the MEG fromthe right sensorimotor and mesial areas predicted the right STN LFP. Similar bidirectional interactions were observed for left STN with the signals predicted by STN LFP more focal over the left sensorimotor area and the signals predicting STN activity found over bilateral sensorimotor cortex. In case 2 (male, 54 years old, right-handed, 18 years post PD onset) bidirectional interactions were observed between the right STN and mesial areas. The left electrode in case 2 passed about 2 mm postero-medial to the sensorimotor area of the STN. No coupling was detected between this left channel and MEG; confirming the regional specificity of the cortico-STN coupling. We conclude that simultaneous recording of the LFP from DBS electrodes and MEG enables one to establish patterns of coupling between cortex and focal subcortical structures. In the case of the STN, this coupling is clearly bidirectional, in keeping with its cortical input via the corticostriatal and hyper-direct pathways, and output back to cortex via globus pallidus and thalamus.

*Supported by Marie Curie Intra European fellowship (contract MEIF-CT-2006-038858), British Technion Society*.

# Abstract: Synaptogenesis of adult-born neurons in themouse olfactory bulb

**Authors:** Y. Livneh, G. Raday, A. Mizrahi

**Affiliations:** Department of Neurobiol & ICNC, The Hebrew University of Jerusalem, Israel

Throughout adulthood, the olfactory bulb (OB) continuously receives newborn neurons that integrate into the network as functional interneurons. This unique population serves as a model system to study how synapses are formed during neuronal integration into a preexisting network. Here, we set out to directly image newborn neurons and their underlying synapses by combining in vivo imaging and electronmicroscopic (EM) analysis. Adult-born neurons were transduced at the subventricular zone with lentiviruses to express a postsynaptic density protein fused with green fluorescent protein (PSD95-GFP) revealing both dendrites and synapses. We first carried out an EM analysis of the PSD95-GFP puncta. To this end, we analyzed young developing neurons 10-14 days post injection (DPI) where only 63% of PSD95-GFP puncta had a clear pre-synaptic partner. This indicates that 37% of the synapses are still developing. Notably, newborn neurons made synapses almost exclusively with axons of olfactory receptor neurons. Second, we analyzed synaptic distributions and dynamics of whole neurons. At 10–14 DPI, newborn puncta were sparsely distributed along dendrites (up to 20 microns between synaptic puncta). In marked contrast, puncta of mature neurons (at 57DPI) were distributed more densely (peak distance– 3 *μ*m between puncta). Finally, in vivo two-photon timelapse imaging of newborn neurons over 36 hours revealed that synaptic puncta display a rich repertoire of dynamics, continuously appearing and disappearing. For example, only ∼60% of synaptic puncta remained stable through the imaging session. Taken together, these experiments provide an experimental “snapshot” into the dynamic behavior of synapse formation in the intact mammalian brain.

# Abstract: Variable Processing of Texture Signals by Whisker Vibrations

**Authors:** E. Lottem, R. Azouz

**Affiliations:** Department of Physiology, Faculty of Health Sciences, Ben Gurion University of the Negev, Israel

Rodents in their natural environment use their whiskers to distinguish between surfaces having subtly different textures and shapes. They do so by actively sweeping their whiskers across surfaces in a rhythmic motion. To determine how textures are transformed into vibration signals in whiskers and how these vibrations are expressed in neuronal discharge patterns, we induced active whisking in anaesthetized rats, monitored the movement of whiskers across surfaces, and concurrently recorded from trigeminal ganglion (TG) neurons. We found that whisker movements across surfaces are composed of whisking and texture-related vibration signals, both carrying information about surface roughness. During contact with textures, TG neurons presented a broad range of responses, which encoded the two signals. To determine whether these signals can support texture discrimination we examined their dependence on surface roughness. We found that despite a large variability in this translation process, different textures are translated into distinct vibrations profiles. These response profiles vary across whiskers, are dependent on the radial distance of the textures, on whisking frequency, and may be influenced by head movements. Using the characteristics of these signals, we employed discriminant analysis to statistically evaluate the ability of whiskers to discriminate between textures. Texture identity was correctly classified in 87% of whisks. This classification did not depend on whisker identity and whisking frequency, while deteriorating with radial distance. Finally, increasing the number of whisks and integration of information from multiple whiskers improved texture discrimination. These results indicate that surface roughness is translated into distinct whisker vibration signals that result in neuronal discharge pattern.

# Abstract: Chronic inhibition of brain cytochrome oxidase in rats inducesmemory deficits and associated with oxidative stress and alterations in cholinergic transmission

**Authors:** L. Luques, S. Shoham, M. Weinstock

**Affiliations:** ^1^Department of Pharmacology School of Pharmacy Hebrew University of Jerusalem, Israel; ^2^Research Department Herzog Hospital, Jerusalem, Israel

The present study tests the hypothesis that in Alzheimer's disease early reduction in cytochrome oxidase (COx) activity could initiate a cascade of events which includes oxidative stress (OS) leading to cognitive impairment via involvement of cholinergic neurons. Reduction in COx and memory impairment can be mimicked in rats by chronic administration of sodium azide (NaN3).

**Methods**

NaN3 or saline was given s.c. for 4 weeks (1mg/kg/hr) by minipumps to male Sprague-Dawley rats. After 2 or 5 weeks, brains were fixed and sectioned for immunohistochemical mapping of the marker of OS, manganese-dependent superoxide dismutase (Mn-SOD), and markers of cholinergic neurons, choline acetyltransferase (ChAT) and of vesicular acetylcholine transporter (VAChT).

**Results**

NaN3–treated rats showed a significant decrease in COx activity in the cingulate and parietal cortex and in the hippocampus. At 2 weeks, NaN3 induced a significant increase inMn-SOD (indicating the presence of OS) in the basal forebrain including diagonal band, medial septum, (the major sources of cholinergic input to the hippocampus and cingulate cortex) and in the hippocampus. At 5 weeks, there was a reduction of 20% in mean size of cholinergic neurons in the diagonal band, suggesting that these neurons were under stress although cholinergic neuron loss was not observed. There was a compensatory increase of about 100% in immunoreactive varicosities and GAP-43 (a presynaptic membrane phosphoprotein marker of plasticity) in the subgranular layer of the dentate gyrus.

**Conclusions**

The present study demonstrates a unique sensitivity of certain neuronal populations, among them, the septohippocampal system, to reduction in cytochrome oxidase activity. The present study also suggests that oxidative stress is involved in this process.

# Abstract: Attentional demands predict short-term memory load response in posterior parietal cortex

**Authors:** H. Magen, S. Kastner, A. Treisman

**Affiliations:** ^1^Psychology Department, the Hebrew University, Israel; ^2^Psychology Department, Princeton University, USA

The ability to maintain information for short periods of time plays an essential role in cognition. One of the most intriguing aspects of short-term memory is its limited capacity, typically around 3 or 4 items. Functional magnetic resonance imaging (fMRI) research revealed a large network of brain areas involved in the short-term retention of information. Recently it has been suggested that activity in a specific part of this network, the posterior parietal cortex (PPC), is correlated with the behavioral estimates of capacity limitations and might serve as a capacity-limited store (Todd & Marois, 2004). We present the results of a set of experiments that failed to find this close correlation between fMRI activity and behavioral capacity limitations. Instead, we show that when visual rehearsal is more prominent in the task, fMRI activity in PPC increase with memory load beyond the behaviorally determined limits of capacity. We suggest that activity in PPC reflects the attentional demands of the short-term memory task rather than a capacity limited store. This interpretation is consistent with the role of PPC in attentional processes and with the close correlation between brain areas that are involved in attention and those that mediate short-term memory.

# Abstract: Cannabidiol (CBD) ameliorates cognitive impairments associated with amodel of chronic liver disease in mice

**Authors:** I. Magen, Y. Avraham, E. Berry, Z. Ackerman, R. Mechoulam

**Affiliations:** ^1^Department of Human Nutrition and Metabolism, Braun's School of Public Health, Hebrew University, Israel; ^2^Department of Internal Medicine, Hadassah–Hebrew University Medical Center, Mount Scopus, Israel; ^3^Department of Medicinal Chemistry and Natural Products, Medical Faculty, Hebrew University, Israel

**Background**

Hepatic encephalopathy (HE) is a major neuropsychiatric complication of both acute and chronic liver failure. However, its pathogenesis is still unknown. It has been suggested that the cognitive deficits characterizing this state result, at least in part, from an inflammatory response in the brain. Ca nnabidiol (CBD) is an active compound of the plant Cannabis Sativa known for its antiinflammatory properties. We hypothesized that CBD may have therapeutic potential in chronic liver disease through antiinflammatory actions. Methods: Female Sabra mice were subjected to ligation of the bile duct (BDL). Sham operated animals were used as controls. 2 weeks and 3 weeks post-surgery, animals receiving either vehicle or 5mg/kg CBD were evaluated for cognitive function in the Eight Arm Maze and the T-maze tests. The animals were sacrificed and their hippocampi were analyzed for mRNA levels of IL-1 beta by RT-PCR analysis, and their livers were analyzed for MDA levels, indicating oxidative stress by the TBARS method. Results: IL-1 beta expression in the hippocampus increased significantly in BDL mice 3 weeks post-surgery, and was restored to normal values by CBD. Oxidative stress in the liver increased in BDL animals and was decreased by CBD. Cognitive function was significantly impaired in BDL mice and these impairments were also ameliorated by CBD. Conclusion: These results indicate an involvement of inflammatory processes in the hippocampus in the pathogenesis of HE. This inflammatory response may be associated with the behavioral impairments, and both were ameliorated by CBD. CBD may act via the adenosine system, since an upregulation of this system was observed in hippocampal slices of human HE patients, post mortem.

# Abstract: The role of intrinsically-disordered protein segments in mediating Kv channel clustering: implications for synapse assembly, maintenance and function

**Authors:** H. Magidovich, I. Orr, D. Fass, U. Abdu, O. Yifrach

**Affiliations:** ^1^Department of Life Sciences, Ben-Gurion University of the Negev, Zlotowski Center for Neurosciences, Israel; ^2^Weizmann Institute of Science, Israel

The interaction of membrane-embedded voltage-activated potassium channels (Kv) with intracellular scaffold proteins, such as the post-synaptic density 95 (PSD-95) protein, is mediated by the channel C-terminal segment. This interaction underlies Kv channel clustering at unique membrane sites and is important for the proper assembly and functioning of the synapse. In the current study, we address the molecular mechanism underlying Kv/PSD-95 interaction. We provide experimental evidence, based on hydrodynamic and spectroscopic analyses, indicating that the isolated C-terminal segment of the archetypical Shaker Kv channel (ShB-C) is a random coil, suggesting that ShB-C belongs to the recentlydefined class of intrinsically disordered proteins. We show that isolated ShB-C is still able to bind its scaffold protein partner and support protein clustering in vivo, indicating that unfoldedness is compatible with ShB-C activity. Pulldown experiments involving C-terminal chains differing in flexibility or length further demonstrate that intrinsic disorder in the C-terminal segment of the Shaker channel modulates its interaction with the PSD-95 protein. Our results thus suggest that the C-terminal domain of the Shaker Kv channel behaves as an entropic chain and support a “fishing rod” molecular mechanism for Kv channel binding to scaffold proteins. The importance of intrinsically disordered protein segments to the complex processes of synapse assembly, maintenance, and function is discussed.

# Abstract: Towards behavioural measures of action space–manipulability vs. compatibility in objects affordances

**Authors:** T. Makin, M. Vax, E. Zohary

**Affiliations:** ^1^Neurobiology Department, Life Science Institute, Israel; ^2^Interdisciplinary Center for Neural Computation, Hebrew University of Jerusalem, Israel

We perceive in order to act on the environment. Affordance theory states that objects are perceived not only in terms of shapes and spatial relationships but also in terms of possibilities for action (affordances). That is, perception drives action in a direct and immediate way. According to this assertion, seen objects should automatically potentiate components of the actions they afford. Previous studies have demonstrated affordance by showing nonspatial compatibility effects for central objects, based on the orientation of their handles. However, it is possible that the visual asymmetry created by the handles directed the subjects' attention towards one side, thus creating spatial compatibility effects, irrespective of any affordances. In order to dissociate between affordance and spatial compatibility effects, we tested whether responding to manipulable objects could result in speeded responses, as compared to nonmanipulable objects with similar visual asymmetries. To this end, we presented pictures of manipulable and nonmanipulable objects, half of which contained metal, on the right or left side of the screen. On each trial, subjects were required to judge whether the depicted object containedmetal or not, by pushing a button with their left or right hand. Our results showed significant effects of both spatial compatibility and of manipulability: Subjects responded quickest andmost accurately when manipulable objects were presented compatibly with the responding hand, and slowest when nonmanipulable objects were presented incompatibly with the responding hand. These results suggest that both effectsmay prime responses equally. However, subjects' performance in a separate spatial perception task (landmark) correlated with the effect of compatibility, but not of manipulability, suggesting that whereas the former relies on perception space, the latter might rely more on action space.

# Abstract: Rapidmodulation of motor cortex excitability–TMS evidence for hand-centered visual representations of space

**Authors:** T. Makin, N. P. Holmes, C. Brozzoli, Y. Rossetti, A. Farne

**Affiliations:** ^1^Neurobiology Department, Life Sciences Institute, Hebrew University of Jerusalem, Israel; ^2^Psychology Department, Hebrew University of Jerusalem, Israel; ^3^INSERM, U864, Espace et Action, Bron, France; ^4^Universite Lyon, UMR-S 864, Lyon, France; ^5^Hospices Civils de Lyon, Hopital Neurologique, Mouvement et Handicap, Lyon, France

Perception and action are interdependent and inseparable– an animal's motor behavior should be reflected in its perception. Indeed, electrophysiological studies in premotor cortex have revealed visual receptive fields, selective for moving 3D objects, that shift with the position of a monkey's arm, regardless of gaze direction. This suggests a representation of visual space in hand-centered coordinates. In the present study, we explored hand-centered modulation of visual space in the human motor cortex. Subjects performed a simple “Go” response task to a central visual target, by pushing a button on the left or right side of fixation, while simultaneously a 3D ball approached the left or the right side. Between 40–120 ms later, a single TMS pulse was applied to the primary motor cortex contralateral to the responding finger in order to elicit motor evoked potentials (MEPs). We found that the mean peak- to-peak MEP amplitude was smaller when the ball approached the position of the responding hand, as compared to far from the hand, regardless of the hand position with respect to fixation. We hypothesized that this rapid modulation reflected the suppression of an existing motor plan (the “Go” response), caused by the approaching object. We designed a battery of experiments to test this hypothesis, manipulating the type of visual stimulus presented, the required motor task, and the direction of subject's overt (changing fixation position) and covert visual attention (presenting peripheral cues). We found that the hand-centered modulation of MEP amplitudes was critically dependent upon the timing of the TMS pulse (70– 80 ms), the type of visual stimulus (moving balls versus static LEDs), and the presence of an ongoing motor plan. Manipulating the location of visual attention only partially modulated these hand-centered effects, suggesting contributions of both eye- and hand-centered spatial representations to activity in primary motor cortex.

# Abstract: Watching synapses form: live imaging of the formation of presynaptic boutons by growth cones of cultured aplysia neurons

**Authors:** G. Malkinson, M. E. Spira

**Affiliations:** Department of Neurobiology, The Life Sciences Institute, The Hebrew University of Jerusalem, Israel

Synaptogenesis is a fundamental process during neuronal development, learning processes, and regeneration after trauma. We have recently argued that in cultured Aplysia neurons prefabricated varicosities (VRs) serve as ready-to-go presynaptic compartments. Thus VRs exocytose their vesicular contents in a spike activity-dependent manner. The mechanisms by which VRs are formed are not entirely understood. Real-time confocal imaging of growth cones (GCs) and VRs revealed that VR formation is preceded by accumulation of vesicles and cytoskeletal elements in the central domain of a pausing GC. During the phase of organelleaccumulation, (lasting 8–22 minutes), the actin-rich peripheral domain continues to extend and retract. When a “large” mass of organelles accumulates in the central domain, the GC resumes its net growth by actin-rich lamellipodia and filopodia, invaded by MTs. It is important to note that while the advancing GC does not contain large quantities of organelles, it leaves behind a VR loaded by a mass of organelles. With time (8–25minutes), the splayed MTs within the VR bundle to form the core of the VR. This core is very dynamic and can splay or even bend when the VR moves along the neurite. Imaging of Golgi derived vesicles labeled by synaptopHluorins revealed that the growth process is associated with constitutive fusion of vesicles with the GC's plasma membrane. Evoked exocytosis coupled to membrane retrieval operates in both GCs and VRs. Taken together our observations are consistent with the hypothesis that VRs are formed when the supply of anterogradely transported vesicles exceed the amount needed to support the elongation process, which is limited by the assembly of the actin and MT skeleton. This is also consistent with the finding that VRs are almost homogeneously spaced along individual neurites, which represents the local balance between organelle supply and elongation.

# Abstract: Activity–dependent neuroprotective protein constitutes a novel element in the SWI/SNF chromatin remodeling complex andmodulates neural shape

**Authors:** S. Mandel, I. Gozes

**Affiliations:** Human Molecular Genetics and Biochemistry, Sackler Medical School,Tel Aviv University, Israel

Complete deficiency in activity-dependent neuroprotective protein (ADNP), a multifunctional, heterochromatin 1- binding protein, results in dramatic changes in gene expression, neural tube closure defects and death at gestation day 9 in mice. To further understand the cellular roles played by ADNP, the HEK293 human embryonic kidney cell line was used as a model for the identification of ADNP interacting proteins. Recombinant green fluorescent protein (GFP)-ADNP was localized to cell nuclei. When nuclear extracts were subjected to immunoprecipitation with specific GFP antibodies followed by protein gel electrophoresis, several minor protein bands were observed in addition to GFP-ADNP. In-gel protein digests followed by mass spectrometry identified BRG1, BAF250a and BAF170, all components of the SWI/SNF (mating type switching/sucrose nonfermenting) chromatin remodeling complex as proteins that coimmunoprecipitate with ADNP. These results were verified utilizing BRG1 antibodies. ADNP shRNA down-regulation resulted in microtubule reorganization and changes in cell morphology including reduction in cell process formation and cell number. These morphological changes are closely associated with the SWI/SNF complex multifunctionality (JBC 2007). Furthermore, using P19 cells as a differentiation model, we showed that ADNP expression and cytoplasm/ nuclar distribution is unique in neuronal differentiated cells compared to cardiovascular and nondifferentiated pluripotent cells. Small hairpin RNA down regulation was used to further investigate ADNP involvement in p19 neurodifferentiation. A ∼80% reduction in ADNP led to a substantial reduction in embryoid body formation and to a significant reduction (∼50%) in neurite number. These results position ADNP in direct association with neuronal cell differentiation and maturation.

*Supported by BSF, ISF, Lily, and Avraham Gildor Chair, Adams Super Center and Allon Therapeutics Inc*.

# Abstract: Dynamics of neuronal variability inmotor cortical areas during and switching between different tasks. What may it reflect?

**Authors:** Y. Mandelblat-Cerf, E. Vaadia

**Affiliations:** ^1^Department of Physiology, Faculty of Medicine, Hebrew university, Jerusalem, Israel; ^2^The Interdisciplinary Center for Neural Computation (ICNC), Hebrew University, Jerusalem, Israel

We present analyses designed to test the idea that variability of single cells' activity in motor cortical areas, reflects underlying processes associated with nonadaptation (over trained, center-out reaching, standard task), adaptation (visuomotor rotation) tasks, and switching from one to the other. Under the assumption that increase in variability is a result of neuronal processes, we investigate the hypothesis that adaptation starts at pre-motor areas, higher in the hierarchy of computation, gradually transfers to primary areas, until a stable representation is formed there. In contrast, a standard task, that incorporates merely the retrieval of an internal model and not its formation, is expressed mainly by changes in primary areas. To this end, we measured the across-trial variability of single cells responses, and found it has a significant dynamics for movement preparation period. During standard task, although it is assumed to be a stationary process, single cells in M1 showed a tendency of a gradual decrease in variability but neuronal activity in SMA did not show any significant dynamics. Switching to adaptation task, caused variability in M1 to increase, and later on, decreased back to initial level. Interestingly, similar dynamics was found in SMA, but earlier in the adaptation session, returning to initial level approximately when movement reached its asymptotic best level according to the learning curve. Surprisingly, when returning to standard task the dynamics in M1 was similar to that during adaptation task and no significant dynamics was seen in SMA as in the first standard task. These results suggest that any switching between internal models is reflected in M1 by an increase followed by a decrease in neuronal variability. Furthermore, they strengthen the notion that adaptation process starts in SMA and M1 follows it.

*Supported by Mafat, Johnson and Johnson,BSF,* *and by a contributions of Abraham and Ida Baruch Foundation and the Rosetrees trust.*

# Abstract: Novel TSPO ligands as neuroprotective agents

**Authors:** I. Maniv, L. Veenman, S. Leschiner, I. Spanier, I. Marek, A. Shterenberg, E. Hadad, M. Gavish

**Affiliations:** ^1^Faculty of Medicine, Technion-Israel Institute of Technology, 31096 Haifa, Israel; ^2^Faculty of Chemistry, Technion-Israel Institute of Technology, 32000 Haifa, Israel

Secondary brain damage often occurs after traumatic brain injury traumatic brain injury (TBI). TBI can result from explosions, including terrorist attacks, traffic and household accidents, and other forms of violence. Neuronal death due to secondary brain damage and neurodegeneration have in common an excitotoxic process leading from overexcitation of glutamate receptors to mitochondrial damage. The mitochondrial damage can lead to neuronal death, including apoptosis. Our studies suggest that the mitochondrial Translocator Protein (TSPO) plays an important role in this process. Recently, we have developed compounds that bind with high affinity to the TSPO and apparently block its apoptotic function. These novel compounds reduced basal apoptotic levels in neuronal cells. Some of them also reduced apoptosis induced by glutamate in the SH-SY5Y cell line by more than 50%. In our in vivo experiments we found that one of our ligands (9a) (15 mg/kg) reduces the adverse effects of kainic acid (9-10mg/kg) in rats. For example, seizures induced by kainic acid are indicative for neurodegeneration in the hippocampus. Severe seizure activity was reduced by 9a by more than 67%. As some of our compounds show antiapoptotic effects others show pro-apoptotic effects, which may have implications for the development of potential anticancer drugs. We envision that secondary brain injury due to TBI may be prevented by soldiers and paramedics carrying with them one of the antiapoptotic drugs we have developed and use it on site. This may reduce the incidence of disabilities presently occurring in the aftermath of TBI. In addition, such a drug might also find application in the treatment of neurodegenerative diseases.

*Supported by Dan Horovitz fund (L.V., M.G) Center for Absorption in Science, Israel (L.V)*.

# Abstract: The Inhibitory Effect of Mood Stabilizers on Adenylyl Cyclase Isoforms

**Authors:** L. Mann, E. Heldman, R. H. Belmaker, G. Agam

**Affiliations:** ^1^Department of Clinical Biochemistry, Israel; ^2^Stanley Research Center, Ben-Gurion University of the Negev and Mental Health Center, Beer-Sheva, Israel

Bipolar disorder has been hypothesized to be associated with an enhanced signaling activity of the cAMP cascade. The mood stabilizers lithium (Li^+^), carbamazepine (CBZ) and valproate, at therapeutic concentrations, exert an inhibitory effect on forskolinandGαs-coupled receptor activated cAMP accumulation. The aim of this work was to study the effects of lithium and carbamazepine on each of the membrane-bound isoforms of aednylyl cyclase (1–9). COS7 cells were transfected with each of the AC isoforms cDNAs with or without D1-dopamine receptor cDNA. AC activity was measured as [3H]cAMP accumulation in cells pre-incubated for two hours with 1mM Li or 0.1mM CBZ followed by incubation with either the D1 agonist FKS-82958 or forskolin. The role of Mg^2+^ in Li's inhibition of AC was studied in membrane preparations from cells expressing AC5. When stimulated by forskolin, a direct activator of AC, both 1mM Li^+^ and 0.1mM CBZ inhibited only AC5 activity. When stimulated via D1 receptors, Li^+^-inhibitable isoforms were AC5 and AC7 (∼40–50% inhibition) while CBZ inhibited all isoforms studied (AC5, 7, 3, 2) by ∼50%. In isolated membrane preparations of AC5-transfected cells stimulated AC5 activity was completely abolished by 1mM Li, and 10mM Mg^2+^ significantly counteracted Li's inhibition. Since forskolin is a direct activator of AC, the specific inhibition of forskolinstimulated AC5 by both Li and CBZ suggests a direct effect of these drugs on this specific isoform. Reversal of Li's inhibition of AC5 activated via the D1-dopamine receptor by Mg^2+^ suggests that competition with endogenous Mg^2+^ ions is involved in the mechanism of the inhibition. Since CBZ inhibited all studied isoforms including AC5 and AC7, it is possible that AC5 and AC7 are involved in the mechanism of mood stabilization.

# Abstract: Actin polymerization in lateral amygdala is essential for fear memory formation

**Authors:** L. Mantzur, R. Lamprecht

**Affiliations:** Department of Neurobiology and Ethology, University of Haifa, Israel

The actin cytoskeleton is involved in key neuronal functions such as synaptic transmission, proteins and vesicles trafficking and morphogenesis. The addition of actin monomers to actin filaments in the processes of polymerization has been shown to be involved in these functions and is tightly regulated in neurons. The aim of the present study is to elucidate the role of actin polymerization in learning andmemory. Toward this end, we used the fear conditioning paradigm in which a rat is exposed to a tone (conditioning stimulus (CS); 40 seconds, 5 kHz) that coterminates with a mild footshock (unconditioned stimulus (US); 0.5 seconds, 1.5 mA). Rats were trained with 5 consecutive CS-US pairings with average ITI of 180 seconds. We microinfused the actin polymerization inhibitor cytochalasin D into the lateral amygdala (LA), a brain area mediating fear conditioning, and studied its effects onmemory formation. Microinfusion of cytochalasinD immediately before fear conditioning training impaired the formation of long-termfear conditioning memory. Cytochalasin D had no effect on fear conditioning memory retrieval when microinfused immediately before the test 24 hours after training, indicating also that cytochalasin D did not impair normal synaptic transmission in LA. The present results show that actin polymerization in LA is essential for fear conditioning memory formation.

# Abstract: The interaction of the drug candidate NAP with microtubules in the presence of tau

**Authors:** M. Maor, D. Dangoor, I. Gozes

**Affiliations:** HumanMolecular Genetic & Biochemistry Sackler Medical School, Tel Aviv University 69978, Israel

NAP (NAPVSIPQ), a short peptide, derived from activitydependent neuroprotective protein (ADNP), was revealed to be a novel neuroprotective drug candidate. Femtomolar concentrations of NAP appear to provide neuroprotection in vitro against tetrodotoxin, N-methyl-D-aspartate, naturally occurring cell death, dopamine toxicity, tumor necrosis factor-α toxicity and oxidative stress. NAP was further shown to reduce mortality and morbidity, produce cerebroprotection, reduce apoptosis and accelerate learning and memory abilities in a broad variety of in vivo models. A significant interaction between NAP and tubulin unraveled a possible mechanism of this novel peptide. Through this interaction NAP seems to promote tubulin assembly and microtubules reorganization even in the presence of zinc intoxication. This study is aimed to assess the characteristics of NAP interaction with microtubules. Pelleting assays were used to determine the effect of NAP on tubulin and co-assembly into microtubules, in the presence of tau. The assembled microtubules were examined by electron microscopy. NAP (1 pM) treatment increased microtubule density (10 *μ*M), in the presence of tau (1 *μ*M), as evaluated by electron microscopy. Additionally, NAP-treated microtubules appear to be significantly longer and more curved compared to the rigid, short microtubules that were observed in the absence of NAP. In conclusion, NAP treatedmicrotubules assume a different structure– namely longer and smoother microtubular structures are formed when NAP is present. Thus, perhaps the interaction between NAP and microtubules resulting in conformational changes (length and shape) leads to the assembly and microtubules reorganization. These findings may be of great importance in elucidating the mechanism of NAP nueroprotective and neurotrophic roles.

*Supported by BSF, ISF, Lily and Avraham Gildor Chair*,* Adams Super Center, and Allon Therapeutics Inc.*

# Abstract: Neuronal substrates of head gaze following in monkeys: an fMRI study

**Authors:** S. Materna, P. W. Dicke, P. Thier

**Affiliations:** Department of Cognitive Neurology, Hertie Institute for Clinical Brain Research, Tuebingen, Germany

The ability to follow the gaze of another person is crucial for normal social interactions. This is not only true for humans, also in other social animals, like nonhuman primates, gaze following plays an important role for example in detecting the location of food and predators. However, compared to humans, nonhuman primates rely more on the head than on the eyes during gaze following. In this study, we attempted to reveal the neuronal substrates involved in head gaze following in rhesus monkeys using functional magnetic resonance imaging. In the first condition (gaze following) the monkeys had to detect the head direction of another monkey (presented on a screen) and to use this information to make a saccade to one of two dots (either left or right of the monkey face). In the control condition (colormatching) the monkeys had to use the color information provided by the fixation dot (presented in between the eyes of the monkey face) to make a saccade to the dot with the same color. Eye movements of the monkeys were recorded using a homemade iris-fit-eyetracking system. During both conditions, i.e., independent of which cue was used, activation was found bilaterally in (or near) the frontal eye fields, probably reflecting the need to make saccades. For the contrast “color matching minus gaze following” specific activation was found bilaterally in the inferior temporal gyrus, a region known to be involved in color processing. The opposite contrast (gaze following minus color matching) revealed a bilateral activation in the superior temporal sulcus (STS), most consistently in the middle part of the sulcus: the mSTS. A region in the posterior STS is known to underlie eye gaze following in humans. Hence, although human and nonhuman primates seem to rely on different directional cues, they may deploy similar and possibly homologous cortical areas to follow the gaze of a conspecific.

*Supported by the EU program “PERACT” and a grant from the BMBF (01GA0503)*.

# Abstract: Blood glutamate scavenging in the prevention of glioma invasiveness

**Authors:** A. Matuzany-Ruban, V. I. Teichberg

**Affiliations:** Department of Neurobiology, Weizmann Institute of Science, Rehovot, Israel

Studies performed in the last few years have shown that malignant glioma cells secrete in the peritumoral space massive amounts of the neurotransmitter glutamate, and by doing so, promote their own expansive growth at the expense of the neighboring neurons that undergo excitotoxic cell death. Since these studies suggest now that interfering with brain glutamatemay control glioma cell proliferation and invasion, and may limit brain neuronal injury caused by glutamate excess, we have evaluated in the present study the therapeutic efficacy against glioma of a novel neuroprotective strategy recently developed in our laboratory that is based on the ability to cause a decrease of excess brain glutamate levels. By accelerating a naturally occurring brain-to-blood glutamate efflux, we cause a decrease of deleterious glutamate levels in brain. This is achieved by the administration of oxaloacetate (OxAc) which, by scavenging blood glutamate levels, increases the driving force for the efflux of glutamate from brain into blood. Methods: Cultured C6 glioma cells were implanted in rat brain to induce tumor growth and its accompanying neovascularization. OxAc was daily prepared and added to the drinking water of the implanted rats while the rats in the control group drunk a NaCl solution at the same osmolarity of the OxAc solution (0.4 M). Each group consisted of 12 rats housed in individual cages. The OxAc or NaCl treatment was started five days after glioma implantation and the tumor development was followed using Magnetic Resonance Imaging (MR)I up to three weeks. Results: MRI analysis shows that OxAc-treated rats displayed a fold tumor growth of 8.9 ± 1.9 (*n* = 12) while that of the NaCltreated rats was 20.5 ± 4.6 (*n* = 8; *P* = .018). Conclusion: The preliminary results obtained support the concept that decreasing excess glutamate in blood/brain may serve as an alternative therapeutic strategy in the management of malignant gliomas.

# Abstract: The neurophysiological correlates of abnormal behavior in a primate model of Tourette syndrome and their response to high frequency stimulation

**Authors:** K. McCairn, M. Bronfeld, I. Bar-Gad

**Affiliations:** Gonda Brain Research Center & Goodman Faculty of Life Sciences, Bar-Ilan University, Israel

Tourette syndrome (TS) is characterized by chronic motor and vocal tics and has a high comorbidity rate with obsessive compulsive behavior (OCB) and attention deficit/hyperactivity disorder (ADHD). Despite the different clinical manifestations of these disorders, they have all been associated with a common neuronal pathway: the corticobasal ganglia loop. Most of the nuclei of the basal ganglia use GABA (gamma-aminobutyric acid) as their primary neurotransmitter for both inter and intranucleus information transfer. Bicuculline (GABAA antagonist) injection to the primate putamen evokes motor tics resembling the primary TS symptoms while injections to different domains within the globus pallidus external segment (GPe) produce behavioral correlates of the comorbid disorders (stereotypy and hyperactivity with attention deficits). In our study we use extracellular multielectrode recording to characterize the changes in firing patterns of multiple single neurons and to uncover the neuronal interaction within small neuronal networks following the injection. Our results demonstrate that local blockade of GABA leads to the formation of intermittent high-frequency activity within the GPe. Moreover, neurons within the injected domain transition from uncorrelated firing to a synchronized firing pattern. The observed changes in neuronal activity are highly correlated to the behavioral symptoms as assessed by the limb kinematics and video analysis of expressed behaviors. Neurons within the GPi display highly locked activity prior to the tic performance. This investigation of GABAergic transmission within the corticobasal ganglia loop provides unique insight into the information encoding within these circuits and how the breakdown of normal activity leads to the severe clinical symptoms associated with TS. Our studies of HFS within the GP demonstrate that it leads to a locked response to stimulation which changes the firing rates and breaks down abnormal correlated activity.

# Abstract: Visual processing under limiting visual conditions

**Authors:** A. Meshulam, O. Yehezkel, Y. Mandel, M. Rosner, B. Belkin, U. Polat

**Affiliations:** Tel Aviv University, Sackler Faculty of Medicine, Goldschleger Eye Research Institute, Tel Hashomer, Israel

Visual perception changes dramatically under day and night conditions. Visual performance under vision-limiting conditions, for example, night vision of all people with either impaired or outstanding night-vision, is affected. In this study we explored how different light conditions change the visual abilities of humans. We measured basic functions of the visual system such as visual acuity (VA), day contrast sensitivity (CS), and foveal mesopic CS. The subjects were tested monocularly (*N* = 30 eyes) and had corrected-to-normal visual acuity. The mesopic CS was measured under conditions of full darkness, using natural density filters to cover the monitor, allowing a background luminance of 0.03 cd/m2. The CS measured for Gabor targets was with spatial frequencies of 3–9 cpd. In addition, we tested the influence of external blur (+0.50 diopter, inducing night myopia) on the tolerance of the visual functions at night. The results show that CS was affected twofold more than the VA under night conditions (reduced by a factor of 7.2, 0.85 log units vs 2.6, 0.4 log unit), compared with the day conditions. The crowding effect did not increase under night conditions. Under the blur condition, VA is only slightly affected, whereas CS is remarkably reduced (66%). Thus, visual functions are affected differently under different visual conditions; while tasks performed near threshold are affected remarkably, supra-threshold tasks are less affected. Therefore, visual perception cannot be predicted from standard visual acuity test.

*Supported by a grant from the Ran Naor* *Foundation for the Advancement of Road Safety Research.*

# Abstract: The anatomical basis of functional MRI

**Authors:** A. Mezer, Y. Yovel, O. Pasternak, T. Gorfine, Y. Assaf

**Affiliations:** ^1^Department of Biochemistry, Faculty of Life Sciences, Tel Aviv University, Tel Aviv, Israel; ^2^Department of Neurobiochemistry, Faculty of Life Sciences, Tel Aviv University, Tel Aviv, Israel; ^3^School of Computer Sciences, Faculty of Exact Sciences, Tel Aviv University, Tel Aviv, Israel; ^4^Functional Brain Imaging, TheWohl Institute for Advanced Imaging.Tel Aviv Sourasky Medical Center, Israel

Functional MRI (fMRI) has become one of the leading methods in neuroscience. It was recently suggested that resting state fMRI can be used to define the default state of brain activity, functional connectivity and basal activity. The measurement of basal activity through resting-state fMRI opened new horizons of this methodology. Here we show that cluster analysis of the repeated blood oxygenation level dependent (BOLD) measurements of the restingstate signal resembles the anatomical and cyto-architectonic arrangement of the tissue in cortical and subcortical gray matter as well as in the white matter. Therefore, without imposing functional paradigms on the BOLD signal (as in rest fMRI), separating the functional contribution to the signal fluctuation from the anatomical contribution is challenging. Nevertheless we suggest that multidimensional brain MRI acquisition (like the repeated rest BOLD signal) can be used for parcellation of the brain into regions that could not be extracted with conventional MRI.

# Abstract: Acute dopamine exposure attenuates GABAA receptor currents in the VTA through D1 and D2 dopamine receptors

**Authors:** A. Michaeli, R. Yaka

**Affiliations:** Department of Pharmacology, School of Pharmacy, The Hebrew University of Jerusalem, Israel

Drugs of abuse act through intervening in the normal activity of the reward system. The reward system is composed from interconnected distinct brain regions, including the ventral tegmental area (VTA), the main source of dopamine in the brain, which synapses onto the nucleus accumbens (NAc) neurons. The psychostimulant cocaine inhibits dopamine reuptake to VTA neurons, as a consequence synaptic dopamine concentration in the NAc elevates, and a typical psychostimulant effect is achieved. NAc dopamine levels can also be modulated by changes in the activity of VTA dopaminergic neurons. Over excitation of these neurons or its reduced inhibition can lead to enhanced dopamine release in the NAc. Therefore we aimed to determine whether cocaine increases the activity of VTA neurons through inhibition of GABAA receptors-mediated currents. Rat brain slices containing the VTA were prepared, and VTA dopaminergic neurons were identifiedmorphologically and electrophysiologically. Whole cell GABAA inhibitory post synaptic currents (IPSCs) were recorded using standard patch clamp technique, in response to electrical stimulation of the pre frontal cortex (PFC) afferents and bath application of dopamine. We found a significant reduction of GABAA-mediated IPSCs following application of 30 microM dopamine. This reduction was blocked either by dopamine D1/D5 receptors antagonist SCH23390 or by the dopamine D2 receptor antagonist eticlopride. Although the molecular mechanism by which dopamine reduces IPSCs is not fully understood, these results reveal a new possible aspect of cocaine action in the VTA. This finding suggests that in addition to cocaine inhibition of dopamine reuptake, a reduction of IPSCs occurs, and together these events mediate the development of cocaine addictive properties.

*Supported partially by grant from the National Institute for Psychobiology in Israel.*

# Abstract: ADNP messenger RNA knock down—a novel approach for cancer therapy

**Authors:** I. Michailovici, D. Dangoor, I. Gozes

**Affiliations:** Human Molecular Genetic and Biochemistry Sackler Medical School, Tel Aviv University 69978, Israel

The human activity-dependent neuroprotective protein (ADNP) gene was mapped to chromosome 20q12-13.2, a region associated with aggressive tumor growth, frequently amplified in many neoplasias. Human ADNP mRNA expression was shown to be significantly increased in colon and breast cancer tissues in comparison to normal adjacent tissues, while inhibition of ADNP expression by antisense oligodeoxynucleotides results in HT-29 (colon cancer) cell death associated with increases in P53 expression. The current research aims at improving ADNP knockdown in order to potently inhibit cell proliferation in cancer therapy. Assays were preformed using 3 cell lines: HT29 (colon cancer), A375 (melanoma) and PC3 (prostate cancer). Cell viability was measured by the MTS colorimetric assay. Inhibition of growth was performed by antisense oligodeoxynucleotides. ADNP knockdown was examined at the mRNA level, using quantitative real time PCR, and at the protein level, using western blot analysis. Dramatic enhancements in ADNP knockdown were obtained as follows: (1) The use of a transfection reagent that increases the uptake of the ADNP antisense into cells. (2) The use of the aureolic acid antibiotic, Mithramycin, which binds selectively to GC-rich DNA sequences and blocks preferential binding of proteins to GC-rich elements in gene promoters (such as the ADNP promoter). Both inhibition of ADNP mRNA by antisense oligonucleotides, and inhibition through the blockade of ADNP transcription factor from binding to its promoter caused a significant decline in ADNP expression. Down regulation of ADNP inhibited cell proliferation, suggesting ADNP inhibition as a novel approach for cancer therapy. Previous studies have shown that ADNP is essential for brain function ([1]), requiring tissue-specific targeting methods.

*Lily and Avraham Gildor Chair for* *the Investigation of Growth Factors.*

## References

[1] (2007). *Journal of Pharmacology and Experimental Therapeutics*.

# Abstract:  the “good teacher” and the “bad teacher”?

**Authors:** R. Mitelman, H. Bergman

**Affiliations:** ^1^The Interdisciplinary Center for Neural Computation, Hebrew University, Jerusalem, Israel; ^2^Department of Physiology, Hebrew University-Hadassah School of Medicine, Jerusalem, Israel; ^3^The Eric Roland Center for Neurodegenerative Diseases, Hebrew University-Hadassah School of Medicine, Israel

Dopamine (DA) neurons in the basal ganglia (BG) play amajor role in reinforcement learning, as the DA signal is proportional to the difference between actual and predicted reward. Such a signal could play the role of the prediction error signal of a TD learning algorithm implemented in the BG (1-4). Indeed, a proportional increase in the firing of DA neurons has been found in cases of positive errors (event's value higher than expected). However, no such outcomes have been reported in the negative domain (lower than expected value). Instead, the decrease in firing rate is either by a smaller gain than of the positive domain (1), or it is lowered to a constant level (2). Our work focuses on the possibility of such asymmetric signals to be an error signal in a TD-like algorithm. We simulated a probabilistic task, in which the agent sequentially received a set of stimuli, with different probabilities of reward or aversion. The algorithm calculated the value of each of the stimuli, using a method similar to TD, but negative error values were decreased by a multiplicative factor, S, which can vary between 0 and 1, imitating the observed asymmetric DA signal. The calculated values are proportional to the stimulus outcome and probability even with S close to 0. When S is closer to 0, the learned values are less linear and learning speed decreases. The nonlinearity is always an increase of the calculated values, in comparison with the original TD values. The differences between the calculated and the original values are dependent on the probability of future reward/aversive. We conclude that TD asymmetric signal could be used as an error signal in a reinforcement learning algorithm, as implemented in the BG; though the resulted learning is slower and less accurate than with a symmetric signal.

## References

[1] Satoh T, Nakai S, Sato T, Kimura M (2003). Correlated coding of motivation and outcome of decision by dopamine neurons. *Journal of Neuroscience*.

[2] Bayer HM, Glimcher PW (2005). Midbrain dopamine neurons encode a quantitative reward prediction error signal. *Neuron*.

[3] Schultz AS (1997). *Science*.

[4] Sutton RS, Barto AG (1998). *Reinforcement Learning*.

# Abstract: Facilitating synapses mediate working memory

**Authors:** G. Mongillo, O. Barak, M. Tsodyks

**Affiliations:** ^1^Laboratory of Neurophysics and Physiology, UMR 8119, CNRS–Université René Descartes, France; ^2^Department of Neurobiology, Weizmann Institute of Science, Israel

The common mechanistic account of short term memory (for several seconds) requires persistent firing of selective groups of neurons. This mechanism suffers from a substantial metabolic cost, and is vulnerable to interference. Recent experiments also show that persistent firing often has a very low rate and can even disappear for parts of the period. We propose a differentmechanism, based on short termsynaptic plasticity, where the information about memories is stored using pre synaptic calcium that mediates synaptic facilitation. We implement this mechanism using a rate model and a large scale simulation of spiking neurons. The advantages of this mechanism are illustrated and discussed.

# Abstract: Statistical analysis of rhythmicmotor patterns produced by the spinal cord

**Authors:** Y. Mor, A. Lev-Tov

**Affiliations:** Department of Anatomy and Cell Biology, The Hebrew University Medical School, Jerusalem, Israel

The central pattern generator (CPG) is a network of spinal neurons that produces the rhythmic motor patterns required for coordinated walking running, and swimming. Accurate reproducible quantification is crucial for the proper interpretation of the rhythm produced under normal and pathological conditions. Because the output of the CPG varies with time its analysis cannot be performed by statistical methods that assume data stationarity. The present work introduces the Wavelet (WT) and WT coherence as tools for automated quantitative analysis of the nonstationary rhythmic patterns produced by the spinal pattern generating circuitry in the isolated spinal cord preparation of the neonatal rat, and of the surface EMG signals recorded from limb muscles of spinal cord injury patients during body weight supported treadmill locomotion. The analyses enabled us to characterize the dynamic profile of the signals, to assess the linear relation between spectra of any given pair of signals and to uncover hidden components of the rhythm in the time/frequency domain. The quantitative indices extracted from the spectra can be used for routine characterization of motor rhythms in experimental animals and for assessing the effectiveness of clinical treatments in spinal cord injury patients. The ability of the WT and WT coherence algorithms to extract the rhythmic parameters of complex nonstationary behaviors over a wide range of frequencies as a function of time, will be demonstrated and its implications will be discussed.

# Abstract: Alterations in hippocampal long-term potentiation following fear conditioning

**Authors:** H. Motanis, M. Maroun

**Affiliations:** Department of Neurobiology and Ethology, Center of Brain and Behavior, University of Haifa, Israel

Studies have shown that exposure to a stressful experience impairs long-term potentiation (LTP) in the CA1 subregion of the hippocampus. In the current study we assessed the potential differences that contextual fear conditioning could elicit in hippocampal CA1 LTP as compared to exposure to elevated platform stressor. In accordance with previous results, thirty minutes of prior exposure to the elevated platform inhibits the induction of LTP in the CA1 hippocampal region, whereas fear conditioning reduced CA1 LTP without inhibiting it completely. We further investigated the effects of fear conditioning on LTP and we found differential effects on LTP depending on time of memory retrieval following fear conditioning, the strength of memory and the context of retrieval. Specifically, rats that underwent memory retrieval one hr after conditioning showed impaired LTP, and rats undergoing retrieval 24 hrs after conditioning showed reduced levels of LTP. Moreover, short-term memory “retrieval” in a different context than the conditioning context resulted in enhanced levels of potentiation, whereas longterm memory “retrieval” resulted in blockade of the LTP in CA1 area. The injection of the NMDA receptor antagonist MK-801 (300 microgram per kilogram) before fear conditioning, prevented the associated effects of fear conditioning on LTP, showing that the effects obtained in this experiment are due to fear conditioning. These data suggest that the interaction between stress and learning process could be of a crucial significance in determining the net effect on CA1 LTP.

# Abstract: Incensole acetate, an incense component, elicits psychoactivity by activating TRPV3 channels

**Authors:** A. Moussaieff, E. Fride, E. Shohami, R. Mechoulam

**Affiliations:** ^1^Department of Medicinal Chemistry, Medical faculty, Hebrew University of Jerusalem, Israel; ^2^Department of Pharmacology, School of Pharmacy, Hebrew University of Jerusalem, Israel; ^3^Department of Behavioral Sciences, Ariel University Center of Samaria, Israel

Burning of Boswellia resin as incense has been part of religious and cultural ceremonies for millennia and is believed to contribute to the spiritual exaltation associated with such events. Transient receptor potential vanilloid 3 (TRPV3) is an ion channel implicated in the perception of warmth in the skin. TRPV3 mRNA has also been found in neurons throughout the brain; however, the role of TRPV3 there remains unknown. Here we show that incensole acetate (IA), a major Boswellia resin constituent, is a potent TRPV3 agonist that causes anxiolytic-like and antidepressive-like behavioral effects in wild type (WT) mice with concomitant changes in c-Fos activation in the brain. These behavioral effects were not noted in TRPV3−/− mice, suggesting that they are mediated via TRPV3 channels. IA robustly activated TRPV3 channels stably expressed in HEK293 cells and in keratinocytes from TRPV3+/+ mice. It had no effect on keratinocytes from TRPV3−/− mice and showed modest or no effect on TRPV1, TRPV2 and TRPV4, as well as on 26 other receptors, ion channels and transport proteins. Collectively, our results imply that TRPV3 channels in the brain play a role in emotional regulation. Furthermore, IA's biochemical and pharmacological effects may also provide a biological basis for deeply rooted cultural and religious traditions.

# Abstract: Limitations of stimulus-anchoringmechanisms: evidence from behavioral and ERP data

**Authors:** M. Nahum, M. Levi, M. Ahissar

**Affiliations:** ^1^ICNC, The Hebrew University of Jerusalem, Israel; ^2^Psychology Department, The Hebrew Univerisry of Jerusalem, Israel

In 2-tone frequency discrimination tasks, participants listen to two sequentially presented tones and are asked to decide which tone is higher. Recent results from our lab (Lubin & Ahissar, ISFN 2005) showed that if one of the tones is repeated across trials (“reference”) at a consistent temporal position, performance dramatically improves compared to a no-reference condition. However, if the location of the reference randomly varies between first and second positions, subjects perform as if there is no reference at all. In order to better understand the reference-anchoring mechanism, we designed a 1-1 paradigm (“1-1 reference”), in which the reference was first on odd trials and second on even trials. Thus, there is no uncertainty in reference position across trials, but there is no consistency across consecutive trials either. We found that overall performance in this condition was significantly worse than when the two reference conditions were assessed separately, mainly due to poor performance on trials with reference at the second position. This worsening suggests that our reference-anchoring mechanism cannot fully benefit even from a very simple temporal structure. We further asked whether training enables better use of the reference in the reference-second condition. We therefore trained a group of subjects for 5 days on this protocol. Training improved both reference-first and reference-second thresholds, which reached a plateau by the third day of training. However, performance in reference-second remained poorer than performance in reference-first. ERP data recorded fromwelltrained subjects showed a clear correlate of this learning, as well as the remaining superiority of the reference-first condition. Together, these results indicate that reference-anchoring favors consistency at trial onset, and can only partially benefit from a consistent temporal pattern across nonconsecutive trials. Training improves but does not eliminate this bias.

*This study was supported by an* *ISF Center of Excellence grant* *and by the Institue of Psychobiology.*

# Abstract: A dynamic model for matching behavior that is based on the covariance of reward and action

**Authors:** T. Neiman, Y. Loewenstein

**Affiliations:** ^1^Department of Neurobiology, Israel; ^2^Department of Cognitive Science, Israel; ^3^the Interdisciplinary Center for Neural Computation, the Hebrew University, Jerusalem, Israel

According to Herrnstein's “matching law” that describes choice behavior in a foraging-like reward schedule, the fraction of time allocated to an alternative is proportional to the number of rewards harvested from that alternative. A change in the reward schedule results in a modification of choice preference such that the matching law is retained. This process of adaptation can be remarkably fast. It has been reported that the rate of adaptation in rats is comparable to the limit set by an ideal observer. The algorithms underlying adaptation to matching behavior are a subject of debate. Melioration theory posits that organisms are sensitive to the local rates of return (number of reinforcements obtained at an alternative divided by time spent at that alternative) and shift their choice preference in the direction of the more profitable alternative. Alternatively, it has been suggested that choice preference depends only on the estimated income from the two alternatives and has no direct dependence on the actions made by the subject. We formalize representatives of the two classes of models in a two-state Markov model in which the transition probabilities are adjustable. We show that in both models, changes in the transition probabilities are driven by the covariance of reward and action and that the two models differ only in their learning rate. Thus, we argue that the separation between return-based and income-based models is artificial. We compare adaptation of a two-state Markov model in which learning is driven by the covariance of reward and action with behavior of rats responding on two levers for pleasurable brain stimulation in a concurrent variable interval schedule. We show that the behaviors of the model and the rat are very similar. In particular, we show that the model reproduces the experimentally observed fast adaptation to matching following a change in the reward schedule.

# Abstract: Slow modulations of spontaneous firing rates and LFP Gamma power in human auditory cortex during wakefulness and sleep

**Authors:** Y. Nir, I. Dinstein, R. Mukamel, L. Fisch, H. Gelbard-Sagiv, A. Arieli, I. Fried, R. Malach

**Affiliations:** ^1^Department of Neurobiology, Weizmann Institute of Science, Rehovot 76100, Israel; ^2^Center for Neural Science, New York University, NY 10003, USA; ^3^David Geffen School of Medicine, University of California Los Angeles (UCLA), CA 90095, USA; ^4^Functional Neurosurgery Unit, Tel Aviv Medical Center, Tel Aviv University, 69978, Israel

Recently, many fMRI studies have focused on spontaneous waves of activity in human cortex, in the absence of sensory stimulation. However, it is still not clear what neuronal activity may underlie these fluctuations. In this study, we examined neuronal action potentials and local field potentials (LFP) in human auditory cortex during spontaneous activity in wakefulness and sleep, in order to examine the dynamics of neuronal activity that may give rise to spontaneous fMRI fluctuations. Three patients underwent monitoring with intracranial depth electrodes for potential surgical treatment. Patients provided written informed consent, and the study conformed to the guidelines of the Medical IRB at UCLA. Simultaneous recordings of single unit and LFP activity from multiple electrodes in human auditory cortex were collected while patients rested in a dimly-lit, silent room (a total of 11 recording sessions in 3 patients). The sound during the experimental session was recorded along with the neuronal data to verify quiet recording conditions. Very slow (<0.1 Hz) fluctuations in gamma power and neuronal firing rates in auditory cortex exhibited high correlation between the two hemispheres. Such correlations were selective for these regions, and did not spread to other recorded locations. These results suggest that large spontaneous fMRI fluctuations could result from slow, coherent modulations in neuronal activity, rather than relatively fast, stimulus-evoked bursts of activity, which are typical of sensory-driven responses. Therefore, these results emphasize the caution that should be exercised when interpreting fMRI data, and may help resolve the paradox of similar fMRI response patterns in sensory cortex in the presence and absence of sensory stimulation.

*Funded by a US-Israel BSF grant* *to I. Fried and R. Malach.*

# Abstract: BL-1021: a new chemical entity for treatment of naturopathic and acute pain

**Authors:** Y. Nisemblat, Y. Geffen, I. Winkler, L. Klapper, K. Savitsky, A. Nudelman, A. Rephaeli, I. Gilad, Weizman

**Affiliations:** ^1^BioLineRx, Ltd., Jerusalem, Israel; ^2^Department of Chemistry, Bar Ilan University, Ramat Gan, Israel; ^3^Felsenstein Medical Research Center, Tel Aviv University, Beilinson Campus, Petach Tikva, Israel

Neuropathic pain (NP) is a pain which initiated or caused by a primary lesion or dysfunction in the nervous system. It is associated with various conditions including shingles and diabetes and affects 15 million people in the USA alone. The available treatment includes antidepressants (as TCAs) and anticonvulsants and consider limited with only 30–50% reduction in pain in ∼50% of the patients. Most of these drugs are associated with significant side effects most noted are sedation and cardiac arrhythmia, which create an urgent need for a new class of medications. BL-1021 is an orally available small molecule which is designated for the treatment of NP. The molecule was orally given to a variety of animal models of acute and chronic pain. In the acute hot plate model, BL- 1021 demonstrated rapid onset and longer latency of nociceptive reaction to heat as compared toNortriptyline (a common antineuropathic TCA). In the acute formalin model, BL-1021 reduced the early nonciceptive response and the late imunological response and was more effective than Nortriptyline. In the Chungmodel (spinal nerve ligation) of neuropathic pain, BL-1021 was significantly more effective than Nortriptyline in reducing mechanical allodynia and thermal hyperalgesia. Moreover, BL-1021 was more effective in reducing thermal hyperalgesia than Gabapentin, the gold standard drug for the treatment of neuropathic pain. BL-1021 showed low side effects relatively to other TCAs used for the treatment of Neuropathic pain: no sedation up to 80 mg/kg in the Irwin test, no decrease in the time spent on the Rotarod as found in Nortriptyline and moreover, BL-1021 was found to be safe at the effective doses observed in the Chung model in the hERG model for the prediction of cardiac arrhythmia,. Additionally, BL-1021 was found to inhibit sodium currents in neuroblastoma cells and more specifically, sodium currents (early and late) through Nav. 1.8, which is associated with Neuropathic pain.

*This work was supported by BioLineRx*.

# Abstract: Perceptuo-motor transparency in bilateral teleoperation

**Authors:** I. Nisky, A. Karniel

**Affiliations:** Department of Biomedical Engineering, Ben-Gurion University of the Negev, Israel

In bilateral teleoperation, the operator holds a local robot which local robot which determines the motion of a remote robot and continuously receives delayed force feedback. The delay may cause instability, distortion in perception, and distortion of action. The nervous system inherently includes significant time delays, and therefore we expect the motor system to be capable of handling delayed feedback. Transparency is a measure of teleoperation system fidelity. The ideal teleoperator system is the identity channel, where there is no delay or distortion. In measuring transparency, one should consider also the human operator and therefore we propose the following three components of transparency: (a) perceptual transparency: the operator cannot distinguish between the teleoperation channel and an identity channel; (b) local motor transparency: the movement of the operator does not change when the teleoperation channel is being replaced by an identity channel; (c) remote transparency: the movement of the remote robot does not change when the teleoperation channel is being replaced by an identity channel. We report series of our recent studies with this context in mind. In our studies of perceptual transparency of spring like surfaces we found that subjects overestimate delayed spring-like surfaces with boundary and underestimate nondelayed boundary shifted surfaces. In our studies of local motor transparency we used an adaptation protocol to extract the behavioral implications of the subjects' expectation. We found that the subjects' answers matched our measure of the motor behavior in the case of delayed stiffness but not in the case of shifted boundary. These discrepancies clearly demonstrate that a multidimensional measure of transparency must be employed in the design of teleoperation systems.

*Supported by Grant no. 2003021 from* *the United States-Israel Binational Science Foundation* *and by the National Institute for Psychobiology in Israel.*

# Abstract: Tempol Diminishes Oxidative Damage and Attenuates Behavioral Sensitization Following Cocaine Administration in Rats

**Authors:** R. Numa, R. Kohen, R. Yaka

**Affiliations:** ^1^Department of Pharmacology, School of Pharmacy, The Hebrew University of Jerusalem, Israel; ^2^Department of Pharmaceutics, School of Pharmacy, The Hebrew University of Jerusalem, Israel

Oxidative stress (OS) and reactive oxygen species play an important role in cocaine addiction and neurotoxicity, throughout excessive production of 6-OHDA or by redox cycling of cocaine oxidized metabolites. Cocaine stimulates the production of OS in a range of organs including the brain. However, the association between acute or repeated cocaine administration and overall antioxidant power in the brain reward system is poorly understood. Here we examine the hypothesis that enrichment of antioxidant power in the brain will attenuate cocaine-induced oxidative damage and in turn will reduce cocaine-induced behavior. In-vitro: PFC andNAc slices were treated with cocaine or tempol + cocaine for 15 min, homogenized and frozen for analysis. In-vivo: rats were received 2 daily i.p. injections of tempol or vehicle followed by cocaine, and locomotor activity was measured for 45 min. On day 19, all rats received a single cocaine challenge and locomotor activity wasmeasured. At the end of these sessions, the PFC and NAc were dissected and frozen for analysis. Analysis of oxidative stress markers were evaluated using TBARS and Griess assays and total antioxidant capacity was quantified using FRAP and ORAC assays. We found that acute cocaine exposure increases OS markers and TAC both in slices and in-vivo. However, treatment with tempol reduces OS markers and preserves TAC both in vitro and in vivo. Interestingly, pretreatment with tempol attenuated initiation and expression of cocaine-induced behavioral sensitization, without affecting basal levels of locomotor activity. Finally, tempol prevented the expansion of OS markers following repeated administration of cocaine. Our findings suggest that tempol has a neuro-protective effect against cocaine toxicity, by averting oxidative stress amplification in principle areas of the reward system. These findings implicate that administration of this agent might be considered as an optional treatment of cocaine addiction.

# Abstract: Haptics and subjective emotional significance

**Authors:** E. Ofek, M. Reiner

**Affiliations:** Human Virtual Reality Lab, Technion, Israel

**Introduction**

a sympathetic pat on the friend's shoulder, a gentle stroke on the face of a crying baby, an enthusiastic handshake or a violent slap, are all related to a kind of touch. Patterns of touch are used to convey emotions. Manual performance involves touch. How would manual performance be affected once the subject's emotional state changes? Improved? Worsened? In this study we used subjectively significant cues (first names) to assess the impact of emotional states on haptic performance of a task in a hapto-visual virtual world. The purpose of this study was to describe the behavioral and neural correlates of emotional and haptic processing. Methods: RT and ERPs were measured from 10 subjects engaged in a haptic task. The subjects were listening to names, while holding a PHANTOM arm in their right hand. They pressed a button when the PHANTOM moved. The effect of subjective valence of the names on the response-time to the haptic targets was assessed. ERPs were measured both to the first names and to the haptic targets. The subjective affective valence of the first names was assessed by a questionnaire (Ofek).

**Results**

Shorter reaction times to haptic targets were found after subjective negative names. Distinct response was found to different names, differentiated by their subjective affective valence. Stronger brain response was found to haptic stimuli after subjective positive names. Distinct brain responses to the same haptic stimuli were found, when administered after names with differential subjective affective valence.

**Discussion**

As proposed, subjective emotions and haptic processing interact. Negative subjective significance, rather than positive, improved haptic performance, suggesting that “threat” may be more effective in improving manual performance. Implications are mainly for design of (tele)manipulation.

# Abstract: Development of declarativememory systems in the human brain

**Authors:** N. Ofen, J. D. Gabrieli

**Affiliations:** ^1^Psychology Department, Stanford University, Stanford, Calif, USA; ^2^Brain and Cognitive Sciences, MIT, Cambridge, Mass, USA

Multiple brain regions including medial temporal (MTL) lateral prefrontal (LPFC) and parietal cortex are involved in successful memory encoding for adults. This study is the first to investigate the development of brain regions involved in successful encoding. Adults (*n* = 14, age range 19–24) and children (*n* = 35, age range 8–17) studied pictures of scenes while being scanned with 1.5T fMRI scanner. Following the study phase, a recognition test was administrated to all participants. Items that were recognized were further judged as either familiar or remembered. Occipital, parietal, and temporal visual regions as well as MTL and DLPFC regions were found to bemore active for items that were later remembered compared to items that were later forgotten. However, within these regions activations associated with successful encoding, positively correlated with age in the DLPFC, but not in MTL, regions. Within parietal regions, right superior parietal lobe (Brodmann area, BA 7) showed increased subsequent memory activation with. In contrast, bilateral lateral parietal regions (BA 39/40) and a posterior cingulate/precuneus region (BA 7/31), showed increased deactivations with increased age for items later remembered compared to items later forgotten. In adults, adjacent regions within the parietal cortex demonstrate opposite subsequentmemory effects. Moreover, these lateral and posterior parietal regions are within identified brain regions that are activated during rest and deactivated during attention-demanding goal-directed tasks, the so called “default mode of brain functions.” Together, these data suggest that with age prefrontal and parietal regions are progressively recruited to reach adult-like brain activation during successful encoding. Furthermore, these data suggest that the magnitude of deactivations in regions previously described as part of the default mode of brain functions increase during development.

# Abstract: Effects of early auditory experience on the auditory space map in the barn owl's brain

**Authors:** A. Oged, Y. Gutfreund

**Affiliations:** Department of Physiology, The Faculty of Medicine, The Technion, Israel

In the midbrain of the barn owl, an auditory localization “specialist,” there are auditory spatial maps based on the computation ofmonaural and binaural localization cues. Because the association between the auditory localization cues and the corresponding locations in space depends on factors that can change throughout the life of an animal (e.g., head size) it is expected that the maps are shaped by sensory experience. We investigated the importance of normal early experience on the development of the auditory map in the optic tectum (OT) by raising newly hatched barn owls in omnidirectional broad-band masking noise (1–15 kHz, 90 dB SPL), depriving them fromcoherent binaural experience. We found that the mapping of interaural level difference (ILD), the main cue for elevation in owls, was deteriorated in noisereared owls. On the other hand, the map of interaural time difference (ITD), the main cue for horizontal location, was found to be less sensitive to early experience. Only in one out of five tested owls we found significant deficits in ITD mapping. Following the removal from the noise box, the tuning of the neurons gradually improved and their mapping resembled more closely that of a normal owl. These results imply that early coherent binaural experience is essential for the correct formation of the auditory map in the barn owl, especially along the vertical domain and that deficits due to early abnormal experience can be corrected with time.

*This work was supported* *by an ISF personal grant.*

# Abstract: Through a barn owl's eyes: interactions between scene content and visual attention

**Authors:** S. Ohayon, Harmening, H. Wagner, E. Rivlin

**Affiliations:** ^1^Computer Science Department Israel Institute of Technology Haifa 32000, Israel; ^2^Institute of Biology II, RWTH University, Kopernikusstrasse 16, 52074 Aachen, Germany

In this study we set to investigate visual attention properties of freely behaving barn owls using a miniature wireless camera attached to their heads. The tubular eye structure of barn owls makes them ideal subjects for this research since it limits their eye movements. Video sequences recorded from the owl's point of view capture part of the visual scene as seen by the owl. Automatic analysis of video sequences revealed that during an active search task owls repeatedly and consistently direct their gaze in a way that brings objects of interest to a specific area in their retina (fixation area). Recording in various types of environments (aviary, office, outdoors) revealed significant statistical differences of low level image properties at the fixation area compared to values extracted at random image patches. These differences are in agreement with results obtained on primates in similar studies. To investigate the role of saliency and its contribution to drawing the owl's attention, we used a popular bottom-up computational model. Saliency values at the fixation area were typically greater than at random patches, yet were only 25% out of the maximal saliency value, suggesting a top-down modulation of gaze control.

# Abstract: Knowing left fromright: a combined ERP-fMRI study of visuospatial attention

**Authors:** H. Okon-Singer, I. Podlipsky, G. Yovel, T. Siman-Tov, T. Hendler

**Affiliations:** ^1^Functional Brain Imaging Unit, Wohl Institute for Advanced Imaging, Tel Aviv Sourasky Medical Center, Israel; ^2^Department of Psychology, Tel Aviv University, Israel

Right hemisphere (RH) specialization for spatial attention has been suggested in numerous studies (e.g.,Heilman, 1980; Mesulam, 1999). A recent functional magnetic resonance imaging (fMRI) study from our laboratory (Siman-Tov et al., in press) found higher activation for left-compared with right-visual field stimulation, in a bilateral attentional network. These findings suggest that the RH advantage relies on efficient recruitment of attention-related regions in both hemispheres, in contrast to the LH attentional network that is unilateral. One possible explanation for such advantage is faster neural conductivity from RH to LH, as has been shown in reaction time and evoked response potential (ERP) studies (Barnett and Corballis, 2005). In the current study, we used simultaneous recording of ERP and fMRI, in order to further investigate the neural basis for the RH advantage in spatial attention. Ten right-handed subjects were asked to ignore pictures of faces or geometric patterns. The pictures were presented for 200 milliseconds, in either the right or the left visual field, while subjects engaged a competing central task. Preliminary fMRI results replicated previous findings, showing activation in brain regions involved in visual processing and attention, such as the fusiform face area (FFA) and parietal areas, as well as higher activation for left visual field stimulation. Preliminary ERP findings reveal visual and attentional components, such as P1 and N170, and faster signal transfer from RH to LH, than vice versa. Further analysis will examine the correlation between the latency and amplitude of specific evoked potentials, such as N170, and activation in regions of interest, such as the FFA. Understanding the basis of attention asymmetry might help in formulating a neural model of lateralization in general. Furthermore, it may guide diagnosis and treatment of attention-related disorders, as well as other disease states characterized by abnormal lateralization.

# Abstract: Synchronized excitation and inhibition during spontaneous and sensory evoked responses in the rat barrel cortex

**Authors:** M. Okun, I. Lampl

**Affiliations:** Weizmann Institute of Science, Israel

The activity of neurons depends both on excitatory and inhibitory inputs, yet the relationship between the two remains poorly understood. In particular, a controversy exists regarding the extent of correlation between excitation and inhibition during spontaneous and sensory evoked activity in the cortex. In the present work we demonstrate a novel approach which allows to present adequate real-time picture of the correlation between excitatory and inhibitory inputs to cortical cells. It is based on simultaneous intracellular recording from pairs of nearby neurons that receive similar synaptic inputs. We have performed such recordings in the barrel cortex of halothane anesthetized rats (in which the spontaneous activity is similar to that observed in awake animals). A marked correlation was observed between excitatory inputs of the neurons (both cells were hyperpolarized, activity was dominated by EPSCs) and between inhibitory inputs (both cells were depolarized, activity was dominated by IPSCs). To estimate the correlation between excitatory and inhibitory inputs, one cell was depolarized and the other hyperpolarized (in both possible combinations). In this case a high (negative) correlation was found in all the pairs that were recorded, which implies that EPSCs and IPSCs of individual cells are also highly correlated. We also found that spontaneous inhibitory potentials lag behind excitatory potentials by several milliseconds. In addition to this high temporal coupling between the EPSPs and IPSPs, the amplitudes of the excitatory and inhibitory events were also highly correlated. A strong correlation was also observed between the amplitudes and the temporal patterns of excitatory and inhibitory synaptic inputs evoked by whisker stimulation. Our study provides the first direct indication that a strong coupling exists in the cortex between excitation and inhibition during both spontaneous activity and evoked responses.

*Supported by grants from the ISF and* *the National Institute for Psychobiology.*

# Abstract: Comparing the dynamics of ongoing activity in awake and anesthetizedmonkey

**Authors:** D. B. Omer, L. Rom, Y. Ulchin, A. Grinvald

**Affiliations:** Department of Neurobiology, Weizmann Institute of Science, Rehovot, Israel

Previous studies using Voltage sensitive dyes imaging on anesthetized cats reported that spontaneous ongoing cortical activity in the primary visual cortex represents dynamic spatial patterns many of which resembling the functional architecture of Orientation domains, and span large cortical areas (Grinvald et al., 1989; Arieli et al., 1995; Arieli et al., 1996; Tsodyks et al., 1999; Kenet et al., 2003; Fox et al., 2006; Fox et al., 2007b). Those results suggest that ongoing activity may play an important role in cortical processing and challenge the classical notion which considers spontaneous ongoing cortical activity as noise (Ferster D., 1996; Ringach D. L., 2003; Fox et al., 2007a). We preformed VSDI of ongoing cortical activity in the visual cortices of awake and anesthetized monkeys. Our results shows that in the anesthetized monkey spontaneous cortical activity shows larger repertoire of cortical states which resemble both Ocular Dominance domains as well as Orientation domains. We found large bias toward the representation of cortical states which resemble ocular dominance domains rather than orientation domains. In comparison to the spontaneous ongoing activity in the anesthetized monkey the dynamics of ongoing activity in the awake monkey is much faster, and the coherence-length is much smaller.

*Supported by the Daisy EU grant and the Goldsmith Foundation*.

# Abstract: The influence of NAP treatment on the severity of experimental autoimmune encephalomyelitis

**Authors:** S. Oz, I. Gozes

**Affiliations:** Human Molecular Genetics and Biochemistry, Sackler Medical School, Tel Aviv University, Israel

Activity-dependent neuroprotective protein (ADNP) was shown to be essential for brain formation (Brain Res. 2003 144:83). Peptide scanning identified a motif on ADNP, NAPSVIPQ (NAP) that provides femtomolar neuroprotection ([1]). Multiple sclerosis (MS) is a chronic disabling disease of the CNS. MRI studies in very early disease stages suggest that inflammation and neuronal injury are not strictly related ([2]). These experiments showed that significant axonal injury occurs early in the disease and that this is only indirectly linked to inflammatory activity. These studies suggested that treatment strategies for MS need to address both the inflammatory and neurodegenerative components of the disease, and that antiinflammatory therapies may only be able to control the inflammation-related process adequately ([3]). In order to assess the influence of NAP, a series of experiments on C57BL/6 female mice, were conducted, in which experimental autoimmune encephalomyelitis (EAE), a model for MS, was induced by immunization with a peptide derived from myelin oligodendrocyte glycoprotein (MOG). Intraperitoneal daily treatment withNAP that started 10 days after MOG immunization and continued for 17–47 days showed that the severity of the disease, represented by the degree of motor dysfunction was reduced in the NAP treated mice with dosages of 2 ± 1 *μ*g/animal. These results complement previous studies with intranasal and intravenous administration of NAP. BSF, ISF, Lily and Avraham Gildor Chair, Adams Super Center and Allon Therapeutics Inc.

## References

[1] Bassan M, Zamostiano R, Davidson A (1999). Complete sequence of a novel protein containing a femtomolar-activity- dependent neuroprotective peptide. *Journal of Neurochemistry*.

[2] (2003). *Neurological Sciences*.

[3] Chitnis T, Imitola J, Khoury SJ (2005). Therapeutic strategies to prevent neurodegeneration and promote regeneration in multiple sclerosis. *Current Drug Targets: Immune, Endocrine and Metabolic Disorders*.

# Abstract: Ladostigil reduces release of NO from activated microglia and oxidative stress in neuronal cells by differentmechanisms

**Authors:** R. Panarsky, M. Weinstock

**Affiliations:** Department of Pharmacology, School of Pharmacy, Hebrew University of Jerusalem, Israel

Ladostigil [Ethyl-methyl-carbamic acid (R)–3-prop-2- ynylamino-indan-5-yl ester], a cholinesterase (ChE) and monoamine oxidase inhibitor with neuroprotective activity, given orally for 2 weeks at a dose of 2.9 mumoles/kg, prevented microglial activation and the appearance of oxidative-nitrative stress in discrete brain areas induced by icv injection of streptozotocin. This dose was too small to inhibit either enzyme.

**Aim**

The aim is to determine whether ladostigil produced these effects by acting directly on microglia, and/or indirectly, by preventing neuronal damage. Methods: The effect of ladostigil and its active metabolites was measured on the release of NO induced by LPS (10 mug/ml) from mouse microglia, and on cytotoxicity induced by the tert-butyl hydroperoxide (TBHP) in SNKMC cells. The nitrite levels were determined spectrophotometrically with Griess reagent. The antiapoptotic effect of the compounds was measured by decrease in caspases 3/7 or prevention of the fall in the mitochondrial potential by JC1, and cell death, by trypan blue staining. Results: Ladostigil, its metabolites which lacked either the propargyl or the carbamate moieties or aminoindan itself at concentrations of 1 nM-1 muM reduced by almost 50% the release of NO from microglia. Higher concentrations were less effective. On the other hand, only ladostigil and metabolites containing a propargylamine group (100 nM-10 muM) significantly reduced apoptosis, the fall in mitochondrial potential and cell death induced by TBHP in SKNMC cells indicating that two different mechanisms were involved in these actions of ladostigil. Conclusion: The reduction of microglial activation and nitrative stress induced by a low dose of ladostigil in the rat brain can result from a direct action on microglia.

# Abstract: Synaptic strength regulated by astrocytic gap junctions

**Authors:** U. Pannasch, P. Ezan, K. Willecke, C. Giaume, N. Rouach

**Affiliations:** INSERM U840, Collége de France, 11 placeMarcelin Berthelot, 75005 Paris, France

Recent data suggest an active participation of astrocytes in neurotransmission by dynamic interactions with neurons. However the contribution of astrocytes may not only result from individual cells, but also from cellular networks, as they express numerous gap junctions. The aim of this work was to determine whether the connectivity of astrocytic networks participate in hippocampal neurotransmission and plasticity. Synaptic transmission was studied in Cx30 (one of the two gap junction proteins in astrocytes) knockout mice, whose astrocytic network connectivity is reduced by ∼50%. Field excitatory postsynaptic potentials in Cx30−/− mice are reduced by ∼35% compared to wild-typemice. This decrease is not due to changes in intrinsic properties and excitability of CA1 pyramidal neurons or postsynaptic receptor density. It is rather due to a decrease in glutamate synaptic concentration, as the frequency but not amplitude, of mEPSCs is decreased in Cx30−/− mice and the ratio of AMPA to NMDA synaptic currents is unchanged. The altered synaptic transmission is due to Cx30 deletion, as it is not observed in young mice (?P10), where Cx30 is not yet expressed. In addition, this effect is specific to glutamatergic transmission of CA1 pyramidal cells, as miniature IPSCs from pyramidal cells, as well as mEPSCs from interneurons, are not changed in Cx30−/− mice. The contribution of astrocytic networks to short and long-term synaptic plasticity was also studied. Paired-pulse facilitation was increased, whereas post-tetanic potentiation was reduced in Cx30−/− mice. Prolonged repetitive stimulation (10 Hz, 30 sec) led to an increased initial facilitation, followed by a slower synaptic depression. Furthermore longterm plasticity is also altered in Cx30−/− mice, as they show a 70% decrease in the amplitude of LTP. Altogether these results indicate for the first time that astrocytic gap junctions regulate neuronal excitatory transmission and plasticity.

*Supported by Human Frontier Science Program, Agence Nationale de la Recherche, French Ministry of Research.*

# Abstract: Membrane lipidsmodulation removes divalent open channel block of TRPL and NMDA channels

**Authors:** M. Parnas, B. Katz, S. Lev, V. Tzarfaty, A. Gordon-Shaag, B. Minke

**Affiliations:** The Hebrew University, Israel

Removal of divalent open channel block by depolarization plays a critical role in learning and memory, which is mediated by the N-methyl-D-aspartate (NMDA) channel. Other channels also exhibit open channel block, but the physiological mechanism of its removal is still unknown. We show here that lipids produced by phospholipase C (PLC) and hypoosmotic solutions remove divalent open channel block from the Drosophila Transient Receptor Potential-Like (TRPL) channels by bending the plasma membrane. Membrane bending increased single channel current and caused impermeable cation influx. The GsMTx-4 toxin, which specifically inhibits mechanosensitive channels, blocked the lipids effect. We found remarkable commonality between the effects of lipids on the Drosophila TRPL and the mammalian NMDA and TRPV3 channels. We suggest a new lipid-dependent mechanism to alleviate open channel block, which operates under physiological conditions, in synergism with depolarization. The profound effect of lipids modulation allows cross talk between channel activity and lipid-producing pathways.

# Abstract: Mapping and Eliminating FreeWater in the Brain by Diffusion Imaging

**Authors:** O. Pasternak, N. Sochen, N. Intrator, Y. Assaf

**Affiliations:** ^1^School of Computer Science, Tel Aviv University, Israel; ^2^Department of Applied Mathematics, Tel Aviv University, Israel; ^3^Department of Neurobiochemistry,Tel Aviv University, Israel; ^4^Functional Brain Imaging Unit, Tel Aviv Sourasky Medical Center, Israel

We offer a novel mathematical analysis for diffusion imaging data, which is able to separate between free water and the remaining brain tissue. In diffusion imaging we are able to magnetically trace the motion of water molecules. Properties of the motion depend on the surrounding tissue, and the amount of hindrance the molecules face. By analyzing those properties we are able to differentiate between tissue types (CSF, white matter and gray matter). We can also analyze the directionality of the tissue (for whitematter), which allows us to infer connectivity measures between different parts of the brain. The conventional way to analyze the diffusion properties of water molecules is named Diffusion Tensor Imaging (DTI), but this analysis fails to describe a tissue, which is in close proximity to free water. For healthy patients free water in the brain is found in the ventricles, and CSF filled cavities. Clinically, there are diseases and conditions that can be related with free water in the brain, especially in the form of edema. Edema is common, for instance, for patients suffering from brain tumors, stroke or other brain trauma. By applying our framework on such datasets we were able to map the relative volume of the free water compartment across the brain. Once the free water is mapped we can eliminate it in order tomeasure the properties of the remaining brain tissue. Using this method we have correctly identified white matter tracts that pass next to the ventricles, and more importantly were able to delineate the tract of white matter bundles that pass through edematous areas. This analysis allows better inference as to the condition of the tissue due to the edema.

*by the Edersheim-Levi-Gitter Institute*.

# Abstract: Does the right side remember what the left side learned? hemispheric transfer of perceptual learning effects

**Authors:** M. Pavlovskaya, S. Hochstein

**Affiliations:** ^1^Loewenstein Rehabilitation Hospital, Raanana, Sackler School of Medicine, Tel Aviv University, Israel; ^2^Life Science Institute and Neural Computation Center, Hebrew University, Jerusalem, Israel

When one side of the brain learns to perform a perceptual task, does the other side remember what has been learned? The expectation from receptive field size is that low levels in the visual hierarchy will be small, leading to specific local improvement, while higher levels will reflect generalized training, learned and remembered by all (Hochstein & Ahissar, Neuron, 2002). These tasks were always performed with test arrays in the center of the test screen. More recently, we introduced eccentric search arrays (Pavlovskaya et al., Spatial Vision, 2001), which were exploited later to test transfer of learning effects between hemispheres. We found transfer for easy tasks and not for hard tasks when subjects performed color and orientation feature search, each within one hemifield. However, the method used left an ambiguity that the transfer we found could be interpreted as transfer across tasks, and not between hemispheres. We now test the possibility of cross-hemisphere transfer by designing a paradigm whereby two independent tasks with very similar average degrees of difficulty are performed in the two hemifields. We trained subjects on a local detection (orientation pop-out task) in one hemifield and on global identification (vertical versus horizontal array orientation) in the other hemifield. Following training, we switched the sides of the tasks. We found transfer for the trained dimension for easy cases, suggesting that the transfer was due to a hemifield transfer. We confirmthe Reverse Hierarchy Theory that as long as the perceptual task is easy enough to be performed at high cortical levels, transfer will occur to new conditions that depend on the same, broad-spectrum, receptive fields. Results support cross-hemispere transfer for easy task conditions.

*Supported by National Institute for Psychobiology in Israel, USIsrael Binational Science Foundation*.

# Abstract: Parameters distributions and interrelations in compartmental models of neurons

**Authors:** N. Peled, A. Korngreen

**Affiliations:** ^1^The Mina and Everard Goodman Faculty of Life Sciences, Israel; ^2^The Leslie and Susan Gonda (Goldschmied) Multidisiplinary Brain Research Center, Israel

Parameters distributions and interrelations in compartmental models of neurons Noam Peled and Alon Korngreen The Mina and Everard Goodman Faculty of Life Sciences and The Leslie and Susan Gonda (Goldschmied)Multidisiplinary Brain Research Center, Bar-Ilan Univ., Ramat-Gan, Israel. Our understanding of the input-output function of single neurons has been advanced by biophysically accurate multicompartmentalmodels. The large number of free parameters in these models requires the use of automated approaches for finding their optimal values in a very multidimensional parameter-plane. Here we use genetic algorithm as a stochastic optimization algorithm. Due to inherent noise in measuring equipment and the stochastic nature of the neuron, the determination of the accuracy of the obtained parameters is near impossible. Here we show that finding the parameters distribution viaMonte Carlo simulations reveals the sensitivity of each parameter to noise. Furthermore, we calculate the averageHessian matrix over all the global minima to discover the interrelations between the parameters. To use a stochastic optimization algorithm a distance function must be declared, which calculates the distance between a given model output and the desire one. In this work we used several distance functions which are in used in this field. We compare between them, and discuss which can be suitable for such kind of a problem and which one not.

# Abstract: The representation of saccadic eye movements in human cortical regions

**Authors:** Y. Pertzov, G. Avidan, E. Zohary

**Affiliations:** ^1^Department of Neurobiology & Interdisciplinary Center for Neural Computation Hebrew University, Israel; ^2^Department of Psychology, Ben Gurion University of the Negev, Israel

It is well established that various brain regions, such as the frontal eye fields (FEF), supplementary eye fields (SEF), and the intraparietal sulcus (IPS), are involved in various aspects of eye movement planning and execution in both humans and primates. However, the reference frames in which information is represented in each of these areas is hotly debated. Specifically, it is unclear whether the direction of a saccade, its exact origin, final destination point (relative to the head, body or world) or combinations of these representations are multiplexed in these regions. Recently, new multivariate classification methods have been used to assess the reliability of distinctive spatial patterns of the fMRI responses (SPfR) in the visual cortex. We applied similarmethods in order to examine the spatial nature of representation of memory based saccades in cortical regions. Human subjects performed amemory saccade task in which equal amplitude saccades could be executed from several starting points and in a number of directions. We find that the SPfR in the FEF, SEF and IPS are positively correlated when the same saccades are repeatedly executed. The patterns become uncorrelated when saccades of the same magnitude and direction are executed from different starting points. These findings suggest that these regions all contain a neural representation which is sensitive to both the direction and starting point of the saccade and thus, the representation is not entirely oculucentric based, but rather, it is also coded in head, body or world coordinate frames.

# Abstract: Rehabilitation of cognitive impairment in patients with acute cerebellar stroke

**Authors:** D. Pierscianek, M. Frings, U. Bultmann, N. Fritsche, E. R. Gizewski, A. Flossdorf, D. Timmann, M. Maschke

**Affiliations:** ^1^Department of Neurology, University of Duisburg-Essen, Germany; ^2^Department of Diagnostic and Interventional Radiology and Neuroradiology, University of Duisburg-Essen, Germany; ^3^Department of Neurology, Fachklinik Rhein-Ruhr, Essen, Germany; ^4^Department of Neurology, Fachklinik Rhein-Ruhr, Essen, Germany

Introduction Previous studies suggested an impairment of executive functions in patients after cerebellar stroke. Other studies showed contradictory results. The specific role of the cerebellum in cognition is still unknown. The present study investigated cognitive deficits in the acute phase after cerebellar stroke, the recovery of possible symptoms and the effect of a cognitive training. Moreover, differences between the vascular territories, the lesion side and the involvement of cerebellar nuclei were examined. Methods Twelve patients with acute cerebellar stroke of the superior or of the posterior inferior cerebellar artery were matched with healthy controls and were examined by an extensive neuropsychological test battery in the acute phase, after two weeks and three months. Lesions were localised by magnetic resonance imaging in the acute phase and after three months. One half of the patients received a cognitive training, which was developed for patients with an impairment of executive functions after damage to the prefrontal cortex. Results A significant impairment in theWisconsin Card Sorting Test as a measure of action planning could be shown. Patients were also impaired in changing categories of a verbal fluency test (Regensburger Wortfluessigkeitstest) as a measure of cognitive flexibility. After three months differences were no longer significant. There was no difference between treated and untreated patients and no effect of the location of the stroke. Conclusions The present study suggests an impairment of executive functions in the acute phase after cerebellar stroke. These deficits could not be explained by disturbances of attention or visuospatial abilities or by depression. No effect of the neuropsychological training could be shown, possibly because of the small number of patients and a spontaneous recovery of symptoms. The effect of the cognitive training should be investigated in a larger group of patients.

*Supported by BMBF, German-Israeli* *Cooperation in Neuroscience (01GA0508)* *and IFORES (D/107-20540; D/107- 20550).*

# Abstract: Neuropeptides, Antidepressants and Depression

**Authors:** A. Pinhasov, M. Reichenstein, Y. Feder, J. Schneider

**Affiliations:** Department of Molecular Biology, Ariel University Center, Ariel, Israel

Depression is a highly prevalent, severe and often lifethreatening disorder. Despite the advances that have been made in the development of antidepressants, there are clearly still unmet clinical needs that should be addressed. To answer these needs, neuropeptide systems have started receiving increasing attention for the development of novel antidepressants. Pituitary Adenylate Cyclase Activating Polypeptide (PACAP) is a neuropeptide that exhibit central and peripheral effect via selective G-protein coupled receptors namely PAC1, VPAC1 and VPAC2. We are investigating involvement of PACAP and its receptors in the etiology of psychiatric disorders. Using primary rat enriched neuronal cell cultures we have demonstrated that acute (6 hours) and prolonged (48–72 hours) treatment with PACAP38 significantly up-regulates mRNA levels of Brain Derived Neurotrophic Factor (BDNF), the neurotrophin that is thought to be involved in the etiology of affective disorders and in the mechanism of action of antidepressants.. Our results have also revealed that expression of PACAP and its receptors are affected in primary cortical and hippocampal cell cultures treated for 72 hours with antidepressants representing distinct classes. Interestingly, changes in PACAP receptors expression resulting from antidepressant treatment were highly and negatively correlated with BDNF expression. To examine these findings in vivo, we administered imipramine (3 mg/kg) for 21 days to C57Bl6 mice. This chronic treatment resulted in a significant increase in PAC1 mRNA expression in hippocampus and a decrease in BDNF expression both in hippocampus and prefrontal cortex. Our findings suggest that antidepressants may influence synaptic plasticity via modulation of PACAPBDNF signaling and further understanding of these phenomena might contribute to the understanding of etiology of affective disorders and development of novel antidepressants.

# Abstract: Effects of neonatal administration of polyinosinic-polycytidilic acid (Poly I:C) on latent inhibition and discrimination reversal in adulthood

**Authors:** Y. Piontkewitz, M. Arad, I. Weiner

**Affiliations:** Department of Psychology. Tel-Aviv University, Israel

Association between maternal exposure to infection during pregnancy with increased liability to schizophrenia in the offspring has inspired the development of the prenatal immune challenge model of schizophrenia in which the synthetic cytokine releaser polyinosinic-polycytidilic acid (Poly I:C) is used to activate the maternal immune system. Adult offspring of poly I:C treated dams exhibit a wide spectrum of cognitive deficits reflective of schizophrenia, including selective attention abnormalities as measured in latent inhibition (LI) and discrimination reversal (DR). Importantly, the nature of deficits exhibited in the two tasks were reported to differ depending on the time of poly I:C challenge, with challenge early in gestation leading to attentional overswitching (disrupted LI and rapid DR) and late in gestation to attentional perseveration (intact LI and slow DR). We here tested the effects of neonatal poly I:C administration on these two tasks. Male rats were injected on postnatal day 4 with poly I:C (2mg/kg or 4mg/kg) and tested at adulthood in LI and DR. Neonatally treated rats had disrupted LI and slow DR superimposed on learning and/or performance deficit in both tasks. This deficit pattern may have relevance to chronic schizophrenia or autism. While present results support the notion that the long-term deleterious effects of poly I:C challenge depend on the timing of the challenge, comparison to another neonatal manipulation shows that the nature of the insult and not only its timing, determines the outcome.

# Abstract: Detection of differences in learning strategy and referencememory in young and old rats: Prevention of memory deficits by ladostigil

**Authors:** T. Poltyrev, C. Bejar, M. Weinstock

**Affiliations:** Department of Pharmacology, School of Pharmacy, Hebrew University, Jerusalemz, Israel

The aim of the study is to investigate memory and learning in aged rats in tests based on differential task demands and to assess the potential of ladostigil (TV-3326) (a novel drug with antiinflammatory activity)1 to prevent aged-related cognitive decline.

**Methods**

Place memory and detection of novelty in open field test (OF) were evaluated in male Wistar rats, aged 2.5months (YC, *n* = 10) and 15–16 months (AC, *n* = 24) in the course of habituation during 4 consecutive days followed by sudden change in this environment. Ten rats from AC were then randomly assigned for chronic oral treatment with ladostigil (1 mg/kg/day) till the end of testing (A-TV). To assess reference memory (RM) YC at the age of 3.5 months and aged rats at 19–20 months were trained in the holebox (HB) to search for food in 4 fixed positioned holes, marked by light. Spatial memory was also assessed one month later in the Morris Water Maze (MWM) test.

**Results**

At 16 months rats showed less exploratory activity than YC in OF but their response to novelty did not differ. At 20months the temporal organization of behavior in HB did not differ between AC and YC, but AC were much more dependent on the presence of visual cues, and less able to remember the position of all 4 baited holes than YC and A-TV. In MWM, AC demonstrated marked thigmotaxis and took much longer to escape onto the platform, even when close to it in contrast to YC and A-TV. AC had significantly more glial cells in the hippocampus and corpus striatum than YC, which were significantly reduced by ladostigil.

**Conclusions**

In both learning tasks it was possible to detect impairment of RM in AC aged 20-21 months. Daily treatment with ladostigil for 3–5 months from the age before learning deficits were detected, improved memory at the age of 21 months to resemble that of YC. The antiinflammatory effect of ladostigil may contribute to the prevention of age-related cognitive deterioration.

## References

[1] Shoham S, Bejar C, Kovalev E, Schorer-Apelbaum D, Weinstock M (2007). Ladostigil prevents gliosis, oxidative-nitrative stress and memory deficits induced by intracerebroventricular injection of streptozotocin in rats. *Neuropharmacology*.

# Abstract: Real-timemonitoring of blood-brain barrier disruption in the rat cerebral cortex

**Authors:** O. Prager, Y. David, A. Friedman

**Affiliations:** Departments of Physiology and Neurosurgery, Soroka Medical Centre,Ben-Gurion University, Israel

The blood–brain barrier (BBB) is designed to separate the brain's interstitial space from the blood. In both human and animal models there is still lack of reliable quantitative methods for studying the mechanisms underlying vessel permeability under healthy and disease conditions. We report here a new method for estimating BBB permeability based on analysis of fluorescent images of surface vessels in the rat cerebral cortex. Rats are anaesthetized and fixed under a fluorescent stereo-microscope. A cranial window is opened over the cerebral cortex and the dura is removed. During the experiment the brain is continuously superfused with artificial cerebrospinal fluid (ACSF). The animals are then injected intravenously (IV) with the albumin-binding, BBB nonpermeable dye Evans Blue (1 ml, 2%) or with Lucifer Yellow (1 ml, 10 mM). Fluorescent images of the cortical surface are collected at fixed intervals before, during and following dye injection to identify blood vessels and to follow changes in fluorescent intensity within the brain parenchyma. To disrupt the BBB brain was perfused with the bile-salt sodium deoxycholate (DOC, 2 mM), Na^+^/K^+^ ATPase inhibitor, Ouabain (5 uM), the GABA receptor antagonist, penicillin (100 U/uL) or by electrical stimulation of the SPG. Laser Doppler flowmetry and EEG are simultaneously recorded over the surface of the treated region to monitor brain perfusion and neuronal activity, respectively. Vessels diameters and BBB permeation are measured offline using homemade MATLAB scripts. The analysis entails vessels identification following dye injection under control conditions, followed bymeasurements of changes in extravascular fluorescent intensity. Histological analysis was assessed following the in-vivo experiment to confirm BBB opening.The presented method offers a new tool to study mechanisms underlying alterations to brain vascular characteristics including vessels diameter and permeability under normal and pathological conditions.

*This work was supported* *partly by “BrainsGate.”*

# Abstract: Sustained image presentation supports a role for gamma LFP in object perception

**Authors:** E. Privman, L. Fisch, U. Kramer, S. Kipervasser, F. Andelman, M. Y. Neufeld, Y. Yehezkel, I. Fried, R. Malach

**Affiliations:** ^1^School of Computer Science, Sackler Faculty of Exact Sciences, Tel Aviv University, Tel Aviv, Israel; ^2^Department of Neurobiology, Weizmann Institute of Science, Rehovot, Israel; ^3^Functional Neurosurgery Unit, Tel Aviv Medical Center, Tel Aviv, Israel; ^4^Electroencephalography and Epilepsy Unit, Tel Aviv Medical Center, Tel Aviv, Israel; ^5^Sackler School of Medicine, Tel Aviv University, Tel Aviv, Israel

A fundamental issue in the study of the human visual system concerns the neuronal dynamics that may underlie the emergence of a visual percept. In local field measurements a number of putative signals have been described as related to visual processing including transient potentials (ERP), delta waves, alpha-beta power disynchronizaiton (ERD) and gamma power modulations. A striking characteristic of human perception is the ability to maintain a conscious visual percept for an extended period of time. Here we employed this phenomenon to identify putative candidates for object perception. Recording was done using sub-dural electrodes that were implanted in patients with epilepsy strictly for clinical purposes. During recordings subjects were exposed to images of objects lasting 2 seconds. The task of the subjects was to report contrast changes in the images. Our results point to gamma power as the most consistent electrophysiological marker whose activity was maintained in close correlation with the perceptual state of the subjects. Another clear cut electrophysiological signal whose activity wasmaintained throughout the percept was ERD. In contrast to the gamma enhancement, this signal showed a much broader tuning to various image properties.

*Supported by ISF grant to R. Malach and US* *Israel BSF grant to Malach and Fried.*

# Abstract: Behavioral and cortical activation patterns following an optic neuritis episode

**Authors:** N. Raz, S. Dotan, E. Zohary, D. Arkadir, S. Chokron, N. Levin

**Affiliations:** ^1^Department of Neurology, Hadassah Hebrew-University Hospital, Jerusalem, Israel; ^2^Department of Ophtalmology, Hadassah Hebrew-University Hospital, Jerusalem, Israel; ^3^Department of Neurobiology, Hebrew University, Jerusalem, Israel; ^4^Service de Neurologie, Fondation Ophtalmologique Rothschild, France

Optic neuritis (ON) is a common condition that causes reversible loss of vision in young patients. It derives from idiopathic inflammatory demyalination. There is an ongoing debate about whether ON derives from parvocelluar or magnocelluar deficit. Visual function improvement is common in this disease, and is usually attributed to peripheral nerve recovery. Our previous study has suggested that central reorganization processes, that is, the lateral occipital complex (LOC), may also be involved in recovery. The aim of the present work is to study the behavioral and cortical changes taking place following an ON episode, in a longitudinal study. We have found that, after the acute phase, ON eyes had normal or near normal visual acuity, contrast sensitivity and color perception; however, they still had difficulties in recognizing objects defined by motion. This may support a specific magnocelluar deficit in ON. In order to study a possible cortical origin for the behavioral results, we have studied fMRI activation pattern in the ventral and dorsal pathways. While the former may be more concerned with visual acuity and is mainly considered to be stimulated by the parvocelluar system, the latter may contribute to motion perception and is mainly stimulated by the magnocelluar system. We have compared the patterns of fMRI activation elicited by stimulation of the affected and the healthy eyes, along the visual cortical hierarchy. As previously, we found reduced fMRI activation in V1 during stimulation of the affected eye compared with the healthy eye. However, the magnitude of difference in fMRI signal decreased with progression along the visual hierarchy, both in objects and motion related areas, suggesting cortical adjustment to abnormal input. Those preliminary fMRI results, however, did not support a different role for the affected eye in ventral and dorsal pathways.

*Supported by The Caesarea Edmond* *de Rothschild foundation.*

# Abstract: Auditory and visual responses in the tectofugal pathway of the barn owl

**Authors:** A. Reches, Y. Gutfreund

**Affiliations:** Department of Physiology and Biophysics, The Faculty of Medicine, The Technion, Israel

The entopallium (E) in birds is the major thalamorecipient forebrain structure of the tectofugal pathway The E receives most of its input from the nucleus rotundus, the avian homolog of the mammalian thalamic pulvinar complex . Previous research has characterized the tectofugal pathway in mammals and pigeons as an extra geniculate pathway leading visual information to the cortex. This pathway has been suggested to be involved in bottom-up attentional control. We show that, in the barn owl, entopallial cells respond to auditory as well as visual stimuli, suggesting that the tectofugal pathway is not strictly visual but carries multisensory information. We characterized single unit responses in the E to binaural localization cues including interaural time difference (ITD) and interaural level difference (ILD), as well as average binaural sound intensity (ABSI). Similar to visual receptive fields, auditory receptive fields were typically large and covered most of the contralateral space. In addition, a majority of the cells presented nonmonotonic rate level functions with a preference towards weak intensity sounds. Interestingly, most multisensory neurons in the E responded stronger to spatially aligned visual and auditory stimuli than to the sum of the responses to the unimodal components (superadditive enhancement). Finally, we show that the bimodal enhancement is context dependent appearing more robustly in responses to rare stimuli, a result that is in-line with the notion that the E is involved in multisensory control of spatial attention.

*This work was supported by a grant from the* *National Institute for Psychobiology in Israel and a BIK.*

# Abstract: Effect of antidepressants on PACAP and its receptors in rat primary neurons

**Authors:** M. Reichenstein, M. Rehavi, A. Pinhasov

**Affiliations:** ^1^Department ofMolecular Biology, Ariel University Center,Ariel, Israel; ^2^Department of Physiology and Pharmacology, Sackler Faculty of Medicine, Tel Aviv University, Israel

According to the monoaminergic hypothesis of depression, most of the antidepressants are aimed to stimulate monoamine systems. In spite of their predominant effect on monoamine neurotransmitter levels, these drugs may also mediate changes in synaptic plasticity by modulating neuropeptide action in brain areas, such as hippocampus and prefrontal cortex. Pituitary Adenylate Cyclase Activating Polypeptide (PACAP) is a neuropeptide exerting pleiotropic effects via three types of receptors, PAC1, VPAC1 and VPAC2. We have investigated the effects of various antidepressants on the expression of PACAP, its receptors and brain derived neurotrophic ffactor (BDNF), a neurotrophin implicated in the mechanism of action of antidepressants. Using a real-time PCR analysis, we found that selective serotonin reuptake inhibitors, Paroxetine and Citalopram, significantly and dose-dependently decreased PAC1 and VPAC2 mRNA levels following 72-hour treatment, whereas tricyclic antidepressant Imipramine exhibited an opposite effect. No effect on VPAC1 expression was observed. Furthermore, we found an intriguing correlation between the changes in PAC1/VPAC2 and BDNF expression after treatment with antidepressants. In addition, we examined the effect of these antidepressants on neuronal cell survival, using a cell proliferation assay based on metabolic activity. Incubation of cortical neurons for 24 hours with antidepressants (0.3– 10 *μ*M) had no effect on metabolic activity and cell viability. Incubation for 72 hours increased cell metabolic activity with the exception of Paroxetine and Imipramine at 10 *μ*M that proved to be cytotoxic. Cotreatment with antidepressants (3– 10 *μ*M) and PACAP or it's antagonist PACAP(6– 38) was ineffective in cells exposed to Paroxetine 10 *μ*M. Moreover, co-treatment with PACAP and Imipramine 3– 10 *μ*M had an adverse effect on cell viability. In summary, our results point toward PACAP signaling involvement in synaptic plasticity processes mediated by antidepressants.

# Abstract: Amidic GABA derivatives of antidepressants display improved pain suppressing activity

**Authors:** A. Rephaeli, I. Gil-Ad, N. Trasenko, I. Tarasenko, A. Aharoni, Y. Geffen, K. Savitsky, A. Weizman, A. Nudelman

**Affiliations:** ^1^Felsenstein Medical Research Center, Tel-Aviv University, Israel; ^2^Department of Chemistry, Bar Ilan University, Ramat Gan; ^3^BioLineRx Ltd., Jerusalem, Israel

Inadequate pain management, which is widely prevalent, raises the urgent need to develop new and improved drugs. Some antidepressants, antiepileptics and GABAergic drugs are used for management of pain syndromes. To improve the activity of these agents, GABA derivatives of GABA, valproic acid, nortriptyline and fluoxetine, were synthesized and tested in animal models of pain. In the hot plate model, the GABA esters of GABA (AN-214) or valproic acid (AN-216) did not elicit a significant effect. The GABA amides of the antidepressant nortriptyline, BL1021, or fluoxetine, BL1024, significantly increased the latency of nociceptive reaction of Balb/c mice to heat and displayed significantly higher analgesic effects than the parent antidepressants. Intraplantar injection of formalin to Balb/cmice resulted in a typical biphasic flinching behavior of the injected paw. In this model BL1021 and BL1024 significantly reduced the early nociceptive and the late inflammatory peripheral responses compared to equimolar po doses of nortriptyline, fluoxetine, or equimolar mixtures of the antidepressants and GABA or gabapentin. Skin samples from the injected paws, exhibited significantly lower levels of interferon- gamma and TNF-alfa in mice treated with BL1021 compared to those treated with nortriptyline, and to the mixture of nortriptyline and GABA or gabapentin. InWistar rats injected intraplantarly with carrageenan, BL1021 showed significantly better efficacy in inhibiting edema, than those treated with nortriptyline. In addition, a significantly increased latency of nociceptive reaction by these rats was demonstrated using the hot-plate-test. The notably better efficacy of the amides compared to their parent drugs towards both the central perception and the peripheral phase of pain, make them promising candidates for the treatment of acute and neuropathic pain conditions.

*This research was partially* *supported by BioLineRx.*

# Abstract: Associative odor learning induces non uniform increase in the synaptic efficacy

**Authors:** I. Reuveni, D. Saar, E. Barkai

**Affiliations:** Department of Neurobiology and Ethology, Faculty of Sciences, Haifa University, Israel

We have previously shown that rule learning of an olfactory discrimination task is accompanied by increased synaptic efficacy in the piriform cortex. In the current study we investigated the quality of this change by recording spontaneous excitatory post synaptic currents (sEPSC) in piriform cortex pyramidal neurons. The averaged amplitude of the sEPSCs in neurons from trained rats (13.5 ± 6.5 pA, *n* = 19) was 55% larger (*P* < .002) than the averaged sEPSCs in neurons from pseudo-trained (8.7 ± 1.7, *n* = 14) and naïve rats (9.2 ± 4.5, *n* = 15), which had similar values. Principal component analysis (PCA) of the amplitude distributions was applied to further characterize the nature of the change. PCA showed that the variability between cells resulted from two different components: (1)Homogenous change of all sEPSCs in a given cell. (2) The proportion of large versus small spontaneous events. Such analysis showed homogenous increase of around 1.5 pA in all synapses in pseudo-trained compared to naïve (*P* < .02). Such increase was not paralleled with increase in the averaged amplitude of spontaneous events due to an statistically insignificant change in the proportion between large (half width at 7–12 pA) and small (half width at 2–5 pA) events. In contrast, in neurons from trained rats, when compared with pseudo-trained, only a fraction of the small sEPSCs (3.75–7.25) in each cell was substituted by very large sEPSCs (10–25pA). The size of this fraction varied between cells and ranged between 0–100% of the small sEPSCs in the averaged pseudo-trained cell. Our data show that while the change between the pseudo-trained and the naïve groups could be described as a uniform change over the whole population of cells and synapses, the change between the trained and the pseudo-trained group is apparent in only fraction of synapses and to a different extent in each cell, imposing a fundamental change in the information processing and coding within the piriform cortex network.

# Abstract: The Neurorestorative Effect of the Green Tea Polyphenol (-)-Epigallocatechin-3-Gallate Combined With the Selective MAO-B Inhibitor, Rasagiline in MPTPMice Model

**Authors:** L. Reznichenko, M. B. H. Youdim, S. A. Mandel

**Affiliations:** Eve Topf Centers, Department of Pharmacology, Faculty of Medicine, Technion, Haifa, Israel

The neuropathology of neurodegenerative diseases is associated with a gradual loss of neurons in the respective affected brain areas. Our previous studies have shown that the green tea polyphenol (-)-epigallocatechin-3-gallate (EGCG) prevented neuronal cell death, both in vivo and in vitro, as well as revealed a neurorescue effect against several toxinsinduced cell death. It has been clearly demonstrated that a “domino” cascade of neurotoxic complex events can initiate neurodegeneration in Parkinson's (PD) and Alzheimer's diseases (AD). Thus, it is assumed that a combination of well established neuroprotective drugs, such as antioxidants and iron chelators (e.g., EGCG), and neurotrophic drugs (e.g., rasagiline), will be amore effective therapy for these diseases. In the present work, EGCG alone (5 mg/kg/day, 14 days) and in combination with rasagiline (0.05 mg/kg/day, 14 days) has been studied in vivo, using neurorescue paradigm of MPTP model of PD. The drugs were administered orally to mice C57/Bl, 4 days after MPTP (4 × 20 mg/kg/day). The MPTP treatedmice demonstrated a progressive demise of dopaminergic neurons in substantia nigra (SN) (assessed by immunohistochemistry), followed by significant decrease in the striatal dopamine (DA) content (assessed by HPLC), as previously reported by us. However, EGCG alone, and more importantly, the cocktail of EGCG plus rasagiline, significantly rescued cell function of SN dopaminergic neurons, indicated by elevation of the DA content in the striata. Additionally, the administration of these drugs significantly attenuated cell death of SN DA-containing neurons, as demonstrated by tyrosine hydroxylase-cell labeling. The present findings indicate that EGCG alone and in combination with rasagiline may have a positive impact on aging and neurodegenerative diseases to retard or perhaps even reverse the accelerated rate of neuronal degeneration.

# Abstract: Microsomal enzymesmetabolize the endogenous lipid, N-arachidonoyl dopamine, to hydroxylated metabolites active at recombinant human TRPV1 receptors

**Authors:** N. Rimmerman, H. B. Bradshaw, B. Tan, J. M. Walker

**Affiliations:** Psychological and Brain Sciences and the Gill Center for BioMolecular Sciences, Indiana University, Ind, USA

N-arachidonoyl dopamine (NADA) is an endogenous lipid identified in our lab, which modulates signal transduction in nociceptive pathways (Huang et al., 2002). NADA is found in the mammalian nervous system and activates the nonselective cation channel, transient receptor potential vanilloid type 1 (TRPV1). NADA also activates cannabinoid receptor 1 and inhibits the NF-kappa B, NFAT and Activator Protein 1 signaling pathways. To investigate the metabolism of NADA through the cytochrome P450 (CYP450) metabolic pathway, we studied the in vitro production of hydroxylated metabolites and their activity at recombinant human TRPV1 receptors. Method: Rat liver and brain microsomal fractions were isolated using differential centrifugation. The microsomal metabolites of NADA were partially purified from methanolic extracts on solid phase cartridges. Metabolites were were identified by HPLC/quadrupole time-of-flight mass spectrometry and quantified tandem mass spectrometry. Major hydroxylated metabolites were tested for calcium influx on human recombinant TRPV1 receptors overexpressed in human embryonic kidney cells (HEK293). Key results: Following microsomal activation in the presence of NADA, omega and (omega-1) hydroxylated metabolites were formed. These hydroxylated NADA metabolites were active at recombinant human TRPV1 receptors, inducing a dosedependent calcium influx (EC50: 1.3, 1.2microMolar, respectively). Both metabolites exhibited lower potency and efficacy compared to NADA. Conclusions and implications: CYP450 enzymes are capable of metabolizing several fatty acids (e.g., arachidonic acid) and fatty acid-containing lipids (e.g., N-arachidonoyl ethanolamine) forming a larger family of neuromodulators with potential activity at various lipid receptors. The current study shows that NADA metabolism occurs through microsomal enzymes. Future studies will examine the antiinflammatory activity of these metabolites.

*Supported by NIDA (DA-018224, DA-020402, F32-DA-016825)*.

# Abstract: Low-pass filter properties of basal ganglia (BG) networks and possiblemechanisms of action of high-frequency deep brain stimulation (DBS)

**Authors:** M. Rivlin-Etzion, O. Marmor, B. Rosin, M. Slovik, H. Bergman

**Affiliations:** Physiology and ICNC, The Hebrew University, Jerusalem, Israel

Oscillatory bursting activity is commonly found in the BG of the Parkinsonian brain. The frequency of these oscillations is often similar to Parkinson's disease (PD) tremor, but their relationship to the tremor and other Parkinsonian symptoms is still under debate. We studied the frequency-dependency of information transmission in the BG networks by recording simultaneously from multiple electrodes located in the primary motor cortex (MI) and in the globus pallidus (GP) of two vervet monkeys before and after MPTP treatment and induction of PD symptoms. We mimicked the PD bursting oscillations by stimulating at different frequencies (1–15 Hz) through micro-electrodes located in MI or GP. Since the bursts used in this study contained 8 pulses given at 200 Hz, the high (10,15 Hz) frequency, 20 seconds long, tests can be depicted as fragmented-DBS pattern, enabling us to examine the effects of high frequency DBS without the distortion of stimulation artifact. Our findings show that in the normal state, micro-stimulation of one structure does not modulate the discharge rate in the other structure. The functional connectivity between MI and GP is greatly enhanced following MPTP treatment. In the frequency domain, GP neurons usually responded equally to 1–15Hz stimulation bursts in both states. By contrast, MI neurons expressed low-pass filter properties, with a cut-off frequency around 5 Hz. Finally, muscle activation evoked by MI micro-stimulation was markedly attenuated at frequencies higher than 5 Hz. The low-pass properties of the BG networks suggest that despite their similar frequencies-PD tremor is not directly driven (beat to beat) by the BG 5–10 Hz burst oscillations. BG high frequency stimulation would similarly be filtered in the cortex or in previous level. We therefore suggest that DBS alleviates the PD symptoms by blocking the abnormal oscillations of the BG networks and re-establishment of downstream to the BG compensatory processes.

# Abstract: Cerebellar cortical responses to mossy fiber repetitive stimulation are enhanced by dissinhibition

**Authors:** D. Rokni, Y. Yarom

**Affiliations:** Department of Neurobiology, and ICNC, Hebrew University, Jerusalem, Israel

Cerebellar Purkinje cell (PC) responses to mossy fiber (MF) stimulation, are composed of a fast excitatory component followed by prolonged inhibition. It was suggested that the inhibitory component, generated by the molecular layer interneurons (MLIs), limits the time window for synaptic integration, and ensures a precisely timed output (Mittmann et al. 2005). However, it was previously shown that a single stimulus to the parallel fibers (PFs) inactivates the inhibitory component for tens of milliseconds (Cohen and Yarom 2000, Mann-Metzer and Yarom). Thus, inputs that are composed of high frequency spike trains (Chadderton et al. 2004), are likely to inactivate the inhibitory component. To asses the role of the MLIs in shaping the time course and spatial organization of MF responses, we used the voltage sensitive dye RH-414, and imaged responses to stimulation of MFs in the isolated cerebellum of a Guinea pig. We confirmed that the responses to MF stimulation are spatially organized in a radial patch occasionally bounded by measurable inhibitory responses. We found that this inhibition which exists throughout the responding patch, shapes the time course but not the spatial organization of MF responses. Responses were prolonged when either (1) GABAergic transmition was blocked (GABAzine 5 *μ*M), (2) MF stimulation was preceded by activation of the MLIs by surface stimulation, (3) MF stimulation was preceded by anotherMF stimulation (repetitive stimulation). However, no further prolongation of the responses in conditions 2 and 3 occurred in the presence of GABAzine. This indicated that prolongation of the responses in these conditions was due to a lack of the inhibitory component, and not a change in the excitatory component. When MLIs receive input from MFs they inhibit other MLIs thus preventing them from contributing to following MF inputs. Therefore, the possibility that the MLIs act to limit the time window of PC integration is of limited significance.

# Abstract: Toll-like receptors regulate adult neurogenesis

**Authors:** A. Rolls, R. Shechter, A. London, Y. Ziv, A. Ronen, R. Levy, M. Schwartz

**Affiliations:** Department of Neurobiology, Weizmann Institute of Science, Israel

Neurogenesis—the formation of new neurons in the adult brain—is considered to be one of the mechanisms by which the brain maintains its lifelong plasticity in response to extrinsic and intrinsic changes. The mechanisms underlying the regulation of neurogenesis are largely unknown. We discovered that Toll-like receptors (TLRs), a family of highly conserved pattern-recognizing receptors involved in neural system development in Drosophila and innate immune activity in mammals, regulate adult hippocampal neurogenesis. We show that TLR2 and TLR4 are found on adult neural stem/progenitor cells (NPCs) and have distinct and opposing functions in NPC proliferation and differentiation both in vitro and in vivo. TLR2 deficiency in mice impaired hippocampal neurogenesis, whereas the absence of TLR4 resulted in enhanced proliferation and neuronal differentiation. In vitro studies further indicated that TLR2 and TLR4 directly modulated self-renewal and the cell-fate decision of NPCs. The activation of TLRs on the NPCs was mediated via MyD88 and induced PKCalpha/beta-dependent activation of the NF-kappaB signalling pathway. Thus, our study identified TLRs as players in adult neurogenesis and emphasizes their specified and diverse role in cell renewal. These results were recently published in Nature Cell Biology in the cover and were accompanied by News and views by Marino and Pluchino. It was also chosen as editor's choice in Science STKE.

*This study was supported, in part, by an NRSAD* *award and by a research grant from Mr and Mrs Richard D. Siegal.*

# Abstract: Searching for anatomical based functional organization in the human brain: The case of the Supplementary Motor Area

**Authors:** K. Rosenberg, I. Fried, T. Hendler

**Affiliations:** ^1^Wohl Institute for Advanced Imaging, Tel-Aviv Sourasky Medical Center, Israel; ^2^Epilepsy and Functional Neurosurgery Unit, Tel-Aviv Sourasky Medical Center, Israel; ^3^Sackler Faculty of Medicine, Physiology department, Tel-Aviv University, Israel

A close relation between structural and functional features in the brain is a long-standing axiom, albeit its mechanism is poorly understood. The Supplementary Motor Area (SMA), a brain area in the medial wall of Brodmann's area 6, has been subdivided based on cytoarchitectonic features into posterior and anterior parts: SMA proper and pre-SMA, respectively. However, the functional relevance of these subdivisions has not yet been fully revealed. The location of the SMA between areas involved in executive and cognitive aspects of action in the prefrontal cortex points to a possible in-between representation of functional hierarchy within it. On the whole, animal and human studies suggested the involvement of the SMA in processing voluntary aspects of action that underlie self-generated processes such as volition and planning. The goal of our study is to uncover principles of functional organization in the human SMA. For that purpose we will use fMRI on healthy subjects with paradigms that differ in their mode of action (language versus motor), planning load (simple versus complex) and the source of act initiation (internal versus external). Based on these tasks, we will establish an SMA-selective functional localizer. Thirteen healthy subjects participated in the study. The “complex” versus “simple” condition activated the SMA proper, while the “internally guided” versus “complex” condition activated the Pre-SMA. The results demonstrated functional organization within the SMA area with the pre-SMA more related to the complex and internally guided acts then the SMA proper.

*This research was supported by the Adams* *Super Center for Brain Studies.*

# Abstract: The contribution of forced oscillations of the arm to the discharge pattern of primary motor cortex (M1) and globus pallidus (GP) neurons

**Authors:** B. Rosin, M. Slovik, R. Mitelman, M. Rivlin-Etzion, H. Bergma

**Affiliations:** ^1^Department of Physiology, Hebrew University-Hadassah School of Medicine, Jerusalem, Israel; ^2^The Interdisciplinary Center for Neural Computation, Hebrew University, Jerusalem, Israel; ^3^The Eric Roland Center for Neurodegenerative Diseases, Hebrew University-Hadassah School of Medicine, Israel

Low-frequency tremor is one of the pivotal symptoms of Parkinson's disease (PD). In parallel, the firing pattern of the basal ganglia (BG) and cortical neurons becomes bursty and, in part, oscillatory and synchronized. Albeit the coappearance of the tremor and the changes in discharge pattern suggests a connection between them, their interrelation remains illusive. Either one of the phenomena could cause the other, both could be caused by some unknown confounding factor and, finally, they could be completely unrelated and caused by different aspects of the disease. In this study we examined the contribution of sensory feedback paths to the discharge patterns of GP and M1. We have recorded from the arm related area of M1 and the GP of a healthy African green monkey (Cercopithecus aethiops), while applying forced oscillations (artificial tremor) to the left arm at several discrete frequencies for a duration of 90 seconds per frequency. We report the following results: 60/152 (39%) of the M1 neurons displayed a discharge pattern that was phase locked and coherent to the movement induced by the experimental procedure. The coherence showed a tendency to increase with the increase in movement frequency and amplitude, perhaps suggesting reaction to acceleration rather than to movement frequency. 12/62 (19%) of neurons in the GPe showed a discharge pattern that was phase locked and coherent with the movement. Interestingly, the maximal coherence in the majority of neurons was achieved at 5 Hz, which is coincidently the frequency of the PD tremor. The phase locking did not seem to be affected by amplitude changes, suggesting that the reaction was indeed to movement frequency rather than to acceleration. Our results imply that PD tremor is probably not the cause of the change of discharge patterns of M1 and GP neurons in MPTP-treated primates. Future recordings in the GPi and in the MPTP-treated primate model of PD will be performed to validate this conclusion.

# Abstract: Magnetic stimulation of living and dissociated brains

**Authors:** A. Rotem, E. Moses

**Affiliations:** Physics of Compex Systems, Weizmann Institute of Science, Israel

Magnetic stimulation of nerves is attracting increased attention recently, as it has been found to be useful in therapy of neural disorders in humans. In an effort to uncover the mechanisms of magnetic stimulation we apply magnetic stimulation on ex-vivo neuronal preparations and demonstrate the first magnetically evoked activity in cultures. Magnetic pulses initiate neuronal activity only in 1D patterned cultures of hippocampal and cortical neurons. The sensitivity of neurons to the magnetic pulses is affected by coil orientation, geometry, morphology, pharmacology, neural activity and neuronal density. Reciprocally, magnetic stimulation, with either single or repetitive pulses, has an obvious effect on immediate and progressive neuronal activity. In a related experiment the interaction between myelination and TMS is explored by applying TMS on Wistar and Myelin Deficient (MD) rats and by magnetically stimulating myelinated cultures. Intriguing findings regarding TMS on rats in general and differences between myelinated and non myelinated systems will be described. These findings may have an impact on the way TMS research is conducted on humans.

# Abstract: Differential postsynaptic efficiency of electrosensory afferents and parallel fibers in the principal neurons of the shark dorsal octavolateral nucleus

**Authors:** N. Rotem, E. Sestieri, J. Hounsgaard, Y. Yarom

**Affiliations:** ^1^Neurobiology Deparment, Institute of Life Science, Hebrew University of Jerusalem, Israel; ^2^Otto Loewi Minerva Center, Inter-University Institute for Marine Biology, Eilat, Israel; ^3^Department of Medical Physiology, Panum Institute, University of Copenhagen, Denmark

Learning to predict the sensory component resulting from the organism's own activity enables it to respond appropriately to unexpected stimuli. In sharks, predictable electric fields of the electrosensory sense are filtered in the principal neuron of the Dorsal Octavolateral Nucleus (DON). Postsynaptic interactions between the electro-sensory primary afferents (aff) and parallel fibers (pf) are the proposed underlying mechanism. We used the isolated brain stem of the shark to characterize these two inputs. Intracellular recording and labeling ensure that our study in confined to the principal neuron and application of extracellular field ensure voltage modulation at remote areas. We found that: (a) the aff postsynaptic potential (EPSP) has a faster rise time (3.9 ± 1.8 ms), low amplitude (2.9 ± 1.74 mV) and short duration (17.4 ± 7.8 ms); (b) facilitation was never encountered; (c) threshold for action potentials was 3.5 mV when evoked by aff stimulation. (d) The EPSP was insensitive to change in postsynaptic potential, induce by intracellular current injection or by applied extracellular fields. Hence, the aff input is located close to the spike initiation site and the EPSP has the characteristics of electric transmission. The pf input is characterized by (a) EPSP had a slow rise time (8.8 ± 3.5 ms), a high maximal amplitude (12 ± 5.58 mV) and long duration (36 ± 15.1ms); (b) prominent facilitation was elicited by short train of stimuli; (c) threshold for action potential was 20 mV when evoked by pf stimulation. The highly efficient connection between the afferent input and the principal neuron leaves little room for summation with parallel fibers response. Hence the filtering is most likely mediated via secondary neurons that adjust the sensitivity to afferent input.

# Abstract: Microglia activation is subject tomodulation in injury and disease

**Authors:** S. Rotshenker

**Affiliations:** Department of Anatomy and Cell Biology, Faculty of Medicine, Hebrew University, Israel

The removal of degenerated myelin is essential for repair by regeneration after injury to axons and for minimizing damage to intact axons and myelin in multiple sclerosis. Microglia remove degenerated myelin by phagocytosis. We focus presently on complement receptor-3 (CR3) and scavenger receptor-AI/II (SRA) mediated myelin phagocytosis in microglia. Paradoxically, these receptors are expressed in microglia after injury to CNS axons but myelin is not phagocytosed, suggesting that phagocytosis can be modulated between efficient and deficient states. We sought after mechanisms that modulate myelin phagocytosis and shall presently discuss three that involve Galectin-3 (Gal-3), cAMP and Rho/Rock. First, Gal-3 is expressed in microglia that phagocytose but not in microglia that do not phagocytose myelin. Additionally, Gal-3 stabiles K-Ras-GTP that activates PI3K which, in turn, activates phagocytosis. An explanation is offered, thereby, for efficient and deficient phagocytosis by microglia that, respectively, express or do not express Gal-3. Second, cAMP plays a dual role in phagocytosis in a concentration dependent manner: it activates phagocytosis at normal levels through PKA and inhibits phagocytosis at high levels through PKA and Epac. Thus, phagocytosis is inhibited by increasing cAMP levels pharmacologically. Third, Rho/ROCK signaling activates the production of stress fibers and downregulates filopodia formation. Conversely, the inhibition of Rho/ROCK signaling disrupts stress fibers and upregulates filopodia formation along with augmenting phagocytosis, thus suggesting that the three are causally linked. Notably, the latter two mechanisms are of special interest with respect to regeneration in view of the observations that elevated cAMP levels and Rho/ROCK inhibition promote regeneration by overriding growth-cone collapse induced by degenerated myelin.

*Supported by Israel Science Foundation and the Ministry of Health*.

# Abstract: Dopaminergic Neuronal Cluster Size is Determined During Early Forebrain Patterning

**Authors:** N. Russek-Blum, A. Gutnick, H. Nabel-Rosen, J. Blechman, N. Staudt, R. Dorsky, C. Houart, G. Levkowitz

**Affiliations:** ^1^Department ofMolecular Cell Biology, Weizmann Institute of Science, Rehovot, Israel; ^2^Medical Research Council Center for Developmental Neurobiology, King's College, London, UK; ^3^Department of Neurobiology and Anatomy, University of Utah, Salt Lake City, Utah, USA

The population size of distinct nerve cell types is determined by intracellular programming that is later modified by extracellular signals. Uncovering developmental cues that regulate the nearly fixed number of dopaminergic (DA) neurons is important for understanding the contribution of aberrant DA development to neurological disorders. Although the physiological and clinical aspects of the DA system have been intensively studied, relatively little is known about the lineage of DA cell clusters and the mechanisms controlling their progenitors number. We demonstrate in zebrafish embryos that the number of diencephalic DA cells increases following attenuation of canonical Wnt pathway without affecting the fates and number of neighboring neurons. We found that Wnt8b is a modulator of DA cell number that requires the activity of the Fezf2/Fezl transcription factor in this process. Birthdating analysis indicates that Wnt inhibition does not delay proper exit of DA progenitors from the cell cycle. Fate mapping identified the DA progenitor zone in the diencephalic anlage of the neural plate and show that this neurogenin- positive DA progenitor domain is affected by Wnt attenuation. Conditional inhibition of Wnt and of cell proliferation demonstrates thatWnt restricts the amount of DA progenitors during a window of plasticity, which occurs at primary neurogenesis. Our findings show that diencephalic DA population size is modulated inside the neural plate, much earlier than expected, and illustrate how robust patterning signals precisely regulate cell number.

*Supported by National Institute for Psychobiology, Benozyio Center, National Parkinson Foundation, Israel Science Foundation*.

# Abstract: The reliability and validity of the face-specific N170 ERP component recorded simultaneously with functional MRI

**Authors:** B. Sadeh, I. Podlipsky, A. Zhdanov, T. Hendler, G. Yovel

**Affiliations:** ^1^Department of Psychology, Tel Aviv University, Israel; ^2^Functional Brain Imaging Unit, Tel-Aviv Sourasky Medical Center, Israel

The ERP component N170 is a well-established ERP marker of face-selective neural response. Although numerous studies have investigated face-selective neural responses with ERP and functional MRI, the current study is the first to measure face-selective ERP response simultaneously withMR acquisition. Given the massive distortion MR acquisition generates in the EEG signal, our goal here was to assess the reliability and validity of the N170 during functional MRI data collection. To that end, 10 individuals participated in an ERP experiment in the scanner. Half of the scans were collected during and half without MR acquisition. Reliability and validity of the N170 were measured by correlating, across subjects, several measures that were recorded during MR acquisition following artifact removal, with the same measures that were collected without MR acquisition. These measures were amplitude, latency, face-selectivity (the difference between the amplitude to faces and nonface objects) and laterality (the difference between the amplitude to faces over the right and the left hemispheres). We found very high test-retest reliability coefficients for the N170 peak amplitude (*r* = 0.93 and *r* = 0.75, right and left hemisphere respectively), for the N170 latency (*r* = 0.91, *r* = 0.98, right and left hemisphere respectively), as well as high validity measures for the N170 laterality (*r* = 0.71) and its face selectivity (*r* = 0.76). Our results suggest that the face-selective N170 can be reliably measured in a simultaneous ERP-fMRI setup after the application of proper artifact removal tools.

# Abstract: The role ofmonoamine oxidase subtypes in striatal metabolism of dopamine produced froml-dopa in the rat

**Authors:** O. Sader-Mazbar, J. P. Finberg

**Affiliations:** Molecular Pharmacology Department, Rappaport Medical Faculty, Technion, Haifa, Israel

The MAO-B inhibitor drug, rasagiline is an effective adjunct treatment to L-DOPA in Parkinson's disease (PD), however Israel Society for Neuroscience 95 its precise action on dopamine (DA) metabolism still needs to be clarified. Since in advanced PD there is pronounced degeneration of serotonergic and noradrenergic, as well as DA neurons, we have used amodel of serotonergic (5-HT) as well as DA depletion in the rat striatum as a closer model of PD, and have investigated the striatal metabolism of L-DOPAderived DA by MAO subtypes in this model system. Striatal MAO activity and GFAP expression were investigated following DA lesion with 6-hydroxydopamine alone (single) or with additional 5-HT lesion with 5,7-dihydroxytryptamine (combined). There was a significant increase (137.5 +/− 0.06%, *P* < .01) in MAO-B activity in the combined lesion model, with no significant change in MAO-A activity. The increase in MAO-B activity following the combined lesion was associated with a significant increase in GFAP expression. Using microdialysis technique, the effect of chronic MAO-A and MAO-B inhibition in rats bearing either single or combined lesion was investigated. Rats were treated daily with either saline, clorgyline (1 mg/kg) or rasagiline (0.05 mg/kg) and on the 14th day striatal microdialysates were collected following a single systemic injection of LDOPA/ Carbidopa (25 and 6 mg/kg respectively). DA levels were 10 fold higher in the clorgyline groups in both types of lesions compared to controls. Rasagiline, on the other hand, increased DA levels moderately with more pronounced effect in the combined lesion than in the single lesion group (an increase of 3 and 2 folds, resp.). These results indicate that most of the MAO-A activity in striatum is not located in either DA or 5-HT axonal varicosities. In addition, MAO-B inhibition becomes important in the maintenance of higher L-DOPA-derivedDA levels in the striatum in advanced stages of PD.

# Abstract: The “human rat”—learning to use a new sense via sensory substitution principles

**Authors:** A. Saig, A. Arieli, E. Ahissar

**Affiliations:** Department of Neurobiology, TheWeizmann Institute of Science, Israel

Sensory-substitution is a device that attempts to substitute one sensory modality (e.g., vision) by another (e.g., touch). This field has practical implications as a mean to alleviate sensory deficits for populations, such as the blind and the deaf, who may be able to rely on an alternative sensory modality to gain missing information about the outside world. How does the brain learn to adopt and optimize a new sensory modality? Several fundamental questions involve mapping of one sensory modality onto another: Is active sensing crucial for a significant learning progress? What are the dynamics involved? Which motor strategies are optimal and how they can be achieved? Addressing these questions is valuable for understanding how the human brain represents the outside world through the senses. In the current research, an adaptation of a novel sensory modality was investigated in normal human subjects. Adult human participants were dressed with long elastic rods on their fingers, mimicking rat's whiskers. The participants were given 4 perceptual tasks: roughness estimation, poles localization in the horizontal and radial dimensions, and 3D shape recognition. Interestingly, with little or no practice humans were able to localize objects and estimate roughness as accurate as, and even better than, rats in equal tasks. The number of available whiskers did not affect horizontal or radial localization accuracy, while significantly affecting shape recognition. For a horizontal localization task, active sensing was significantly superior to passive sensing. Contact time analysis revealed that subjects rely primarily on temporal cues in horizontal localization, and ignore proprioceptive information. Further analysis in humans and rats is required in order to test whether these two species use similar sensory-motor mechanisms when faced with similar tasks, and, more globally, to understand how brains learn to adopt new sensory modalities and how to optimize such learning.

*This work was supported by the German* *BMBF–Israeli MOS Foundation.*

# Abstract: Maternal corticosterone mediates anxiety but not learning deficits induced by prenatal stress in rats

**Authors:** S. Salomon, M. Weinstock

**Affiliations:** Department of Pharmacology, School of Pharmacy, Hebrew University of Jerusalem, Israel

In previous studies we found that prenatal stress during the last week of gestation in rats impairs spatial learning only in the male offspring but induces anxiety in both genders.

**Aim**

The aim is to determine whether excess maternal corticosterone (COR) alters programming of the fetal brain thereby inducing learning deficits and anxiety.

**Methods**

Pregnant Wistar rats (*n* = 6) were adrenalectomized (ADX) on day 9 of pregnancy via the dorsal approach, 3 were shamoperated (SO) and the remaining 3 rats were left undisturbed and served as controls (C). Maternal resting levels of COR were maintained by addition to the drinking fluid (0.9% saline) of 25 *μ*/ml. On days 13–21 of gestation, 3 ADX and the 3 SO rats were stressed once daily by varied stressors. In order to replicate the elevation of plasma COR induced by stress, 3 ADX rats were injected s.c, once daily with COR (3 mg/kg). All the offspring were tested at the age of 60 days for spatial memory deficits in the Morris water maze and for anxiogenic behavior in the plus maze (PM).

**Results**

Both male and female offspring of SO rats spent less time than controls in the open arms of the PM. This increased anxiety was abolished by maternal ADX and restored by COR. Spatial learning of male offspring of SO rats showed a clear learning deficit that was also abolished by maternal ADX, but was not restored by COR.

**Conclusions**

Maternal adrenal hormones mediate both the anxiety, seen in the offspring of both sexes, and the learning deficits seen only in males. Of these, COR appears to be responsible for the anxiogenic behavior but not the learning deficits.

# Abstract: Is That Me in theMirror? The neural correlates of self recognition throughmovement

**Authors:** R. Salomon, R. Malach, D. Lamy

**Affiliations:** ^1^Department of Psychology, Tel Aviv University, Israel; ^2^Department of Neurobiology, Weismann Institue of Science, Israel

The question of how people recognize themselves and separate themselves from the environment and others has intrigued scientists for centuries. Currently the study of self recognition is in the forefront of neuroscientific research. Recent findings have linked several regions of the brain corresponding to the “default brain” or “intrinsic system” to self related processing. The current study used a fMRI paradigm in which subjects were asked to decide if a viewed movement belonged to them or another person based on minute synchronization differences. The results show differential brain activity in the self recognition condition in several ROIs of the intrinsic system. The findings shed light on the neural systems underlying bodily self recognition.

*This study was supported by the Levie-Edersheim-Gitter Institute For Functional Brain Imaging*.

# Abstract: Gene expression profile of Pac1 and Vpac1/2 receptors in developing and adult rat brain

**Authors:** J. Schneider, Y. Feder, M. Reichenstein, A. Pinhasov

**Affiliations:** Department of Molecular Biology, Ariel University Center, Ariel, Israel

Pituitary Adenylate Cyclase Activating Polypeptide (PACAP) is a Polypeptide (PACAP) is a multifunctional neuropeptide that binds to two classes of GPCR receptors. Type I is known as the PAC receptor and type II (VPAC1 and VPAC2) receptors are known as VIP receptors. In this study, we have investigated gene expression profiles of PAC1 isoforms (hop1, hop2, hip, hip-hop1 and short are results of alternative splicing in the third intracellular loop) and type II receptors in the developing and aging rat brain. Gene expression studies were performed using Stratagene MxPro Real Time PCR apparatus. Our data show that type I splice variants were highly expressed in the rat brain of newborns with marked decrease in expression during later stages of development. The mRNA levels of VPAC2 receptor were significantly lower than that of type I receptors in newborns. Yet, during maturation there was significant upregulation of VPAC2 mRNA expression. VPAC1mRNA was hardly expressed in newborns, but its levels increased with age in a pattern similar to that observed for VPAC2. During early stages of postnatal development the expression of type I receptor in the hippocampus was significantly higher in females than in males, whereas no significant difference was observed in the sexually mature animals. No sex dependent differences in expression were observed for the type II receptors. When we examined the expression patterns of type I and type II in the cortex, hippocampus, cerebellum and striatum, we found that type I receptor predominates in the hippocampus, while expression of VPAC1 was more prominent in the cortex and VPAC2 in the striatum. In summary, our experiments confirm that type I and type II receptors are differentially expressed during postnatal development in various parts of the brain. High expression of type I binding sites and its gender differences in the developing hippocampus will contribute to our understanding of the role of PAC1 receptor in normal brain development and function.

# Abstract: An fMRI study of striatal activity during reinforcement learning in Parkinson's Disease

**Authors:** T. Schonberg, N. Daw, R. Inzelberg, J. O'Doherty, D. Joel

**Affiliations:** ^1^Department of Psychology, Tel Aviv University, Tel Aviv, Israel; ^2^Center for Neural Science and Department of Psychology, New York University, NY, USA; ^3^Rappaport Faculty of Medicine, Technion, Haifa, Israel; ^4^Division of the Humanities and Social Sciences, Caltech, Calif, USA; ^5^Department of Psychology, Tel Aviv University, Tel Aviv, Israel

Dopamine serves a crucial role in classical and instrumental conditioning. Animal studies have shown that activity of midbrain dopamine neurons can be described accurately by computational models of reinforcement learning, and specifically by the temporal difference model. Projections of dopamine neurons have been suggested to provide the striatum information on the temporal difference error during reinforcement learning. Recent fMRI studies have used reinforcement learning models to analyze neuroimaging data obtained in healthy subjects during learning, and have shown that this approach allows tracking putative activity of midbrain dopamine neurons in the striatum. Parkinson Disease (PD) is known to involve depletion of midbrain dopamine neurons. As expected, this depletion has been shown to result in deficiency to acquire tasks which depend upon intact functioning of the dopamine system and the striatum in particular. No imaging study so far, however, tested the functionality of dopamine activity in the striatum of PD patients using computational reinforcement learning models. The aim of this project was therefore to study dopamine activity in the striatum of PD patients compared to healthy controls using computational reinforcement learning models and fMRI.Nine PD patients and 16 age matched healthy controls were scanned while performing a reinforcement learning task. Subjects' choice behavior and actual rewards obtained during the task were analyzed using a temporal difference learning model. The output of the model includes a prediction error (PE) term which is the delta between expected and actual outcome of a certain stimuli chosen by the subjects. This PE regressor is then correlated with fMRI brain activity. If dopamine activity is providing prediction error to the striatum, and is the source of the correlation observed in healthy subjects between the striatal fMRI signal and the PE regressor, such a correlation should not be observed in PD patients.

# Abstract: Molecular correlates of TTX-induced cell death

**Authors:** E. Schonfeld, I. Fishbein, V. Greenberger, M. Segal

**Affiliations:** Department of Neurobiology, TheWeizmann Institute of Science, Israel

Spontaneous neuronal network activity is an essential attribute of the nervous system. Such activity is also found in primary neuronal cultures, which we used to investigate the possible contribution of network activity to neuronal survival. We have previously demonstrated that when chronically silenced by tetrodotoxin (TTX), cultured cortical neurons undergo a process of progressive cell death over a period of up to two weeks. In addition we found that the remaining miniature excitatory synaptic currents (mEPSCs) have a crucial role in the induction of this neuronal death, pointing to a cellular inability to cope with otherwise nontoxic calcium inward currents. In the current study we set to investigate the molecular mechanisms responsible for this TTX induced neuronal degeneration. The relatively slow rate of neuronal degeneration and the absence of necrotic features such as cell swelling andmembrane rupture, made apoptosis amajor candidate for the observed neurodegeneration. We therefore tested TTX treated neurons for the expression of different apoptotic markers. While some classical apoptotic features such as positive TUNEL staining, mitochondria fission and the enhanced transcription of proapoptotic genes were indeed present, neither caspase 3 activation nor DNA laddering were evident. As to the upstream death signal, we found the calcium sensitive enzyme calpain to be involved, since its blockade by its endogenous blocker calpastatin significantly reduced neuronal death triggered by TTX. This result concurred with our original hypothesis regarding the involvement of calcium in the apoptotic process. In summary, TTX induced neuronal death appears to be a caspase-independent formof programmed cell death that shares at least part of the apoptotic pathway.

# Abstract: Gender differences in coping with “lean” and “obese” postnatal environments in a rat model of obesity

**Authors:** M. Schroeder, L. Shbiro, T. H. Moran, A. Weller

**Affiliations:** ^1^Faculty of Life Sciences, Israel; ^2^Gonda Brain Research Center, Bar Ilan University, Israel; ^3^Psychology Department, Bar Ilan University, Israel; ^4^Department of Psychiatry and Behavioral Sciences, Johns Hopkins University School of Medicine, Israel

OLETF rats are a model of hyperphagia-induced obesity. OLETF pups are heavier than their LETO controls at the time of birth and present hyperphagic characteristics early in life. In the present study, we used cross-fostering to assess the influence of the postnatal environment on short and long term obesity and examined differences between the sexes. Body weight was examined frombirth every fifth day and intake after weaning was assessed daily. In the second lactating week, an independent ingestion test was performed. The pups were sacrificed and blood plasma was collected for Leptin analysis at weaning or at postnatal day (PND) 90. Three different fat pads were collected and weighed. The estrous cycle of the females was monitored from PND 40 until PND75. The postnatal environment appeared to have a strong influence on short term obesity. OLETF males and females raised by LETO dams were leaner than controls, with normalized (to LETO controls) fat levels. LETO pups raised by OLETF dams were only slightly heavier than LETO controls, but with significantly higher fat percentages. Interestingly, in the independent ingestion test, the genotype of the pups instead of their phenotype determined intake: OLETF pups overate even when their body weight was normal. On the other hand, LETO pups ate as controls regardless of their body weight or fat percentages. After weaning, even though they became overweight, OLET Fmales remained leaner than controls until the end of the experiment. OLETF females managed to recover all the fat and weight. LETO rats returned to their lean genotypical profile. Furthermore, the structure of the estrous cycle was affected by the postnatal environment. In sum, there is a gender effect: Males can be successfully biased to a more “lean” adulthood, while females, though affected, manage to show an almost complete recovery.

*Supported by NIH/NIDDK R01DK057609-06A1.*

# Abstract: Repeated cocaine exposure induces long term alterations in NMDAR subunits in the nucleus accumbens (NAc)

**Authors:** J. Schumann, R. Yaka

**Affiliations:** Department of Pharmacology, School of Pharmacy, The Hebrew University of Jerusalem, Israel

Cocaine-induced modifications of glutamatergic synaptic transmission in the brain reward system play a key role in adaptations that promote addictive behaviors. In particular, the activation of ionotrophic glutamate N-Methyl- D-Aspartate receptor (NMDAR) in the VTA is critical for the initiation of cocaine sensitization. However, the role of NMDARs in the NAc, the brain region that mediates the expression of sensitization, remains to be explored. Using locomotor-sensitization as a behavioral paradigm, we found that repeated cocaine injections resulted in an increase in NR1, NR2A and NR2B subunits of the NMDAR in the NAc, 21 days after the last cocaine injection. However, no change in these subunits was found one day following the last cocaine injection. These changes were associated with an increase in the GluR1 subunit of the AMPA receptor. Interestingly, we found an increase in ERK activity which correlated with the increase inNMDAR subunits 21 days following cessation of cocaine injections. Taken together these results suggest that the NMDAR system, through activation of ERK signaling, is part of the long term neuroadaptations that results in the expression of cocaine sensitization. Understanding the mechanisms that underlie the alterations of NMDAR activity following repeated cocaine exposure, will enable us to identify targets for the design of therapeutic agents in brain areas thatmediate not only the development of cocaine addiction, but also addiction to other drugs of abuse.

*Supported by grant from the Israel Science Foundation (ISF).*

# Abstract: Population code formoving target in the archer fish retina

**Authors:** R. Segev

**Affiliations:** Department of Life Sciences, Ben Gurion University of the Negev, Israel

Archer fishes (Toxotes jaculatrix) are famed for their ability to shoot down insects resting on foliage above water using a jet of water from their mouth. To get a good jet of water, the snout sticks out of the water while the rest of the fish remains underwater. They direct the jet of water by pressing their tongue against a groove in their mouth while forcing the gills' covers inwards. After the prey is dislodged from its position, several archerfish compete for the falling prey by rapidly predicting the point where the insect will later land at the water surface. Each fish can initiate a single turn to point its body towards the position where the prey will later land. This turn occurs within 120–150 ms after the prey is dislodged. An open question is how the retina encodes visual information in order for the fish to achieve rapid reaction to prey landing. This extreme behavior of the archer fish is interesting to study and may serve as an important test case for our understanding of population neural codes of the retina. In order to predict the target landing position, the archer fish retina needs to transmit information to the brain about target position, speed and direction of flight. One possibility for such information transmission from the retina is by specialized direction and speed selective ganglion cell. Another possibility is integration of information from several ganglion cells by the fish brain to estimate the target's initial trajectory. Using a 256 multielectrode array to record from the archerfish retina we study how visual information critical for detection of target trajectory is encoded in the ganglion cell spikes.

*This work was supported by The Center for Complexity Science, Rich Foundation and Zlotowski Center.*

# Abstract: Pregabalin in the treatment of trigeminal neuralgia: a prospective open-label clinical trial

**Authors:** K. Sensen, M. Obermann, M. Yoon, M. Maschke, H. Diener, Z. Katsarava

**Affiliations:** ^1^Department of Neurology, University of Duisburg-Essen, Essen, Germany; ^2^Department of Neurology and Neurophysiology, Bruederkrankenhaus Trier, Germany

**Backround**

The International Headache Society defined trigeminal neuralgia as unilateral disorder characterized by paroxysmal sudden, intense attacks of facial or frontal pain that last for a few seconds and is limited to the distribution along one or more divisions of the trigeminal nerve. Important in the history of medical treatment and today still, gold standard' was the successful treatment with carbamazepine in 1962. Pregabalin has shown good results in the treatment of neuropathic pain associated with postherpetic neuralgia and diabetic peripheral neuropathy. The aim of this observational study was considering the possible benefits of pregabalin in the treatment of trigeminal neuralgia. Methods: We investigated 53 patients suffering from classical, chronic and symptomatic trigeminal neuralgia. They have been treated with pregabalin tittraded from 75 mg to 600 mg daily. Over a period of one year they were prospectively followed and every patient was asked to fill out a standardized individual pain diary.

**Results**

After a period of 8 weeks 24.5 percent of patients experienced complete pain relief remaining over the whole year of observation. The majority of patients (49,1 percent) showed a remarkable pain reduction and reduction of attack frequency. Under 50 percent pain reduction under treatment only with pregabalin was observed by 26.4 percent. 4.8 percent terminated the treatment. Conclusion: In this prospective clinical trial pregabalin has proven to be effective concerning the relief and reduction of pain as well as the reduction of attack frequency. The positive effect of pregabalin on patients suffering from trigeminal neuralgia is comparable to other anticonvulsant drugs reported for carbamazepine and gabapentin. Of importance are pharmacological characteristics of pregabalin. Pregabalin has shown less side effects, has the advantage of a rapid titration potential, fast pain reduction and it is easy to use by its administrations twice a day.

# Abstract: Effects of weanling stress and acute pre-mating shock on affect in progeny in rats

**Authors:** A. Shachar-Dadon, B. Even, C. Maroun, I. Galon, J. Schulkin, Leshem

**Affiliations:** ^1^Department of Psychology, University of Haifa, Israel; ^2^Department of Physiology and Biophysics and Neuroscience, Georgetown University, DC, USA

Pregestational adversity impacts offspring affective behavior (Shachar-Dadon et al., 2006). Here, as amodel of the possible effect of childhood abuse to a mother, we examined whether 3 days of varied, unpredictable, stress to a weanling female rat would impact its future progeny. Stress protocol was PN 27:10 minutes swim, room temperature. PN 28:3×0.5 hours raised platform, 1-hour intervals. PN 29: shock 6×0.8 mA/1s, 0.5min intervals. In another group, we modeled the possible effects of rape on the offspring by shocking female rats immediately before mating (6 s× 2 mA/2s, 1-minute intervals). Conception rates of weanling stressed females were very low (3/9), but shock before mating did not affect conception rates. One-hundred-and-seventy-one offspring were evaluated. Birth-weight of offspring of childhood stressed dams was 10% lower (*P* < .001). However, offspring of females that underwent weanling stress or acute shocks before mating were 6% heavier at weaning (*P* < .001). Performance in shuttle avoidance, and activity in the open field, were not altered by either preconception stress model. Childhood stress to the mother marginally decreased anxiety in elevated maze, but acute preconception shocks had little effect. In contrast, acoustic startle response was doubled in males of both experimental groups. These initial findings suggest that adversity to a female, even before sexual maturity, as in childhood abuse, may affect her fertility, and in her future progeny, birth-weight and aspects of their behavior, depending upon their gender. Our findings also suggest that trauma, proximate to conception as in rape, may alter offspring behavior.

*Supported by the BSF to ML and JS.*

# Abstract: The role of BDNF Val66Met and stress response in smoking cessation in young women

**Authors:** I. Shalev, Y. Meron, G. Laibe, E. Lerer, E. Laiba, Y. Raz, S. Israel, R. Bachner-Melman, F. Uzefovsky, C. Dina, M. Kaitz, R. P Ebstein

**Affiliations:** ^1^Brain and Behavior Science, Hebrew University, Jerusalem, Israel; ^2^Human Genetics, Hebrew University, Jerusalem, Israel; ^3^Psychology Department, Hebrew University, Jerusalem, Israel; ^4^CNRS 8090-Institute of Biology, Pasteur Institute, Lille, France; ^5^S. Herzog Memorial Hospital, Jerusalem, Israel

Attempts to prevent young people from initiating smoking have been notably unsuccessful suggesting that focusing on the underlying genetic and psychosocial factors in encouraging smoking cessation is an important public health strategy. Towards this end, we investigated the role of the val66met BDNF polymorphism in contributing to “kicking the habit” in a group of young smokers. Since psychological stress is hypothesized to be a principal component in initiating, maintaining and cessation of smoking the effect of val66met on salivary cortisol responses in a laboratory stress test was explored. Three young groups (<35 years) were genotyped for the val66met polymorphism: 275 ex-smokers (1 year abstinent), 233 current smokers and 1017 never smokers. 57 subjects (never and ex-smokers) were also examined in the Trier Social Stress Test (TSST). Significant association was observed between BDNF and the ex-smoking phenotype (UNPHASED; global *P* value = .00086). The val allele was significantly overtransmitted from a heterozygote parent to female ex-smokers and not to male ex-smokers. There was no association between BDNF and current smoking status. Changes in salivary cortisol in the TSST were modulated by val66met and sex. Tests of within-subjects effects showed a significant three way interaction (SPSS GLM; Time x BDNF x sex: *P* = .018). In male subjects val/val homozygotes showed a greater rise in salivary cortisol than val/met heterozygotes whereas in female subjects the opposite was observed. Tests of between subjects affects showed a significant BDNF x sex interaction (*P* = .040). This is the first report showing association between the BDNF val66met polymorphism and quitting smoking. The val allele is preferentially transmitted to female ex-smokers. Moreover, val/val female ex- and never smokers show blunted salivary cortisol responses to the TSST suggesting that the effect of BDNF on smoking cessation is partially mediated by modulation of HPA-axis responsivity.

# Abstract: Absentminded but accurate: delaying responses increases accuracy but decreases error awareness

**Authors:** S. Shalgi, R. G. O'Connell, L. Y. Deouell, I. H. Robertson

**Affiliations:** ^1^Department of Cognitive Science, The Hebrew University of Jerusalem, Israel; ^2^Institute of Neuroscience (TCIN) and School of Psychology, Trinity College, Dublin 2, Ireland; ^3^Department of Psychology and the Interdisciplinary Center, The Hebrew University of Jerusalem, Israel

Previous work has suggested that conscious error awareness may fluctuate with levels of attention. Here, we explore this relationship by showing that error awareness can be impaired when exogenous support to attentional systems is reduced by decreasing task demands. Twenty participants performed a manual Go/No-Go response-inhibition task optimized to examine error awareness. In one condition (Immediate), participants were asked to respond as quickly and as accurately as possible to each Go stimulus, and in the other condition (delayed) they were asked to time their responses to the offset of the stimulus, thereby decreasing task difficulty and imposing a more automated response set. As expected, speeding increased the error rate. However, contrary to the expectation (and to participants' subjective reports), that speeding would impair awareness of performance, we found the opposite to be true–errors were more likely to be unnoticed when the task was easier. We suggest that this tradeoff reflects two qualitatively different types of errors arising from the different cognitive demands of the Immediate and Delayed conditions. We propose that unaware errors reflect pure lapses of sustained attention and are therefore more susceptible to changes in task demands, while aware errors mostly reflect failures to inhibit responses, and are therefore most susceptible to increased response speed. This finding is important because it identifies the conditions under which error awareness can be maximally challenged, which is especially useful for ERP and fMRI experiments which require a significant number of aware and unaware errors for comparison between them.

# Abstract: Translocator Protein ligands attenuate themitochondrial membrane collapse normally induced by the antineoplastic agent Erucylphosphohomocholine

**Authors:** Y. Shandalov, L. Veenman, S. Leschiner, W. Kugler, M. Lakomek, M. Gavish

**Affiliations:** ^1^Faculty of Medicine, Technion-Israel Institute of Technology, 31096 Haifa, Israel; ^2^Pädiatrie I, Universitätsklinikum Göttingen, Göttingen, Germany

Malignant gliomas are the most common brain tumors, which in most instances are resistant to radiation and chemotherapy and therefore remain largely incurable. The novel chemotherapeutic drug, Erucylphosphohomocholine (ErPC3) has shown potent antineoplastic activity on various malignant tumor cell lines. ErPC3 activates the mitochondrial apoptosis pathway. The mitochondrial apoptosis pathway is triggered by opening of the mitochondrial permeability transition pore (MPTP). By genetic manipulation, we found previously that the MPTP associated Translocator Protein (TSPO) is needed for apoptosis induction by ErPC3. Presently, we study ErPC3 induced apoptosis levels in the glioma cell lines, U118MG, A172 and U87MG: (1) by using the cell death detection ELISAplus kit of Roche, and (2) by Fluorescence Assisted Cell Sorting (FACS) determining the percentage of cells in the pre-G1 phase of the cell cycle. We found, for example, that the classical TSPO ligand, PK 11195 (25 uM), reduces the levels of apoptosis triggered by ErPC3 in the U118MG glioma cells by 80%. We also show by viability experiments using Propidium Iodide that PK 11195 reduces the percentage of dead cells after ErPC3 treatment, e.g., PK 11195 (25 uM) reduces the proportion of dead U118MG cells by 75%. Using the dye JC-1, we found that PK 11195 attenuates ErPC3 induced opening of the MPTP in the U118MG, A172 and U87MG glioma cell lines. For example, PK 11195 (25 uM) causes a 57% reduction in the number of U118MG cells displaying opening of the MPTP. Thus, it appears that ErPC3 reduces viability of cancer cells via opening of the MPTP leading to cell death, including apoptosis. Furthermore, it appears that the TSPO ligand PK 11195 can prevent antineoplastic effects of ErPC3 by preventing the opening of the MPTP and cell death, including apoptosis. These data provide further evidence that the TSPO can be targeted for novel approaches to treat cancer.

*Research support: WK, LV, ML, and MG (Joint Lower Saxony- Israeli Research Projects, VWZN2047)*.

# Abstract: The effects of stimulus expectation on perception and the dynamics of cortical activity

**Authors:** R. Shani-Hershkovich, A. Arieli

**Affiliations:** The Weizmann Institute of Science, Rehovot, Israel

The notion that context influences perception and behavior is not new. Yet, there is little knowledge about the principles and the actual details of this process. In this study we have run a series of experiments on human participants investigating to what extent it affects our behavior and whether ongoing activity expresses the brain's internal context: what information it contains and what cortical regions represent it. For assessing the effect of prior information on performance we have developed a behavioral paradigm in which an orientation cue informs participants about subsequent target shape. We used a spatial 2-alternative-forced-choice contrast discrimination task, while pre-cueing target orientation (cued condition) or not (un-cued condition) for manipulating expectation on the orientation domain. During the performance of the psychophysical task, EEG activity was recorded from the entire skull (64 electrodes) simultaneously with high resolution recording of eye movements. We found a significant effect of pre-cueing target orientation on detecting target location, providing positive evidence for the debatable effects of attention to stimulus features on task performance. Preliminary analysis shows that this behavioral effect was accompanied by reduced ocular activity before and during target onset. On the brain activity level (EEG), we are currently examining the manifestation of these interactions on the correlations between the single-trial pre-stimulus (ongoing) activity and the participant's prediction of target orientation (cue versus un-cued conditions), as well as on taskrelated cortical dynamics in related areas.

*This work was supported by the German Federal Ministry of Education and Research, Israeli Ministry of Science and Technology Foundation*.

# Abstract: The Ectosylvian Sulcal Audiotory Field (FAES) and Localization of Sounds

**Authors:** A. Shapira, G. Jacobson, I. Nelken

**Affiliations:** ^1^Neurobiology Department, Life Science Institute, Hebrew University of Jerusalem, Israel; ^2^Interdisciplinary Center for Neural Computation, Hebrew University, Israel

Both the primary auditory cortex (A1) and Anterior Ectosylvian Sulcal Auditory Field (FAES) are believed to be involved in localization of sound. In both fields, neurons have been found to be sensitive to the direction of sound sources. Furthermore, FAES is known to project heavily to the deep layers of the superior colliculus. We have recently shown that the posterior part of AES has special response properties, with an over-representation of frontal space relative to A1 and the anterior part of AES. We examined the selectivity of a single unit to azimuth and elevation in the FAES (aAES and pAES) and compared it to neural responses in A1. We used virtual space stimuli consisting of short noise bursts (100 ms) filtered through head-related transfer functions covering the frontal region (from −75 to 75 degrees in azimuth, −60 to 30 degrees in elevation). In order to find the physical cues to which the neurons were sensitive, we tested the same neurons with modified stimuli in which some of the physical cues were changed (spectral notches, interaural time differences, interaural level differences). Spatial sensitivity was quantified by the mutual information (MI) between stimuli and responses. The overall selectivity to spatial location was similar in A1, posterior AES and anterior AES. Single neurons showed sensitivity to all three physical cues. However, at the population level, modifying any of the physical parameters resulted in significant reduction in the sensitivity to azimuth of neurons in pAES, but not in aAES nor in A1, and a significant reduction in the sensitivity to elevation in both pAES and aAES but not in A1. We conclude that integration mechanisms underlying spatial sensitivity in pAES are different from aAES and A1: azimuth sensitivity in pAES is not due to interaural level differences only, but is shaped in addition by the direction-dependent spectral cues.

# Abstract: Towards a characterization of natural sounds

**Authors:** O. Shayevitz, E. Nelken

**Affiliations:** ^1^Department of Neurobiology, Institute of Life Sciences, The Hebrew University of Jerusalem, Israel; ^2^Interdisciplinary Center for Neural Computation, The Hebrew University of Jerusalem, Israel

It is often assumed that the auditory system is adapted, through both evolutionary and developmental processes, to the statistics of the sounds to which it is exposed. However, actual examples of auditory adaptation to the structure of natural sounds are rare. In a groundbreaking study, Aertsen and collaborators (1979) coined the term “acoustic biotope” and studied neural responses to a small set of representative natural sounds. Yet, while natural sounds are obviously only a small subset of all possible sounds, there is no statistical characterization of this set among all sounds. A parametric representation of natural sounds could be used to guide experiments that appropriately sample the space of all sounds, thus exploring the nature of the neural responses to relevant sounds. Furthermore, better understanding of the structure of natural sounds could reveal the underlying evolutionary reasons for perceptual phenomena such as auditory masking, temporal integration, and auditory pattern recognition. For example, Nelken et al. (1999) analyzed a large set of natural sounds and suggested that comodulation masking release is an adaptation of the auditory system to natural statistics. We explored the structure of natural sounds using dimensionality reduction methods. By resynthesizing sounds from reduced representations, we evaluated the importance of the ignored dimensions. Principal component analysis of spectrograms showed that spectro-temporal structure could be substantially reduced (to about 6 components) without affecting the naturalness of the sounds. On the other hand, the phases had to be represented relatively faithfully in order to avoid serious distortions. We explored parametric representations of phases using linear regression and reduced encoding of the regression errors.

# Abstract: Maternal obesity levels at the time of weaning are influenced by the strain of the pups: insights from a cross-fostering study in the OLETF ratmodel of obesity

**Authors:** L. Shbiro, M. Schroeder, T. H. Moran, A. Weller

**Affiliations:** ^1^Psychology Department, Israel; ^2^Gonda Brain Research Center, Bar Ilan University, Israel; ^3^Faculty of Life Sciences, Bar Ilan University, Israel; ^4^Department of Psychiatry and Behavioral Sciences, Johns Hopkins University School of Medicine, Md, USA

The OLETF rat has been extensively studied as a model of hyperphagia-induced obesity. OLETF pups are heavier than their LETO controls at the time of birth and they present hyperphagic characteristics in independent ingestion and nursing tests throughout lactation. In the present study, we used the cross-fostering strategy to assess the influence of the pups' genotype on the dams' obesity levels at the time of weaning. The aim was to attain a better understanding of the interaction between the dams and the pups and how this influences their tendency towards obesity. Body weight of the dam was examined every fifth day and her intake was assessed daily. At the end of the third postnatal week, a nursing test was performed. At the time of weaning the dams were sacrificed and blood plasma and three different fat pads were collected and weighed. LETO dams appeared not to be influenced by the strain of the pups, and presented similar physiological parameters at the time of weaning, when raising pups of both strains. In contrast, OLETF females were strongly influenced by the strain of the pups: raising genetically “lean” offspring induced them to accumulate even greater amounts of fat, and to present increased body weight and Leptin levels at the time of weaning, even though their intake was the same as controls. In the nursing test, both OLETF and LETO dams nursed for similar durations when raising pups from the opposite strain. This nursing length was significantly longer than that observed in the LETO control strain, but significantly lower than the time usually observed in the OLETF strain. Taken together, the results suggest a strong influence of the offspring's characteristics on maternal behavior and the dam's physiological adaptation to lactation. Obese OLETF dams appear more susceptible than controls to this influence.

*Supported by NIH/NIDDK R01DK057609-06A1.*

# Abstract: Identification of neuroprotective pathways in brain trauma using heat acclimation preconditioning

**Authors:** N. A. Shein, M. Horowitz, E. Shohami

**Affiliations:** ^1^Department of Pharmacology, The Hebrew University, Jerusalem, Israel; ^2^Department of Physiology, The Hebrew University, Jerusalem, Israel

The development of therapeutic strategies in traumatic brain injury (TBI) is hampered by insufficient understanding of pathological and protective roles played by discrete molecular processes. Experimental induction of protective pathways is therefore a powerful research model, possibly contributing to future drug design and testing. In our ongoing work, endogenous protective pathways were induced in a mouse model of closed head injury (CHI) by using long-term heat-acclimation (HA, 30 days at 34 ± 1°C), a physiological model which is capable of facilitating protection against various stressors including TBI. The benefits of HA on motor and cognitive functions, neurodegeneration and brain edema were initially established. Subsequently, multiple pathways suggested as being pivotal in either HA or neuroprotection (NP) were studied in an effort to investigate their involvement in endogenous NP following CHI. HA-induced NP was associated with a network of concerted changes consisting of absolute and dynamic alterations of factors and processes. These included augmented pre-injury levels of hypoxia-inducible factor-1, erythropoietin receptor, brain-derived neuroptrophic factor (BDNF) and antiinflammatory cytokines, all of which are well-established as being neuroprotective. Pre-injury changes were complemented by modified post-injury dynamic responses harboring sustained post-injury hypothermia, reduced expression of pro-inflammatory cytokines and selective enhancement of BDNF-positive microglia after injury. When combined with selective pharmacological manipulation, the use of the HACHI model enabled us to determine the necessity of Akt phosphorylation for NP, bringing to light the potential therapeutic value of this pathway. Taken together, the findings substantiate the use of this paradigmfor the study of endogenous protection, providing valuable insight which can conceivably be utilized for development of treatment interventions in a physiological-mimetic manner.

# Abstract: Endocannabinoid- andmGluR5-dependent short-term synaptic depression in an isolated neuron/bouton preparation fromthe hippocampal CA1 region

**Authors:** A. Sheinin, D. M. Lovinger

**Affiliations:** ^1^National Institute on Alcoholism and Alcohol Abuse, National Institutes of Health, Md, USA; ^2^Department of Neurobiochemistry, GeorgeW. Wise School of Life Sciences, Tel-Aviv University, Israel

Endocannabinoids released from the postsynaptic neuronal membrane can activate the presynaptic CB1 receptors and inhibit neurotransmitter release. In hippocampal slices, depolarization of the CA1 pyramidal neurons elicits an endocannabinoid-mediated inhibition of GABA release known as depolarization-induced suppression of inhibition (DSI). Utilizing the highly reduced postsynaptic neuron/ synaptic bouton preparation from CA1 region of hippocampus, we have begun to examine endocannabinoiddependent short-termdepression (STD) of inhibitory synaptic transmission under well-controlled physiological and pharmacological conditions in an environment free of other cells. Application of the CB1 synthetic agonist WIN55212- 2 and endogenous cannabinoid 2-AG produced a decrease in sIPSC frequency and amplitude, indicating the presence of CB1 receptors at synapses in this preparation. Endocannabinoid-dependent STD is different from DSI found in hippocampal slices and the neuron/bouton preparation from basolateral amygdala (BLA) since depolarization alone was not sufficient to induce suppression of spontaneous inhibitory postsynaptic currents (sIPSCs). However, concurrent application of the mGluR agonist DHPG and postsynaptic depolarization resulted in a transient (30– 50 seconds) decrease in sIPSC frequency and amplitude. Application of DHPG alone had no effect on sIPSCs. The depolarization/DHPG-induced STD was blocked by the CB1 antagonist SR141716A and the mGluR5 antagonist MPEP, and was sensitive to intracellular calcium concentration. The observed STD was not related to depletion of the readilyreleasable pool of neurotransmitter, probed with high sucrose challenges. Comparing the present findings with earlier works in hippocampal slices and BLA, it appears that endocannabinoid release is less robust in isolated hippocampal neurons.

# Abstract: MRI detects in vivo migration of rat's bone marrow derivedmesechymal stemcells towards quinolinic acid lesion

**Authors:** N. Shemesh, O. Sadan, D. Offen, E. Melamed, Y. Cohen

**Affiliations:** ^1^Department of Chemistry, Faculty of Exact Sciences, Tel Aviv Univeristy, Israel; ^2^Laboratory of Neurosciences, FMRC, Rabin Medical Center, Sackler School of Medicine, Tel Aviv University, Israel

MRI is the most important imaging method in studying microstructures and functions of normal and diseased CNS. Huntington's disease (HD) is a hereditary neurodegenerative disease, manifested mainly by a cognitive decline and hyperkinetic movement disorder. The hallmarks of the condition include striatal neurodegeneration. Quinolinic acid (QA) injection to the mid-striatum unilaterally causes a slowly progressing excitotoxic lesion which resembles the neurodegeneration in HD (1). Stem cells have been proposed as a possible treatment to neurodegenerative disorders. However, a major setback post-implantation is proving the viability of the implanted cells. It is difficult to delineate these cells from normal brain tissue noninvasively. In this study, we labeled mesenchymal stem cells (MSC) post differentiation to neurotrophic factors producing cells with superparamagnetic iron oxide (SPIO) particles, and transplanted them posterior to the QA lesion. We were able to detect the migration of the SPIO labeled cells towards the lesion over a period of several weeks. The MRI images clearly show the accumulation of SPIO in the lesion and mainly on its borders. Moreover, one animal that had a cortical damage showed accumulation of the SPIO in the cortex, implying that the cells can react to chemotactic signals derived from the lesion site, and find the paths to migrate towards it. Control animals were injected with SPIO particles alone, and exhibited no accumulation and no path of migration. Post-mortem histologic evaluation demonstrated that the distribution of the cells was comparable to the MRI images. These findings prove that in this experimental model of HD, the cells are viable, and that MRI can be used as a platform to detect the paths of migration. This gives hope to future research on these cells, chiefly on their pharmaceutical capacity ([1]).

## References

[1] Schwarcz R, Whetsell WO, Mangano RM (1983). *Science*.

# Abstract: Tau-inducedmicrotubules polarity reorientation leads to axonal traffic jams and neurodegeneration

**Authors:** O. A. Shemesh, H. Erez, I. Ginzburg, M. E. Spira

**Affiliations:** ^1^Department of Neurobiology, Institute of Life Science, The Hebrew University, Jerusalem, Israel; ^2^Department of Neurobiology, Weizmann Institute of Science, Rehovot, Israel

At the forefront of tauopathies research are the attempts to understand the molecular and cellular cascades that lead to the pathologies, in particular Alzheimer's disease (AD). It is generally accepted that the cascade generated by tau overexpression culminates in impaired organelle transport leading to neurodegeneration. Several mechanisms have been proposed to link tau overexpression and impaired axoplasmic transport. Nevertheless, the underlying mechanisms are not clear. Here we developed a novel cellular platform and used confocal microscope imaging to address the above questions. For the study, we overexpressed human wild-type tau or double mutant tau (containing both mutations K257T and P301S) fused with cerulean. In order to label polymerizing microtubules we expressed the plus end microtubule tracking protein EB3-GFP. For imaging of anterograde and retrograde transport we imaged cherry-SNAP-25 and the fluid phase endocytotic marker–sulphorhodamin 101 respectively, and for imaging of mitochondria we used the mitotracker –RPAC. Overexpression of cerulean-tau generates three abnormal phenotypes characterized by (1) transient appearance of MicroTubules-Array (MTA) in the axon. The MTA locally disrupted the axonal transport and spontaneously disappeared within 48–72 hours; (2) reversal of the polar orientation of a significant fraction of the MTs. This led tomassive mitochondria and vesicular “traffic jam;” (3) the accumulation of tau and MTs at the submembranal domain, concomitant with condensation of organelles within the axon core followed by neurodegeneration. As in other model systems, the pathological effects of the mutant form of human tau were more prominent than its wild type form, indicating our model systemmay be suitable for the study of tauopathies.To conclude, we unraveled a new mechanism that underlies the aberrant transport characteristic of tau overexpressing neurons, namely, the reorientation of MTs polarity and their displacement.

# Abstract: Syntactic processing and lexical retrieval during sentence comprehension: an fMRI study

**Authors:** E. Shetreet, N. Friedmann, U. Hadar

**Affiliations:** ^1^Department of Psychology, Tel Aviv University, Israel; ^2^School of Education, Language and Brain Laboratory, Tel Aviv University, Israel

The construction of grammatical sentences relies on syntactic rules, as well as on lexical information associated with the verb in the sentence. This fMRI study explored the retrieval of verb-dependant lexical information and the processing of syntactic structures during sentence comprehension, by identifying their cortical locations and patterns of activation. Stimuli were either verbs that select prepositional-phrases (PP) or noun phrases (NP), but not sentential complement (CP; e.g., taste from… /the… ), or verbs that select PP or CP, but not NP (e.g., complain about… /that… ). For syntactic differences, we compared sentences containing syntactically complex complements (CP) with sentences containing syntactically simpler complements (NP). For lexical differences, PP-complements were used, allowing comparison of syntactically identical structures, but differential lexical information. Nineteen Hebrew speakers performed a semantic decision task on auditorily presented sentences. Comparing CP-complements with NP-complements resulted in clear activations in several areas, including left-IFG (Broca's area), bilateral STG (Wernicke's area), MTG and SMG, as well as medial precuneus. Comparing sentences including CP-verbs with PP-complements and sentences including NPverbs with PP-complements (CP-verbs > NP-verbs) revealed activations only in bilateral MTG and medial precuneus. The reverse comparisons yielded no activations. The results regarding the lexical activations can be attributed to either lexical-syntactic differences between the sentences, or to lexical-semantic differences between the verbs. However, the extensive network of areas identified in the syntactic comparison can clearly be attributed to the syntactic processing of embedding, in this case CP-complements of verbs. This indicates that complex syntactic structure load on many cortical resources.

*This study was supported by The Levie-Edersheim-Gitter Institute For Functional Brain Imaging.*

# Abstract: Viewpoint dependent representation of observed actions

**Authors:** L. Shmuelof, U. Hertz, E. Zohary

**Affiliations:** ^1^Neurobiology Department, Hebrew University, Jerusalem, Israel; ^2^Interdisciplinary Center for Neural Computation, Hebrew University, Jerusalem, Israel

The anterior intraparietal cortex (aIPS) is known to be involved in visually guided grasping. Recently, we have shown that this area is also active when we observe grasping movements. Here we investigate to what extent the performance in an imitation task and the aIPS activation is dependent on the viewpoint of the observer. Ten subjects observed video clips of right or left hands making grasping movements. Importantly, in one set of clips the hands were shown from an egocentric point of view (as if the observer is the actor), while in the other set the clips were shown from an allocentric point of view (as if the actions are made by an actor facing the subject). The results confirm our previous findings that in the egocentric point of view, the fMRI activation in the aIPS is contralateral to the observed hand (i.e., the right aIPS was significantly more active during observation of left hand clips than right hand clips. In the left aIPS, the preference was the opposite). Interestingly, for an allocentric point of view, the representation is ipsilateral. Subsequently, we looked for a behavioral correlates for this phenomenon: Subjects observed sequences of finger tapping movements made by the right or left hand from an egocentric or allocentric point of view and had to imitate them with their right hand. Imitation of the right egocentric and left allocentric clips was significantly faster than imitation of the left egocentric and right allocentricly aligned clips, respectively. To summarize: actions seen from an egocentric point of view elicit greater activation in the contralateral aIPS, and consistently, faster imitations with the observed hand. Actions seen from an allocentric view point (as performed by someone else) elicit greater ipsilateral aIPS activation and faster imitation of the opposite hand. The results suggest that simulation of observed actions is affected by the field of interaction between the action of the actor and the viewer's mental simulation.

# Abstract: Serotonin is a neuromodulator of short- and long term synaptic plasticity in the octopus vertical lobe

**Authors:** T. Shomrat, N. Feinstein, M. Klein, B. Hochner

**Affiliations:** ^1^Department of Neurobiology, Institute of Life Sciences and the ICNC, Hebrew University, Jerusalem, Israel; ^2^Department of Physiological Science, UCLA; Los Angeles, Calif, USA

Comparative analysis of brain function in invertebrates with sophisticated behaviors, such as the octopus, may advance our understanding of the evolution of the neural processes that mediate complex behaviors. In a previous study (Hochner et al., 2003) we discovered a hippocampal-like LTP in the octopus vertical lobe (VL), an area of the octopus brain involved in learning and memory. Here we studied whether the well known mechanisms of molluscan 5HT-dependent synaptic plasticity are conserved in the octopus VL system. We found that as in other mollusks, and in contrast to vertebrates, 5HT has a facilitatory effect on synaptic transmission, albeit at about one order of magnitude higher concentrations (e.g., 100 versus 10 *μ*M in Aplysia). In contrast to Aplysia sensory-motor synapses, repetitive exposures to 5HT do not lead to long-term facilitation. This suggests that in the octopus 5HT is not directly involved in mediation of long-term synaptic modifications. However, we describe a strong 5HT reinforcing effect on LTP induction whereby in the presence of 5HT high-frequency stimulation trains of a shorter duration than in control, are required for induction of a saturated level of LTP. Our study therefore supports the notion raised by recent experimental and theoretical studies suggesting that heterosynaptic modulation of homosynaptic activity-dependent long-termsynaptic plasticity is an important and universal principle in complex learning systems.

*Supported by Israel-US BSF and Smith Laboratory for Collaborative Research, the National Institute for Psychobiology, Israel.*

# Abstract: Work better in the dark: close your eyes

**Authors:** D. I. Shore

**Affiliations:** Department of Psychology, Neuroscience and Behaviour McMaster University, Canada

Visual deprivation for a short (e.g., 90 minutes), medium (e.g., 5 days) or long (e.g., blindness) duration increases the neural response to, and behavioural accuracy with, a tactile target. At the same time, simply closing the eyes can affect the cortical representation of a tactile stimulus. Using a novel active tactile search task, we explored the effect of closing the eyes in a completely dark room. Matching of mechanical nuts and bolts was both faster and more efficient when the eyes were closed compared to when they were open, despite equal amounts of visual information. Thus, the simple act of closing the eyes can modulate behavioural performance, and presumably the underlying neural processing. These transient changes in connectivity may represent the starting point for the more substantial changes involved in the visuotactile plasticity seen with longer deprivation.

*Supported by the Natural Science and Engineering Council of Canada.*

# Abstract: Hemispace- rather than hemisphere-superiority as the basis for visuospatial attention asymmetry: new insights fromfMRI

**Authors:** T. Siman-Tov, A. Mendelssohn, T. Schonberg, G. Avidan, L. Pessoa, N. Gadoth, L. Ungerleide, I. Podlipsky, T. Hendler

**Affiliations:** ^1^Functional Brain Imaging Unit, Wohl Institute for Advanced Imaging, Tel Aviv Sourasky Medical Center, Israel; ^2^Department of Behavioral Sciences, Ben Gurion University of the Negev, Beer Sheva, Israel; ^3^Laboratory of Brain and Cognition, National Institute of Mental Health, Bethesda, Md, USA; ^4^Department of Psychological and Brain Sciences, Bloomington, Ind, USA; ^5^Sackler Faculty of Medicine, Tel-Aviv University, Israel

Neurologists have long noted the asymmetry of hemispatial neglect, which is much more frequent and disabling following lesions of the right hemisphere (RH). RH dominance for spatial attention has been proposed, but its neural bases have not been elucidated. The present fMRI study reveals bi-hemispheric left-hemifield superiority in activation of the intact visuospatial attention network, thus proposing a hemispace- rather than hemisphere-superiority as the basis for attention asymmetry. Using mixed block-event related design paradigm we presented pictures of either facial expressions or houses for 150 ms to the left and the right hemifields separately. Nineteen right-handed healthy adult females participated in the studies. Multistudy statistical brain maps of the left versus right visual field contrast revealed that LVF epochs differentially activated a cortico-subcortical network, which has previously been associated with covert visuospatial attention. This LVF enhanced activation was evident bilaterally .The magnitude of the LVF superiority effect was estimated by a ROI analysis on the right and left IPS. Threeway ANOVA disclosed an overall LVF superiority, which was more prominent in the RH. Our study disclosed bilateral LVF superiority in a network that has been associated with covert visuospatial attention, thus suggesting a hemispacerather than hemisphere-based model for attention asymmetry. This model first offers a unified framework for understanding manifestations of visuospatial attention asymmetry in both the lesioned and the intact brain. In view of recent intra-operative and electrophysiological findings we propose that superior neural connectivity within the RH and/or from the RH to the LH might underlie the bi-hemispheric leftward bias. This new insight may shed light on the mechanisms of functional hemispheric lateralization in the intact brain and may help elucidating the neural bases of attention disorders.

# Abstract: Role of Sensory Feedback in determining the Firing Patterns of Vibrissa Motoneurons

**Authors:** E. Simony, E. Ahissar, D. Golomb

**Affiliations:** ^1^Department of Neurobiology, The Weizmann Institute of Sciecne, Rehovot 76100, Israel; ^2^Department of Physiology and Zlotowski Center for Neuroscience, Ben-Gurion University, Beèr-Sheva, Israel

The vibrissa sensory-motor system is organized as nested loops. In the lowest order loop in the brainstem, sensory neurons in the trigeminal nucleus project to the motoneurons in the facial nucleus. What is the role of this excitatory feedback in controlling the firing patterns of motoneurons? We explore this issue using a simplified model of the system, which includes a neuron coupled to itself with a delay. This conceptual single-neuron model stands for any loop where all the neurons in each nucleus fire in full synchrony. Preliminary results reveal that feedback effects depend on the factors that cause motoneurons to fire. One possibility is that the facial motoneurons are stimulated periodically by a central pattern generator (CPG). Assuming a sinusoidal stimulation, we find that the average firing rate depends on the stimulus frequency via an inverted U-shaped function. At low frequencies, the neuron fires one spike per cycle, whereas at high frequency the neuron fires once every few cycles or not at all. The sensory feedback can increase the number of spikes per cycle, and hence the firing rate, but not the underlying frequency, which is governed by the CPG. Another possibility is that motoneurons are pacemakers. In this case, weak or moderate feedback has almost no effect if the delay duration is on the order of 10ms, which is the experimentally-measured value of the first peak feedback response. With weak to moderate feedback, and our simplified model of the loop, only sensory neurons that fire at the second half of the whisking cycle, such as specific Whisking cells, Detach cells, or Touch cells that encounter objects during retraction, can affect the timing of the next motoneurons spike. Analysis of a network model, in which diverging feedback events induce a positive (delayed) coupling between different pacemaking motoneurons, is currently in progress.

*Supported by ISF Grant 311/04, ISF Grant 959/06, and BSF Grant 2003222.*

# Abstract: To freeze or not to freeze? Cognitive enhancement in themodulation of associative learning: a study of forebrain neuronal glycine transporter 1 (GlyT1) deletion

**Authors:** P. Singer, B. K. Yee, D. Boison, H. Mohler, J. Feldon

**Affiliations:** ^1^Laboratory of Behavioural Neurobiology, ETH Zurich, Schorenstrasse 16, CH-8603 Schwerzenbach, Switzerland; ^2^R.S. Dow Neurobiology Laboratories, Legacy Research, Portland, OR 97232, USA; ^3^Institute of Pharmacology, University of Zurich, Winterthurerstr. 190, CH-8057 Zurich, Switzerland

The NMDA receptor (NMDAR) is critically involved in the induction of long-term potentiation (LTP)–a type of experience dependent change in synaptic strength considered being the cellular basis of learning and memory. Under physiological conditions, NMDAR activity is tightly regulated by glycine–the endogenous obligatory co-agonist of NMDARs. In turn, the synaptic availability of glycine is controlled by glycine transporter 1 (GlyT1). Elevation in extracellular glycine levels can be achieved by knockout of the Glyt1 gene in forebrain neurons, which augments NMDARmediated currents, and is sufficient to influence and bias associative learning as shown here using the Pavlovian conditioned fear procedures. First, forebrain neuronal GlyT1 deletion enhanced both the magnitude and persistence of the conditioned freezing response to a tone conditioned stimulus (CS) previously having been paired with a foot shock unconditioned stimulus (US). This effect remained apparent even in aged 24-month old mice. Second, enhanced contextual fear was observed in these mutant mice when the animals were explicitly conditioned to a novel context in the absence of any discrete CS. Third, such excessive conditioned responding was not at a cost to the selectivity of associative learning. Indeed, sensitivity to the CS pre-exposure effect (i.e., latent inhibition) and protracted CS-US intervals (i.e., trace conditioning effect) was more pronounced in these animals. The responsiveness to CS preexposure or CS-US trace interval was enhanced in the mutant mice. Hence, rather than simply strengthening any potential associative links between stimuli, increased ambient glycine concentration in synapses can facilitate selective learning and enhance cognitive flexibility. We propose that factors such as CS-US contiguity/ contingency and CS/US associative history ought to be taken into consideration when evaluating potential cognitive improving interventions using Pavlovian conditioning procedures

# Abstract: Sequence structure identification and characterization of C6orf217: a positional candidate gene for schizophrenia

**Authors:** A. Slonimsky, A. Alkelai, D. Amann-Zalcenstein, T. Olender, E. Ben-Asher, D. Lancet, B. Lerer

**Affiliations:** ^1^Biological Psychiatry Laboratory, Hadassah Ein Karem, Jerusalem, Israel; ^2^Department ofMolecular Genetics, Weizmann Institute of Science, Rehovot, Israel

Genome scan and fine mapping of an inbred Arab-Israeli family sample mapped a schizophrenia susceptibility locus to human chromosome 6q23 (Lerer et al.,2003; Levi et al.,2005). Genotyping of single nucleotide polymorphisms (SNPs) was performed (Amann-Zalcenstein et al.,2006). The most significant genetic association with schizophrenia for single SNPs and haplotypes was within a 500 kb genomic region, which harbors the Abelson Helper Integration Site 1 (AHI1) gene and an adjacent putative gene, C6orf217. AHI1 is a well-established neurodevelopmentally implicated gene, involved in Joubert Syndrome with brain malformation and mental retardation (Ferland et al., 2004). In contrast, C6orf217 is a putative gene of unknown function. In silico analysis in our laboratory predicted the gene coding sequence of C6orf217 on the basis of ESTs cluster (Uni- Gene Hs.510098). The longest open reading frame of existing shows no structural similarity to any known protein. The putative coding sequence and protein show similarity to chimpanzee and rhesus monkey genomes, but is not conserved in other organisms. Relatively highly conserved regions were found inside intronic sequences of the gene. Possible responsibility of the sequence for regulatory or other functions on an RNA level was explored. The putative gene was found to be a primate specific unique RNA or protein-coding gene. Rapid amplification of cDNA ends PCR of the transcript from whole brain cDNA library confirmed the expression of mRNA sequence in brain, defined the transcript boundaries and identified the unique splice variant that is expressed in the human brain (Accession number EU130902).DNA and cDNA samples from 35 schizophrenic, 35 bipolar and 35 healthy control brains were obtained from the Stanley Array collection (www.stanley.org). Taqman gene expression analysis for both C6orf217 and AHI1 transcripts is in progress. Further analysis will explore the possible role of C6orf217 in schizophrenia pathogenesis.

# Abstract: The physiological and pathological activities of amyloid-beta peptide: From synaptic release to information processing in neuronal networks

**Authors:** I. Slutsky, E. Abramov, E. Ruff

**Affiliations:** Department of Physiology and Pharmacology, Sackler School of Medicine, Tel Aviv University, Israel

Alzheimer's Disease (AD) is the most common late-life dementia. Diverse lines of evidence suggest that amyloid-beta polypeptide (A-beta) plays a central role in pathogenesis of AD. Current AD hypothesis states that soluble A-beta assemblies form in a concentration-dependent manner and trigger down-regulation in the number, strength and plasticity of synaptic connection, resulting eventually in cognitive decline. Although A-beta hypothesis gains strong support, the endogenous processes triggering synaptic dysfunction in AD remain obscure. Recent findings suggest that A-beta release and, therefore, its concentration in extracellular space are regulated by synaptic activity through vesicle exocytosis. Therefore, decreased synaptic activity reduces A-beta level, a process that should improve memory function. However, on other hand, memory-related forms of synaptic plasticity are manifested as increase in synaptic activity. We use optical, electrophysiological, and molecular techniques in hippocampal cultures to explore the casual relationship between synaptic activity, A-beta release and plasticity of synaptic networks at physiological and pathological conditions. We address following questions. First, whether amount and pattern of neuronal activity differentially affect A-beta release? Second, what are the immediate effects of endogenously released A-beta on transmitter release associated with different patterns of neuronal activity? And third, what are the mechanisms transforming accumulation of A-beta to synapse loss, the primary factor contributing to memory decline in AD patients. Understanding the initiation phase of synaptic dysfunction by A-beta is essential for the development of new strategies to prevent memory decline in AD.

# Abstract: Development of input connections in neural cultures

**Authors:** J. Soriano-Fradera, M. Rodriguez Martinez, T. Tlusty, E. Moses

**Affiliations:** Department of Physics of Complex Systems, Weizmann Institute of Science. Rehovot 76100, Israel

We introduce a novel approach for the quantitative assessment of the connectivity in neuronal cultures, based on the statistical mechanics of percolation on a graph. This allows us to follow the development of the culture and see the emergence of connectivity in the network. The culture becomes fully connected at a time equivalent to full term. The spontaneous bursting activity that characterizes cultures develops in parallel with the connectivity. The average number of inputs per neuron can be quantitatively determined in units of m0, the number of activated inputs needed to excite the neuron. For *m*0 = 10 we find that hippocampal neurons have on average 40–80 inputs while cortical neurons have 50–100, depending on neuronal density. The ratio of excitatory to inhibitory neurons is determined using the GABAA antagonist bicuculine. This ratio changes during development and reaches the final value at day 7-8, coinciding with the expected time of the GABA switch. For hippocampal cultures the inhibitory cells comprise about 30% of the neurons in the culture while for cortical cultures they are about 20%. Such detailed global information on the connectivity of networks in neuronal cultures is at present inaccessible by any electrophysiological or other technique.

# Abstract: The involvement of the raphe nuclei in parkinson's disease

**Authors:** D. Sourani, G. Goelman

**Affiliations:** ^1^MRI\MRS Laboratory of the Human Biology Research Center, Israel; ^2^Department of Medical Biophysics and Nuclear Medicine, Israel; ^3^Hadassah Hebrew University Medical Center, Jerusalem, Israel

Parkinson's disease (PD) is a neurodegenerative disease which is primarily considered to affect movement due to loss of dopaminergic cells. However, a large percentage of PD patients suffer, in addition, from mental disorders. This suggests the involvement of the serotonergic system in the disease. Our previous studies demonstrated reduced connectivity between the basal ganglia and the raphe nuclei (the main serotonin generator) through the habenula complex in unilateral 6-OHDA injected rats. Our aim in this study was to evaluate these findings by comparing the anterograde connectivity of the raphe nuclei between sham and 6-OHDA rats. Direct intracranial injection to the raphe nuclei (AP:-0.93, ML:0.03,DV:-0.8) of paramagnetic manganese ions which are known to enter neurons, transfer anterogradely and cross synapses, was monitored for 96 hours by T1-weighted MRI. A unique model-free principal component analysis was used and comparison between groups (sham:N=7, 6-OHDA:N=8) performed. Significant signal enhancement (compared to baseline) was observed in the lateral habenula (*P* < .0004), in the thalamic and hypothalamic areas (*P* < .001), in field CA3 of the hippocampus (*P* < .001), and in left entorhinal and perirhinal cortex (*P* < .002). In 6-OHDA rats, signal enhancement was found in the lateral habenula (*P* < .003), in the thalamic and hypothalamic areas (*P* < .001) and in field CA3 of the hippocampus (*P* < .003). No significant signal enhancements in left entorhinal or perirhinal cortex were found. Comparison of signal enhancement volume between sham and 6-OHAD rats reviles a reduction of 64% (*P* < 6 ∗ 10-7) and 69% (*P* < 5 ∗ 10-8) in the left and right lateral habenula respectively and of 82% (7 ∗ 10-9) and 64% (*P* < 5 ∗ 10-6) in left and right field CA3 of the hippocampus respectively in 6-OHDA rats. These results support our previous hypothesis of reduced raphe activity in 6-OHDA rats suggesting alternations in the coupling between the dopaminergic and serotonergic systems in PD.

# Abstract: Evaluating early preventive antipsychotic drug treatment during the prodromal phase in a neurodevelopmental animal model of schizophrenia

**Authors:** E. Spoerri, U. Meyer, B. K. Yee, J. Feldon

**Affiliations:** Laboratory of Behavioural Neurobiology, ETH Zurich, Schorenstrasse 16, CH-8603 Schwerzenbach, Switzerland

Early pharmacological treatment during the prodromal state of schizophrenia might represent an early preventive strategy to suppress the onset of a full-blown psychotic episode in adulthood. Studying in humans the long-term consequences of early preventive antipsychotic drug treatment in peri-adolescence clearly constitutes an ethical issue. Therefore, the explorative investigation of early preventive strategies in animal models of schizophrenia is warranted. The present study evaluated the long-term effects of peri-adolescent administration with the typical antipsychotic drug, haloperidol, in a neurodevelopmental mouse model of schizophrenia-related disorders. This model is based on prenatal immune activation by the viral mimic, polyriboinosinic-polyribocytidilic acid (PolyI:C), which is known to precipitate a wide variety of post-pubertal behavioral, cognitive and pharmacological abnormalities implicated in schizophrenia. The prenatal PolyI:C model also captures a “prodromal phase” in the form of enhanced sensitivity to acute dopaminergic stimulation by amphetamine, which is apparent already before the onset of the fullblown schizophrenia-related phenotype in adulthood. Using this model, we revealed that chronic haloperidol exposure (5 mg/kg/day, delivered in drinking water) during periadolescence (day 35 to 70) did not confer any protective effects against prenatal infection-induced sensorimotor gating deficiency emerging in adulthood (PND 90). In fact, periadolescence haloperidol treatment in control animals led to adult sensorimotor gating deficits (comparable to the deficit observed in the treated animals). On the other hand, the prenatal infection-induced potentiation of psychostimulant sensitivity in adulthood was blocked by preventive haloperidol treatment.Our experimental findings thus highlight both beneficial as well as detrimental long-term effects of chronic exposure to the typical antipsychotic drug haloperidol during peri-adolescence.

# Abstract: Functional architecture of collinear interactions induce spatial and temporal correlation as a basis for binding

**Authors:** A. Sterkin, U. Polat

**Affiliations:** Tel Aviv University, Sackler Faculty of Medicine, Goldschleger Eye Research Institute, Tel Hashomer

Grouping of small stimuli, presented in the visual field, into global objects is an important question in contemporary neuroscience. Studies have found that principles of perceptual organization exist, suggesting that the spatial relationships between the local retinal inputs are the parameters that may determine the observer's performance in grouping global images. An alternative view for grouping is to consider the temporal activity of the neural representations, suggesting that binding induces a stimulus-dependent synchrony between the grouped elements. It was previously shown that detection of low-contrast Gabor patches (GPs) is improved when flanked by collinear GPs, whereas suppression is observed for high-contrast GPs. The facilitation resembles the principles of Gestalt theory of perceptual organization. We propose a model for contour integration in the context of noise that incorporates a temporal element into this spatial architecture. It is based on the following principles: (1) the response increases with increasing contrast, whereas the latency decreases; (2) activity-dependent interactions: facilitation for low and suppression for high activity (3) the variance increases with contrast for both responses, rates, and latency; (4) inhibition has a shorter time-constant than excitation. When a texture of randomly oriented GPs is presented, the response to every element is decreased due to fast inhibition between the neighboring elements, thus shifting the activity to the facilitatory regime of the collinear interactions. During the next stage, the slower excitation induces selective facilitation only along the contour elements. Consequently, the response to the contour is increased, resulting in decreased variance of the response rate and latency, an effect that provides better correlation between the contour elements. Thus, collinear facilitation increases the saliency of contours by decreasing the response variance of both the rate and temporal codes.

*Supported by the Israel Science Foundation and the* *National Institute of psychobiology in Israel.*

# Abstract: Synaptic and dendritic plasticity in aged mammalian hippocampus induced by a cell adhesion molecule mimetic, FGL peptide: a 3-D ultrastructural study

**Authors:** M. G. Stewart

**Affiliations:** Department of Life Sciences, The Open University, Milton Keynes, MK7 6AA, UK

We have carried out a 3-dimensional (3D) morphometric study at electron microscope (EM) level to investigate the effect of a Neural Cell Adhesion Molecule (NCAM) mimetic on synaptic and dendritic spine plasticity in the hippocampus of aged rats. NCAMs are members of the Ig superfamily expressed on the surface of neural cells involved in cellcell interactions and synaptic plasticity. NCAM signals via a direct interaction with the fibroblast growth factor receptor (FGFR). FGL (Fibroblast Growth Loop, provided by Elizabeth Bock and Vladimir Berezin, University Copenhagen) is an NCAM mimetic consisting of a 15 amino acid peptide derived from the FGF binding site of NCAM. FGL (icv) facilitates learning and spatial memory consolidation, and can reduce the B-amyloid load in rats. Aged rats (22months, ∼560 g) (Marina Lynch TCD, Dublin) were injected subcutaneously with FGL (8 mg/kg) at 2-day intervals until 19 days after the experiment start; control rats were injected with sterile water. Animals were perfused with fixative, and coronal brain sections containing the hippocampus cut at 100 um, and hippocampal volume was measured. Tissue was embedded and ultrathin sections viewed in a new JEOL 1400 EM (120 kv) with a high resolution AMT digital camera used for image capture. Analyses were made of synaptic and dendritic parameters following stereological analyses and 3D reconstruction via images from up to 150 serial sections (see http://synapses.bu.edu). FGL altered neither hippocampal volume, nor spine or synaptic density in the medial molecular layer. However, it increased the ratio of mushroom to thin spines, the number of endosomes, and the abundance of spine apparatus, whilst it decreased synaptic and spine curvature. This indicates that FGL induces large scale changes in synapses and dendritic spines in the hippocampus of aged rats complimenting data showing its marked effect on cognitive processes.

*Supported by EU FPVI Promemoria Contract no. 51201.*

# Abstract: Context-dependent Stimulus-Specific Adaptation in Rat Auditory Cortex

**Authors:** N. Ta‘aseh, A. Yaron, I. Nelken

**Affiliations:** Department of Neurobiology and the Interdisciplinary Center for Neural Computation, The Hebrew University, Israel

Stimulus-specific adaptation (SSA) in auditory cortex was investigated in the halothane-anesthetized rat. We used oddball sequences, in which a rare (deviant) tone appeared over a background of common (standard) tones. We recorded local field potentials and multiunit activity. The response to a tone, when deviant, was substantially stronger than the responses to the same tone when used as standard. The frequency difference between deviants and standards could be as small as 20%, substantially smaller than the typical width of cortical tuning curves. At a frequency difference of 10%, the responses to standard and deviants weremostly the same. The stronger response to the deviant may be due to a reduction in the responses to the standard, but may also be due to change potentials evoked by the violation of the expectations created by the presentations of the standard tone. To test for change potentials, we embedded the deviant tones in a sequence in which many different tones were played randomly, so that each tone was as frequent as the deviant in the oddball sequence. In such a sequence, the appearance of the deviant tone is not associated with violation of expectations and change potentials are not expected. We show that the response to a deviant over a background of many standards is similar to the response to the deviant over a background of a single standard. Nevertheless, a simple model based on fatigue cannot account for the qualitative pattern the results, and it seems that the fact that a stimulus is novel in the sensory scene plays a significant role in cortical SSA. In an attempt to generate change potentials, we assigned a behavioral importance to a specific frequency by using it as a conditioned stimulus (CS) in a fear conditioning procedure. Preliminary results suggest an enhancement in the responses to CS and the presence of change potentials when the CS is used as a deviant, but not when other tones are used as deviant, in oddball sequences.

# Abstract: When noise can mask visual targets

**Authors:** S. Tadmor, U. Polat

**Affiliations:** Sackler Faculty of Medicine, Tel Aviv University, Goldschleger Eye Research Institute, Tel Hashomer, Israel

The masking (suppression) effect can be achieved either when noise is used, or when the target and mask share the same physical properties. Thus, the properties of efficient masking are not well defined. We probed the efficacy of the suppression effect on target threshold by manipulating the ISI (0–120 milliseconds), contrast (0–80%), spatial frequency (SF, 0-factor of two), and orientation (0–90 degrees). The relationships between facilitation using collinear flankers and suppression were also tested under these conditions. We found that suppression increased with increasing Israel Society for Neuroscience 109 contrast (being effective above 4.25 times the target threshold); it was maximal for simultaneous presentation (ISI = 0, mask contrast = 80%) and equal target-mask SF but decreased with increasing ISI. Suppression is maximal for near iso-orientations and decreased for increasing orientation differences. Collinear flankers did not produce facilitation for ISI = 0 but it produced more suppression for ISI = 30. Thus, efficient masking is not noise per se, rather, it is determined by combining parameters such as the similarity between the target and mask in their temporal presentation as well as their physical properties (similar SF and orientations) and the balance between excitation and inhibition. The results suggest that iso-orientation inhibition is stronger than crossorientation inhibition.

*Supported by a grant from the Israel Science Foundation.*

# Abstract: State dependantmodification in the waveformof the complex spikes

**Authors:** Z. Tal, Y. Yarom

**Affiliations:** Department of Neurobiology and The ICNC, The Hebrew University of Jerusalem, Israel

Climbing fiber activation evokes a large all-or-none response in Purkinje cells (PCs), known as the complex spike (CS). The CS, triggered by massive EPSPs to the dendritic arbor, consists of dendritic calcium currents and somatic sodium spikes. It is thus expected that the properties of these events will depend on the PCs membrane potential. Furthermore, we have recently showed that the membrane potential of PC attains two stable state values: a hyperpolarized quiescent state (down state) and a depolarized active state (up state). Therefore, we examined the CS waveform that was elicited during the up state and compared it to the waveform obtained during the down state. To this end, we performed invitro whole cell and loose-patch recordings fromPCs. We use concentric metal electrode to stimulate the climbing fibers and to evoke CSs. Intracellular current injection was used to shift the cells between the two stable states. We found that during the down state the CSs, which waveform tends to be more stable, were characterized by 4–6 fast fluctuations that followed the initial spike. In the upstate the CSs, which waveform tends to have a longer duration, were characterized by significantly higher variability of fewer and slower fluctuations. These differences may account for a lower threshold for calcium activation during up state. Since calcium concentration has been shown to play a pivotal role in synaptic plasticity, it is tempting to speculate that plastic processes in the cerebellar cortex, which are triggered by climbing fibers, are more effective during the up state.

# Abstract: Dissociable effects of real and illusory size on perception and visuomotor control

**Authors:** M. Tanzer, M. A. Goodale, T. Ganel

**Affiliations:** ^1^Department of Behavioral Science, Ben-Gurion University of the Negev, Israel; ^2^Department of Psychology, University ofWestern Ontario, Canada

A large body of evidence from behavioral, neuropsychological, and neuroimaging studies, supports the idea that visionfor- action and vision-for-perception aremediated by distinct neuroanatomical and functional systems. Yet, an ongoing debate exists as to whether visual illusions, that by definition affect the way people perceive objects, have similar effects on visuomotor control. In this study we have attempted to differentiate–within the same experimental design–the effects of the real size and the apparent size of objects on action and perception. To this end, two real rectangular objects of different sizes were presented within a variant of the Ponzo size-contrast illusion. Most experimental trials were designed such that the object that was perceived as the larger one was actually physically smaller than the other object. This design allowed us to focus on trials in which subjects have overtly decided that one of the objects is the smaller (or larger) of the pair, and to test whether or not the opening between the fingers of the grasping hand goes in the direction of this erroneous decision. The results showed that although subjects were affected by the illusion when making size decisions, the opening of their fingers in flight reflected the real size differences between the two objects. This pattern of results was reversed in a second experiment, in which subjects were asked to make perceptual estimations of size. In this case, the estimates reflected the illusory size of the objects. Thus, the real and apparent differences in the size of the objects had opposite effects on action and perception. These findings provide evidence for a double dissociation between visuomotor control and visual perception in the context of visual illusions and support the idea that visuomotor control and visual perception are mediated by distinct functional systems.

# Abstract: Vascular endothelial growth factor increases neurogenesis following traumatic brain injury

**Authors:** O. Thau-Zuchman, E. Shohami, H. Ovadia, R. R. Leker

**Affiliations:** ^1^Department of Neurology, Hebrew University-Hadassah Medical Center, Jerusalem, Israel; ^2^Department of Pharmacology, School of Pharmacology, The Hebrew University, Jerusalem, Israel

**Background**

Activation of endogenous stem cells has been proposed as a novel form of therapy in a variety of neurological disorders, including traumatic brain injury (TBI). Vascular endothelial growth factor (VEGF) is expressed in the brain following TBI and serves as a potent activator of angiogenesis and neurogenesis. Methods: TBI was induced with closed head injury in sabra mice. Mice were injected with BrdU to label newborn cells. We administered exogenous VEGF into the lateral ventricles using miniosmotic pumps to evaluate the effects on recovery and functional outcome as assessed with the neurological severity scale score. Immunohistochemistry was used to study the fate of newborn cells at 90 days post TBI.

**Results**

Our results show that VEGF significantly increases the number of proliferating cells in the subventricular zone and in the peri-lesion cortex. This increase is associated with a significantly better functional outcome following TBI as measured with the neurological severity scale. Fate analysis showed that most newborn cells differentiated into astrocytes and oligodendroglia and not into neurons.

**Conclusions**

VEGF significantly augments angiogenesis and neurogenesis after TBI butmost newborn cells adopt a glial fate. Nevertheless, there is a significant improvement in recovery rates and functional outcome as a result of these changes.

*This work was supported in part by a grant* *form the Israeli Defense Forces.*

# Abstract: Learning to move an arm inside a maze

**Authors:** K. E. Thonhauser, L. Zullo, R. A. Byrne, B. Hochner, M. J. Kuba

**Affiliations:** ^1^Institute of Zoology, Karl-Franzens-University, Graz, Austria; ^2^Department of Neurobiology & ICNC, Hebrew University, Israel; ^3^Department of Neuroscience and Brain Technologies, The Italian Institute of Technology, Italy

The arms of an octopus, the therewith-conducted movements and the coordination of these movements play an essential role in the natural behaviour of this animal. Locomotion, foraging, probing and mating are dependent on proficient arm use. In particular searching and probing movements are hypothesized to be largely autonomous (i.e., controlled by the peripheral motor system). For the octopus survival, nonvisual arm coordination and also the ability to remember a beneficial food patch are essential for its daily survival. These two issues became the basis for our experimental design. Our present study seeks to investigate if an octopus can learn to navigate a two ways choice maze based only on proprioceptive information. We used a plexiglass plate with a Y shaped maze glued to it. The octopus had to insert only one of its arms into the maze and reach a goal box located on both ends of the Y maze. Six adult Octopus vulgaris were introduced to the maze baited on both sides to determine the individual side preferences of the animals. Once this was tested the goal side was either ascribed at random, or contrary to the animals side preference. To control that chemical cues did not influence the animals' decision an equal amount of food was put into both goal boxes, but only available in the correct goal box. A criterion for successful learning was the completion of 8 out of 10 trials. Four out of six animals complete this task in less than 90 trials. Using a simple two ways choice discrimination-learning paradigm, our study shed new light on the ability of the octopus to control its motor output based on proprioceptive information only.

*Supported by Israel Science Foundation. M. Kuba was* *supported by a Golda Meir fellowship.*

# Abstract: The role ofmembers of the BMP 60A subgroup in the development of catecholaminergic neurons and the anterior pituirary

**Authors:** H. Tilleman, K. Liser, O. Scheffner, L. Nashelsky, I. Meir, A. Lanir, C. Brodski

**Affiliations:** Department ofMorphology, Faculty of Health Sciences, Ben-Gurion University of the Nege, Israel

Studies performed in zebrafish and chicken indicate that Bone Morphogenetic Proteins (BMPs) play an important role in the development of catecholaminergic (dopaminergic and noradrenergic) neurons in the CNS. However, the Bmp or combination of Bmps controlling the development of these cell populations in mammals is still elusive. In addition Bmp2 and Bmp4 have been implicated in pituitary development. However, the role of other Bmps in this process is still elusive. Using in situ hybridization, we compared the spatio-temporal expression pattern of different BMPs to developing catecholaminergic neurons in mice. We found that members of the 60A subgroup, Bmp-5, -6 and -7, are expressed in close vicinity to the neuronal population of our interest. Next, we could show that dopaminergic, and noradrenergic markers are normally expressed at postnatal day 0, in Bmp-5, -6, and -7 knockout mice. This indicates that BMP5, BMP6, and BMP7 as individual genes are not essential for the presence of dopaminergic and noradrenergic neurons in vivo. These results are very surprising, since previous in vitro data and results obtained in chicken and zebrafish, suggested the dependency of noradrenergic neurons on those BMPs. Based on our results we hypothesize that BMPs are functionally compensating for each other. In order to test our hypothesis, we will characterize monoaminergic populations in BMP compound mutants. In addition, Current experiments aim to characterize the role of BMPs in pituitary development. For this purpose we are analyzing the mRNA expression of different hormones produced by the anterior pituitary by in situ hybridization and RT-PCR.

*The work was supported by the ISF,* *The National Institute of Psychobiology,* *and the Ministry of Health.*

# Abstract: Effects ofmid-hindbrain organizer genes and members of the Bmp 60A subgroup in the development of noradrenergic locus coeruleus neurons

**Authors:** H. Tilleman, V. Hakim, K. Liser, O. Scheffner, L. Nashelsky, I. Meir, A. Lanir, C. Brodski

**Affiliations:** Department of Morphology, Faculty of Health Sciences, Ben-Gurion University of the Negev, Israel

The embryonic mid-hindbrain organizer (MHO) is composed of a transient cell population located at the developing midbrain-hindbrain junction, that controls the formation of the brain stem. Mouse mutants with a caudally Israel Society for Neuroscience 111 shifted MHO show an increase in number of dopaminergic neurons and a decrease in number of serotonergic neurons. Interestingly, cranial nerves, originating close to the MHO are not affected in these mutants, indicating that the MHO has a specific effect on cell populations forming adjacent to it. Behavioral studies revealed that these mutants show increased activity and altered locomotor response to psychostimulants. The major noradrenergic nucleus in the brain, the locus coeruleus originates at the intersection of Fgf8 that mediates MHO activity and dorsalizing Bmp signals. Deleting Fgf8, leads to a loss of the whole mid- and hindbrain region including the locus coeruleus. However, the specific role ofMHO position and activity for the generation of the locus coeruleus has so far been unknown. Inactivating bmp7 in zebrafish or blocking Bmp-5 and -7 signaling in chicken have indicated that these morphogens are necessary for the formation of the LC. However, if Bmp function is conserved in mammals and if Bmp-5 and -7 have redundant functions is elusive. Here we provide evidence that mouse mutants with a caudally enlarged Otx2 expression domain and concomitantly caudally shifted MHO (En1+/Otx2) have less noradrenergic neurons. Unexpectedly En1+/Otx2 mutants showed an enlarged Fgf8 expression domain and no changes in Bmp-5, -6, or -7 expression. A further analysis of Bmp-5, -6 or -7 knockout mice indicate that the LC develops normally in these mutants. Taken together, our data indicates that the MHO controls the development of the LC through an Otx2/Gbx2 interaction and that Bmp-5, -6 or -7 are not required, as individual genes, for the formation of the mammalian LC in vivo.

*This work was supported by ISF,* *National Institute of Psychobiology* *in Israel, and Ministry of Health.*

# Abstract: Keeping an eye on induced Gamma activity: a study of combined EEG and eye-tracking

**Authors:** O. Tomer, S. Yuval-Greenberg, L. Y. Deouell

**Affiliations:** ^1^Hadassah Medical School, The Institute of Medical Sciences; ^2^Department of Psychology, Israel; ^3^Interdisciplinary Center of Neural Computation, The Hebrew University of Jerusalem, Israel

In the last decade a large amount of electrophysiological research has focused on high frequency brain activity in humans, and specifically on the “induced gamma response” (iGBR). The scalp recorded iGBRs is manifested typically as an increase in the gamma band (>30 Hz) peaking around 250 milliseconds post stimulus, following the presentation of a visual stimulus. The iGBR was hypothesized to represent the neural synchronization involved in various cognitive functions such as binding and consciousness. Our findings show a strong correlation between the induced gamma activity and eye movements (recorded by an eye tracker). However, the direction of causality is still unclear. In the current study we present a possible model explaining the appearance of iGBR in the EEG data and its relation to eye movements. In filtered (30–100 Hz) single trials the iGBR is seen as a sharp peak which appears strongly (but in opposite polarities) in central channels and in the electroocular channels (EOG). In all four EOG channels (vertical-superior, verticalinferior, horizontal-left, horizontal-right) the peak appears at the same polarity. The pattern in the EOG channels described above cannot represent a simple saccade. Since a vertical (or horizontal) saccade will typically be demonstrated by opposite polarization of the vertical superior and inferior channels (or the horizontal left and right channels respectively). We discuss the possible involvement of a convergence or divergence movement in the manifestation of the iGBR.

# Abstract: Blood-brain barrier disruption in post-traumatic epilepsy

**Authors:** O. Tomkins, I. Shelef, I. Kaizerman, D. Zumsteg, A. Friedman

**Affiliations:** ^1^Departments of Physiology and Neurosurgery, Soroka Medical Center, Ben-Gurion University, Israel; ^2^Department of Neuroradiology, Soroka Medical Center, Beer-Sheva, Israel; ^3^Department of Neurology, University Hospital Zurich, Zurich, Switzerland; ^4^Institute for Neurophysiology, Charité University Medicine, Berlin, Germany

**Background**

Traumatic brain injury (TBI) is an important cause of focal epilepsy. Animal experiments indicate that disruption of the blood-brain barrier (BBB) plays a critical role in the pathogenesis of post-traumatic epilepsy (PTE). Objective: To investigate the frequency, extent and functional correlates of increased BBB permeability in PTE patients.

**Methods**

Thirty two patients were included in the study, with 17 suffering from PTE. All underwent brain magnetic resonance imaging (bMRI) and were evaluated for BBB disruption, using novel quantitative techniques. Cortical dysfunction was measured using quantitative electroencephalography (qEEG), and localized using standardized low resolution brain electromagnetic tomography (sLORETA).

**Results**

TBI patients displayed significant pathological qEEG slowing. No significant differences were found between spectral qEEG analyses from PTE and nonepileptic patients. While bMRI revealed that PTE patients were more likely to present with intracortical lesions (*P* = .02), no relation to the size of the cortical lesion (*P* = .19) was found. Increased BBB permeability was found in 76.9% of PTE patients compared to 33.3% of nonepileptic patients (*P* = .047), and could be observed several years following the trauma. Cerebral cortex volume with BBB disruption was larger in PTE patients (*P* = .001). In 70% of patients, slow (delta band) activity was co-localized, by sLORETA, with regions showing BBB disruption.

**Conclusions**

Lasting BBB pathology is common inmild TBI patients, with increased frequency and extent in PTE patients. A correlation between disrupted BBB and abnormal neuronal activity is suggested.

# Abstract: Computer-assisted diagnosis of blood-brain barrier disruption

**Authors:** O. Tomkins, I. Shelef, I. Kaizerman, A. Friedman

**Affiliations:** ^1^Departments of Physiology and Neurosurgery, Soroka Medical Center, Ben-Gurion University, Israel; ^2^Department of Neuroradiology, Soroka Medical Center, Beer-Sheva, Israel; ^3^Institute for Neurophysiology, Charité University Medicine, Berlin, Germany

Blood brain barrier (BBB) disruption occurs under many diverse pathological conditions, and recent studies suggest it may be involved in the pathogenesis of neurological disorders. Visually based evaluation of contrast-enhanced brain imaging is often used to detect BBB disruption. However, no established method exists for the quantitative evaluation of BBB permeability in human patients. We have recently established two methods for evaluating BBB permeability in humans, utilizing brain MRI scans. The first method compares T1- weighted magnetic resonance brain scans before and following the administration of Gadolinum-DTPA. Examining groups of 16 voxels at a time and searching for statistically significant changes in signal enhancement results in a semiquantitative estimation of the spatial location and extent of BBB disruption. In the second, dynamic method, repetitive T1 scans are performed during contrast agent administration. Following blood vessels elimination the dynamics of contrast agent concentration in each pixel are calculated. A quantitative map of BBB function is created according to the activity of the BBB throughout the parenchyma. Using both methods we receive a highly sensitive estimate of BBB patency throughout the brain parenchyma. Our study shows that BBB disruption is common in diverse neurological and systemic conditions and possibly lasts for long periods. Furthermore, the extent of disruption, its progression with time, and functional correlations can be identified.

# Abstract: Dynamic changes in the recovery after traumatic brain injury in mice: effect of injury severity on T2-weighted MRI and functional recovery

**Authors:** J. Tsenter, L. Beni-Adani, Y. Assaf, A. G. Alexandrovitch, V. Trembovler, E. Shohami

**Affiliations:** ^1^Department of Pharmacology, Hebrew University and Hadassah Medical Center, Jerusalem, Israel; ^2^Rehabilitation, Hebrew University and Hadassah Medical Center, Jerusalem, Israel; ^3^Pediatric Neurosurgery Dana Children's Hospital, Sourasky Medical Center, Israel; ^4^Neurobiochemistry, Tel-Aviv University, Israel

Memory and neurobehavioral dysfunctions typically occur after human and experimental traumatic brain injury. A Neurological Severity Score (NSS, range 0–10) was developed for evaluation of trauma severity in mice. The object recognition task (ORT) measures specific episodic memory in rodents, and is expressed by the percent time spent at a novel object (discrimination index, DI). The present study describes the effect of injury severity on the spontaneous recovery of neurobehavioral and cognitive outcome after severe and mild focal trauma in mice. Mice were subjected to closed head injury (NSS at 1h post injury of 7.52 ± 0.34 and 4.62 ± 0.14, resp.) andNSS was further evaluated during 25d. NSS decreased by 3.86 ± 0.26 and 2.54 ± 0.35 units in the severe andmild injured mice, respectively. ORT was performed between days 3 and 28 after trauma. Whereas DI in noninjured mice is ∼75%, se51.7 ± 6.15% on 3rd day and 66.2 ± 6.81% verely injured animals show DI of 51.7 ± 6.15 % and 66.2 ± 6.81% at days 3 and 21 after trauma, indicating inability to distinguish between familiar and novel objects. In contrast, mildly injured mice do not show cognitive impairment throughout the same period.NSS1h strongly correlated with the damage seen on MRI 24 h post injury (*R* = 0.8, *P* < .001). We suggest that NSS is a reliable tool for invivo continuous evaluation of neurological damage in headinjured mice, and its value at 1 h predicts the extent of motor dysfunction, cognitive damage and brain water characteristics as depicted by T2-weighted MRI. Combined assessment of neurobehavioral and cognitive functions along with MRI is most useful in evaluating recovery from injury, and was successfully applied in testing of novel pharmacological treatments.

# Abstract: Maintaining the presynaptic site—questions arising fromlive imaging experiments

**Authors:** S. Tsuriel, M. Stern, R. Kahana, N. Ziv

**Affiliations:** Rappaport Institute and the Department of Physiology, Technion Faculty of Medicine, Haifa 31096, Israel

Presynaptic sites of CNS synapses appear as small axonal varicosities often formed en passant along axons. These specializations are not closed compartments, but are continuous, to a large degree, with the axonal cytoplasm and membranes. Yet, in spite of this lack of obvious physical barriers, presynaptic structures manage to maintain their unique organization. If presynaptic specializations were static structures, this would not be that remarkable. However, presynaptic sites are loci of extensive membrane dynamics, that involve exocytosis, endocytosis and synaptic vesicle trafficking, all of which are accelerated by synaptic activity. This being the state of affairs, the persistent, focal organization of presynaptic sites is not an obvious outcome. As a step toward a better understanding the processes involved in maintaining presynaptic structure we set out to examine some of the molecular dynamics exhibited by several presynaptic molecules of various classes, including cytomatrix, membrane and active-zonal proteins. Fluorescence recovery after photobleaching (FRAP) and photoactivation experiments revealed that presynaptic molecules are continuously incorporated into and lost from individual synaptic structures over various times scales, from minutes to many hours. Moreover, these dynamics can be accelerated is some cases by synaptic activity. Finally, we find that synaptic molecules can be continuously exchanged between nearby synaptic structures at significant rates that greatly exceed the rates at which synapses are replenished with molecules arriving from somatic sources. Our findings thus support the possibility that key presynaptic molecules are continuously lost from, incorporated into and exchanged among nearby presynaptic sites at significant rates and thus further highlight questions as to how presynaptic sites manage to maintain their structural and functional characteristics over long durations.

*Supported by US-Israel Binational Science Foundation (BSF), German Israel Foundation for scientific R and D (GIF).*

# Abstract: Hippocampal neural activity in freelymoving echolocating bats

**Authors:** N. Ulanovsky, C. F. Moss

**Affiliations:** ^1^Department of Neurobiology, Weizmann Institute of Science, Rehovot 76100, Israel; ^2^Department of Psychology, University of Maryland, College Park, MD 20742, USA

The hippocampus is crucial for episodic and spatial memory. In freely-moving rodents, hippocampal pyramidal neurons exhibit spatially-selective firing when the animal passes through a neuron's “place field,” and theta-band oscillation is continuously present during locomotion. Here we report on the first hippocampal recordings from echolocating bats, mammals phylogenetically distant from rodents. Big brown bats (Eptesicus fuscus) were trained to crawl and forage for mealworms in an open-field arena, while we recorded local field potentials and single unit activity from hippocampal area CA1 using a tetrode microdrive, and tracked the position of light-emitting diodes on the animal's head. These recordings revealed place-cells very similar to rodents, as well as rodent-like high frequency ‘ripple’ oscillations. Theta oscillation, however, differed from rodents in two important ways: (i) theta occurred when bats explored the environment without locomoting, using distal sensing via echolocation; (ii) theta was not continuous but occurred in short intermittent bouts. The intermittence of theta suggests that models of hippocampal function relying explicitly on continuous theta may not apply to bats. These data support the hypothesis that theta in mammals is involved in sequence-learning, and hence theta power increases with sensory-input rates– explaining why theta-power correlates with running-speed in rodents and with echolocation-call-rate in bats. Finally, we demonstrate rapid modulation of hippocampal spatial representation in the bat, with a ∼1-second timescale, which is 1-2 orders of magnitude faster than previously reported. Future plans include recording of neural activity from the hippocampus of freely-flying bats, using a telemetry system–as well as the analysis of the relation between the animal's behavior and the network activity of dozens of simultaneouslyrecorded single units from bat hippocampus.

# Abstract: Non stationary fluctuation analysis of calcium in secretory vesicles

**Authors:** M. Valitsky, P. Blank, A. Raveh, J. Coorssen, J. Zimmerberg, R. Rahamimoff

**Affiliations:** ^1^Department of Physiology, The Hebrew University-Hadassah Medical School, Jerusalem, Israel; ^2^Laboratory of Cellular and Molecular Biophysics, NICHD/NIH, Bethesda, Md, USA; ^3^Weizmann Institute of Science, Rehovot, Israel; ^4^Department of Physiology and Biophysics, Faculty of Medicine, University of Calgary, Canada

Calcium ions have several important functions in synaptic in synaptic communication. They are directly involved in the triggering of secretory vesicle content release by exocytosis. Synaptic vesicles however, are too small for confocal optical analysis. Sea urchin eggs have larger vesicles (∼1 *μ*M) and are suitable and convenient for studying calcium dependent exocytosis. Previous work has shown that sea urchin vesicles contain a high calcium concentration. Fluorescent confocal microscopy demonstrated well defined calcium dynamics. Here we analyzed the calcium fluctuations. The presence of bleaching required the use of nonstationary stochastic analysis. This analysis showed that the variance of the calcium signal is larger than the mean; the calcium signal can not be described as a Poisson process. We speculate that this extravariance analysis can contribute to the understanding of the processes that control intracellular calcium. We found that, as expected, the highest levels of calcium induced fluorescence were in the center of the secretory vesicle. The Fano factor (variance\mean fluorescence) is the lowest in the center and increased monotonically towards the edge of the vesicle. The highest extravariance is at the edges of the secretory vesicle. Due do this extra variance, we speculate that there is an interaction between the calcium in the vesicle and the calcium in the cytosol. Moreover, we speculate that the secretory vesicle takes part in the regulation of cytosolic calcium concentration and may dramatically alter the local calcium concentration near the surface of the vesicle.

*Supported by The Israel Science Foundation.*

# Abstract: Multisensory integration of speech and script in fluent and dyslexic readers

**Authors:** N. M. Van Atteveldt, V. Blau, E. Formisano, A. Roebroeck, L. Blomert, R. Goebel

**Affiliations:** ^1^Department of Cognitive Neuroscience, Faculty of Psychology, Maastricht University, The Netherlands; ^2^Maastricht Brain Imaging Center (M-BIC), Maastricht, The Netherlands

In alphabetic scripts, letters and speech sounds are the basic elements of correspondence between spoken and written language. Therefore, investigations of the mechanism by which letters and speech sounds are associated in the brain increase our understanding of the neural basis of literacy. Using functional magnetic resonance imaging (fMRI), we showed in fluent readers that the response to speech sounds in the auditory association cortex is enhanced by congruent letters (e.g., letter “a” with phoneme /a/) and suppressed by incongruent letters (e.g., “o” with /a/). Interestingly, temporal proximity between auditory and visual input was critical for this congruency effect to occur. We interpreted these results as a neural correlate of letter-sound integration, driven by the congruency of letter-sound pairs learned during reading acquisition. Furthermore, we suggested that the modulation of auditory cortex was the result of feedback from the superior temporal sulcus (STS). In a subsequent study, we used event-related fMRI to investigate the effect of task instructions on neural letter-sound integration, and investigated the interactions between STS and auditory cortex directly through the analysis of effective connectivity using Granger Causality Mapping. Our findings indicated that top-down task demands can overrule the stimulus-driven (bottom-up) integration mechanism. Furthermore, effective connectivity analysis provided support for the feedback modulation of auditory cortex by STS based on the letter-sound congruency. These findings provide a working model for letter-sound integration in the “literate brain,” which serves as a basis for investigating deviant literacy development. Recent findings from our lab indicate anomalous processing of letter-sound pairs in auditory cortex and STS in dyslexic readers.

# Abstract: Optimal activity level of presynaptic GABAB receptors maximizes the capacity of synapses for correlation-based plasticity

**Authors:** I. Vertkin, B. Bettler, I. Slutsky

**Affiliations:** ^1^Department of Physiology and Pharmacology, Sackler School of Medicine, Tel Aviv University, Israel; ^2^Pharmacenter, University of Basel, Switzerland

Presynaptic GABA(B1a)-Rs play an important role in hippocampus-dependent synaptic plasticity, learning and memory. However, the mechanisms transforming activation of GABA(B1a)-Rs to plasticity and memory function remain elusive. To address this question, we use functional FM-based imaging to quantify probability of transmitter release and distribution of functional presynaptic terminals at the level of single synapses in hippocampal cultures. Our results show that mice lacking presynaptic GABA(B1a)-Rs exhibit reduced short-term and long-term presynaptic plasticity. Given that presynaptic GABA(B)Rs act as a high-pass filter, preferentially inhibiting transmission of single spikes, we propose that these receptors might regulate synaptic plasticity through fine-tuning the level of ongoing background synaptic activity. To test this hypothesis, we first quantified the effect of baclofen, an agonist of GABA(B)Rs, on transmitter release for single spikes (mimicking background activity) versus burst inputs in WT cultures. Our results show the degree of presynaptic facilitation for bursts is maximized at the optimal level of GABA(B)-Rs activation. At low baclofen concentrations (∼0.1 uM) hippocampal synapses act as band-pass filters with low degree of short-termfacilitation; moderate concentrations of baclofen (∼1 uM) significantly increase facilitation index, whereas higher baclofen concentrations (>20 uM) induce pattern-independent inhibition, and consequently reduces presynaptic facilitation. Interestingly, chronic treatment of hippocampal neurons with 1uM baclofen resulted in enhanced long-term presynaptic plasticity. However, 50uM does not affect the capacity of synaptic network for modifications. These findings indicate that the capacity of neuronal network to undergo persistent modifications is regulated by the level of ongoing background activity through presynaptic GABA(B)-Rs.

# Abstract: Vergence in reverspective: effects of real and virtual depth cues

**Authors:** M. Wagner, W. H. Ehrenstein, T. V. Papathomas

**Affiliations:** ^1^Ariel University Center of Samaria and Smith Psychobiology Laboratory, Hebrew University of Jerusalem, Israel; ^2^Leibniz Research Center forWorking Environment and Human Factors, University of Dortmund, Germany; ^3^Rutgers University, Laboratory of Vision Research, 152 Frelinghuysen Road, Piscataway, NJ 08854-8020, USA

“Reverspectives” (by artist Patrick Hughes) consist of truncated pyramids with their smaller faces closer to the viewer such as to allow a realistic scene to be painted on them. With their pictorial perspectives that reverse the physical depth arrangement, reverspectives provide a bistable paradigm of competing depth percepts, with illusory depth being strongest monocularly and beyond (individually varying) critical viewing distances (Papathomas, Perception 2002). “Properspectives” are the same solid pyramids, painted on the inside, thus possessing a pictorial perspective that conforms with (and thus augments) real depth. Vergence eye movements were recorded (EyeLink II system) comparatively for monocular and binocular reverspective and binocular properspective conditions (optimized to obtain illusory, illusory/ veridical, or veridical depth percepts, respectively). Gaze locations were signaled by LEDs, inserted in 8 positions on the model. While fixating identical LED-positions, the eyes converged with veridical depth percepts and diverged with illusory depth percepts. These results indicate that pictorial cues are as effective as physical cues for vergence control.

# Abstract: Involvement of plasminogen activator systemin immunomoudulation of experimental autoimmune myasthenia gravis

**Authors:** H. Wald, C. Sicsic, M. Irony-Tur-Sinai, S. Akkawi, A. E. Higazi, T. Brenner

**Affiliations:** ^1^Department of neurology, Hadassah Hebrew University Medical Center, Jerusalem, Israel; ^2^Department of biochemistry, Hadassah Hebrew University Medical Center, Jerusalem, Israel

Myasthenia gravis (MG) is an autoimmune disease characterized by muscle weakness caused by antibodies to the nicotinic acetylcholine receptor (nAChR) at the post-synaptic site of the neuromuscular junction (NMJ). Experimental autoimmune MG (EAMG), the animal model of MG, whose chronic phase closely resembles human MG, is widely used to study numerous aspects of MG. The plasminogen activator (PA) system and matrix metalloproteinases (MMP) are extracellular proteolytic enzymes which modulate cell migration, cell-matrix interactions and signaling pathways. It is also known that inflammatory cells express the uPA receptor (UPAR) and that tissue PA (tPA) and urokinase PA (UPA) activities are elevated in inflammatory areas. A part from exploring the inflammatory aspect of the myasthenic NMJ and based on our previous findings on the involvement of the PA system in CNS autoimmune conditions, in the present study we tested the association of the PA system with EAMG pathogenesis. EAMG was induced in female C57bl mice by immunization with purified Torpedo AChR. Four groups were used: mice genetically deficient in UPA, UPAR, and tPA and wild type (wt). In comparison to the wt all the knock out (ko) mice developed a more severe disease: disease incidence was 30% in the control wt group, versus 60%, 100% and 80% in the UPA−/−, UPAR−/− and tPA−/− groups respectively. However, lymphocyte reactivity towards mitogens was reduced in cells derived from all the ko mice. The addition of peptides derived from PAI-1 increased T-cell reactivity in vitro by 20–70%, whereas, tPA caused inhibition. Our results imply that a network of functionally redundant proteases is involved in EAMG pathogenesis. Modulation of the PA system could serve as a potential target for treatment of EAMG and MG.

# Abstract: ARTS structure and expression in rat cortical neurons

**Authors:** N. Weinstein, J. P. Finberg

**Affiliations:** Molecular Pharmacology Department, Rappaport Faculty of Medicine,Technion, Haifa, Israel

The pro-apoptotic protein ARTS is a member of the septin family, but is derived by alternate splicing from the H5/PNUTL2/hcdcrel2a/2b gene and differs in its C-terminal from other products of this gene. ARTS promotes apoptosis induced by a variety of apoptotic stimulators. In cells of human peripheral tissue origin, ARTS is located in the mitochondria and translocates to the nucleus at the start of apoptosis, but in rat cortical neurons ARTS is localized in both cytoplasm and nucleus. ARTS induces apoptosis by binding to and antagonizing IAPs (inhibitors of apoptosis proteins), thus removing caspase inhibition and activating the apoptotic cascade. ARTS is strongly expressed in the brain and may play an important role in the nervous system. The ARTS protein in peripheral cells has a molecular size on gel electrophoresis of 32 kDa in human and 28 kDa in rat. However ARTS in human brain tissue has a molecular weight of 28 kDa and 22 kDa in rat brain tissue. We have sequenced the ARTS cDNA from rat brain. Alignment between the putative amino acid sequences of human and rat ARTS shows 83% identity. Rat brain ARTS lacks 5 amino acids in the Nterminal region, a 14 amino acid sequence between amino acids 76 and 89 and 3 amino acids between 123–125. The effect of a few apoptotic stimuli were tested on rat E18 cortical primary neuronal cells for their effect on the expression of ARTS and active caspase-3 (apoptosis indicator). Neurons were grown in Neurobasal medium containing B- 27 additives. Removal of B-27 at DIV 7 induced apoptosis. Western blot showed an increase in ARTS protein expression in cells which were incubated 24 h in medium without B27. High concentrations of glutamate (1–75 mM) also induced apoptosis. At 50 mM, glutamate increased expression of ARTS and active caspase-3 (*P* .05). ARTS may be involved in neuronal apoptosis, but its precise role is still unclear.The pro-apoptotic protein ARTS is a member of the septin family, but is derived by alternate splicing from the H5/PNUTL2/hcdcrel2a/2b gene and differs in its C-terminal from other products of this gene. ARTS promotes apoptosis induced by a variety of apoptotic stimulators. In cells of human peripheral tissue origin, ARTS is located in the mitochondria and translocates to the nucleus at the start of apoptosis, but in rat cortical neurons ARTS is localized in both cytoplasm and nucleus. ARTS induces apoptosis by binding to and antagonizing IAPs (inhibitors of apoptosis proteins), thus removing caspase inhibition and activating the apoptotic cascade. ARTS is strongly expressed in the brain and may play an important role in the nervous system. The ARTS protein in peripheral cells has a molecular size on gel electrophoresis of 32 kDa in human and 28 kDa in rat. However ARTS in human brain tissue has a molecular weight of 28 kDa and 22 kDa in rat brain tissue. We have sequenced the ARTS cDNA from rat brain. Alignment between the putative amino acid sequences of human and rat ARTS shows 83% identity. Rat brain ARTS lacks 5 amino acids in the Nterminal region, a 14 amino acid sequence between amino acids 76 and 89 and 3 amino acids between 123–125. The effect of a few apoptotic stimuli were tested on rat E18 cortical primary neuronal cells for their effect on the expression of ARTS and active caspase-3 (apoptosis indicator). Neurons were grown in Neurobasal medium containing B- 27 additives. Removal of B-27 at DIV 7 induced apoptosis. Western blot showed an increase in ARTS protein expression in cells which were incubated 24 h in medium without B27. High concentrations of glutamate (1–75 mM) also induced apoptosis. At 50 mM, glutamate increased expression of ARTS and active caspase-3 (*P* < .05). ARTS may be involved in neuronal apoptosis, but its precise role is still unclear.

# Abstract: Expression level-dependent changes in GIRK1/2 activity in Xenopus laevis oocytes: amodeling study

**Authors:** D. Yakubovich, N. Dascal

**Affiliations:** Department of Physiology and Pharmacology, Sackler School of Medicine, Tel Aviv University, Israel

Increase of injectedm-RNA concentration of GIRK1/2 channel in Xenopus laevis oocytes causes increase in basal (agonist–independent) and either no change or decrease in evoked (agonist-induced) activity in whole cell two-electrode voltage clamp experiments. The same phenomenon is observed when the channels are activated in a receptorindependent manner, by co-expression of G[betagamma] subunits or by application of G[betagamma] to bath solution in excised patches. These changes are completely reversed by co-expression of G[alpha]i subunits. Paradoxically, the total activity (sun of basal and evoked activity) remains unchanged. In contrast, coexpression of phosducin (common G[betagamma] scavenger molecule) reduced the total activity. Biochemical data suggested that the rise in GIRK1/2 density is accompanied by increase in the concentration of G protein subunits (mostly G[betagamma]) in plasma membrane (co-trafficking). We developed a kinetic model of GIRK1/2 activation by G[betagamma] which accounts for the activation of the channel via G protein cycle. This model is based on the idea of graded contribution of each G[betagamma] liganded GIRK monomer to channel opening. The model renders sufficiently fast activation rate by agonist application (tau of 1-2 s). We assumed cotrafficking of GIRK1/2 with different amounts of G protein subunits to the membrane and simulated the channel activity with different channel concentrations inmembrane (density effect). The model reasonably reproduced the observed data (changes in basal, evoked, total activities and the activation ratio). However, the model failed to account for the difference observed between the co-expression of G[alpha]i and phosducin. This fact points towards additional, possibly allosteric, function of G[alpha]i subunit in modulation of GIRK channel activity.

# Abstract: A Complex Norepinephrine and Glucocorticoid Intercellular Interaction: Relevance to Plasticity Hypothesis of Depression

**Authors:** Yaniv S. P, E. Klein, D. Ben-Shachar

**Affiliations:** ^1^Laboratory of Psychobiology, B. Rappaport Faculty of Medicine, Technion, Israel; ^2^Department of Psychiatry, Rambam Medical Center, Israel

The plasticity hypothesis of major depression states that glucocorticoids (GCs)may be detrimental to neuronal plasticity while monoamines, as well as antidepressants, may reconstitute cellular plasticity. Given the key role norepinephrine (NE) and GC play in stress and depression, our aim is to search for a shared NE and GC intracellular pathway affecting plasticity with relevance to depression and its treatment. In SH-SY5Y human neuroblastoma cells, DEX, a synthetic GC receptor agonist, altered cellular morphology opposite to the differentiative effects of NE. This was accompanied by a reduction in mRNA and protein expression of the differentiation markers Gap-43 and L1 as well as protein levels of an additional marker laminin. Down-regulation was also observed in the transcription factor CREB and in phosphorylated CREB (pCREB). NE treatment increased Gap-43, L1 and laminin protein and mRNA levels as well as pCREB protein levels. The opposite effects of NE and DEX on identical genes suggest a common signaling pathway. The MAPK induced transcription factor AP-1 (c-Jun/c-Fos) can modulate the aforementioned genes. Both ERK and c-Jun were activated following NE treatment, while DEX did not affect these proteins yet reduced c-Fos expression. Co-administration of DEX and NE resulted in over-activation of the ERK pathway, associated with an increase p-c-Jun and c-Fos, and was accompanied by differentiative morphology. In addition, coadministration, as well as NE, increased AP-1 activity. Furthermore, in these cells expressing solely the Gi coupled alpha2 adrenergic receptor, pCREB was hyper-activated in an ERK independent manner. These results suggest AP-1 as a convergent point for NE and DEX induced signaling pathways. Elucidating a pathway affected by stress and antidepressants, represented by DEX and NE, respectively, involved in neuronal plasticity may contribute to understanding the biological basis of depression, its etiology and treatment.

*Supported by Israel Ministry of Health.*

# Abstract: Is conductance change involved in the shift between UP and DOWN states of rat neocortical neurons?

**Authors:** A. Yaron-Jakoubovitch, C. Koch, I. Segev, Y. Yarom

**Affiliations:** ^1^Department of Neurobiology, The Hebrew University, Jerusalem, Israel; ^2^The Interdisciplinary Center for Neural Computation, The Hebrew University, Jerusalem, Israel; ^3^Computation and Neural Systems Program, California Institute of Technology, Pasadena, Calif, USA

Recently, with the advance of intracellular recording, it was found that neurons in the mammalian cortex exhibit spontaneous transition in membrane potential from UP to DOWN states. It has been suggested that these oscillatory fluctuations in membrane potential, which are the cellular manifestation of the brain’s rhythmic activity, reflect network- rather than cellular-properties. However, the biophysical mechanisms underlying these network oscillations are still debated. Using an in-vivo whole cell technique, we recorded from 56 pyramidal neurons in the prefrontal and somatosensory cortices of adult rats under Ketamine-Xylazine anesthesia. The membrane potential of the cells displayed rhythmic alternations (average frequency of 3 Hz), between high and low levels of membrane potentials and were manifested as a prominent peak in the voltage spectrogram. Overshooting action potentials occurred solely during the depolarized levels. The distributions of the recorded voltage were either unimodal (with a prolonged tail) or bimodal. DC current injection into cells that showed bimodal distribution revealed that, in 37% of these cells, the voltage distribution was independent of the average membrane potential. In the remaining cells, changing the holding potential significantly alters the voltage distribution, increasing the voltage difference between the two peaks of the histogram upon hyperpolarization. This effect on the voltage distribution can only be accounted for if each of the two peaks represents a different conductance state. Indeed, an average 54% change in input conductance between the UP and DOWN states was calculated for 10 cells. The similar dependence of the two states on the current injection can be explained by assuming that the location of the conductance change that generates the UP and/or DOWN state is spatially remote from the recording electrode. Hence the integrative capabilities of a population of cortical neurons can be independent of their state.

# Abstract: The impact of geometry in shaping exploratory behavior in rats: interpretation in terms of urban planning

**Authors:** O. Yaski, D. Eilam

**Affiliations:** Department of Zoology, Faculty of Life Sciences, Tel Aviv University

In order to study the impact of the surrounding landscape on exploratory behavior of rats, two experiments were conducted. In the first rats were tested in a square arena with additional free-standing (portable) corners, featuring the same properties as the arena corners. It was found that the routes of progression converged upon the added corners probably since the added corners were distinct against the background of the arena enclosure. This implies that the four arena corners and walls were encoded as one geometric module, and the distinctness of the added corners made them a target of exploration. Thus, the impact of a corner extended beyond the specific self-geometry and depends on the background environment. In the second experiment rats were introduced into either a round or a square arena with various arrays of free-standing corners that differed in spacing, orientation, and number. The rats were able to detect the geometry of the enclosure, the array, and the landmarks: they favored the added corners particularly in the round compared with the square arena, and stayed at the interior of the added corner regardless of their orientation in the arena. Only geometric changes in landmark array affected rats' level of activity. It is suggested that the context and geometry of the environment affects the distinctness and the meaning of landmarks for the observer, which in turn results in a different distribution of activity. Altogether, the two experiments illustrate that the impact of the environment on exploratory behavior of rats resembles that of environmental elements in urban environments. We suggest that principles involved in shaping the image of a city in urban planning may be applicable for understanding how the physical structure of the environments shapes exploration and navigation in animals.

# Abstract: Natural developmental optical astigmatism during childhood induces “cortical astigmatism” in the adult visual cortex

**Authors:** O. Yehezkel, U. Polat

**Affiliations:** Sackler Faculty of Medicine, Tel Aviv University, Goldschleger Eye Research Institute, Tel Hashomer, Israel

Maturation of neural connectivity is experience dependent in such a way that the state of the visual input during the critical period can be inferred fromthe visual functions in adults. In meridional amblyopia, the visual input during the critical period was asymmetrical; hence, the perception in one meridian was blurred, whereas the orthogonal one was normal. Consequently, in adults, the contrast sensitivity and the lateral interactions are dramatically reduced in the blurred meridian, whereas they are practically normal in the orthogonal one. However, asymmetric visual input is common during normal development and children below four years of age tend to be astigmatic (with the vertical meridian being more blurred than the horizontal meridian). However, this effect decreases and usually disappears with increasing age. Here we explored whether this visual asymmetry during the critical period, shaped the development of the visual connectivity and induced cortical asymmetry that was retained during adulthood in people with normal vision (i.e., better development of the horizontal meridian). We tested subjects with corrected vision of 6/6 or better (*n* = 11). Contrast sensitivity (CS) to Gabor patches (GP) was measured for a few spatial frequencies (3, 6, 9, and 12 cpd) under monocular conditions. We also measured the monocular collinear facilitation (9 cpd, target-flanker separation of 3 lambada). We found a significant asymmetry in monocular CS; it was better in the horizontal meridian. The facilitation was significantly different between the two meridians regardless of the contrast thresholds of the single target. Our results show that neural connectivity in the normal visual cortex of adults is asymmetric, displaying “cortical astigmatism,” thus mirroring the early optical asymmetry during the developmental period in childhood.

*Supported by a grant from the Israel Science Foundation.*

# Abstract: Working memory across nostrils

**Authors:** Y. Yeshurun, Y. Dudai, N. Sobel

**Affiliations:** Department of Neurobiology, TheWeizmann Institute of Science, Rehovot 76110, Israel

Olfactory working memory (OWM) maintains information over short information over short time frames. Whether this involves verbal representations or neural images of odor per se, is unknown. Also, the contribution of primary olfactory structures versus higher-order brain systems is unclear. To address this, we set out to test OWM across nostrils. Considering the predominantly ipsilateral nature of olfactory projections, we hypothesized that nonverbal OWM images would result in no effect of nameability and better overall performance within the same nostril, and in contrast, that verbal OWM images would result in a main effect of nameability, and a possible left nostril advantage. Twentyone subjects participated in a button-press delayed-matchto- sample task (trials = 96, ISI = 12 s, ITI = 30 s), with 10 nameable and 10 unnameable odorants, chosen per subject. In 1/3 of the trials “sample” and “match” were presented in the same nostril, 1/3 of the trials “sample” was presented in one nostril, and “match” in the other, and in 1/3 of the trials “sample” and “match” were delivered to both nostrils. Odorant order was randomized, and test side was counterbalanced. Presented results refer to trials where “sample” and “match” were the same. There was a nameability main effect (F(1,20) = 8.75, *P* < 0.0077), reflecting more mistakes when the odorants were not nameable. Reaction time (RT) measurements revealed a nostril-side main effect (F(2,40) = 5.1, *p* < 0.01), reflecting overall longer RT when the odor was delivered across nostrils then in both nostrils, and a nameability by side interaction (F(2,40) = 6.33,*P* < .004) implying that the nameability effect (longer RT for unnameable odors) was more pronounced when subjects were tested across nostrils (*P* < .015). These findings imply dual representations in OWM: when a verbal representation is available, it is equally accessible to both nostrils, but when an odor image is remembered, it offers preferred access to the nostril that generated it.

# Abstract: The endocannabinoid-like N-arachidonoyl-L-serine improves outcome after traumatic brain injury

**Authors:** A. Yeshurun-Cohen, R. Leker, A. Alexandrocivh, R. Mechoulam, E. Shohami

**Affiliations:** ^1^Department of Pharmacology, The Hebrew University School of Pharmacy, Jerusalem, Israel; ^2^Department of Medicinal Chemistry, The Hebrew Unviersity Faculy of Medicine, Jeursalem, Israel; ^3^Department of Neurology, Hadassah University Hospital, The Hebrew University, Jerusalem, Israel

**Background**

The endocannabinoid 2-arachidonoyl-glycerol (2-AG) was shown to afford neuroprotection via CB1-receptor mechanisms. Recently a non-fCB1, non-CB2 endocannabinoid-like N-arachidonoyl-L-serine (AraS) was found in the brain. It is an endothelium-dependent vasodilator with antiapoptotic effects, and was shown to stimulate Akt phosphorylation in human umbilical vein endothelial cells and suppress LPSinduced TNFalfa in macrophages. We here compared the effects of this compound after traumatic brain injury (TBI) to those of 2-AG.

**Methods**

Mice were subjected to TBI and treated 1 h later with either AraS, 2-AG or vehicle. Neurobehavioral function of the injured mice was assessed by the Neurological Severity Score (NSS), and lesion volume was evaluated by staining with 2,3,5-triphenyltetrazolium chloride, that binds to viable mitochondria. In addition, the effect on the proliferation of neural progenitor cells was assessed in-vitro by the size of neurospheres isolated from embrional mouse brain tissue (E15) and grown for 4d, using the Image-Pro-Plus software.

**Results**

During 90d after injury the neurobehavioral outcome of the mice treated with 2-AG or AraS was significantly better than that of the vehicle-treated mice. In addition, lesion volume measured 24 hours after trauma was smaller in the groups subjected to either 2-AG or AraS compared to vehicle. Moreover, preliminary experiments showed that exposure of neurospheres, consisted of multipotential neural precursor cells to AraS led to a 2 to 4-fold increase in the size of the treated spheres after 4d as compared to untreated ones.

**Conclusion**

The endocannabinoid-like AraS, similarly to the 2-AG, may act as a neuroprotectant after TBI by reducing infarct volume, facilitating long-term function recovery and probably also by promoting proliferation of neural precursor cells. The cellular mechanisms of these effects are currently being investigated.

*Supported by grants from AMRF/APNRR and from NIDA no. DA9289.*

# Abstract: Dynamic changes in DNA CpG dinucleotide methylation in the brain-derived neurotrophic factor (BDNF) promoter area during thermal control establishment

**Authors:** M. Yossifoff, T. Kisliouk, N. Meiri

**Affiliations:** Institute of Animal Science, ARO, The Volcani Center, Bet Dagan 50250, Israel

The critical period for thermal control establishment in chicks spans between postnatal days three and five. Heat conditioning of chicks during that time frame enables them to be more tolerant to heat later in life. This modulation is thought to be carried out by alteration of neuronal properties in the preoptic anterior hypothalamus (PO/AH) which is probably realized by the subset of proteins which are expressed. Epigenetic mechanisms such as DNA methylation are emerging as important regulators of neuronal gene expression. Hence the present study analyzed the patterns of methylation of the promoter of a known regulator of neuronal plasticity, the brain-derived neurotrophic factor (BDNF), during a 24 hour heat conditioning of chicks on the third postnatal day and during 24 hours of thermal challenge a week later. The experiments looked at the expression of the BDNF gene along with the levels of DNA methylation of 6 different CpG positions on the BDNF promoter at different time points during heat conditioning and challenge (day 10). Our data show a transient increase in BDNF expression during heat conditioning which peaked 6 and 12 hours into conditioning. Concurrently, there was a transient induction of methylation of two CpG positions (the first and the third CpG location downstream from the BDNF-ATG) and a reduction of methylation of one CpG position (1000 bp downstream from the BDNFATG), while the other positions did not show significant change. Furthermore, our data demonstrate a significant difference between the levels of methylation of the BDNF promoter of conditioned compared to nonconditioned chicks during a thermal challenge, which correlates with the different levels of BDNF in the two groups. These data suggest that complex and dynamic changes in DNA methylation might be involved in the regulation of thermotolerance acquisition and support the role of DNA methylation in the regulation of gene expression during neuronal plasticity.

# Abstract: Coding selectivity of object location in the secondary somatosensory cortex during active vibrissal touch

**Authors:** C. Yu, S. Haidarliu, D. Derdikman, N. Rubin, E. Ahissar

**Affiliations:** Department of Neurobiology, Weizmann Institute of Science, Israel

Sensation is an active process. When the sensors move, both motion and touch signals are crucial for sensory processing. Recently, we showed that whisker-motion and contact signals are conveyed in parallel via the thalamus. Yet, it is unknown how downstream brain areas process these signals to localize objects. Specifically, the function of secondary somatosensory cortex (S2), which receives massive thalamic projections from both whisker-motion and contact pathways, is poorly understood. Here, neuronal activities from S2 in response to object touch were recorded using artificial whisking in anesthetized rats. We found that high proportion of neurons (41%) in deeper layers (layers 4, 5 and 6) of S2 (S2-L46) show significant selectivity to object location, in distinction with the superficial layers (layers 2/3) of S2 (20%). Similar to the latter, small fractions (<23%) of neurons in three thalamic nuclei (POm, VPMdm, VPMvl) and three layers (2/3, 4 and 5a) of the primary somatosensory cortex (S1) displayed significant selectivity to object location. We further investigated the dynamics of responses in all these somatosensory sites during whisking in free air and against objects in various locations. Comparison of the dynamics of location-selective (S) cells with those of nonselective cells revealed several differences. Among these differences was a lack of a slow-wave dynamics in S cells, different response dynamics during retraction, and a weaker response of S cells to the first cycle in VPMdm and S1-L4. Comparison of response dynamics across nuclei revealed two major patterns of cortical dynamics, one evident in S2-L46 and S1-L5a and another in S2- L23 and S1L4. The analysis so far thus suggests that cortical processing of active touch is done in parallel in at least two streams, that processing of object location involves several brain sites, among which a significant processing of object location is performed in the deep layers of S2.

# Abstract: Induced gamma in the eye of the beholder: single trial analysis of induced gamma band responses in human observers

**Authors:** S. Yuval-Greenberg, O. Tomer, L. Y. Deouell

**Affiliations:** ^1^Department of Psychology, The Hebrew University of Jerusalem, Israel; ^2^The Interdesciplinary Center for Neural Computation, The Hebrew University of Jerusalem, Israel; ^3^Department of Neurobiology, The Hebrew University of Jerusalem, Israel

In the last decade a large amount of electrophysiological research has focused on high frequency brain activity in humans, and specifically on the “induced gamma response” (iGBR). This response is a typical increase in the gamma band (>30 Hz) peaking around 250 milliseconds post stimulus, following the presentation of a visual stimulus. The iGBR was hypothesized to represent the neural synchronization involved in various cognitive functions such as binding and consciousness. The iGBR is calculated by averaging single trials’ spectral data, circumventing phase cancellation due to inter-trial latency jitter Our study is the first attempt to look at gamma responses in single trials. Subjects performed a visual paradigm in which all subjects demonstrated a prominent iGBR. In each subject many of the single trials exhibit a gamma increase at a similar latency, topography and frequencies as the average iGBR. Examination of the EEG data shows that each gamma increase is characterized by a sharp peak in the filtered data (30–100 Hz) of central channels. In addition, a prominent peak with an opposite polarity appears at the EOG channels placed around the eyes. In a simultaneous recording of eye position (using an eye tracker device) it was found that these gamma increases are frequently accompanied by an eye movement. As a further analysis, trials were sorted according to eye movements in the relevant time window. Averaging across the third of trials with the strongest eye movements, the iGBR was exceptionally high. In contrast, when the average was performed across a third of trials showing the least eye movements, the iGBR was eliminated. The relation between eye movements and gamma power was further demonstrated when sorting by gamma power and examining the eye movements. These findings show a strong relation between iGBR and eye movements, challenging prevailing models of the neural origin of the scalp recorded iGBR and the related cognitive processes.

# Abstract: Circuitry underlying perceptual learning in behaving monkeys

**Authors:** N. Zach, D. Inbar, Y. Grinvald, E. Vaadia

**Affiliations:** ^1^Physiology Department, Hadassah Medical School, Hebrew University Jerusalem, Israel; ^2^ICNC, Interdisciplinary Center for Neural Computation, Hebrew University Jerusalem, Israel

Learning new sensorimotor tasks involves plastic changes in responses of the primary motor cortex (M1) and the Premotor area (PM). We have shown such changes accompanying both the learning process and the consolidation into long term memory that follows learning. Since contextual information has been shown to affect the learning of various sensorimotor tasks, we investigated the effect of context on neuronal response in the motor cortices. In order to do so, we simultaneously recorded neuronal activity from 32 electrodes in M1 and Premotor cortices of two behaving monkeys. In each session, the monkeys performed first a center-out task and then, they had to learn a novel arbitrary associations task (where they had to learned a color-direction association), and then they performed a second block of the centerout task. We compared neuronal responses during trials featuring the same movement direction and target color, either made during the center-out task or during the arbitrary association task (only trials of stable plateau performance was included). Also, we compared the two center-out blocks to eliminate effects of passage of time. We found that about 65% of the neurons showed significant difference in responses when the very similarmovements made in different contexts. Differences included both firing rate change and change in response pattern. These differences could not be accounted for by any change in movement parameters. These results demonstrate that the motor cortices are liable to contextual modulation, and broaden our knowledge on motor cortices dynamics.

# Abstract: Capacity of binned neural channels during experiments with brain-machine-interfaces

**Authors:** M. Zacksenhouse, M. Lebedev, M. Nicolelis

**Affiliations:** ^1^Faculty of Mechanical Engineering, Technion-IIT, Haifa, Israel; ^2^Center for Neuroengineering, Duke University, Durham, NC, USA

Brain machine interfaces extract relevant information, like the velocity of the intended movement, from recorded neural activity. However, while the recorded neural activity is in the form of spike trains, most decoding algorithms are based on the binned spike counts. The bin-width (BW) is usually determined by trial and error or based on previous experience. Here we investigate the effect of the bin-width on the signal-to-noise ratio (SNR), and on the ratio SNR/BW. The latter is shown to be related to the capacity of the neural channel under different assumptions. Furthermore, while the SNR increases with the BW, the update rate decreases, suggesting that the ratio SNR/BW should be considered as a relevant criterion. The SNR is estimated under the assumption that the neural spike trains are realization of doubly stochastic Poisson processes–the simplest point processes that can encode stochastic signals. Analysis of neural spiketrains recorded during BMI experiments from different cortical areas, indicate that the mean SNR/BWhad a broad peak around 100 milliseconds—the bin-width that was selected by trial and error. However, detailed analysis indicates that the SNR/BW of different neurons peak at different bin-widths. In particular, the SNR/BW of M1 neurons peak at shorter bin-widths, while that of PMd neurons peak at longer binwidths.

*Supported by Fund for Promotion of Research at the Technion, DARPA, NIH, NSF*.

# Abstract: Adaptive motor control during experiments with BrainMachine Interfaces

**Authors:** M. Zacksenhouse, K. Beiser

**Affiliations:** Faculty of Mechanical Engineering, Technion-IIT, Haifa, Israel

Recent experiments with Brain Machine Interfaces indicate that the extent of neural modulations increases abruptly upon starting to operate the interface. In contrast, neural modulations due to the trajectory profile remain relatively unchanged. Furthermore, the enhanced modulations subside with further training, mirroring the trend in task performance, which degraded upon starting to operate the interface and improved gradually with training. Here we investigate the hypothesis that the enhanced modulations reflect internal representation of trajectory errors, which results in corrective commands in the short term and adaptive modifications of internal models in the long term. A simplified unidimensional model is analyzed to demonstrate the observed transient enhancement in neural modulations during the operation of Brain machine Interfaces. Identifying the source of the transient enhancement in neural modulation would provide insight into adaptive motor control and facilitate the improvement of future Brain Machine Interfaces. Analysis of the model yields the conditions under which it is stable, reduces the motor error and acquires the correct inverse internal model/feedforward controller. Simulations of both skilled and BMI operations, using parameters and signals from experiments, yield results with similar characteristics as those observed in experiments in regards to both tracking performance and to neuronal modulation levels. Our model gives rise to the hypothesis that motor neurons are tuned both to preferred desired directions and preferred error directions, further more, it stresses the importance of adequate tuning to error directions without which the model is not stable and unable to reduce the motor error by feedback control or to adapt the correct internal inverse model that will perform as feedforward controller.

*Fund for Promotion of Research at the Technion.*

# Abstract: The correlation between the Translocator Protein (TSPO) and apoptosis in human glioma cells under hypoxia

**Authors:** S. Zeno, L. Veenman, S. Leschiner, M. Zaarur, M. Gavish

**Affiliations:** ^1^Neurology, Medical Faculty, Technion-Israel Institute of Technology, 31096 Haifa, Israel; ^2^Rambam Medical Center, 31096 Haifa, Israel

The translocator protein (TSPO) can be found in conjunction with the mitochondrial 32 kDa voltage-dependent anion channel (VDAC) and the 30 kDa adenine nucleotide transporter (ANT). The VDAC and ANT form the core elements of the mitochondrial permeability transition pore (MPTP). Opening of the MPTP can start the mitochondrial apoptosis cascade. Furthermore, the TSPO has been suggested to act as an oxygen sensor. Oxygen related mechanisms induce prominent clinically relevant changes in cancer cells and tumor biology through the control of gene expression. Therefore, we studied the levels of TSPO and apoptosis in glioma cells under hypoxic conditions. To do this, we applied either an environment with 1% oxygen (hypoxia) or added CoCl2 to the medium (0.25 mM, 0.4 mM, and 1 mM). Treatments with 1% oxygen resulted in an increase in the levels of TSPO (Western) and an increase in TSPO binding density. Furthermore, 1% oxygen levels resulted in an increase in apoptosis levels (50%). Adding CoCl2 (0.25 mM) to the cells also resulted in an increase in apoptosis levels (40%). However, the binding density of TSPO was unchanged with application of CoCl2. We also found that under treatment with CoCl2, the amount of P53 is decreased, suggesting a deregulation of the cell cycle. Stabilization of HIF1-α level indicated that in our model CoCl2 (0.25 mM) treatment indeed mimics hypoxia conditions. Our results indicate that reduced oxygen levels affect both TSPO levels and apoptosis, while CoCl2 only affects apoptosis. Other preliminary results, from TSPO knockdown with siRNA, suggest however that CoCl2 also induces apoptosis via TSPO. These results suggest that TSPO as an oxygen sensor may play a role in the induction of apoptosis levels due reduced oxygen levels. This may have implications for our understanding of the involvement of TSPO in pathological conditions such as ischemia and cancer.

*Research support: WK, LV, ML, and MG (Joint Lower Saxony- Israeli Research Projects, VWZN2047).*

# Abstract: Inferring cognitive states from magnetoencephalographic (MEG) signals: feature selection framework

**Authors:** A. Zhdanov, T. Hendler, L. Ungerleider, N. Intrator

**Affiliations:** ^1^Functional Brain Imaging Unit, Tel Aviv Sourasky Medical Center, Israel; ^2^Psychology Department and Faculty of Medicine, Tel Aviv University, Israel; ^3^Lab of Brain and Cognition, NIMH, NIH, Bethesda, USA; ^4^School of Computer Science, Tel Aviv University, Israel

We develop a robust linear classification framework for inferring mental states from electrophysiological (MEG and EEG) signals. The framework is centered around the concept of temporal evolution of regularized Fisher Linear Discriminant classifier constructed from the instantaneous signal value. The value of the regularization parameter is selected to minimize the classifier error estimated by cross-validation. We build upon the proposed framework to develop a feature selection technique. We demonstrate the framework and the feature selection technique on MEG data recorded from 10 subjects in a simple visual classification experimente show that using a very simple adaptive feature selection strategy yields considerable improvement of classifier accuracy over the strategy that uses fixed number of features.

*We develop a robust linear classification framework for inferring mental states from electrophysiological (MEG and EEG) signals. The framework is centered around the concept of temporal evolution of regularized Fisher Linear Discriminant classifier constructed from the instantaneous signal value. The value of the regularization parameter is selected to minimize the classifier error estimated by cross-validation. We build upon the proposed framework to develop a feature selection technique. We demonstrate the framework and the feature selection technique on MEG data recorded from 10 subjects in a simple visual classification experiment. We show that using a very simple adaptive feature selection strategy yields considerable improvement of classifier accuracy over the strategy that uses fixed number of features.*

# Abstract: Do you rememberme? retrieving memories for faces—evidence from theta (4–8Hz) and gamma band (>30 Hz) Activity

**Authors:** E. Zion Golumbic, M. Kutas, S. Bentin

**Affiliations:** ^1^Department of Cognitive Science, The Hebrew University of Jerusalem, Israel; ^2^Department of Cognitive Science, University of California, San Diego, Calif, USA; ^3^Department of Psychology, The Hebrew University of Jerusalem, Israel; ^4^Center for Neural Computation, The Hebrew University of Jerusalem, Israel

Social interaction depends on our ability to create, store and retrieve memories for human faces. Learning new faces is unique not only due to the special nature of face processing but also because it requires forming detailed neural representations. To date few studies have investigated the neural mechanism involved in learning new faces. In the present study subjects studied a series of faces and were then tested on their memory for them when presented again interspersed among new faces. Both famous and nonfamous faces were used, in separate blocks, in order to compare memory for faces which subjects have prior experience with and new faces which subject have no previous representation for. EEG was recorded and we focused primarily on two electrophysiological measures of neural activity: theta-band activity (4- 8Hz) which has been associated with successful encoding and retrieval of memories and Gamma-band activity (>30 Hz) which has been linked to activating a neural representation matching an incoming stimulus. We compared activity during the test phase in response to correctly remembered faces (hits) with correctly categorized new faces (CR). Theta activity was similar for both famous and nonfamous faces and was larger for hits than for CRs. In contrast, Gamma activity was larger for famous faces than for nonfamous faces. Hits elicited smaller Gamma amplitudes than CR, which is in line with previous studies demonstrating a reduction in Gamma activity for repeated stimuli and has been attributed it to a “sharpening” of the cortical representation activated by the stimulus. This study demonstrates that both Theta and gamma activity play a role in retrieval of memories for faces, and suggests that whereas Theta activity is related to mnemonic processing irrespective of pre-experimental familiarity with the stimuli, Gamma activity is involved in the activation of neural representations and is modulated by recent exposure to the same stimulus.

# Abstract: Proteolysis and membrane capture of F-spondin generates combinatorial guidance cues from a singlemolecule

**Authors:** S. Zisman, K. Marom, O. Avraham, A. Klar

**Affiliations:** Department of Anatomy and Cell Biology, Hebrew University, Hadassah Medical School, Jerusalem, Israel

The formation of neuronal networks is governed by a limited number of guidance molecules, yet it is immensely complex. The complexity of guidance cues is augmented by posttranslational modification of guidance molecules and their receptors. We report here that cleavage of the floor plate guidance molecule F-spondin generates two functionally opposing fragments: a short-range repellent protein deposited in the membrane of floor plate cells, and an adhesive protein that accumulates at the basement membrane. Their coordinated activity, acting respectively as a short-range repellant and a permissive short-range attractant, constricts commissural axons to the basement membrane beneath the floor plate cells. We further demonstrate that the repulsive activity of the inhibitory fragment of F-spondin requires its presentation by LRP receptors ApoER2, LRP2/megalin and LRP4, which are expressed in the floor plate. Thus, proteolysis and membrane interaction coordinate combinatorial guidance signaling originating from a single guidance cue.

# Abstract: Early perceptual loss in depression

**Authors:** A. Zomet, R. Amiaz, U. Polat

**Affiliations:** ^1^Goldschleger Eye Research Institute, Tel-Aviv University; ^2^Department of Psychiatry, Israel; ^3^Sheba Medical Center, 52621 Tel Hashomer, Israel

In our previous study, we found that the filling-in process is impaired in patients with depression, as evident by their reporting less target present than the control group. We suggested that this deficit is due to an abnormal balance of the excitation and inhibition between neurons. However, it is still possible that the response criterion in these subjects is more conservative, due to general indifference. In this study, we used a forced choice task (to neutralize the internal criterion), and a static presentation (no limitation of information processing time) to minimize the attentional effect. We measured the contrast threshold of a Gabor target that was presented at the left or the right side of the screen (1.2° each side) between two flankers (12 cpd), separated at 7 distances from 0 to 12 lambda. The short distances test contrast discrimination and the far distances contrast detection. We found that the function of the control group is similar to the standard function, found when using 2 temporal alternative forced choice (2AFC), showing suppression in 0 and 1 lambda and facilitation at the far distances. On the other hand, the depressed subjects performed like the control subjects only at the far distances, whereas at the short distances (contrast discrimination), the expected suppression is absent and is replaced by facilitation. Since suppression in contrast discrimination tasks is explained as largely due to response saturation of neurons, we suggest that high levels of inhibition prevent the neurons from reaching saturation. High levels of inhibition reduced the level of excitation, which in turn will lead to an impaired filling-in process in depressed subjects.

*Supported by the National Institute of Psychobiology in Israel.*
